# Disease consequences of higher adiposity uncoupled from its adverse metabolic effects using Mendelian randomisation

**DOI:** 10.7554/eLife.72452

**Published:** 2022-01-25

**Authors:** Susan Martin, Jessica Tyrrell, E Louise Thomas, Matthew J Bown, Andrew R Wood, Robin N Beaumont, Lam C Tsoi, Philip E Stuart, James T Elder, Philip Law, Richard Houlston, Christopher Kabrhel, Nikos Papadimitriou, Marc J Gunter, Caroline J Bull, Joshua A Bell, Emma E Vincent, Naveed Sattar, Malcolm G Dunlop, Ian PM Tomlinson, Sara Lindström, INVENT consortium, Jimmy D Bell, Timothy M Frayling, Hanieh Yaghootkar

**Affiliations:** 1 Institute of Biomedical and Clinical Science, University of Exeter Medical School, Research, Innovation, Learning and Development building, Royal Devon & Exeter Hospital Exeter United Kingdom; 2 Research Centre for Optimal Health, School of Life Sciences, University of Westminster London United Kingdom; 3 Department of Cardiovascular Sciences, University of Leicester Leicester United Kingdom; 4 NIHR Leicester Biomedical Research Centre Leicester United Kingdom; 5 Department of Dermatology, University of Michigan Ann Arbor United States; 6 Ann Arbor Veterans Affairs Hospital Ann Arbor United States; 7 The Institute of Cancer Research London United Kingdom; 8 Department of Emergency Medicine, Massachusetts General Hospital Boston United States; 9 Department of Emergency Medicine, Harvard Medical School Boston United States; 10 Nutrition and Metabolism Branch, International Agency for Research on Cancer Lyon France; 11 MRC Integrative Epidemiology Unit at the University of Bristol Bristol United Kingdom; 12 Population Health Sciences, Bristol Medical School, University of Bristol Bristol United Kingdom; 13 School of Cellular and Molecular Medicine, University of Bristol Bristol United Kingdom; 14 Institute of Cardiovascular and Medical Sciences, University of Glasgow Glasgow United Kingdom; 15 University of Edinburgh Edinburgh United Kingdom; 16 Western General Hospital Edinburgh United Kingdom; 17 Edinburgh Cancer Research Centre, IGMM, University of Edinburgh Edinburgh United Kingdom; 18 Department of Epidemiology, University of Washington Seattle United States; 19 Division of Public Health Sciences, Fred Hutchinson Cancer Research Center Seattle United States; 20 Centre for Inflammation Research and Translational Medicine (CIRTM), Department of Life Sciences, Brunel University London Uxbridge United Kingdom; University of Melbourne Australia; University of Zurich Switzerland

**Keywords:** Mendelian randomisation, obesity, favourable adiposity, cardiovascular disease, cancer, Human

## Abstract

**Background:**

Some individuals living with obesity may be relatively metabolically healthy, whilst others suffer from multiple conditions that may be linked to adverse metabolic effects or other factors. The extent to which the adverse metabolic component of obesity contributes to disease compared to the non-metabolic components is often uncertain. We aimed to use Mendelian randomisation (MR) and specific genetic variants to separately test the causal roles of higher adiposity with and without its adverse metabolic effects on diseases.

**Methods:**

We selected 37 chronic diseases associated with obesity and genetic variants associated with different aspects of excess weight. These genetic variants included those associated with metabolically ‘favourable adiposity’ (FA) and ‘unfavourable adiposity’ (UFA) that are both associated with higher adiposity but with opposite effects on metabolic risk. We used these variants and two sample MR to test the effects on the chronic diseases.

**Results:**

MR identified two sets of diseases. First, 11 conditions where the metabolic effect of higher adiposity is the likely primary cause of the disease. Here, MR with the FA and UFA genetics showed opposing effects on risk of disease: coronary artery disease, peripheral artery disease, hypertension, stroke, type 2 diabetes, polycystic ovary syndrome, heart failure, atrial fibrillation, chronic kidney disease, renal cancer, and gout. Second, 9 conditions where the non-metabolic effects of excess weight (e.g. mechanical effect) are likely a cause. Here, MR with the FA genetics, despite leading to lower metabolic risk, and MR with the UFA genetics, both indicated higher disease risk: osteoarthritis, rheumatoid arthritis, osteoporosis, gastro-oesophageal reflux disease, gallstones, adult-onset asthma, psoriasis, deep vein thrombosis, and venous thromboembolism.

**Conclusions:**

Our results assist in understanding the consequences of higher adiposity uncoupled from its adverse metabolic effects, including the risks to individuals with high body mass index who may be relatively metabolically healthy.

**Funding:**

Diabetes UK, UK Medical Research Council, World Cancer Research Fund, National Cancer Institute.

## Introduction

Obesity is associated with a higher risk of many diseases, notably metabolic conditions such as type 2 diabetes, but many individuals are often relatively metabolically healthy compared to others of similar body mass index (BMI). Whilst these metabolically healthier individuals may be at lower risk of some obesity-related conditions, they may be at risk of conditions that are linked to other aspects of obesity, such as the load-bearing effects. The burden of obesity on individuals and health-care systems is very large, and in the absence of a widely applicable, sustainable treatment or effective public health measures, it is important to understand the disease consequences of obesity, and how they may be best alleviated, in more detail.

To better understand the disease consequences of obesity, many previous studies have used the approach of Mendelian randomisation (MR) (Smith and Ebrahim, 2004). These studies used common genetic variants robustly associated with BMI as proxies for obesity to assess the causal effects of higher BMI on many diseases. MR studies have provided strong evidence that higher BMI leads to osteoarthritis (Tachmazidou et al., 2019), colorectal cancer (Thrift et al., 2015; Suzuki et al., 2021; Bull et al., 2020), and psoriasis (Budu-Aggrey et al., 2019), as well as metabolic conditions such as type 2 diabetes, cardiovascular disease (Hägg et al., 2015), and heart failure (Cheng et al., 2019; Corbin et al., 2016; Fall et al., 2013). Other MR studies indicate that higher BMI may lead to lower risk of some diseases, including postmenopausal breast cancer (Guo et al., 2016) and Parkinson’s disease (Noyce et al., 2017).

Obesity is heterogeneous – for example, for a given BMI, people vary widely in their amount of fat versus fat free mass, predominantly muscle, and their distribution of fat, predominantly subcutaneous versus ectopic and upper versus lower body fat. Even when there is strong evidence of causality, obesity may lead to disease through a variety of mechanisms. Despite many MR studies testing the role of higher BMI in disease, few have attempted to separate and test the different mechanisms that could lead from obesity to disease. Some MR studies have investigated the effects of fat distribution using genetic variants associated with waist-hip ratio (WHR) adjusted for BMI and shown that adverse fat distribution (more upper body, less lower body) leads to higher risk of metabolic disease (Emdin et al., 2017), some cancers (Cornish et al., 2020), and gastro-oesophageal reflux disease (Green et al., 2020).

Previous studies have identified genetic variants associated with more specific measures of adiposity. For example, several studies have characterised variants associated with ‘favourable adiposity’ (FA) or reduced adipose storage capacity using a variety of approaches (Ji et al., 2019; Lotta et al., 2017; Kilpeläinen et al., 2011; Huang et al., 2021). We recently identified 36 FA alleles which are collectively associated with a favourable metabolic profile, higher subcutaneous fat but lower ectopic liver fat (Ji et al., 2019; Martin et al., 2021), resembling a polygenic phenotype opposite to lipodystrophy (Semple et al., 2011). We also identified 38 unfavourable adiposity (UFA) alleles which are associated with higher fat in subcutaneous and visceral adipose tissue, and higher ectopic liver and pancreatic fat (Ji et al., 2019; Martin et al., 2021), resembling monogenic obesity (Supplementary file 1a). We performed MR studies and showed that FA and UFA have opposite causal effects on six metabolic conditions (Martin et al., 2021). While both FA and UFA were associated with higher adiposity, FA was causally associated with lower risk of type 2 diabetes, heart disease, hypertension, stroke, polycystic ovary syndrome, and non-alcoholic fatty liver disease. In contrast, as expected, UFA was associated with higher risk of these conditions. These results confirmed the ability of the two sets of adiposity variants to partially separate out the metabolic from the non-metabolic effects of higher adiposity.

In this study, we aimed to investigate the effects of separate components to higher adiposity on risk of additional metabolic diseases and many non-metabolic diseases. We used genetic variants associated with BMI, body fat percentage, FA, and UFA to understand the components of higher adiposity that are the predominant causes of disease risk. Our findings may give guidance on some obesity-related risks which are not dependent on metabolic consequences, thereby guiding appropriate medical care.

## Methods

### Study design

**Figure 1.**
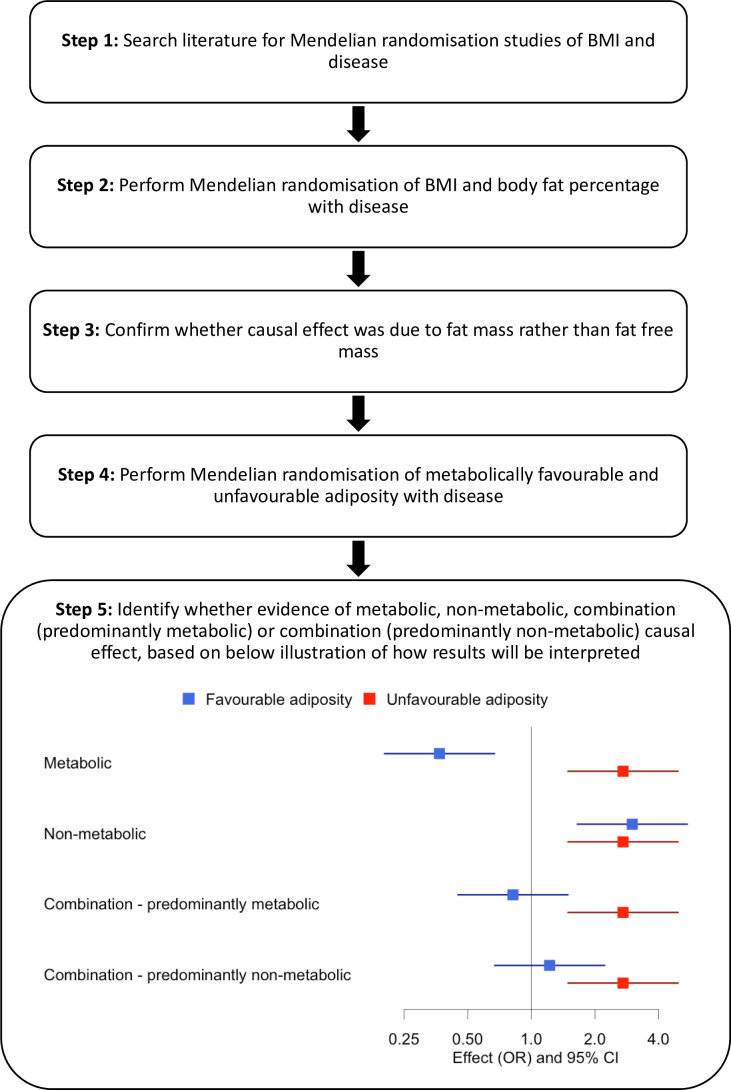
Study design.

An overview of our approach is shown in Figure 1. First, we identified diseases by performing a literature search of studies that had used MR to assess the consequences of BMI on outcome phenotypes. We used the search terms ‘BMI and Mendelian randomisation’ and ‘BMI and Mendelian randomization’. We identified 37 diseases associated with BMI and for which MR studies had previously been performed (Supplementary file 1b). We included all diseases regardless of the MR result in the published study. Second, we reperformed MR studies using BMI as an exposure. Third, for those diseases where MR indicated higher BMI was causal, we tested the effects of body fat percentage to confirm that the causal effect was due to fat mass rather than fat-free mass. Fourth, for diseases where MR suggested the BMI effect was due to excess adiposity, we used genetic variants more specific to the metabolic and non-metabolic components of higher adiposity to help understand the extent to which these factors influence disease.

### Data sources

We used three data sources for disease outcomes: (i) published genome-wide association studies (GWAS; Okada et al., 2014; Nikpay et al., 2015; Jones et al., 2017; Michailidou et al., 2017; Phelan et al., 2017; Scelo et al., 2017; Tsoi et al., 2017; Day et al., 2018; Mahajan et al., 2018; Malik et al., 2018; O’Mara et al., 2018; Roselli et al., 2018; Schumacher et al., 2018; Wray et al., 2018; An et al., 2019; Ferreira et al., 2019; Huyghe et al., 2019; Jansen et al., 2019; Kunkle et al., 2019; Law et al., 2019; Lindström et al., 2019; Morris et al., 2019; Nalls et al., 2019; Shah et al., 2019; Tachmazidou et al., 2019; Tin et al., 2019; Wuttke et al., 2019; Huyghe et al., 2021) and (ii) FinnGen (FinnGen, 2021) as our main results, and (iii) UK Biobank (RRID:SCR_012815; Collins, 2012) as additional validation. FinnGen is a cohort of 176,899 individuals with linked medical records. UK Biobank is a population cohort of >500,000 individuals aged 37–73 years recruited between 2006 and 2010 from across the UK. For the 37 identified diseases, 25 had summary GWAS data available from both a published GWAS consortium and FinnGen, and 12 diseases had GWAS summary data available in FinnGen only. In addition, data from 31 of the 37 diseases were available in the UK Biobank. No GWAS data were available for Barrett’s oesophagus, but we included gastro-oesophageal reflux. The characteristics of the studies and measures, disease outcomes, and the definition of cases and controls are described in Supplementary file 1ci–iii.

### GWAS of UK Biobank participants

For the GWAS of 31 diseases available in UK Biobank, we used a linear mixed model implemented in BOLT-LMM to account for population structure and relatedness (Loh et al., 2015). We used age, sex, genotyping platform, study centre, and the first five principal components as covariates in the model.

### Genetic variants

We used four sets of genetic variants as proxies of four exposures (Supplementary file 1d).

#### Body mass index

In the broadest category, we used a set of 73 variants independently associated with BMI at genome-wide significance (p<5 × 10^–8^). These variants were identified in the GIANT consortium of up to 339,224 individuals of European ancestry (Locke et al., 2015).

#### Body fat percentage

We used 696 variants from a GWAS in the UK Biobank (Martin et al., 2021). We used bio-impedance measures of body fat % taken by the Tanita BC-418MA body composition analyser in 442,278 individuals of European ancestry.

The BMI and body fat percentage variants were partially overlapping (n = 5 variants), but we used exposure-trait-specific weights for each variant.

#### FA variants

There are 36 FA variants (Martin et al., 2021). These variants were identified in two steps. First, they were associated (at p<5 × 10^–8^) with body fat percentage and a composite metabolic phenotype consisting of body fat percentage, HDL-cholesterol, triglycerides, SHBG, alanine transaminase, and aspartate transaminase. Second, in a k-means clustering approach (a hard clustering approach) (Martin et al., 2021), they formed a cluster of variants that were collectively associated with higher HDL-cholesterol, higher SHBG, and lower triglycerides and liver enzymes – resembling a phenotype opposite to lipodystrophy.

#### UFA variants

There are 38 UFA variants (Martin et al., 2021). These variants were identified in two steps. First, they were associated (at p<5 × 10^–8^) with body fat percentage and a composite metabolic phenotype as detailed above. Second, in a k-means clustering approach (Martin et al., 2021), they formed a cluster of variants that were collectively associated with lower HDL-cholesterol, lower SHBG, and higher triglycerides and liver enzymes - resembling monogenic obesity.

### Mendelian randomisation

We investigated the causal associations between the four exposures (BMI, body fat percentage, FA, and UFA) and 37 disease outcomes by performing two-sample MR analysis (Pierce and Burgess, 2013). We used the inverse-variance weighted (IVW) approach as our main analysis, and MR-Egger and weighted median as sensitivity analyses in order to detect and partially account for unidentified pleiotropy of our genetic instruments. For BMI, we used effect size estimates from the GWAS of BMI (Locke et al., 2015), and for body fat percentage, FA, and UFA, we used effect size estimates from the GWAS of body fat percentage (442,278 European ancestry individuals from the UK Biobank study) (Ji et al., 2019).

To estimate the effects of variants on our disease outcomes, we used two main sources of data: FinnGen GWAS summary results and published GWAS of the same diseases (Supplementary file 1ci–ii). We performed MR within each data source and then meta-analysed the results across the two datasets using a random-effects model with the R package *metafor* (RRID:SCR_003450; Viechtbauer, 2010), where the data was available in both. For one published GWAS (the GECCO consortium), we only had information for FA and UFA variants. To provide further MR evidence, we used a third source of disease data – disease status in the UK Biobank (Supplementary file 1ciii). We ran the same models but did not meta-analyse with published GWAS and FinnGen because most of the body fat percentage, FA, and UFA variants were identified in the UK Biobank.

We obtained heterogeneity Q statistics for each IVW MR and MR-Egger, and *I^2^* statistics for each MR-Egger analysis using the *MendelianRandomization* R package (Yavorska and Burgess, 2017). All statistical analyses were conducted using R software (R Development Core Team, 2020). Given the number of tests performed, we used a Benjamini–Hochberg false discovery rate (FDR) procedure and an FDR of 0.1 to define meaningful results for each of the four exposures (Benjamini and Hochberg, 1995).

## Results

We identified 37 diseases as associated with obesity and for which MR studies had previously been performed. Of these 37, 5 metabolic conditions were part of our previous study that validated the use of FA and UFA genetic variants as a way of partially separating the metabolic from non-metabolic components of higher adiposity (Martin et al., 2021). Once we had tested BMI and body fat percentage, we further characterised the likely causal component of higher adiposity using FA and UFA variants as follows (Figure 1, step 5): (i) diseases with evidence that the metabolic effect of higher adiposity is causal. Here, MR using the UFA genetic variants indicated that higher adiposity with its adverse metabolic consequences was causal to disease, whilst MR using the FA genetic variants indicated that higher adiposity with favourable metabolic effects was protective (at FDR 0.1). (ii) Diseases with evidence that there is a non-metabolic causal effect (e.g. mechanical effect, psychological/adverse social effect). Here, MR using the FA genetic variants indicated that higher adiposity without its adverse metabolic consequences was likely contributing to the disease, as well as the MR using the UFA genetic variants. (iii) Diseases with evidence that there is a combination of causal effects but with a predominantly metabolic component. Here, MR using the UFA genetic variants indicated that higher adiposity with its adverse metabolic consequences was causal to disease, and MR using the FA genetic variants was directionally consistent with higher adiposity with favourable metabolic effects being protective but FDR > 0.1. (iv) Diseases with evidence that there is a combination of causal effects but with a predominantly non-metabolic component. Here, MR using the UFA genetic variants indicated that higher adiposity without its adverse metabolic consequences was likely contributing to the disease, and MR of the FA genetic variants was directionally consistent with this but FDR > 0.1.

We grouped these disease outcomes into seven major categories – cardiovascular and metabolic conditions, musculoskeletal, gastrointestinal, nervous, integumentary and respiratory systems, and cancer. MR analysis of five conditions (coronary artery disease, hypertension, stroke, type 2 diabetes, and polycystic ovary syndrome) was part of our previous study (Martin et al., 2021). We focused on the MR of body fat percentage if a causal effect of BMI was indicated, and the MR of FA and UFA if a causal effect of BMI and body fat percentage was indicated, but have presented all results in Supplementary file 1e for completeness. Where random-effects meta-analyses were performed, the heterogeneity statistics are given in Supplementary file 1f.

### (i) Diseases with evidence that the metabolic effect of higher adiposity is causal

**Figure 2.**
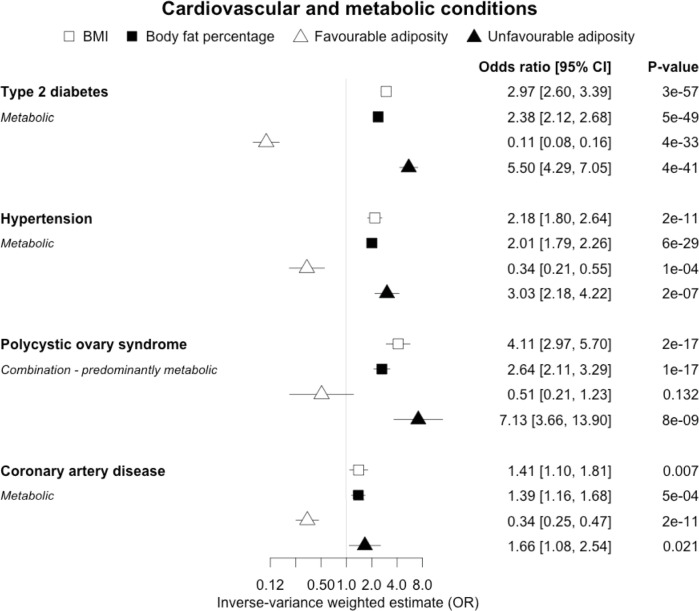
The inverse-variance weighted (IVW) two-sample MR analysis/meta-analysis of the effects of body mass index (BMI), body fat percentage (BFP), “favourable adiposity” (FA) and “unfavourable adiposity” (UFA) on type 2 diabetes, hypertension, polycystic ovary syndrome and coronary artery disease. The error bars represent the 95% confidence intervals of the IVW estimates in odds ratio per standard deviation change in genetically determined BMI, body fat percentage, FA and UFA. *Italics give our best interpretation of the data using the FDR 0.1 results.*

**Figure 3.**
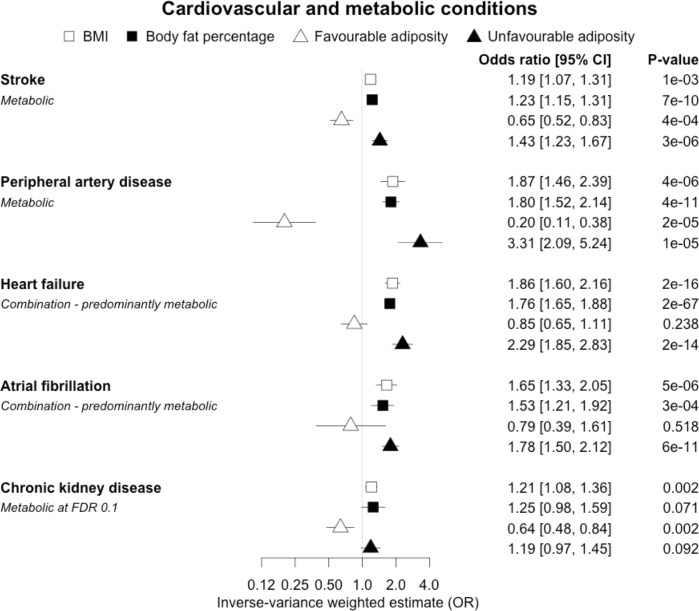
The inverse-variance weighted (IVW) two-sample MR analysis/meta-analysis of the effects of body mass index (BMI), body fat percentage (BFP), “favourable adiposity” (FA) and “unfavourable adiposity” (UFA) on stroke, peripheral artery disease, heart failure, atrial fibrillation and chronic kidney disease. The error bars represent the 95% confidence intervals of the IVW estimates in odds ratio per standard deviation change in genetically determined BMI, body fat percentage, FA and UFA. *Italics give our best interpretation of the data using the FDR 0.1 results.*

**Figure 4.**
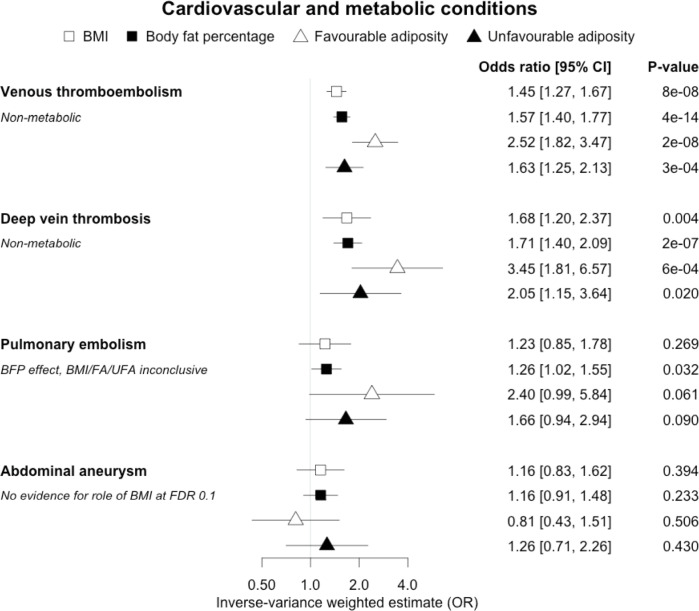
The inverse-variance weighted (IVW) two-sample MR analysis/meta-analysis of the effects of body mass index (BMI), body fat percentage (BFP), “favourable adiposity” (FA) and “unfavourable adiposity” (UFA) on venous thromboembolism, deep vein thrombosis, pulmonary embolism and abdominal aneurysm. The error bars represent the 95% confidence intervals of the IVW estimates in odds ratio per standard deviation change in genetically determined BMI, body fat percentage, FA and UFA. *Italics give our best interpretation of the data using the FDR 0.1 results.*

**Figure 5.**
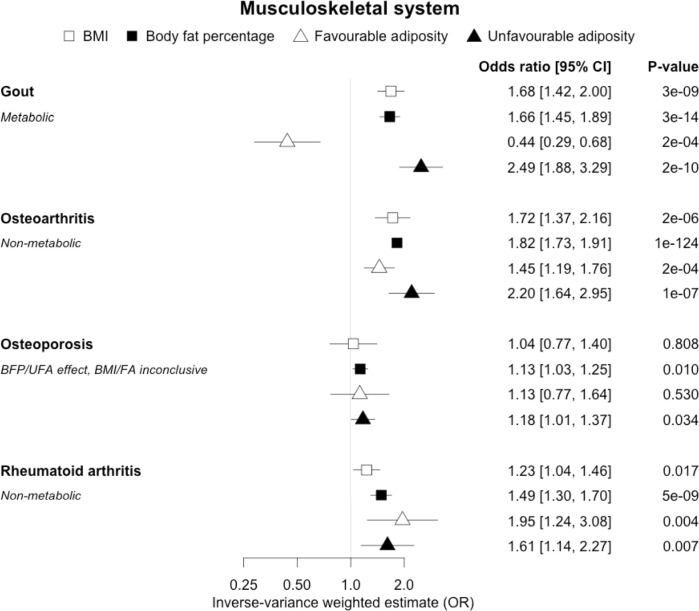
The inverse-variance weighted (IVW) two-sample MR analysis/meta-analysis of the effects of body mass index (BMI), body fat percentage (BFP), “favourable adiposity” (FA) and “unfavourable adiposity” (UFA) on gout, osteoarthritis, osteoporosis and rheumatoid arthritis. The error bars represent the 95% confidence intervals of the IVW estimates in odds ratio per standard deviation change in genetically determined BMI, body fat percentage, FA and UFA. *Italics give our best interpretation of the data using the FDR 0.1 results.*

**Figure 6.**
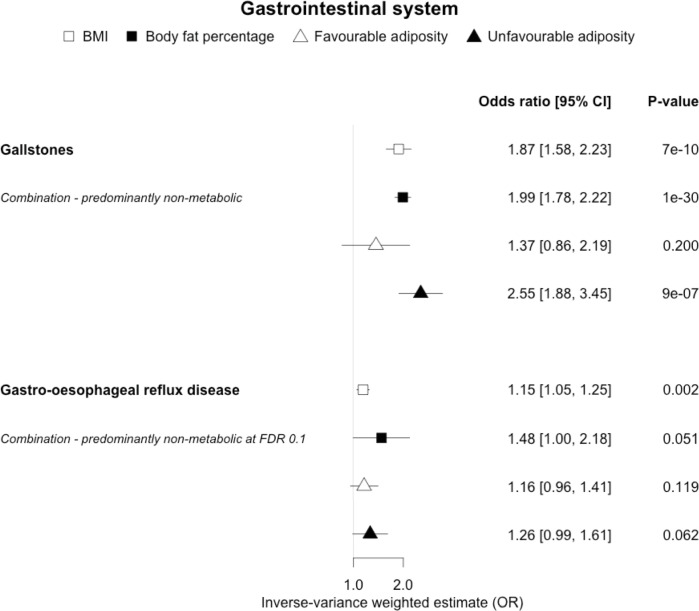
The inverse-variance weighted (IVW) two-sample MR analysis/meta-analysis of the effects of body mass index (BMI), body fat percentage (BFP), “favourable adiposity” (FA) and “unfavourable adiposity” (UFA) on gallstones and gastro-oesophageal reflux disease. The error bars represent the 95% confidence intervals of the IVW estimates in odds ratio per standard deviation change in genetically determined BMI, body fat percentage, FA and UFA. *Italics give our best interpretation of the data using the FDR 0.1 results.*

**Figure 7.**
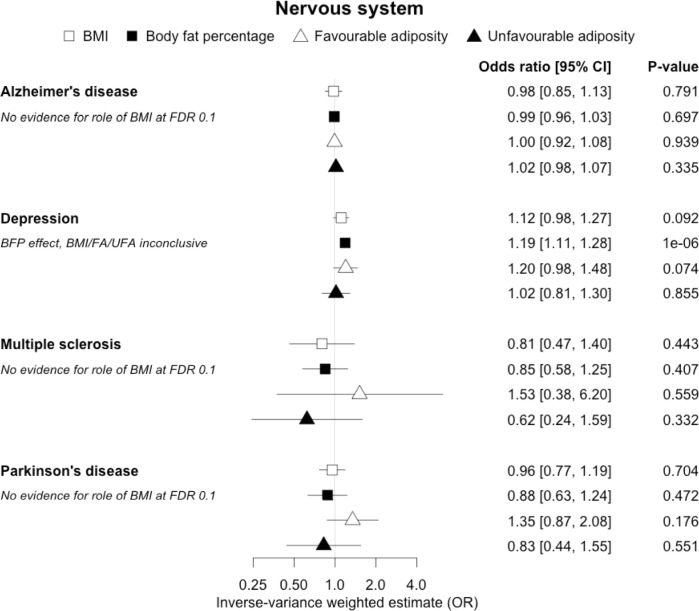
The inverse-variance weighted (IVW) two-sample MR analysis/meta-analysis of the effects of body mass index (BMI), body fat percentage (BFP), “favourable adiposity” (FA) and “unfavourable adiposity” (UFA) on Alzheimer’s disease, depression, multiple sclerosis and Parkinson’s disease. The error bars represent the 95% confidence intervals of the IVW estimates in odds ratio per standard deviation change in genetically determined BMI, body fat percentage, FA and UFA. *Italics give our best interpretation of the data using the FDR 0.1 results.*

**Figure 8.**
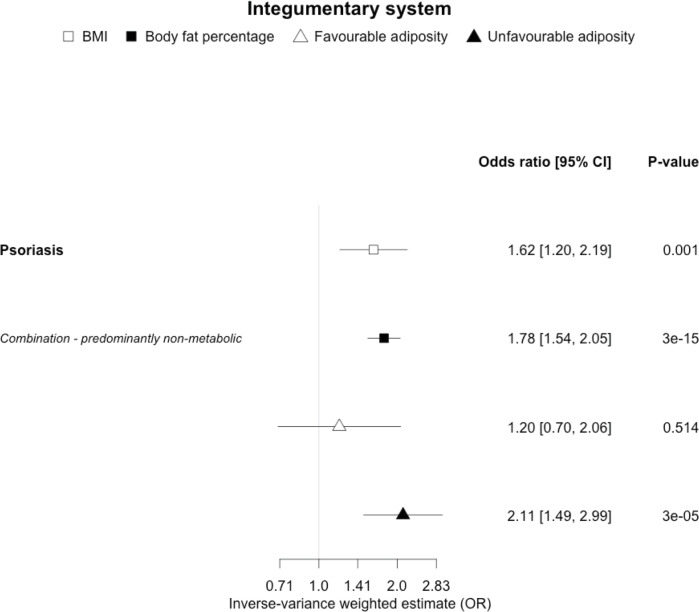
The inverse-variance weighted (IVW) two-sample MR analysis/meta-analysis of the effects of body mass index (BMI), body fat percentage (BFP), “favourable adiposity” (FA) and “unfavourable adiposity” (UFA) on psoriasis. The error bars represent the 95% confidence intervals of the IVW estimates in odds ratio per standard deviation change in genetically determined BMI, body fat percentage, FA and UFA. *Italics give our best interpretation of the data using the FDR 0.1 results.*

**Figure 9.**
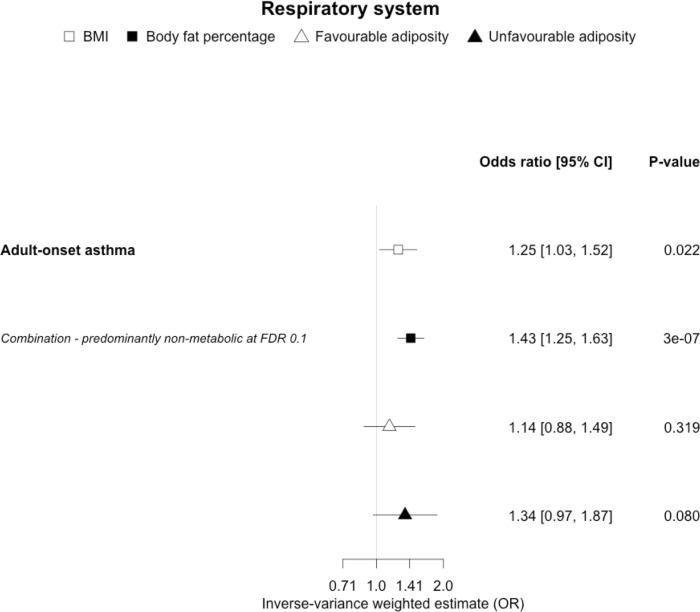
The inverse-variance weighted (IVW) two-sample MR analysis/meta-analysis of the effects of body mass index (BMI), body fat percentage (BFP), “favourable adiposity” (FA) and “unfavourable adiposity” (UFA) on adult-onset asthma. The error bars represent the 95% confidence intervals of the IVW estimates in odds ratio per standard deviation change in genetically determined BMI, body fat percentage, FA and UFA. *Italics give our best interpretation of the data using the FDR 0.1 results.*

**Figure 10.**
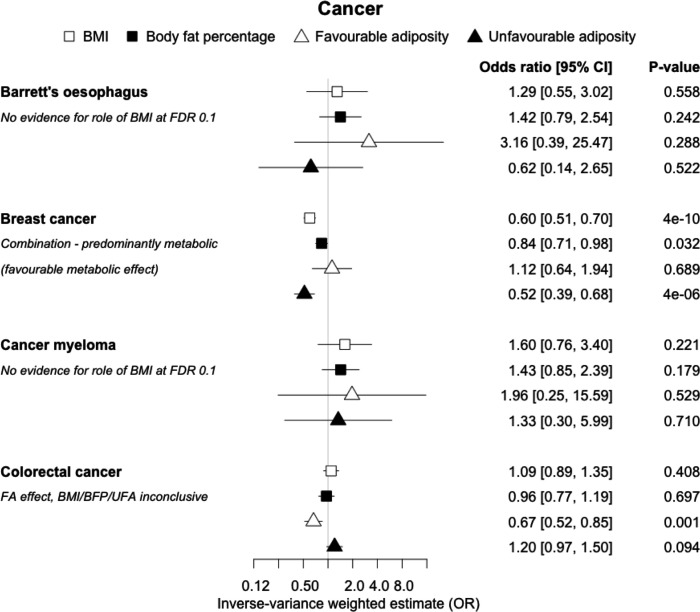
The inverse-variance weighted (IVW) two-sample MR analysis/meta-analysis of the effects of body mass index (BMI), body fat percentage (BFP), “favourable adiposity” (FA) and “unfavourable adiposity” (UFA) on Barrett’s oesophagus, breast cancer, cancer myeloma and colorectal cancer. The error bars represent the 95% confidence intervals of the IVW estimates in odds ratio per standard deviation change in genetically determined BMI, body fat percentage, FA and UFA. *Italics give our best interpretation of the data using the FDR 0.1 results.*

**Figure 11.**
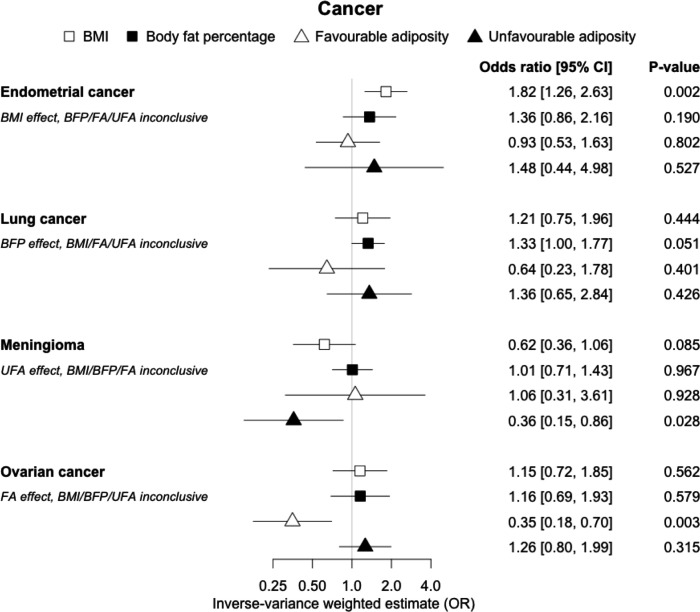
The inverse-variance weighted (IVW) two-sample MR analysis/meta-analysis of the effects of body mass index (BMI), body fat percentage (BFP), “favourable adiposity” (FA) and “unfavourable adiposity” (UFA) on endometrial and lung cancer, meningioma and ovarian cancer. The error bars represent the 95% confidence intervals of the IVW estimates in odds ratio per standard deviation change in genetically determined BMI, body fat percentage, FA and UFA. *Italics give our best interpretation of the data using the FDR 0.1 results.*

**Figure 12.**
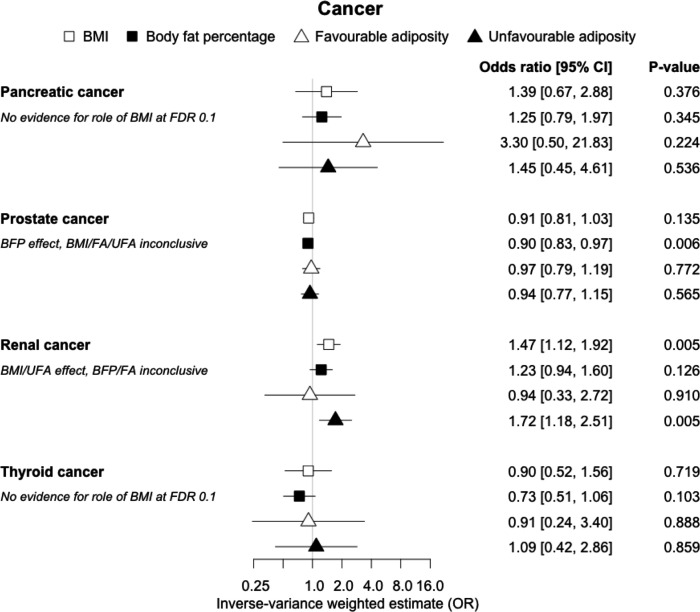
The inverse-variance weighted (IVW) two-sample MR analysis/meta-analysis of the effects of body mass index (BMI), body fat percentage (BFP), “favourable adiposity” (FA) and “unfavourable adiposity” (UFA) on pancreatic, prostate, renal and thyroid cancer. The error bars represent the 95% confidence intervals of the IVW estimates in odds ratio per standard deviation change in genetically determined BMI, body fat percentage, FA and UFA. *Italics give our best interpretation of the data using the FDR 0.1 results.*

When comparing the MR analyses for FA and UFA, our results provided evidence that the metabolic effect of higher adiposity is contributing causally to coronary artery disease, peripheral artery disease, hypertension, stroke, type 2 diabetes, and gout (Figures 2—12, Supplementary file 1e). For stroke, our results were consistent when using sub-types of the condition (Figure 3—figure supplement 1, Supplementary file 1g). Our results also indicated that the metabolic effect of higher adiposity is causal to chronic kidney disease, although the results from BMI and body fat percentage were less conclusive (Figure 3).

**Figure 3—figure supplement 1.**
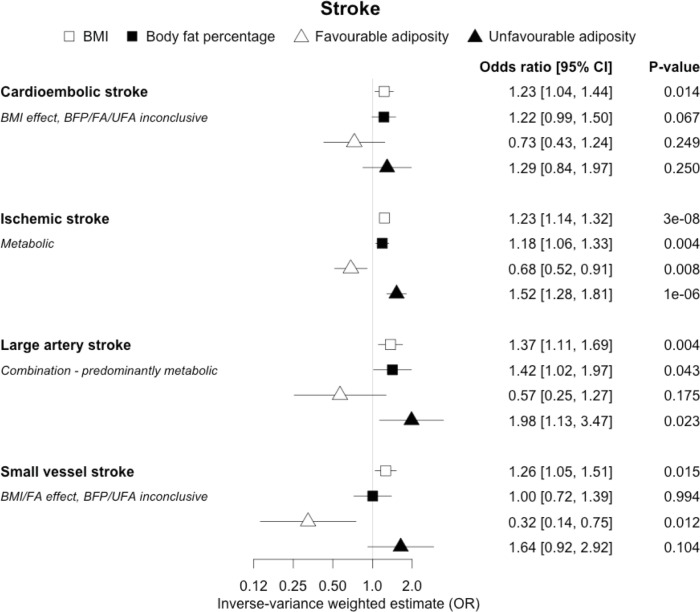
The inverse-variance weighted (IVW) two-sample MR analysis/meta-analysis of the effects of body mass index (BMI), body fat percentage (BFP), “favourable adiposity” (FA) and “unfavourable adiposity” (UFA) on sub-types of stroke. The error bars represent the 95% confidence intervals of the IVW estimates in odds ratio per standard deviation change in genetically determined BMI, body fat percentage, FA and UFA. *Italics give our best interpretation of the data using the confidence intervals.*

**Figure 5—figure supplement 1.**
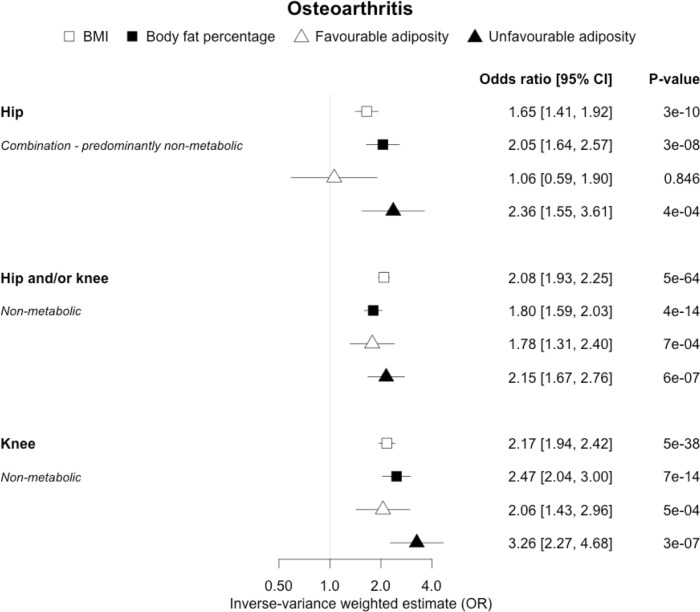
The inverse-variance weighted (IVW) two-sample MR analysis/meta-analysis of the effects of body mass index (BMI), body fat percentage (BFP), “favourable adiposity” (FA) and “unfavourable adiposity” (UFA) on sub-types of osteoarthritis. The error bars represent the 95% confidence intervals of the IVW estimates in odds ratio per standard deviation change in genetically determined BMI, body fat percentage, FA and UFA. *Italics give our best interpretation of the data using the confidence intervals.*

**Figure 9—figure supplement 1.**
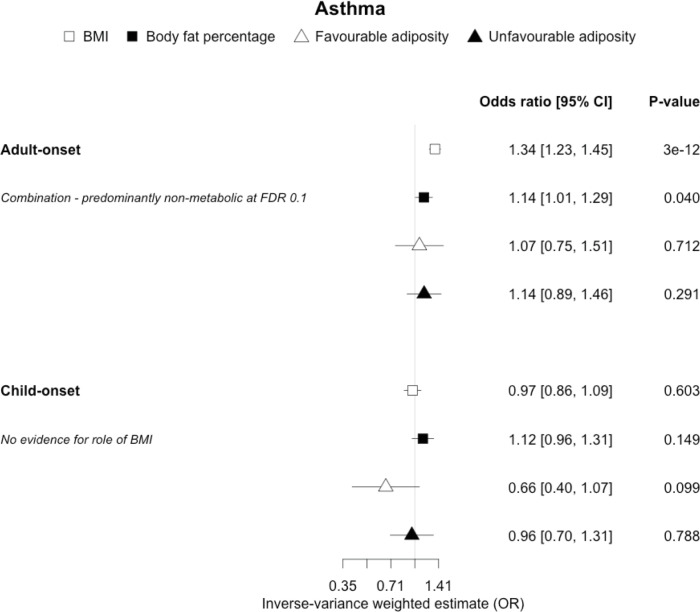
The inverse-variance weighted (IVW) two-sample MR analysis/meta-analysis of the effects of body mass index (BMI), body fat percentage (BFP), “favourable adiposity” (FA) and “unfavourable adiposity” (UFA) on sub-types of asthma. The error bars represent the 95% confidence intervals of the IVW estimates in odds ratio per standard deviation change in genetically determined BMI, body fat percentage, FA and UFA. *Italics give our best interpretation of the data using the confidence intervals (†or FDR 0.1 results).*

**Figure 10—figure supplement 1.**
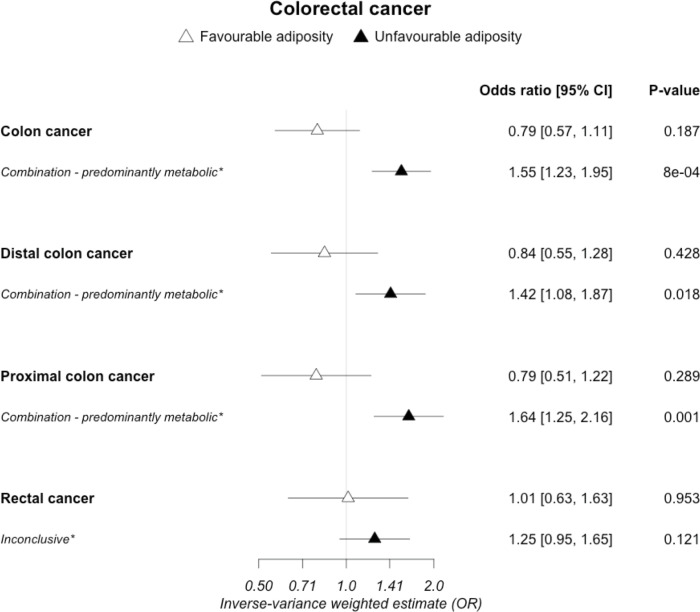
The inverse-variance weighted (IVW) two-sample MR analysis/meta-analysis of the effects of body mass index (BMI), body fat percentage (BFP), “favourable adiposity” (FA) and “unfavourable adiposity” (UFA) on sub-types of colorectal cancer. The error bars represent the 95% confidence intervals of the IVW estimates in odds ratio per standard deviation change in genetically determined BMI, body fat percentage, FA and UFA. *Interpretations are limited to FA and UFA because SNPs were not available from the full BMI and body fat percentage list for this dataset. *Italics give our best interpretation of the data using the confidence intervals.*

**Figure 11—figure supplement 1.**
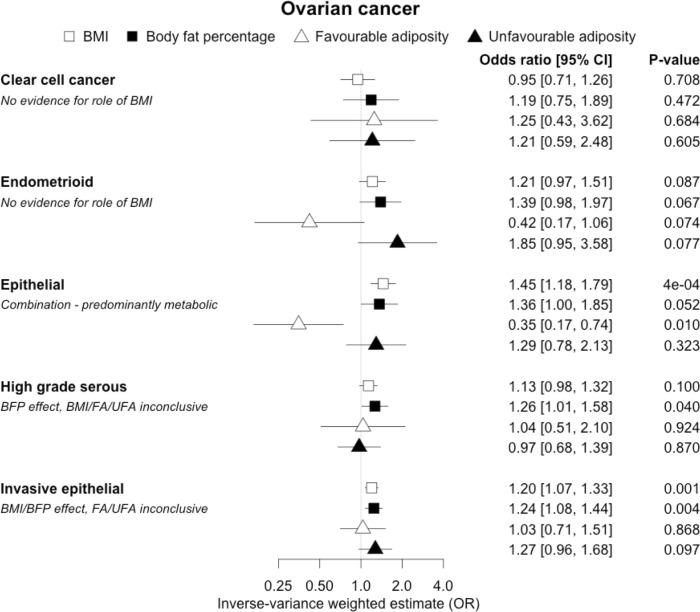
The inverse-variance weighted (IVW) two-sample MR analysis/meta-analysis of the effects of body mass index (BMI), body fat percentage (BFP), “favourable adiposity” (FA) and “unfavourable adiposity” (UFA) on 5 sub-types of ovarian cancer. The error bars represent the 95% confidence intervals of the IVW estimates in odds ratio per standard deviation change in genetically determined BMI, body fat percentage, FA and UFA. Italics give our best interpretation of the data using the confidence intervals.

**Figure 11—figure supplement 2.**
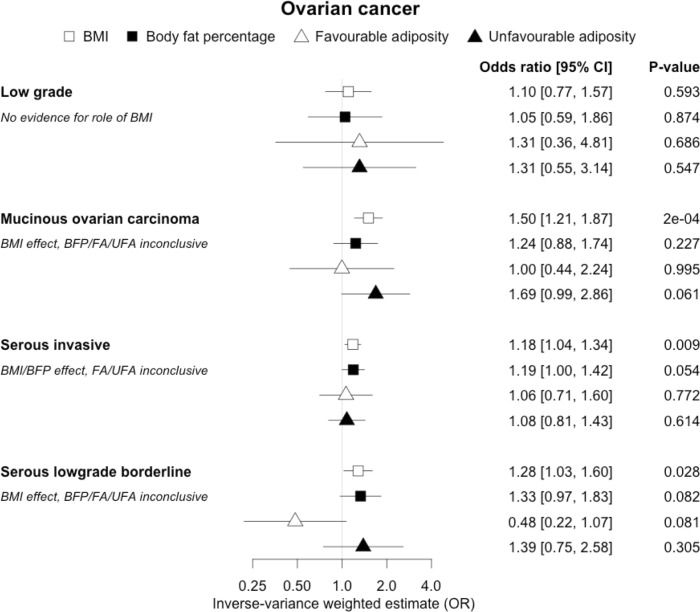
The inverse-variance weighted (IVW) two-sample MR analysis/meta-analysis of the effects of body mass index (BMI), body fat percentage (BFP), “favourable adiposity” (FA) and “unfavourable adiposity” (UFA) on 4 sub-types of ovarian cancer. The error bars represent the 95% confidence intervals of the IVW estimates in odds ratio per standard deviation change in genetically determined BMI, body fat percentage, FA and UFA. *Italics give our best interpretation of the data using the confidence intervals.*

### (ii) Diseases with evidence that there is a non-metabolic causal effect

When comparing the MR analyses for FA and UFA, our results provided evidence that some non-metabolic effect of higher adiposity is contributing causally to venous thromboembolism, deep vein thrombosis, osteoarthritis, and rheumatoid arthritis (Figures 2—12, Supplementary file 1e). For osteoarthritis, our results were consistent when using sub-types of the condition (Figure 5—figure supplement 1, Supplementary file 1g).

### (iii) Diseases with evidence that there is a combination of causal effects but with a predominantly metabolic component

When comparing the MR analyses for FA and UFA, our results provided evidence that the metabolic effect of higher adiposity is the predominate cause of the link between higher BMI and polycystic ovary syndrome, heart failure, and atrial fibrillation. Our results also provided evidence that the metabolic effect of higher adiposity is the predominate cause of the link between higher BMI and a reduced risk of breast cancer and higher risk of renal cancer, although the results from body fat percentage were less conclusive (Figures 2—12, Supplementary file 1e).

### (iv) Diseases with evidence that there is a combination of causal effects but with a predominantly non-metabolic component

When comparing the MR analyses for FA and UFA, our results suggested that some non-metabolic effect of higher adiposity is the predominant cause of the link between higher BMI and gallstones, gastro-oesophageal reflux disease, adult-onset asthma, and psoriasis (Figures 2—12, Supplementary file 1e). Our results also indicated that some non-metabolic effect of higher adiposity is causal to osteoporosis, although the results from BMI were less conclusive (Figure 5). Our results found no evidence (at p<0.05) of an effect of BMI or adiposity on child-onset asthma (Figure 9—figure supplement 1, Supplementary file 1g).

### All other disease outcomes

Fifteen disease outcomes did not fit the criteria for definitions i–iv. For five of these conditions, our MR results indicated a causal effect of higher BMI or adiposity, but results from FA and UFA were inconclusive: pulmonary embolism, depression, endometrial cancer, lung cancer, and prostate cancer (Figures 2—12, Supplementary file 1e). Additionally, we identified some evidence of a metabolic effect of higher adiposity with colorectal and ovarian cancer, with the MR of FA indicating lower odds of colorectal (0.67 [0.52, 0.85]) and ovarian (0.35 [0.18, 0.70]) cancers, but MR of UFA was consistent with the null (p>0.05). For colorectal and ovarian cancer, our results were consistent when using sub-types of the conditions (Figure 10—figure supplement 1, Figure 11—figure supplements 1 and 2, Supplementary file 1g).

### Sensitivity analyses

Out of 82 disease outcomes (including subtypes), weighted median MR results were directionally consistent with IVW analysis for 75 diseases for BMI and 73 for body fat percentage, with 33 and 47 of these having p<0.05, respectively. For FA and UFA, where sub-type colorectal cancer data was available, the total number of diseases was 87, and 76 were directionally consistent for both exposures, with 22 and 39 having p<0.05, respectively.

MR-Egger results were broadly consistent with the primary IVW MR results, indicating that pleiotropy (variants acting on the outcomes through more than one mechanism) appears to have had limited effect on our results. MR-Egger results were directionally consistent with IVW for 71 diseases for BMI and 70 for body fat percentage, with 25 and 38 of these having p<0.05, respectively. For FA and UFA, MR-Egger was directionally consistent for 60 and 67 diseases, with 6 and 15 having p<0.05, respectively (Supplementary file 1g). Of the 31 diseases available in the UK Biobank, the IVW analysis of these was directionally consistent with the FinnGen and/or published GWAS analysis for 28, 27, 24, and 27 traits for BMI, body fat percentage, FA, and UFA, respectively (Supplementary file 1h). Of these, 18, 21, 9, and 16 had p<0.05, respectively.

## Discussion

We used a genetic approach to understand the role of higher adiposity uncoupled from its adverse metabolic effects in mechanisms linking obesity to higher risk of disease. We first used MR to provide evidence that higher BMI was causally associated with 21 diseases, broadly consistent with those from previous studies. For the majority (17) of these diseases, our results indicated that the BMI effect was predominantly due to excess adiposity rather than a non-fat mass component to BMI. We then used a more specific approach to test the separate roles of higher adiposity with and without its adverse metabolic effects. We provided genetic evidence that the adverse metabolic consequences of higher BMI lead to coronary artery disease, peripheral artery disease, hypertension, stroke, type 2 diabetes, polycystic ovary syndrome, heart failure, atrial fibrillation, chronic kidney disease, renal cancer, and gout, and the adverse non-metabolic consequences of higher BMI likely contribute to osteoarthritis, rheumatoid arthritis, osteoporosis, gastro-oesophageal reflux disease, gallstones, adult-onset asthma, psoriasis, deep vein thrombosis, and venous thromboembolism.

Understanding the reasons why obesity leads to disease is important in order to better advise health professionals and patients of health risks linked to obesity, whether or not they show metabolic derangements. Many previous studies have used an MR approach to support a causal role of higher BMI in disease, but here we attempted to systematically test many conditions and the role of separate components of higher BMI. We discuss some of the more notable, and potentially clinically important, results below.

### Cardiometabolic diseases

Previous studies, including those using MR, have shown that higher BMI leads to many cardiometabolic diseases (Larsson et al., 2020; Riaz et al., 2018; Xu et al., 2020), but our results provide additional insight into the likely mechanisms. In addition to the previously established opposing effects of metabolically FA and UFA for coronary artery disease, stroke, hypertension, and type 2 diabetes (Martin et al., 2021), our results confirmed similarly strong metabolic components to peripheral artery disease and chronic kidney disease. These results are consistent with the well-established adverse metabolic effects of higher BMI on these diseases (contributing to atherosclerotic effects or linked to specific haemodynamic impacts) (Sattar and McGuire, 2018). For two further cardiovascular conditions, heart failure and atrial fibrillation, the results were less certain. For these two conditions, the evidence of a predominantly metabolic effect of higher BMI was very clear – with the MR of UFA consistent with effects at least as strong as those for coronary artery disease. However, in contrast to the results for coronary artery disease, the MR of FA was consistent with no effect. This comparison between the effects of FA and UFA may indicate that there is a partial mechanical, or other non-metabolic component, as well as metabolic effect, perhaps mediated by excess weight of any type placing extra strain on the heart.

In contrast to the results for most of the cardiometabolic diseases, our MR analyses provided evidence for a likely non-metabolic component mediating the effect of higher BMI on venous thromboembolism and deep vein thrombosis (two closely related conditions). This finding is clinically important as it suggests that treating metabolic risk factors associated with obesity without changing weight may not reduce the risk of deep vein thrombosis in individuals with obesity. Possible mechanisms could include higher intra‐abdominal pressure (due to excess fat) and slower blood circulation in the lower limbs (due to a more sedentary lifestyle secondary to obesity, or mechanical occlusion of veins) promoting clot initiation and formation (Lorenzet et al., 2012).

### Musculoskeletal diseases

We observed clear differences for the role of higher BMI in different musculoskeletal diseases. For gout, opposing effects of FA and UFA clearly indicated a metabolic effect. Gout is a form of inflammatory arthritis caused by the deposition of urate crystals within the joints (Dalbeth et al., 2016). Weight loss from bariatric surgery is associated with lower serum uric acid and lower risk of gout (Maglio et al., 2017). A previous MR study showed that overall obesity, but not the central location of fat, increased the risk of gout (Larsson et al., 2018). The protective effect of FA could be due to improved insulin sensitivity leading to less insulin-enhanced reabsorption of organic anions such as urate (Choi et al., 2005). In contrast to gout, our MR analysis provided evidence that a non-metabolic effect of higher adiposity is a likely cause of osteoarthritis and rheumatoid arthritis – with both FA and UFA leading to disease. For osteoarthritis, the effect of UFA was stronger than that of FA, indicating both a metabolic and non-metabolic component. This is consistent with a causal association between higher adiposity and higher risk of osteoarthritis in non-weight-bearing joints including hands (Reyes et al., 2016). For rheumatoid arthritis, the effects of FA and UFA were similar, suggesting the non-metabolic effect accentuating, or more readily unmasking, the autoimmune background risk, as the key BMI-related factor, although the confidence intervals were wider than those for osteoarthritis. The UFA variants may potentially influence these conditions by load-bearing mechanisms, and tissue enrichment analysis for the FA and UFA variants previously found that FA and UFA loci are enriched for genes expressed in adipocytes and adipose tissue, and mesenchymal stem cells, respectively (Martin et al., 2021). For osteoporosis, we did not replicate the previous finding of a causal association between higher BMI and risk of osteoporosis (estimated by bone mineral density; Song et al., 2020); however, we observed a causal association between higher body fat percentage and a higher risk of osteoporosis with consistent risk increasing effects of both FA and UFA. This finding adds to the complex relationship between higher BMI and osteoporosis, where higher BMI at earlier ages may increase bone accrual, but in later years results in adverse effects.

### Gastrointestinal diseases

We observed differences in the effects of BMI when comparing the two gastrointestinal diseases, although the results are less conclusive than those for the musculoskeletal conditions. Here, our results were consistent with a predominantly non-metabolic effect contributing to the association between higher BMI and higher risk of gallstones. Higher BMI has been shown to be causally associated with higher risk of gallstones (Yuan et al., 2021). There are several possible mechanisms that could explain how higher BMI without its adverse metabolic effects could increase the risk of gallstones. These could include a sedentary lifestyle and gallbladder hypomotility secondary to increased abdominal fat mass (Mathus-Vliegen et al., 2004). Metabolic mechanisms could include hepatic de novo cholesterol synthesis (Ståhlberg et al., 1997; Cruz-Monserrate et al., 2016). For gastro-oesophageal reflux, the consistent direction and effect sizes of higher FA and UFA indicate a non-metabolic component, an effect that may be mechanical and better explained by higher central adiposity rather than overall BMI (Green et al., 2020).

### Other diseases

For most of the other diseases tested, it was difficult to draw firm conclusions about the role of metabolically FA and UFA. For some diseases, this was in part due to the lack of MR evidence for a role of any form of higher BMI. For example, our MR analyses provided no evidence for the role of higher BMI in the neurodegenerative diseases Alzheimer’s disease, multiple sclerosis, and Parkinson’s. These results are consistent with some but not all previous studies. For example, higher BMI is listed as a key risk factor for Alzheimer’s disease (Livingston et al., 2020), although with little evidence of causality, including MR studies that failed to show an effect (Larsson et al., 2017; Nordestgaard et al., 2017). In contrast to our results, recent MR studies have indicated that higher BMI is protective of Parkinson’s disease (Noyce et al., 2017) and causally associated with higher risk of multiple sclerosis (Mokry et al., 2016). For the inflammatory skin disorder psoriasis, our results indicated that both higher BMI and higher body fat percentage are causally associated with higher risk, but determining the underlying mechanism from the MR of FA and UFA was difficult. Higher BMI is a known cause of psoriasis (Budu-Aggrey et al., 2019; Iskandar et al., 2015) and weight loss is a recommended treatment (Iskandar et al., 2015). It is possible that both metabolic and non-metabolic pathways are driving the risk. The non-metabolic pathways could include inflammation which is one of the possible causal mechanisms (Sbidian et al., 2017; Dowlatshahi et al., 2013). Further work is required to understand if psoriasis could be effectively treated by targeting the metabolic factors alone, or whether only weight loss will benefit such patients. For cancers, our results do not provide any clear additional insight into the likely mechanisms, with potentially stronger effects for BMI and UFA compared to body fat percentage in some analyses hard to explain biologically. The reasons why higher BMI is associated with cancers is uncertain, although several MR studies indicate that the association with many is causal (Mariosa et al., 2019; Vincent and Yaghootkar, 2020), and that central adiposity may play a role (Jarvis et al., 2016). Exposure to higher insulin levels is a plausible mechanism, and some studies have used MR to test insulin directly (Nead et al., 2015; Shu et al., 2019; Carreras-Torres et al., 2017b; Carreras-Torres et al., 2017a; Johansson et al., 2019). Our MR analysis reproduced the previous finding between higher adiposity and higher risk of endometrial cancer (Painter et al., 2016) and renal cell carcinoma (Johansson et al., 2019), and lower risk of breast cancer (Guo et al., 2016; Shu et al., 2019). In contrast to previous MR studies showing a causal link between higher BMI and higher risk of prostate cancer (Kazmi et al., 2020; Davies et al., 2015), we identified a causal association between higher body fat percentage but lower risk of prostate cancer. The relationship between higher BMI and risk of breast cancer is complicated, with MR studies indicating that higher BMI is protective of postmenopausal breast cancer (Gao et al., 2016). This contrasts with the epidemiological associations but could be explained by effects of childhood BMI (Richardson et al., 2020).

### Strengths and limitations

Our study had a number of limitations. First, we do not know all of the potential effects of the FA and UFA genetic variants on intermediary mechanisms. For example, the inflammatory profile of the FA variants needs further characterisation. However, the consistent association of the FA genetic variants with lower risk of a wide range of metabolic conditions – from type 2 diabetes where insulin resistance predominates, to stroke where atherosclerotic and blood pressure mechanisms predominate – indicates that these variants collectively represent a profile of higher adiposity and favourable metabolic factors. Second, for some diseases, we may have not had sufficient power to detect an effect of BMI or to separate the effects, and this could explain some of the null findings, especially for conditions where we might have expected an effect, such as pulmonary embolism and aortic aneurysm, but there were smaller numbers of cases available. Third, in some situations it was harder to interpret the results from the MR FA and UFA analyses, especially when one appeared to show an effect and the other did not. One possibility is that some diseases are a combination of both non-metabolic and metabolic effects. Osteoarthritis was the best example of this potential scenario because both FA and UFA increased the risk of disease, but UFA to a greater extent. However, for other diseases, it could be hard to detect a combined effect because the MR with FA could be protective (if metabolic effects predominate), increase risk (if non-metabolic effects predominate), or null (if the two have similar effects). Finally, we used an FDR of 0.1 as a guide to discussing meaningful results. We observed 21 out of the 37 outcome diseases reaching an FDR of 0.1 (based on the Benjamini–Hochberg procedure) for BMI, and 19, 11, and 20 out of the 21 diseases causally associated with BMI reaching this FDR for body fat percentage, FA, and UFA, respectively. Equivalent numbers for an FDR of 0.05 were 21, 17, 11, and 17. Excluding the five metabolic conditions used in our previous study (which were all causally associated with BMI), these results are 16, 14, 7, and 15 for an FDR of 0.1, and 16, 12, 7, and 12 for an FDR of 0.05. In addition to correcting for multiple tests, we noted that 74 of the 37 × 4 MR tests reached a p-value of <0.05 when we would only expect 7 by chance, suggesting many of the tests that did not reach a strict Bonferroni p<0.05 were meaningful.

In summary, we have used a genetic approach to test the separate roles of higher adiposity with and without its adverse metabolic effects. These results emphasize that many people in the community who are of higher BMI are at risk of multiple chronic conditions that can severely impair their quality of life or cause morbidity or mortality, even if their metabolic parameters appear relatively normal.

## Data Availability

GWAS data from the outcome diseases studied is available from links published in the original studies (Supplementary File 1ci). FinnGen data is available at: https://finngen.gitbook.io/documentation/, and the list of disease outcomes used is in Supplementary File 1cii. Individual-level UK Biobank data cannot be provided, but it is available by application to the UK Biobank: https://www.ukbiobank.ac.uk, and a list of the traits used is in Supplementary File 1ciii. Code used to conduct this analysis will be made available on GitHub after removing any sensitive information (https://github.com/susiemartin/uncoupling-bmi, copy archived at swh:1:rev:f3472762ad6cb7f313656f684e07c14b8735efe5).

## Additional files

Supplementary file 1. Supplementary methods and Mendelian randomisation (MR) results file. (**a**) Characterisation of monogenic obesity, lipodystrophy, unfavourable adiposity (UFA), and favourable adiposity (FA) using body fat percentage and a selection of metabolic biomarkers. (**b**) Mendelian randomisation (MR) studies testing the role of obesity (usually as body mass index [BMI]) identified in literature search. (**c**) (i) Summary statistics of published genome-wide association studies (GWAS) used. Mean (standard deviation [SD] or range) are given for continuous study characteristics where available, mean ranges are given for meta-analyses unless otherwise specified. *Statistics represent only UK Biobank cohort of those included in meta-analysis. (**c**) (ii) Summary statistics of FinnGen studies used. Mean age of cases is given where available, BMI is not adjusted for, and UK Biobank is not included in these studies. ICD codes taken from hospital discharge register and/or causes of death register. (**c**) (iii) Summary statistics of UK Biobank studies used. Mean (SD) are given for continuous study characteristics of cases. Self-report code is from n_20002_* variable in UK Biobank. (**d**) The summary of 73 BMI and 696 body fat percentage genetic variants, the latter including 36 FA and 38 UFA genetic variants. Beta, SE, and p are from the GWAS of BMI and body fat percentage in UK Biobank, respectively. BMI variants were discovered using non-UK Biobank cohorts, and so some SNPs listed may have zero effect size in the UK Biobank GWAS of BMI. (e) The inverse-variance weighted two-sample MR analysis/meta-analysis of 37 identified diseases from published GWAS and/or FinnGen for BMI, body fat percentage, FA, and UFA clusters. *Italicised results are those that were interpreted – including all BMI, body fat percentage if a causal effect of BMI was indicated, and FA/UFA if a causal effect of BMI and body fat percentage was indicated*. (**f**) Heterogeneity statistics from random-effects meta-analysis of inverse-variance weighted MR of published GWAS and FinnGen studies. (**g**) (i) The inverse-variance weighted, weighted median, Egger, and penalised weighted median MR analyses for BMI using FinnGen and published GWAS. (**g**) (ii) The inverse-variance weighted, weighted median, Egger, and penalised weighted median MR analyses for body fat percentage using FinnGen and published GWAS. (**g**) (iii) The inverse-variance weighted, weighted median, Egger, and penalised weighted median MR analyses for FA using FinnGen and published GWAS. (**g**) (iv) The inverse-variance weighted, weighted median, Egger, and penalised weighted median MR analyses for UFA using FinnGen and published GWAS. (**h**) The inverse-variance weighted MR analysis of identified diseases from UK Biobank for BMI, body fat percentage, FA, and UFA clusters. PMID, PubMed ID; N, sample size; OPCS, operating procedure codes; SE, standard error; p, p-value; OR, odds ratio; 95% CI, 95% confidence interval; Q, Q-statistic; I^2^, I^2^-statistic; LCI, lower 95% confidence interval; UCI, upper 95% confidence interval; Intercept p, intercept p-value; I^2^ MR-Egger, I^2^-statistic MR-Egger.

Transparent reporting form
