# Correction: Disease consequences of higher adiposity uncoupled from its adverse metabolic effects using Mendelian randomisation

**DOI:** 10.7554/eLife.80233

**Published:** 2022-05-18

**Authors:** Susan Martin, Jessica Tyrrell, E Louise Thomas, Matthew J Bown, Andrew R Wood, Robin N Beaumont, Lam C Tsoi, Philip E Stuart, James T Elder, Philip Law, Richard Houlston, Christopher Kabrhel, Nikos Papadimitriou, Marc Gunter, Caroline Bull, Joshua A Bell, Emma E Vincent, Naveed Sattar, Malcolm G Dunlop, Ian PM Tomlinson, Sara Lindström, INVENT consortium, Jimmy D Bell, Timothy Frayling, Hanieh Yaghootkar

**Keywords:** Human

Martin S, Tyrrell J, Thomas EL, Bown MJ, Wood AR, Beaumont RN, Tsoi LC, Stuart PE, Elder JT, Law P, Houlston R, Kabrhel C, Papadimitriou N, Gunter MJ, Bull CJ, Bell JA, Vincent EE, Sattar N, Dunlop MG, Tomlinson IPM, Lindström S, INVENT consortium, Bell JD, Frayling TM, Yaghootkar H. 2022. Disease consequences of higher adiposity uncoupled from its adverse randomisation metabolic effects using Mendelian. *eLife* **11**:e72452. doi: 10.7554/eLife.72452.

Published 25 January 2022

The main analysis in the final paper involved a genome-wide association study (GWAS) summary statistic dataset for venous thromboembolism. This GWAS was conducted by the International Network of Venous Thromboembolism Clinical Research Networks (INVENT) consortium. In addition, Dr Sara Lindström was instrumental in this GWAS and sharing the data. We are therefore formally correcting the eLife paper to include both Dr Lindström and the INVENT consortium on this paper. Both have reviewed the manuscript and support the conclusions, and agree to be responsible for all parts of the paper in its published form.

The details of Dr Lindström’s and the INVENT consortium’s contributions are given below:

Dr Lindström and the INVENT consortium were both instrumental in conducting the GWAS of venous thromboembolism and sharing the associated summary statistic dataset. Both Dr Lindström and the INVENT consortium have reviewed the manuscript and its conclusions.

**New authors list:**

Susan Martin, Jessica Tyrrell, E Louise Thomas, Matthew J Bown, Andrew R Wood, Robin N. Beaumont, Lam C Tsoi, Philip E. Stuart, James T Elder, Philip Law, Richard Houlston, Christopher Kabrhel, Nikos Papadimitriou, Marc J Gunter, Caroline J Bull, Joshua A Bell, Emma E Vincent, Naveed Sattar, Malcolm G Dunlop, Ian PM Tomlinson, Sara Lindström, INVENT consortium, Jimmy D Bell, Timothy M Frayling, Hanieh Yaghootkar.

**Original authors list:**

Susan Martin, Jessica Tyrrell, E Louise Thomas, Matthew J. Bown, Andrew R Wood, Robin N Beaumont, Lam C Tsoi, Philip E Stuart, James T Elder, Philip Law, Richard Houlston, Christopher Kabrhel, Nikos Papadimitriou, Marc J Gunter, Caroline J Bull, Joshua A Bell, Emma E Vincent, Naveed Sattar, Malcolm G Dunlop, Ian PM Tomlinson, Jimmy D Bell, Timothy M Frayling, Hanieh Yaghootkar.

**Details for the omitted authors:**

Sara Lindström

Department of Epidemiology, University of Washington, Seattle, WA, USA.

Division of Public Health Sciences, Fred Hutchinson Cancer Research Center, Seattle, WA, USA.

**Contribution:** Resources, Writing - review and editing

**Competing Interests:** SL declares no competing interests.

INVENT consortium

**Contribution:** Resources, Writing - review and editing

**Competing Interests:** INVENT declares no competing interests.

The article has been corrected accordingly.

