# Supplementary material for: Disease consequences of higher adiposity uncoupled from its adverse metabolic effects using Mendelian randomisation
Source: eLife. 2022 Jan 25;11:e72452. doi: 10.7554/eLife.72452 (PMC8789289; doi:10.7554/eLife.72452)
Supplement: Supplementary file 1. — (a) Characterisation of monogenic obesity, lipodystrophy, unfavourable adiposity (UFA), and favourable adiposity (FA) using body fat percentage and a selection of metabolic biomarkers. (b) Mendelian randomisation (MR) studies testing the role of obesity (usually as body mass index [BMI]) identified in literature search. (c) (i) Summary statistics of published genome-wide association studies (GWAS) used. Mean (standard deviation [SD] or range) are given for continuous study characteristics where available, mean ranges are given for meta-analyses unless otherwise specified. *Statistics represent only UK Biobank cohort of those included in meta-analysis. (c) (ii) Summary statistics of FinnGen studies used. Mean age of cases is given where available, BMI is not adjusted for, and UK Biobank is not included in these studies. ICD codes taken from hospital discharge register and/or causes of death register. (c) (iii) Summary statistics of UK Biobank studies used. Mean (SD) are given for continuous study characteristics of cases. Self-report code is from n_20002_* variable in UK Biobank. (d) The summary of 73 BMI and 696 body fat percentage genetic variants, the latter including 36 FA and 38 UFA genetic variants. Beta, SE, and p are from the GWAS of BMI and body fat percentage in UK Biobank, respectively. BMI variants were discovered using non-UK Biobank cohorts, and so some SNPs listed may have zero effect size in the UK Biobank GWAS of BMI. (e) The inverse-variance weighted two-sample MR analysis/meta-analysis of 37 identified diseases from published GWAS and/or FinnGen for BMI, body fat percentage, FA, and UFA clusters. Italicised results are those that were interpreted – including all BMI, body fat percentage if a causal effect of BMI was indicated, and FA/UFA if a causal effect of BMI and body fat percentage was indicated. (f) Heterogeneity statistics from random-effects meta-analysis of inverse-variance weighted MR of published GWAS and FinnGen studies. (g) (i) The inv [file elife-72452-supp1.doc]

**Disease consequences of higher adiposity uncoupled from its adverse metabolic effects using Mendelian randomisation.**

Susan Martin^1^, Jessica Tyrrell^1^, E. Louise Thomas^2^, Matthew J. Bown^3,4^, Andrew R. Wood^1^, Robin N. Beaumont^1^, Lam C. Tsoi^5^, Philip E. Stuart^5^, James T. Elder^5,6^, Philip Law^7^, Richard Houlston^7^, Christopher Kabrhel^8,9^, Nikos Papadimitriou^10^, Marc J. Gunter^10^, Caroline J. Bull^11,12,13^, Joshua A. Bell^11,12^, Emma E. Vincent^11,12,13^, Naveed Sattar^14^, Malcolm G. Dunlop^15,16^, Ian P.M. Tomlinson^17^, Jimmy D. Bell^2^, Timothy M. Frayling^1^, Hanieh Yaghootkar^1,2,18^

1. Institute of Biomedical and Clinical Science, University of Exeter Medical School, Research, Innovation, Learning and Development building, Royal Devon & Exeter Hospital, Barrack Road, Exeter EX2 5DW, UK.

2. Research Centre for Optimal Health, School of Life Sciences, University of Westminster, London, UK.

3. Department of Cardiovascular Sciences, University of Leicester, Leicester, UK.

4. NIHR Leicester Biomedical Research Centre, Leicester, UK.

5. Department of Dermatology, University of Michigan, Ann Arbor, MI, USA.

6. Ann Arbor Veterans Affairs Hospital, Ann Arbor, MI, USA.

7. The Institute of Cancer Research, London, UK.

8. Department of Emergency Medicine, Massachusetts General Hospital, Boston, MA, USA.

9. Department of Emergency Medicine, Harvard Medical School, Boston, MA, USA.

10. Nutrition and Metabolism Branch, International Agency for Research on Cancer, Lyon, France.

11. MRC Integrative Epidemiology Unit at the University of Bristol, Bristol, UK.
12. Population Health Sciences, Bristol Medical School, University of Bristol, UK.
13. School of Cellular and Molecular Medicine, University of Bristol, UK.

14. Institute of Cardiovascular and Medical Sciences, University of Glasgow, Glasgow, UK.

15. University of Edinburgh, Edinburgh, UK.

16. Western General Hospital, Edinburgh, UK.

17. Edinburgh Cancer Research Centre, IGMM, University of Edinburgh, Edinburgh, UK.

18. Centre for Inflammation Research and Translational Medicine (CIRTM), Department of Life Sciences, Brunel University London, Uxbridge, Middlesex UB8 3PH, UK.

**Corresponding authors:**

Hanieh Yaghootkar, Centre for Inflammation Research and Translational Medicine (CIRTM), Department of Life Sciences, Brunel University London, Uxbridge, Middlesex UB8 3PH, UK, Email: [Hanieh.yaghootkar@brunel.ac.uk](mailto:Hanieh.yaghootkar@brunel.ac.uk)

Timothy M Frayling, Institute of Biomedical and Clinical Science, University of Exeter Medical School, Research, Innovation, Learning and Development building, Royal Devon & Exeter Hospital, Barrack Road, Exeter EX2 5DW, UK, Email: [t.m.frayling@exeter.ac.uk](mailto:t.m.frayling@exeter.ac.uk)

**Supplementary File 1a.** Characterisation of monogenic obesity, lipodystrophy, unfavourable adiposity and favourable adiposity using body fat percentage and a selection of metabolic biomarkers.

|  | **Monogenic** | | **Polygenic** | |
| --- | --- | --- | --- | --- |
| **Trait** | **Monogenic obesity** | **Lipodystrophy** | **Unfavourable adiposity** | **Favourable adiposity (opposite to lipodystrophy)** |
| **Body fat percentage** | **↑** | **↓** | **↑** | **↑** |
| **Alanine transaminase** | **↑** | **↑** | **↑** | **↓** |
| **Triglycerides** | **↑** | **↑** | **↑** | **↓** |
| **HDL-cholesterol** | **↓** | **↓** | **↓** | **↑** |
| **Sex hormone-binding globulin** | **↓** | **↓** | **↓** | **↑** |
| **Adiponectin** | **↓** | **↓** | **↓** | **↑** |

**Supplementary File 1b. Mendelian randomisation studies testing the role of obesity (usually as body mass index (BMI)) identified in literature search. PMID: PubMed ID.**

| **Trait** | **PMID** |
| --- | --- |
| Alzheimer's disease | 26079416 |
| Alzheimer's disease | 28609829 |
| Asthma | 24983943 |
| Asthma | WOS:000208733105823 |
| Atopy (asthma, lung function, hay fever) | 28675761 |
| Atrial conduction | 31306056 |
| Atrial fibrillation | 27974350 |
| Atrial fibrillation | 28739817 |
| Atrial fibrillation | 28739818 |
| Barrett's oesophagus | 25269698 |
| Bone health | 30817851 |
| Breast cancer | 27551723 |
| Breast cancer (survival) | 29232439 |
| Cancer | 19542184 |
| Cancer | 27061578 |
| Cancer myeloma | 28622301 |
| Cardiac structure | WOS:000412219400017 |
| Cardiometabolic disease | WOS:000482110800197 |
| Cardiovascular disease risk factors | 30524135 |
| Cardiovascular disease | 18550552 |
| Cardiovascular disease | 24462370 |
| Cardiovascular disease | 28678979 |
| Cardiovascular disease | 30646365 |
| Cardiovascular disease | 31221000 |
| Cardiovascular disease | WOS:000398515600010 |
| Cardiovascular disease | WOS:000429659703539 |
| Childhood obesity | 18336066 |
| Chronic kidney disease | 29483184 |
| Chronic kidney disease in diabetes | WOS:000359481602125 |
| Colorectal cancer | 25976416 |
| Colorectal cancer | WOS:000349906902388 |
| Coronary artery disease | 22563304 |
| Coronary artery disease | 29739994 |
| Coronary artery disease | 31393416 |
| Coronary artery disease | WOS:000478079000268 |
| Coronary artery disease | WOS:000208231602331 |
| Dairy consumption | WOS:000396461300339 |
| Deep vein thrombosis | 25161014 |
| Deep vein thrombosis | 26568383 |
| Deep vein thrombosis | 24703048 |
| Depression | 24809401 |
| Depression | 25656382 |
| DNA methylation | 28095459 |
| Education | 28645627 |
| Endometrial cancer | 27550749 |
| Fetuin-A levels | 23899227 |
| Gallstones | 23775818 |
| Glucose and lipids | 28889241 |
| Gray matter volume | 23998998 |
| H. Pylori | 29089580 |
| Happiness/well being | 21309863 |
| Homocysteine concentration | 31413611 |
| hsCRP | 20714329 |
| Hypertension | 30045251 |
| Income | 30565280 |
| Incontinence | WOS:000333387100025 |
| Inflammation | WOS:000462825100419 |
| Insulin sensitivity | 27569682 |
| Lactase persistence | 27170764 |
| Lactase persistence | 29187354 |
| Lipids | 31306056 |
| Liver disease | 30848805 |
| Lung cancer | 27487993 |
| Lung cancer | 28594918 |
| Lung cancer | WOS:000468818900320 |
| Lung function | WOS:000455567101341 |
| Meningioma | 30670737 |
| Mental health | 29666151 |
| Mortality | 30358150 |
| Mortality | 30957776 |
| Mortality | 31221000 |
| Mortality | WOS:000456722000022 |
| Mortality | 30049458 |
| Multiple sclerosis | 27351487 |
| Multiple sclerosis | 27351631 |
| Multiple sclerosis | 29307294 |
| Multiple sclerosis | WOS:000383267201193 |
| Multiple sclerosis | WOS:000468918500156 |
| Multiple sclerosis | WOS:000453090801321 |
| Multiple sclerosis | WOS:000411279002441 |
| Offspring birth weight | 30064420 |
| Osteoarthritis | 23921993 |
| Ovarian cancer | 27401727 |
| Ovarian cancer | 31213659 |
| Ovarian cancer | WOS:000371578501333 |
| Ovarian cancer | WOS:000371597104265 |
| Pancreatic cancer | 28954280 |
| Pancreatic cancer | 28954281 |
| Parkinson's disease | 28609445 |
| Parkinson's disease | WOS:000402672301010 |
| Peripheral artery disease | 26945778 |
| Physical activity | 27478388 |
| Polycystic ovary syndrome | 30496407 |
| Post-operation complications | WOS:000447760600169 |
| Prostate cancer | 26387087 |
| Psoriasis | 30528826 |
| Psoriasis | 30703100 |
| Psoriasis and other inflammatory skin diseases | WOS:000431188500431 |
| Psychiatric | 27601421 |
| Puberty | WOS:000480262702053 |
| Puberty (menarche) | 29549348 |
| Renal cancer | 30605491 |
| Rheumatoid arthritis | 30710354 |
| Smoking | 29769355 |
| Smoking | 30561638 |
| Socioeconomic status | 26956984 |
| Stroke | 28667182 |
| Testosterone | 28448539 |
| Thyroid cancer | WOS:000468819504259 |
| Thyroid hormone | 26595101 |
| Type 2 diabetes | 26156736 |
| Type 2 diabetes | 27402723 |
| Type 2 diabetes | 29630776 |
| Type 2 diabetes | 30891058 |
| Type 2 diabetes | 31393416 |
| Type 2 diabetes and deep vein thrombosis | 26002927 |
| Type 2 diabetes, hypertension and nervousness | 30707692 |
| Venous thromboembolism (deep vein thrombosis) | 29779052 |
| Vitamin D levels | 23393431 |
| Vitamin D levels | WOS:000296603100842 |

**Supplementary File 1ci. Summary statistics of published GWAS used. Mean (SD or range) are given for continuous study characteristics where available, mean ranges are given for meta-analyses unless otherwise specified. PMID: PubMed ID; N: sample size; BMI: body mass index; SD: standard deviation. *Statistics represent only UK Biobank cohort of those included in meta-analysis.**

| **Disease** | **PMID** | **Case definition** | **N cases** | **N controls** | **Age cases (years)** | **BMI cases (kg/m^2^)** | **BMI adjusted?** | **UK Biobank included?** | **Trans-ethnic?** |
| --- | --- | --- | --- | --- | --- | --- | --- | --- | --- |
| **Abdominal aneurysm** | 27899403 | Cases had an infrarenal aortic diameter greater than 30mm. Excluded abdominal aortic aneurysms secondary to connective tissue diseases. | 10,204 | 107,766 | 68 - 82 (median range) | NA | N | N | N |
| **Adult-onset asthma** | 30929738 | Doctor-diagnosed asthma self-reported in the UK Biobank - identified using data fields 6152, 20002, 41202, 41204. Adult-onset defined as an age of diagnosis between 20 and 60 (excluded those over 60). | 26,582 | 300,671 | 55.8 (40 - 70) | 28.3 | N | Y | N |
| **Alzheimer's disease (1)** | 30617256 | Cases were diagnosed with late-onset Alzheimer's disease or had a parent with Alzheimer’s disease (as recorded in the UK Biobank). | 71,880 | 383,378 | 59.1 - 80.5 | NA | N | Y | N |
| **Alzheimer's disease (2)** | 30820047 | Clinical or autopsy-reported late-onset Alzheimer’s disease. | 35,274 | 59,163 | 71.1 - 83.5 | NA | N | N | N |
| **Atrial fibrillation** | 29892015 | Paroxysmal or permanent atrial fibrillation, or atrial flutter. | 65,446 | 522,744 | NA | NA | N | Y | Y |
| **Breast cancer** | 29059683 | Cases identified from studies participating in the breast cancer division of the OncoArray consortium. | 122,977 | 105,974 | NA | NA | N | N | N |
| **Cardioembolic stroke** | 29531354 | Cardioembolic stroke subdivided from ischemic stroke largely using the Trial of Org 10172 in Acute Stroke Treatment criteria. Excluded subarachnoid haemorrhages. | 7,193 | 406,111 | NA | NA | N | N | N |
| **Child-onset asthma** | 30929738 | Doctor-diagnosed asthma self-reported in the UK Biobank – identified using data fields 6152, 20002, 41202, 41204. Child-onset defined as an age of diagnosis below 20. | 13,962 | 300,671 | 53.9 (38 - 70) | 27.4 | N | Y | N |
| **Chronic kidney disease** | 31152163 | Chronic kidney disease was defined as an estimated glomerular filtration rate (eGFR) below 60 ml min^–1^ per 1.73 m^2^. | 64,164 | 561,055 | 40.9 (9) - 76.4 (5.5) | NA | N | Y | Y |
| **Colon cancer** | 33632709  GECCO | Colon cancer cases comprised combined proximal colon (ICD-9 codes: 153.4, 153.6, 153.0, or 153.1) and distal colon cancer cases (ICD-9 codes: 153.7, 153.2, or 153.3), and additional colon cases with ICD-9 code 153.9. | 32,002 | 64,159 | NA | NA | N | Y | N |
| **Colorectal cancer (1)** | 31089142 | Diagnosis of colorectal cancer followed World Health Organization guidelines, using ICD-9 codes 153 and 154, and ICD-10 code C18.9, C19 and C20. | 34,627 | 71,379 | NA | NA | N | Y | N |
| **Colorectal cancer (2)** | 30510241  GECCO | Details are given in the study description of 30510241 | 58,131 | 67,347 | NA | NA | N | Y | Y |
| **Coronary artery disease** | 26343387 | Diagnosis of an inclusive coronary artery disease (e.g. myocardial infarction, acute coronary syndrome, chronic stable angina or coronary stenosis of more than 50%). | 60,801 | 123,504 | NA | NA | NA | N | Y |
| **Distal colon cancer** | 33632709  GECCO | Distal colon cancer as any primary tumor arising in the splenic flexure, descending colon, or sigmoid colon (ICD-9 codes: 153.7, 153.2, or 153.3 | 14,376 | 64,159 | NA | NA | N | Y | N |
| **Endometrial cancer** | 30093612 | Endometrial cancer cases confirmed via the Cancer Registry or UK Biobank records. Includes both endometrioid histology and non-endometrioid histology. | 12,906 | 108,979 | NA | NA | N | Y | N |
| **Gastro-oesophageal reflux disease** | 31527586 | Gastro-oesophageal reflux disease (GERD) cases identified using ICD-10 codes, self-reported GERD, or use of heartburn and/or GERD medication. | 80,265 | 305,011 | NA | NA | N | Y | N |
| **Gout** | 31578528 | Cases identified using ICD codes for gout, self-reported gout, or use of urate-lowering medications. | 13,179 | 750,634 | 12.9 (3.9) - 76.4 (5.5) | NA | N | Y | Y |
| **Heart failure** | *bioRxiv* (2019): 682013 | Clinical diagnosis of heart failure of any aetiology with no inclusion criteria based on left ventricular ejection fraction. | 47,309 | 930,014 | 34.5 (11.6) - 85.6 (7) | 15.2 (3.92) - 32.3 (7.25) | N | Y | N |
| **Ischemic stroke** | 29531354 | Ischemic stroke cases were defined based on clinical and imaging criteria. Excluded subarachnoid haemorrhages. | 34,217 | 406,111 | NA | NA | N | N | N |
| **Large artery stroke** | 29531354 | Large artery stroke subdivided from ischaemic stroke largely using the Trial of Org 10172 in Acute Stroke Treatment criteria. Excluded subarachnoid haemorrhages. | 4,373 | 406,111 | NA | NA | N | N | N |
| **Major depressive disorder** | 29700475 | Cases met international consensus criteria (DSM-IV, ICD-9, ICD-10) for a diagnosis of major depressive disorder, defined via structured diagnostic instruments in assessments with trained interviewers, clinician-administered checklists, or medical record reviews. Excluded lifetime bipolar disorder or schizophrenia. | 135,458 | 344,901 | NA | NA | N | N | N |
| **Osteoarthritis (all sites)** | 30664745 | Cases were identified based on clinical evidence of osteoarthritis to the level requiring joint replacement, or radiographic evidence of osteoarthritis (defined as a Kellgren–Lawrence grade ≥2). | 77,052 | 378,169 | NA | NA | N | Y | N |
| **Osteoarthritis (hip and/or knee)** | 30664745 | Cases were identified based on clinical evidence of osteoarthritis to the level requiring joint replacement, or radiographic evidence of osteoarthritis (defined as a Kellgren–Lawrence grade ≥2). Cases restricted to osteoarthritis in hip and/or knee only. | 39,427 | 378,169 | NA | NA | N | Y | N |
| **Osteoporosis** | 30598549 | Osteoporosis fracture cases were detected using two methods: Hospital Episodes Statistics linked via NHS Digital, with the primary or secondary diagnosis field containing a hospital-based fracture diagnosis irrespective of mechanism, and self-reported fracture within the previous five years. Excluded fractures of the skull, face, hands and feet, pathological fractures due to malignancy, atypical femoral fractures, periprosthetic, and healed fracture codes. | 421,084 | 737,530 | 55.8 (8.4) - 58.6 (7.6)* | 27.2 (5.2) - 27.7 (4.3)* | N | Y | N |
| **Ovarian cancer - clear cell cancer** | 28346442 | Most cases were sampled via cancer genetics clinics. | 1,366 | 40,941 | NA | NA | N | N | N |
| **Ovarian cancer - endometrioid** | 28346442 | Most cases were sampled via cancer genetics clinics. | 2,810 | 40,941 | NA | NA | N | N | N |
| **Ovarian cancer - epithelial** | 28346442 | Most cases were sampled via cancer genetics clinics. Some of the included studies were restricted to females aged at least 18 years, and with pathogenic variants in *BRCA1* or *BRCA2*. | 29,396 | 68,502 | NA | NA | N | N | N |
| **Ovarian cancer - high grade serous** | 28346442 | Most cases were sampled via cancer genetics clinics. | 13,037 | 40,941 | NA | NA | N | N | N |
| **Ovarian cancer - invasive epithelial** | 28346442 | Most cases were sampled via cancer genetics clinics. | 22,406 | 40,941 | NA | NA | N | N | N |
| **Ovarian cancer - low grade** | 28346442 | Most cases were sampled via cancer genetics clinics. | 1,012 | 40,941 | NA | NA | N | N | N |
| **Ovarian cancer - mucinous ovarian carcinoma** | 28346442 | Most cases were sampled via cancer genetics clinics. | 1,417 | 40,941 | NA | NA | N | N | N |
| **Ovarian cancer - serous invasive** | 28346442 | Most cases were sampled via cancer genetics clinics. | 14,049 | 40,941 | NA | NA | N | N | N |
| **Ovarian cancer - serous low-grade borderline** | 28346442 | Most cases were sampled via cancer genetics clinics. | 1,954 | 40,941 | NA | NA | N | N | N |
| **Parkinson's disease** | 31701892 | Case diagnosis was based on a clinic visit and standard UK Brain Bank criteria, and also included cases that had a family history of Parkinson’s disease or had self-reported diagnosis (in 23andMe). | 56,306 | 1,417,791 | NA | NA | N | Y | N |
| **Polycystic ovary syndrome** | 30566500 | Cases were diagnosed based on NIH or Rotterdam Criteria, or by self-report. The NIH diagnosis criteria require the presence of both ovulatory dysfunction and clinical and/or biochemical hyperandrogenism. | 10,074 | 103,164 | 27.2 (6.5) - 60.5 (7.9) | 23.1 (5.5) - 35.9 (8.5) | Y | N | N |
| **Prostate cancer** | 29892016 | Cases were identified through cancer registries, hospital referrals or self-report questionnaires. Included advanced and aggressive cases, and death due to prostate cancer. | 79,194 | 61,112 | 56.25 (5.71) - 72.39 (5.49) | NA | N | N | N |
| **Proximal colon cancer** | 33632709  GECCO | proximal colon cancer as any primary tumor arising in the cecum, ascending colon, hepatic flexure, or transverse colon (ICD-9 codes: 153.4, 153.6, 153.0, or 153.1) | 15,706 | 64,159 | NA | NA | N | Y | N |
| **Psoriasis** | 28537254 | Dermatologist-diagnosed or self-reported psoriasis (in 23andMe). Includes cases that may have also developed psoriatic arthritis. | 19,032 | 286,769 | NA | NA | N | N | N |
| **Rectal cancer** | 33632709  GECCO | Rectal cancer as any primary tumor arising in the rectum or rectosigmoid junction (ICD-9 codes: 154.1, or 154.0) | 16,212 | 64,159 | NA | NA | N | Y | N |
| **Renal cell carcinoma** | 28598434 | Cases were restricted to invasive renal cell carcinoma (ICD-O-2 and ICD-O-3 topography code C64), including all histological subtypes. Only included diagnosis in adults (at least 18 years old). | 10,784 | 20,406 | NA | NA | N | N | N |
| **Rheumatoid arthritis** | 24390342 | Cases fulfilled the 1987 criteria of the American College of Rheumatology for rheumatoid arthritis diagnosis, or were diagnosed by a professional rheumatologist. | 14,361 | 43,923 | NA | NA | N | N | N |
| **Small vessel stroke** | 29531354 | Small vessel stroke subdivided from ischaemic stroke largely using the Trial of Org 10172 in Acute Stroke Treatment criteria. Excluded subarachnoid haemorrhages. | 5,386 | 406,111 | NA | NA | N | N | N |
| **Stroke** | 29531354 | Stroke cases were diagnosed following the World Health Organization definition - rapidly developing signs of focal (or global) disturbance of cerebral function, lasting over 24 hours or leading to death, and with no apparent cause other than vascular origin. | 40,585 | 406,111 | NA | NA | N | N | N |
| **Type 2 diabetes** | 30297969 | Type 2 diabetes diagnosis was based on diagnostic fasting glucose or HbA1c levels, hospital discharge diagnosis, use of oral diabetes medication or self-report. | 74,124 | 824,006 | 47.4 (13.9) - 75.31 (3.2) | 25.9 (2.8) - 34.3 (8.1) | Y | N | N |
| **Venous thromboembolism** | 31420334 | Cases experienced a venous thromboembolism, either a deep vein thrombosis or pulmonary embolism. | 30,234 | 172,122 | 40.9 - 72.3 | NA | N | N | Y |

**Supplementary File 1cii. Summary statistics of FinnGen studies used. Mean age of cases is given where available, BMI is not adjusted for and UK Biobank is not included in these studies. ICD codes taken from hospital discharge register and/or causes of death register. N: sample size.**

| **Disease** | **Code** | **N cases** | **N controls** | **Age cases (years)** | **Traits included** | **ICD-10** | | **ICD-9** | **ICD-8** | | **ICD-10 Excl.** | **ICD-9 Excl.** | **ICD-8 Excl.** |
| --- | --- | --- | --- | --- | --- | --- | --- | --- | --- | --- | --- | --- | --- |
| **Alzheimer's disease** | G6_ALZHEIMER | 3,060 | 173,839 | 78.91 |  | G30 | | 3310 | $!$ | |  |  |  |
| **Aortic aneurysm** | I9_AORTANEUR | 1,919 | 167,843 | 66.29 |  | I71[1-9] | | 441[1-9]|0930A | 0930|441 | |  |  |  |
| **Osteoarthritis** | M13_ARTHROSIS | 28,783 | 121,222 | 58.37 | M13_ARTHROSIS_POLY|M13_ARTHTROSIS_COX|M13_ARTHROSIS_KNEE|M13_ARTHROSIS_OTH | M1[5-9] | | $!$ | $!$ | |  |  |  |
| **Osteoarthritis (hip)** | COX_ARTHROSIS | 7,950 | 141,876 | 63.04 | PRIM_COXARTHROSIS|SCND_OTH_COXARTHROSIS|M13_ARTHTROSIS_COX |  | |  |  | |  |  |  |
| **Osteoarthritis (knee)** | KNEE_ARTHROSIS | 17,803 | 141,876 | 58.52 | PRIM_KNEEARTHROSIS|SCND_OTH_KNEEARTHROSIS|M13_ARTHROSIS_KNEE |  | |  |  | |  |  |  |
| **Asthma** | J10_ASTHMA | 15,926 | 113,428 | 44.01 |  | J45|J46 | | 493 | 493 | |  |  |  |
| **Atrial fibrillation** | I9_AF | 17,325 | 97,214 | 66.76 | I9_E_DRONE | I48 | | 4273 | 42792 | |  |  |  |
| **Barrett's oesophagus** | K11_BARRET | 333 | 154,218 | 63.26 |  | K227 | | 5301B | $!$ | |  |  |  |
| **Breast cancer** | C3_BREAST | 6,023 | 94,338 | 58.46 |  | C50 | | 174 | 174 | |  |  |  |
| **Cancer myeloma** | CD2_MULTIPLE_MYELOMA_PLASMA_CELL | 418 | 176,481 | 64.64 |  | C90 | | 203 | 203 | |  |  |  |
| **Cardiovascular disease** | FG_CVD | 79,685 | 97,214 | 51.81 | FG_HYPERTENSION|I9_ISCHHEART|I9_PULMEMB|FG_OTHHEART|FG_CEREBVASC|FG_DOAAC|FG_DISVEINLYMPH|FG_OTH |  |  | |  | |  |  |  |
| **Chronic kidney disease** | N14_CHRONKIDNEYDIS | 2,821 | 172,745 | 62.96 | N14_DIALYSIS | N18 | | 585 | $!$ | |  |  |  |
| **Colorectal cancer** | C3_COLORECTAL | 2,435 | 174,464 | 65.26 | C3_COLON|C3_RECTOSIGMOID_JUNCTION|C3_RECTUM |  | |  |  | |  |  |  |
| **Deep vein thrombosis** | I9_PHLETHROMBDVTLOW | 3,592 | 153,951 | 53.57 |  | I802|I803 | | 4511A|4510A|4512A | 4510 | |  |  |  |
| **Depression** | F5_DEPRESSIO | 17,794 | 156,611 | 41.55 |  | F3[2-3] | | 2961|2968|3004 | 79020|2980 | |  |  |  |
| **Endometrial cancer** | C3_CORPUS_UTERI | 879 | 99,482 | 63.40 |  | C54 | | 182 | 1820 | |  |  |  |
| **Gallstones** | K11_CHOLELITH | 15,012 | 158,425 | 51.04 |  | K80 | | 574 | 574 | |  |  |  |
| **Gastro-oesophageal reflux disease** | K11_REFLUX | 10,167 | 154,218 | 54.26 |  | K21 | | 5301A | $!$ | |  |  |  |
| **Gout** | GOUT | 2,505 | 165,656 | 62.35 | M13_GOUT|GOUT_IDIO|GOUT_KIDNEY|GOUT_SCND|GOUT_NOS |  | |  |  | |  |  |  |
| **Heart failure** | I9_HEARTFAIL | 9,576 | 159,286 | 66.47 |  | I50|I110|I130|I132 | | 4029B|428 | 42700|42710|428|7824 |  | |  |  |
| **Hypertension** | FG_HYPERTENSION | 43,576 | 97,214 | 55.82 | I9_HYPTENSESS|I9_HYPTENSPUL|I9_HYPTENSHR|I9_HYPTENS |  | |  |  | |  |  |  |
| **Ischaemic stroke** | I9_STR_EXH | 8,046 | 164,286 | 65.60 | I9_STR_EMBOLIC | I63|I64 | | 4330A|4331A|4339A|4340A|4341A|4349A|436 | 433|434|436 | | I636 |  |  |
| **Ischemic heart disease** | I9_IHD | 25,366 | 151,533 | 62.72 |  | I2[0-5]|I46|R96|R98 | | 41[0-4]|798 | 41[0-4]|798 | |  | 7980A |  |
| **Lung cancer** | C3_BRONCHUS_LUNG | 1,385 | 175,514 | 68.35 |  | C34 | | 162 | 152 | |  | 1620 | 1620 |
| **Meningioma** | CD2_BENIGN_MENINGES | 934 | 175,965 | 59.77 | CD2_BENIGN_MENINGES_CEREBRAL|CD2_BENIGN_MENINGES_SPINAL|CD2_BENIGN_MENINGES_NOS | D32 | |  |  | |  |  |  |
| **Multiple sclerosis** | G6_MS | 805 | 175,660 | 40.37 |  | G35 | | 340 | 34099 | |  |  |  |
| **Osteoporosis** | M13_OSTEOPOROSIS | 2,452 | 169,858 | 65.59 |  | M8[0-2] | | 733[0-1] | 7230|72391 | |  |  |  |
| **Ovarian cancer** | C3_OVARY | 604 | 99,757 | 55.72 |  | C56 | | 183 | 1830 | |  |  |  |
| **Pancreatic cancer** | C3_PANCREAS | 519 | 176,380 | 68.71 |  | C25 | | 157 | 157 | |  |  |  |
| **Parkinson's disease** | PDSTRICT | 1,495 | 175,312 | 67.91 |  | G20 | | 3320A | 34200 | |  |  |  |
| **Peripheral artery disease** | I9_PAD | 5,323 | 167,843 | 65.19 | I9_PERIPH | E105|E115|E125|E135|E145|I702|I739 | | 4402|4439 | 25006|4402|4439 |  | |  |  |
| **Polycystic ovary syndrome** | E4_PCOS | 462 | 96,172 | 28.72 |  | E282 | | 2564 | 25690 | |  |  |  |
| **Prostate cancer** | C3_PROSTATE | 4,754 | 71,784 | 67.84 |  | C61 | | 185 | 185 | |  |  |  |
| **Psoriasis** | L12_PSORIASIS | 3,399 | 171,917 | 48.61 | L12_PSORI_VULG|L12_PSORI_PUSTGEN|L12_PSORI_ACRODERM|L12_PSORI_PUSTUPALM|L12_PSORI_GUTTATE|L12_PSORI_ARTHRO|L12_PSORI_NAS | L40 | |  |  | |  |  |  |
| **Pulmonary embolism** | I9_PULMEMB | 3,016 | 173,597 | 61.56 |  | I26 | | 415 | 450 | |  |  |  |
| **Renal cancer** | C3_KIDNEY_NOTRENALPELVIS | 775 | 176,124 | 62.93 |  | C64 | | 1890 | 1890 | |  |  |  |
| **Rheumatoid arthritis** | M13_RHEUMA | 4,701 | 121,222 | 51.69 |  | M0[5-6] | | 7140A|7140B|7241|7142 | 712[1-3] | |  |  |  |
| **Stroke** | C_STROKE | 14,171 | 133,027 | NA | I9_SAH|I9_ICH|I9_OTHINTRACRA|I9_STR_EXH|I9_STR_SAH|I9_TIA |  | |  |  | |  |  |  |
| **Stroke (excl. subarachnoid hemorrhage)** | I9_STR | 8,877 | 163,535 | 65.36 |  | I61|I63|I64 | | 431|4330A|4331A|4339A|4340A|4341A|4349A|436 | 431|433|434|436 | | I636 |  | 43101|43191 |
| **Thyroid cancer** | C3_THYROID_GLAND | 811 | 176,088 | 49.73 |  | C73 | | 193 | 193 | |  |  |  |
| **Type 2 diabetes** | E4_DM2_STRICT | 23,338 | 148,190 | 60.02 | E4_DM2COMA|E4_DM2KETO|E4_DM2REN|E4_DM2OPTH|E4_DM2NEU|E4_DM2PERIPH|E4_DM2NASCOMP|E4_DM2NOCOMP |  |  | |  | |  |  |  |
| **Venous thromboembolism** | I9_VTE | 6,913 | 169,986 | 56.81 |  | I26|I80|O871|O882 | | 415|451|6713|6714|6732 | 450|451|671|6739 | | I800 | 4510 |  |

**Supplementary File 1ciii. Summary statistics of UK Biobank studies used. Mean (SD) are given for continuous study characteristics of cases. Self-report code is from n_20002_* variable in UK Biobank. N: sample size; BMI: body mass index; SD: standard deviation; OPCS: operating procedure codes.**

| **Disease** | **N cases** | **N controls** | **Age cases (years)** | **BMI cases (kg/m^2^)** | **ICD-10** | **ICD-9** | **Self-report code** | **OPCS code** |
| --- | --- | --- | --- | --- | --- | --- | --- | --- |
| **Asthma** | 58,188 | 392,815 | 56.93 (8.19) | 28.24 (5.36) | J45 | 493 | 1111 |  |
| **Atrial fibrillation** | 3,745 | 447,354 | 62.46 (6.01) | 28.73 (5.26) | I480 I481 I482 I483 I484 | 4273 | 1471 1483 |  |
| **Barrett's oesophagus** | 4,897 | 446,128 | 60.56 (6.90) | 28.25 (4.51) | K227 | 5302 | 1139 |  |
| **Breast cancer** | 14,000 | 230,802 | 59.69 (7.07) | 27.23 (4.93) | C50 | 174 |  |  |
| **Chronic kidney disease** | 8,060 | 442,965 | 62.86 (6.14) | 29.90 (5.74) | N18 | 585 | 1192 |  |
| **Colorectal cancer** | 5,761 | 445,264 | 61.94 (6.19) | 27.86 (4.63) | C18 C19 C20 | 153 1540 1541 |  |  |
| **Coronary artery disease** | 43,054 | 407,969 | 61.95 (6.22) | 29.10 (5.02) | I20 I21 I22 I23 I24 I25 | 410 411 412 413 414 | 1074 1075 | K401 K402 K403 K404 K411 K412 K413 K414 K451 K452 K453 K454 K455 K491 K492 K498 K499 K502 K751 K752 K753 K754 K758 K759 |
| **Deep vein thrombosis** | 9,463 | 441,560 | 60.04 (7.20) | 29.21 (5.68) | I82 | 453 | 1094 |  |
| **Depression** | 34,858 | 111,125 | 54.82 (7.53) | 27.21 (5.07) | Measure of depression from the mental health questionnaire using definition "Depressed.Ever" summarised in Davis et al. (PMID: 32026800; https://data.mendeley.com/datasets/kv677c2th4/3) | | | |
| **Endometrial cancer** | 1,835 | 242,967 | 61.49 (6.05) | 29.91 (6.90) | C54 C55 | 179 182 |  |  |
| **Gallstones** | 21,564 | 429,460 | 59.35 (7.45) | 29.73 (5.53) | K800 K801 K802 | 592 | 1162 |  |
| **Gastro-oesophageal reflux disease** | 46,779 | 404,241 | 59.27 (7.43) | 28.55 (4.92) | K210 K219 |  | 1138 |  |
| **Gout** | 9,338 | 441,685 | 60.59 (6.92) | 30.63 (4.98) | M10 | 274 | 1466 |  |
| **Hypertension** | 101,426 | 349,599 | 61.33 (6.52) | 29.50 (5.27) | I10 | 401 | 1072 |  |
| **Lung cancer** | 2,922 | 448,103 | 62.49 (5.75) | 27.34 (4.80) | C33 C34 | 162 |  |  |
| **Multiple sclerosis** | 2,048 | 448,977 | 55.84 (7.61) | 26.90 (5.02) | G35 | 340 | 1261 |  |
| **Osteoarthritis** | 81,114 | 369,907 | 60.79 (6.66) | 29.01 (5.30) | M15 M16 M17 M18 M19 | 715 | 1465 |  |
| **Osteoporosis** | 14,663 | 436,361 | 62.15 (5.99) | 26.10 (4.93) | M80 M81 M82 | 7330 | 1309 |  |
| **Ovarian cancer** | 1,381 | 243,421 | 59.77 (7.35) | 27.43 (5.34) | C56 C570 C571 C572 C573 C574 | 183 |  |  |
| **Pancreatic cancer** | 888 | 450,137 | 62.02 (6.20) | 28.16 (4.98) | C25 | 157 |  |  |
| **Parkinson's disease** | 1,929 | 449,096 | 63.36 (5.38) | 27.56 (4.56) | G20 | 332 | 1262 |  |
| **Peripheral vascular disease** | 5,626 | 445,399 | 61.07 (7.06) | 27.88 (5.42) | I73 | 443 | 1561 |  |
| **Polycystic ovary syndrome** | 738 | 244,064 | 48.58 (6.96) | 30.48 (7.48) | E282 | 2564 | 1350 |  |
| **Prostate cancer** | 8,976 | 197,247 | 63.48 (4.89) | 27.65 (3.83) | C61 | 185 |  |  |
| **Psoriasis** | 7,303 | 443,713 | 57.45 (7.92) | 28.48 (5.23) | L40 | 696 | 1453 |  |
| **Pulmonary embolism** | 7,650 | 443,374 | 60.37 (7.12) | 29.31 (5.58) | I26 | 4151 | 1093 |  |
| **Renal cancer** | 1,363 | 449,662 | 61.34 (6.17) | 28.96 (5.14) | C64 | 189 |  |  |
| **Rheumatoid arthritis** | 8,543 | 442,482 | 60.05 (7.06) | 28.54 (5.51) | M05 M06 | 7140 | 1464 |  |
| **Stroke** | 11,926 | 439,096 | 61.49 (6.71) | 28.54 (5.12) | I60 I61 I63 I64 | 430 431 434 436 | 1583 1081 1086 1491 |  |
| **Thyroid cancer** | 638 | 450,387 | 58.22 (7.35) | 27.49 (5.19) | C73 | 193 |  |  |
| **Type 2 diabetes** | 14,371 | 428,017 | 61.68 (6.19) | 32.00 (5.75) | E11 | 2500 | 1223 1220 |  |

**Supplementary File 1d.** The summary of 73 body mass index (BMI) and 696 body fat percentage genetic variants, the latter including 36 "favourable adiposity" (FA) and 38 "unfavourable adiposity" (UFA) genetic variants. Beta, SE and P are from the GWAS of BMI and body fat percentage in UK Biobank respectively. BMI variants were discovered using non-UK Biobank cohorts, and so some SNPs listed may have zero effect size in the UK Biobank GWAS of BMI. SE: standard error; P: p-value.

| **Instrument** | **RSID** | **Chromosome** | **Position** | **Effect allele** | **Other allele** | **Beta** | **SE** | **P** |
| --- | --- | --- | --- | --- | --- | --- | --- | --- |
| **BMI** | rs1000940 | 17 | 5283252 | A | G | -0.01 | 0.002 | 4.E-12 |
| **BMI** | rs10132280 | 14 | 25928179 | C | A | 0.02 | 0.002 | 4.E-22 |
| **BMI** | rs1016287 | 2 | 59305625 | T | C | 0.02 | 0.002 | 9.E-18 |
| **BMI** | rs10182181 | 2 | 25150296 | A | G | -0.03 | 0.002 | 4.E-71 |
| **BMI** | rs10733682 | 9 | 129460914 | A | G | 0.01 | 0.002 | 3.E-11 |
| **BMI** | rs10938397 | 4 | 45182527 | A | G | -0.03 | 0.002 | 1.E-50 |
| **BMI** | rs10968576 | 9 | 28414339 | A | G | -0.02 | 0.002 | 4.E-31 |
| **BMI** | rs11057405 | 12 | 122781897 | G | A | 0.03 | 0.003 | 3.E-19 |
| **BMI** | rs11126666 | 2 | 26928811 | G | A | 0.00 | 0.002 | 0.081 |
| **BMI** | rs11165643 | 1 | 96924097 | C | T | -0.02 | 0.002 | 4.E-23 |
| **BMI** | rs11191560 | 10 | 104869038 | T | C | -0.02 | 0.004 | 2.E-11 |
| **BMI** | rs11583200 | 1 | 50559820 | C | T | 0.01 | 0.002 | 1.E-13 |
| **BMI** | rs1167827 | 7 | 75163169 | A | G | -0.02 | 0.002 | 4.E-26 |
| **BMI** | rs11688816 | 2 | 63053048 | G | A | 0.01 | 0.002 | 5.E-09 |
| **BMI** | rs11727676 | 4 | 145659064 | T | C | 0.01 | 0.003 | 0.003 |
| **BMI** | rs11847697 | 14 | 30515112 | C | T | -0.03 | 0.005 | 3.E-08 |
| **BMI** | rs12286929 | 11 | 115022404 | A | G | -0.01 | 0.002 | 2.E-14 |
| **BMI** | rs12401738 | 1 | 78446761 | G | A | -0.02 | 0.002 | 5.E-15 |
| **BMI** | rs12429545 | 13 | 54102206 | G | A | -0.03 | 0.003 | 3.E-23 |
| **BMI** | rs12446632 | 16 | 19935389 | G | A | 0.03 | 0.003 | 9.E-29 |
| **BMI** | rs12566985 | 1 | 75002193 | G | A | 0.02 | 0.002 | 4.E-19 |
| **BMI** | rs12885454 | 14 | 29736838 | C | A | 0.02 | 0.002 | 8.E-18 |
| **BMI** | rs12940622 | 17 | 78615571 | G | A | 0.02 | 0.002 | 3.E-18 |
| **BMI** | rs13021737 | 2 | 632348 | A | G | -0.06 | 0.003 | 7.E-106 |
| **BMI** | rs13078960 | 3 | 85807590 | T | G | -0.02 | 0.002 | 9.E-19 |
| **BMI** | rs13191362 | 6 | 163033350 | A | G | 0.02 | 0.003 | 4.E-12 |
| **BMI** | rs1516725 | 3 | 185824004 | T | C | -0.03 | 0.003 | 1.E-29 |
| **BMI** | rs1528435 | 2 | 181550962 | C | T | -0.02 | 0.002 | 7.E-15 |
| **BMI** | rs1558902 | 16 | 53803574 | T | A | -0.07 | 0.002 | 6.E-315 |
| **BMI** | rs16851483 | 3 | 141275436 | G | T | -0.03 | 0.004 | 5.E-18 |
| **BMI** | rs16951275 | 15 | 68077168 | T | C | 0.03 | 0.002 | 1.E-38 |
| **BMI** | rs17001654 | 4 | 77129568 | C | G | -0.01 | 0.003 | 1.E-05 |
| **BMI** | rs17024393 | 1 | 110154688 | T | C | -0.07 | 0.006 | 4.E-27 |
| **BMI** | rs17094222 | 10 | 102395440 | T | C | -0.01 | 0.002 | 5.E-09 |
| **BMI** | rs17405819 | 8 | 76806584 | T | C | 0.02 | 0.002 | 3.E-23 |
| **BMI** | rs17724992 | 19 | 18454825 | A | G | 0.02 | 0.002 | 3.E-18 |
| **BMI** | rs1808579 | 18 | 21104888 | C | T | 0.02 | 0.002 | 1.E-23 |
| **BMI** | rs1928295 | 9 | 120378483 | T | C | 0.01 | 0.002 | 2.E-10 |
| **BMI** | rs2033529 | 6 | 40348653 | A | G | -0.02 | 0.002 | 9.E-24 |
| **BMI** | rs2033732 | 8 | 85079709 | T | C | -0.01 | 0.002 | 5.E-08 |
| **BMI** | rs205262 | 6 | 34563164 | A | G | -0.03 | 0.002 | 2.E-40 |
| **BMI** | rs2075650 | 19 | 45395619 | A | G | 0.02 | 0.003 | 1.E-17 |
| **BMI** | rs2112347 | 5 | 75015242 | T | G | 0.03 | 0.002 | 5.E-44 |
| **BMI** | rs2121279 | 2 | 143043285 | C | T | -0.01 | 0.003 | 0.007 |
| **BMI** | rs2176598 | 11 | 43864278 | T | C | 0.02 | 0.002 | 4.E-21 |
| **BMI** | rs2207139 | 6 | 50845490 | A | G | -0.04 | 0.003 | 4.E-53 |
| **BMI** | rs2245368 | 7 | 76608143 | C | T | 0.02 | 0.003 | 3.E-18 |
| **BMI** | rs2287019 | 19 | 46202172 | C | T | 0.03 | 0.003 | 1.E-37 |
| **BMI** | rs2365389 | 3 | 61236462 | C | T | 0.02 | 0.002 | 6.E-16 |
| **BMI** | rs2650492 | 16 | 28333411 | G | A | -0.02 | 0.002 | 3.E-18 |
| **BMI** | rs2820292 | 1 | 201784287 | A | C | -0.02 | 0.002 | 7.E-18 |
| **BMI** | rs29941 | 19 | 34309532 | A | G | -0.02 | 0.002 | 1.E-12 |
| **BMI** | rs3101336 | 1 | 72751185 | T | C | -0.02 | 0.002 | 3.E-30 |
| **BMI** | rs3736485 | 15 | 51748610 | A | G | 0.01 | 0.002 | 3.E-10 |
| **BMI** | rs3810291 | 19 | 47569003 | G | A | -0.03 | 0.002 | 8.E-41 |
| **BMI** | rs3817334 | 11 | 47650993 | C | T | -0.02 | 0.002 | 4.E-37 |
| **BMI** | rs3849570 | 3 | 81792112 | C | A | -0.01 | 0.002 | 5.E-09 |
| **BMI** | rs4256980 | 11 | 8673939 | C | G | -0.02 | 0.002 | 2.E-17 |
| **BMI** | rs4740619 | 9 | 15634326 | T | C | 0.02 | 0.002 | 1.E-23 |
| **BMI** | rs543874 | 1 | 177889480 | A | G | -0.05 | 0.002 | 4.E-89 |
| **BMI** | rs6477694 | 9 | 111932342 | C | T | 0.01 | 0.002 | 2.E-07 |
| **BMI** | rs6567160 | 18 | 57829135 | T | C | -0.05 | 0.002 | 1.E-123 |
| **BMI** | rs657452 | 1 | 49589847 | A | G | 0.02 | 0.002 | 5.E-18 |
| **BMI** | rs6804842 | 3 | 25106437 | A | G | -0.01 | 0.002 | 5.E-12 |
| **BMI** | rs7138803 | 12 | 50247468 | G | A | -0.03 | 0.002 | 7.E-46 |
| **BMI** | rs7141420 | 14 | 79899454 | C | T | -0.02 | 0.002 | 4.E-28 |
| **BMI** | rs7243357 | 18 | 56883319 | T | G | 0.02 | 0.003 | 3.E-12 |
| **BMI** | rs758747 | 16 | 3627358 | C | T | -0.01 | 0.002 | 7.E-08 |
| **BMI** | rs7599312 | 2 | 213413231 | G | A | 0.02 | 0.002 | 2.E-15 |
| **BMI** | rs7899106 | 10 | 87410904 | A | G | -0.03 | 0.004 | 3.E-10 |
| **BMI** | rs9400239 | 6 | 108977663 | T | C | -0.02 | 0.002 | 4.E-17 |
| **BMI** | rs9581854 | 13 | 28017782 | C | T | -0.01 | 0.003 | 5.E-05 |
| **BMI** | rs9925964 | 16 | 31129895 | A | G | 0.02 | 0.002 | 2.E-27 |
| **Body fat percentage** | 10:123402972_CCTTCT_C | 10 | 123402972 | CCTTCT | C | 0.01 | 0.002 | 9.E-09 |
| **Body fat percentage** | 10:130825644_CGT_C | 10 | 130825644 | CGT | C | 0.01 | 0.002 | 5.E-08 |
| **Body fat percentage** | 11:46139679_AGGAT_A | 11 | 46139679 | AGGAT | A | -0.04 | 0.007 | 6.E-09 |
| **Body fat percentage** | 12:133420533_CA_C | 12 | 133420533 | CA | C | -0.01 | 0.002 | 1.E-08 |
| **Body fat percentage** | 12:16463972_AAGAG_A | 12 | 16463972 | AAGAG | A | -0.01 | 0.002 | 3.E-08 |
| **Body fat percentage** | 12:74588713_TA_T | 12 | 74588713 | TA | T | 0.01 | 0.002 | 3.E-08 |
| **Body fat percentage** | 13:64799819_CT_C | 13 | 64799819 | CT | C | -0.01 | 0.002 | 2.E-08 |
| **Body fat percentage** | 13:76386294_TA_T | 13 | 76386294 | TA | T | 0.01 | 0.002 | 2.E-11 |
| **Body fat percentage** | 13:99119385_TAA_T | 13 | 99119385 | TAA | T | 0.01 | 0.002 | 6.E-16 |
| **Body fat percentage** | 14:25933260_TC_T | 14 | 25933260 | TC | T | 0.01 | 0.002 | 2.E-08 |
| **Body fat percentage** | 14:33298731_CA_C | 14 | 33298731 | CA | C | -0.01 | 0.002 | 4.E-17 |
| **Body fat percentage** | 14:41344130_CATGAACAG_C | 14 | 41344130 | CATGAACAG | C | -0.02 | 0.003 | 1.E-09 |
| **Body fat percentage** | 15:95273258_GTT_G | 15 | 95273258 | GTT | G | 0.01 | 0.002 | 6.E-09 |
| **Body fat percentage** | 16:52553888_CAAAA_C | 16 | 52553888 | CAAAA | C | -0.01 | 0.002 | 5.E-08 |
| **Body fat percentage** | 17:31471175_CAAA_C | 17 | 31471175 | CAAA | C | 0.01 | 0.002 | 2.E-10 |
| **Body fat percentage** | 17:61720434_GA_G | 17 | 61720434 | GA | G | 0.01 | 0.002 | 5.E-11 |
| **Body fat percentage** | 17:63960821_AAGGGTGC_A | 17 | 63960821 | AAGGGTGC | A | -0.01 | 0.002 | 4.E-09 |
| **Body fat percentage** | 1:102565758_AT_A | 1 | 102565758 | AT | A | 0.01 | 0.002 | 2.E-08 |
| **Body fat percentage** | 1:195029618_C_CT | 1 | 195029618 | C | CT | 0.01 | 0.002 | 2.E-12 |
| **Body fat percentage** | 1:209524574_TGA_T | 1 | 209524574 | TGA | T | -0.01 | 0.002 | 9.E-09 |
| **Body fat percentage** | 20:16561232_GA_G | 20 | 16561232 | GA | G | -0.01 | 0.002 | 4.E-08 |
| **Body fat percentage** | 20:40075083_TA_T | 20 | 40075083 | TA | T | -0.01 | 0.002 | 3.E-11 |
| **Body fat percentage** | 20:6636986_GAGGAAGGAAGGAAGGA_G | 20 | 6636986 | GAGGAAGGAAGGAAGGA | G | -0.01 | 0.002 | 1.E-08 |
| **Body fat percentage** | 22:18217076_CTT_C | 22 | 18217076 | CTT | C | -0.01 | 0.002 | 3.E-10 |
| **Body fat percentage** | 2:11425387_TA_T | 2 | 11425387 | TA | T | -0.01 | 0.001 | 3.E-08 |
| **Body fat percentage** | 2:50759450_AT_A | 2 | 50759450 | AT | A | -0.01 | 0.002 | 3.E-10 |
| **Body fat percentage** | 3:183513243_ATTTTTTTTTTTTT_A | 3 | 183513243 | ATTTTTTTTTTTTT | A | 0.01 | 0.002 | 7.E-11 |
| **Body fat percentage** | 3:185821409_AACACACACACAC_A | 3 | 185821409 | AACACACACACAC | A | -0.01 | 0.002 | 2.E-10 |
| **Body fat percentage** | 3:47962034_GT_G | 3 | 47962034 | GT | G | -0.01 | 0.002 | 4.E-10 |
| **Body fat percentage** | 3:48703422_CT_C | 3 | 48703422 | CT | C | 0.02 | 0.002 | 4.E-12 |
| **Body fat percentage** | 3:88237473_CAT_C | 3 | 88237473 | CAT | C | -0.02 | 0.002 | 7.E-12 |
| **Body fat percentage** | 4:21758175_CAA_C | 4 | 21758175 | CAA | C | 0.01 | 0.001 | 8.E-09 |
| **Body fat percentage** | 4:25355782_AAAG_A | 4 | 25355782 | AAAG | A | 0.01 | 0.002 | 4.E-12 |
| **Body fat percentage** | 4:28526950_AAAAC_A | 4 | 28526950 | AAAAC | A | 0.02 | 0.002 | 9.E-14 |
| **Body fat percentage** | 5:102833795_GT_G | 5 | 102833795 | GT | G | -0.01 | 0.002 | 2.E-08 |
| **Body fat percentage** | 5:105776549_AT_A | 5 | 105776549 | AT | A | -0.01 | 0.002 | 2.E-08 |
| **Body fat percentage** | 5:128841855_CA_C | 5 | 128841855 | CA | C | -0.02 | 0.003 | 4.E-09 |
| **Body fat percentage** | 5:63940500_CA_C | 5 | 63940500 | CA | C | -0.01 | 0.001 | 4.E-12 |
| **Body fat percentage** | 5:80811501_AT_A | 5 | 80811501 | AT | A | 0.01 | 0.002 | 1.E-09 |
| **Body fat percentage** | 6:126927854_GT_G | 6 | 126927854 | GT | G | 0.01 | 0.001 | 4.E-13 |
| **Body fat percentage** | 6:143187255_CA_C | 6 | 143187255 | CA | C | 0.01 | 0.001 | 8.E-11 |
| **Body fat percentage** | 6:146367900_AT_A | 6 | 146367900 | AT | A | -0.01 | 0.002 | 2.E-08 |
| **Body fat percentage** | 6:24747149_GC_G | 6 | 24747149 | GC | G | 0.01 | 0.003 | 4.E-08 |
| **Body fat percentage** | 7:104885481_AACACAC_A | 7 | 104885481 | AACACAC | A | -0.01 | 0.002 | 8.E-10 |
| **Body fat percentage** | 8:144505206_ACCT_A | 8 | 144505206 | ACCT | A | -0.01 | 0.002 | 2.E-09 |
| **Body fat percentage** | 8:59856160_CT_C | 8 | 59856160 | CT | C | -0.01 | 0.002 | 6.E-09 |
| **Body fat percentage** | 8:79144655_TGTGTGTGC_T | 8 | 79144655 | TGTGTGTGC | T | 0.01 | 0.002 | 4.E-08 |
| **Body fat percentage** | 9:126336452_CT_C | 9 | 126336452 | CT | C | -0.01 | 0.002 | 3.E-08 |
| **Body fat percentage** | 9:35776991_CA_C | 9 | 35776991 | CA | C | 0.01 | 0.002 | 7.E-09 |
| **Body fat percentage** | rs10013793 | 4 | 62742006 | G | T | 0.01 | 0.002 | 2.E-08 |
| **Body fat percentage** | rs1013293 | 1 | 62570321 | G | A | 0.01 | 0.001 | 1.E-22 |
| **Body fat percentage** | rs10139547 | 14 | 88390504 | G | A | -0.01 | 0.002 | 3.E-08 |
| **Body fat percentage** | rs10169594 | 2 | 41637688 | T | C | -0.01 | 0.002 | 5.E-15 |
| **Body fat percentage** | rs10172678 | 2 | 59294558 | T | C | 0.01 | 0.002 | 1.E-20 |
| **Body fat percentage** | rs10204422 | 2 | 133528953 | C | G | -0.01 | 0.002 | 2.E-10 |
| **Body fat percentage** | rs10269783 | 7 | 49616203 | G | A | -0.01 | 0.002 | 2.E-08 |
| **Body fat percentage** | rs1038088 | 17 | 28074563 | T | G | -0.01 | 0.001 | 1.E-15 |
| **Body fat percentage** | rs10411032 | 19 | 1866427 | T | C | 0.01 | 0.002 | 1.E-08 |
| **Body fat percentage** | rs1046080 | 6 | 31595882 | C | A | -0.02 | 0.002 | 4.E-22 |
| **Body fat percentage** | rs10461444 | 5 | 59335217 | A | T | -0.01 | 0.002 | 4.E-09 |
| **Body fat percentage** | rs10496731 | 2 | 135597628 | T | G | 0.01 | 0.002 | 3.E-10 |
| **Body fat percentage** | rs10631643 | 7 | 113013865 | T | TTTC | -0.01 | 0.002 | 3.E-14 |
| **Body fat percentage** | rs10681181 | 9 | 92206788 | T | TTTG | -0.01 | 0.001 | 2.E-12 |
| **Body fat percentage** | rs10681361 | 9 | 136954732 | A | AAAAT | -0.01 | 0.002 | 6.E-10 |
| **Body fat percentage** | rs10685273 | 2 | 207964869 | G | GAAGA | -0.01 | 0.001 | 1.E-08 |
| **Body fat percentage** | rs10745785 | 12 | 97586257 | C | T | 0.01 | 0.002 | 1.E-08 |
| **Body fat percentage** | rs10746056 | 12 | 79526915 | G | C | -0.01 | 0.001 | 2.E-08 |
| **Body fat percentage** | rs10756798 | 9 | 16739763 | C | T | 0.01 | 0.002 | 1.E-18 |
| **Body fat percentage** | rs10766451 | 11 | 2917510 | C | T | -0.01 | 0.002 | 2.E-08 |
| **Body fat percentage** | rs10776693 | 10 | 134902398 | C | T | -0.01 | 0.002 | 4.E-08 |
| **Body fat percentage** | rs10784270 | 12 | 62264742 | T | C | -0.01 | 0.002 | 2.E-09 |
| **Body fat percentage** | rs1078455 | 3 | 108129348 | T | C | -0.01 | 0.002 | 5.E-10 |
| **Body fat percentage** | rs10792 | 17 | 5288179 | A | T | -0.01 | 0.002 | 8.E-11 |
| **Body fat percentage** | rs10803762 | 2 | 161105876 | G | A | -0.01 | 0.002 | 1.E-10 |
| **Body fat percentage** | rs10804146 | 2 | 206061247 | G | C | 0.01 | 0.001 | 1.E-11 |
| **Body fat percentage** | rs10830566 | 11 | 90473957 | A | G | 0.01 | 0.002 | 2.E-09 |
| **Body fat percentage** | rs10854853 | 22 | 48874412 | G | T | -0.01 | 0.001 | 2.E-12 |
| **Body fat percentage** | rs10873302 | 14 | 77547212 | C | T | -0.01 | 0.002 | 2.E-08 |
| **Body fat percentage** | rs10887578 | 10 | 88096047 | G | C | -0.01 | 0.001 | 1.E-10 |
| **Body fat percentage** | rs10892501 | 11 | 119818622 | G | A | 0.01 | 0.002 | 2.E-09 |
| **Body fat percentage** | rs10915840 | 1 | 225668524 | G | A | 0.01 | 0.002 | 4.E-09 |
| **Body fat percentage** | rs10954772 | 8 | 30863938 | T | C | 0.01 | 0.002 | 2.E-12 |
| **Body fat percentage** | rs10973160 | 9 | 36994969 | C | T | 0.01 | 0.002 | 3.E-10 |
| **Body fat percentage** | rs10982884 | 9 | 118462904 | C | T | -0.02 | 0.003 | 3.E-10 |
| **Body fat percentage** | rs10999456 | 10 | 72413827 | C | T | -0.02 | 0.002 | 2.E-21 |
| **Body fat percentage** | rs11012732 | 10 | 21830104 | A | G | -0.02 | 0.002 | 2.E-24 |
| **Body fat percentage** | rs11042030 | 11 | 8690718 | T | C | 0.01 | 0.002 | 6.E-17 |
| **Body fat percentage** | rs11079849 | 17 | 47090785 | C | T | 0.01 | 0.002 | 6.E-17 |
| **Body fat percentage** | rs11085437 | 19 | 9999117 | T | A | -0.01 | 0.001 | 2.E-10 |
| **Body fat percentage** | rs11096549 | 2 | 16607101 | C | T | -0.01 | 0.002 | 4.E-09 |
| **Body fat percentage** | rs11105846 | 12 | 91257268 | G | T | 0.01 | 0.002 | 2.E-10 |
| **Body fat percentage** | rs1111817 | 9 | 23226898 | C | G | 0.01 | 0.002 | 4.E-08 |
| **Body fat percentage** | rs11135450 | 5 | 95554016 | A | G | -0.01 | 0.002 | 2.E-14 |
| **Body fat percentage** | rs11150745 | 17 | 78757626 | A | G | 0.01 | 0.002 | 2.E-15 |
| **Body fat percentage** | rs11165643 | 1 | 96924097 | C | T | -0.01 | 0.002 | 1.E-21 |
| **Body fat percentage** | rs11172113 | 12 | 57527283 | T | C | -0.01 | 0.002 | 5.E-11 |
| **Body fat percentage** | rs11173522 | 12 | 60953472 | C | A | -0.01 | 0.002 | 3.E-09 |
| **Body fat percentage** | rs111884404 | 8 | 87342875 | A | G | -0.01 | 0.002 | 1.E-09 |
| **Body fat percentage** | rs11199266 | 10 | 122061612 | A | G | -0.01 | 0.002 | 3.E-09 |
| **Body fat percentage** | rs11205303 | 1 | 149906413 | T | C | -0.02 | 0.002 | 3.E-35 |
| **Body fat percentage** | rs112108364 | 13 | 86490590 | T | G | -0.01 | 0.002 | 7.E-14 |
| **Body fat percentage** | rs112130918 | 15 | 47952392 | TA | T | 0.01 | 0.002 | 3.E-10 |
| **Body fat percentage** | rs112154095 | 18 | 22204767 | C | T | 0.01 | 0.002 | 4.E-12 |
| **Body fat percentage** | rs11218510 | 11 | 121922587 | G | A | 0.01 | 0.002 | 3.E-08 |
| **Body fat percentage** | rs112323962 | 5 | 284768 | A | AC | -0.02 | 0.003 | 8.E-10 |
| **Body fat percentage** | rs112326082 | 11 | 76479391 | A | AGCCTTCCT | 0.01 | 0.002 | 3.E-09 |
| **Body fat percentage** | rs112454648 | 10 | 70347292 | A | AAAAC | 0.01 | 0.002 | 8.E-11 |
| **Body fat percentage** | rs112712338 | 7 | 65518803 | C | A | -0.01 | 0.002 | 1.E-08 |
| **Body fat percentage** | rs112716157 | 12 | 50907656 | C | CA | 0.01 | 0.002 | 4.E-08 |
| **Body fat percentage** | rs112852122 | 20 | 47498117 | G | A | 0.02 | 0.002 | 1.E-14 |
| **Body fat percentage** | rs113079574 | 4 | 147354089 | C | T | 0.01 | 0.002 | 2.E-09 |
| **Body fat percentage** | rs11343 | 16 | 19279464 | T | G | -0.01 | 0.002 | 5.E-08 |
| **Body fat percentage** | rs11408091 | 7 | 6387863 | A | AG | -0.01 | 0.002 | 3.E-09 |
| **Body fat percentage** | rs11433992 | 6 | 46309760 | C | CT | -0.01 | 0.001 | 1.E-08 |
| **Body fat percentage** | rs114416276 | 2 | 25889076 | C | T | 0.02 | 0.004 | 5.E-08 |
| **Body fat percentage** | rs114529840 | 1 | 150457517 | T | G | 0.03 | 0.003 | 1.E-20 |
| **Body fat percentage** | rs1147345 | 9 | 132212511 | A | T | 0.01 | 0.002 | 7.E-09 |
| **Body fat percentage** | rs114964326 | 2 | 146015229 | G | A | 0.02 | 0.004 | 2.E-08 |
| **Body fat percentage** | rs115092994 | 1 | 46185726 | A | G | 0.03 | 0.004 | 6.E-14 |
| **Body fat percentage** | rs11542009 | 3 | 9517369 | C | T | -0.02 | 0.002 | 4.E-10 |
| **Body fat percentage** | rs115912456 | 5 | 82815158 | A | G | 0.03 | 0.004 | 4.E-14 |
| **Body fat percentage** | rs11603783 | 11 | 122752954 | T | C | -0.01 | 0.002 | 3.E-11 |
| **Body fat percentage** | rs11619393 | 13 | 20262266 | T | C | -0.01 | 0.002 | 3.E-11 |
| **Body fat percentage** | rs11681043 | 2 | 232771343 | A | G | -0.01 | 0.002 | 3.E-08 |
| **Body fat percentage** | rs11682815 | 2 | 110007535 | A | G | -0.01 | 0.002 | 7.E-10 |
| **Body fat percentage** | rs116903188 | 7 | 74347183 | T | G | 0.02 | 0.003 | 2.E-12 |
| **Body fat percentage** | rs11707955 | 3 | 41308348 | T | C | -0.01 | 0.001 | 5.E-11 |
| **Body fat percentage** | rs117103243 | 11 | 48923731 | C | T | -0.02 | 0.003 | 3.E-09 |
| **Body fat percentage** | rs117176448 | 8 | 27261138 | C | G | -0.02 | 0.003 | 1.E-11 |
| **Body fat percentage** | rs11755266 | 6 | 35162141 | C | T | -0.03 | 0.003 | 4.E-26 |
| **Body fat percentage** | rs11781222 | 8 | 23389571 | T | C | 0.02 | 0.002 | 6.E-13 |
| **Body fat percentage** | rs11782074 | 8 | 142617096 | G | T | -0.01 | 0.002 | 8.E-12 |
| **Body fat percentage** | rs11782341 | 8 | 4813459 | A | G | -0.01 | 0.002 | 2.E-08 |
| **Body fat percentage** | rs11786089 | 8 | 21975521 | A | G | -0.01 | 0.001 | 1.E-12 |
| **Body fat percentage** | rs11790018 | 9 | 129702842 | C | G | 0.01 | 0.002 | 1.E-12 |
| **Body fat percentage** | rs11792311 | 9 | 29672405 | G | A | 0.01 | 0.002 | 3.E-10 |
| **Body fat percentage** | rs117959127 | 15 | 80987675 | G | T | 0.01 | 0.002 | 9.E-10 |
| **Body fat percentage** | rs118115428 | 8 | 68278913 | G | T | -0.01 | 0.002 | 9.E-09 |
| **Body fat percentage** | rs1183668 | 13 | 112191837 | C | G | 0.01 | 0.002 | 1.E-12 |
| **Body fat percentage** | rs11855853 | 15 | 78012618 | C | T | 0.01 | 0.002 | 1.E-08 |
| **Body fat percentage** | rs11907932 | 20 | 51148656 | A | G | 0.01 | 0.002 | 2.E-17 |
| **Body fat percentage** | rs11926346 | 3 | 173659090 | A | G | 0.01 | 0.002 | 7.E-10 |
| **Body fat percentage** | rs11951885 | 5 | 86727566 | T | C | -0.03 | 0.005 | 6.E-11 |
| **Body fat percentage** | rs12028517 | 1 | 6661732 | C | A | 0.01 | 0.002 | 4.E-13 |
| **Body fat percentage** | rs12031634 | 1 | 34584393 | G | A | 0.01 | 0.002 | 7.E-10 |
| **Body fat percentage** | rs12042959 | 1 | 243533273 | A | G | 0.01 | 0.002 | 4.E-10 |
| **Body fat percentage** | rs12046534 | 1 | 170709593 | T | G | -0.01 | 0.002 | 9.E-10 |
| **Body fat percentage** | rs12053559 | 2 | 138416391 | T | G | -0.01 | 0.001 | 1.E-09 |
| **Body fat percentage** | rs12072739 | 1 | 98315893 | A | G | -0.01 | 0.002 | 7.E-16 |
| **Body fat percentage** | rs12127506 | 1 | 222075614 | C | A | 0.01 | 0.002 | 8.E-11 |
| **Body fat percentage** | rs12146571 | 11 | 48314077 | C | T | 0.01 | 0.002 | 1.E-11 |
| **Body fat percentage** | rs1229984 | 4 | 100239319 | T | C | -0.03 | 0.005 | 3.E-12 |
| **Body fat percentage** | rs12300276 | 12 | 110697965 | G | A | -0.01 | 0.002 | 3.E-10 |
| **Body fat percentage** | rs12330631 | 3 | 123089834 | C | T | 0.01 | 0.002 | 1.E-15 |
| **Body fat percentage** | rs12375196 | 7 | 103416541 | C | A | -0.01 | 0.002 | 3.E-12 |
| **Body fat percentage** | rs12375898 | 9 | 23843293 | G | A | 0.01 | 0.002 | 4.E-08 |
| **Body fat percentage** | rs12376870 | 9 | 117890567 | G | A | 0.01 | 0.002 | 2.E-10 |
| **Body fat percentage** | rs12419178 | 11 | 117050918 | C | T | -0.02 | 0.003 | 5.E-09 |
| **Body fat percentage** | rs12432026 | 14 | 75282116 | T | G | -0.01 | 0.001 | 5.E-10 |
| **Body fat percentage** | rs12477088 | 2 | 67841326 | T | C | 0.01 | 0.001 | 3.E-14 |
| **Body fat percentage** | rs12478299 | 2 | 193811641 | T | C | 0.01 | 0.002 | 2.E-10 |
| **Body fat percentage** | rs12486983 | 3 | 182733547 | T | C | -0.01 | 0.002 | 3.E-08 |
| **Body fat percentage** | rs1251467 | 1 | 76536649 | T | C | -0.01 | 0.002 | 2.E-08 |
| **Body fat percentage** | rs12538435 | 7 | 71437104 | A | G | 0.01 | 0.002 | 2.E-11 |
| **Body fat percentage** | rs12634936 | 3 | 147716498 | T | C | -0.02 | 0.003 | 1.E-09 |
| **Body fat percentage** | rs12642970 | 4 | 16601492 | G | C | 0.01 | 0.002 | 2.E-08 |
| **Body fat percentage** | rs12711736 | 2 | 112943070 | C | T | 0.01 | 0.002 | 4.E-10 |
| **Body fat percentage** | rs12789119 | 11 | 67262286 | T | A | 0.01 | 0.002 | 2.E-10 |
| **Body fat percentage** | rs12819035 | 12 | 64236002 | T | A | 0.02 | 0.003 | 3.E-09 |
| **Body fat percentage** | rs12885251 | 14 | 99670791 | G | A | 0.01 | 0.002 | 7.E-10 |
| **Body fat percentage** | rs12890931 | 14 | 69753369 | T | G | -0.01 | 0.002 | 9.E-12 |
| **Body fat percentage** | rs12927987 | 16 | 66720206 | A | T | -0.01 | 0.002 | 1.E-08 |
| **Body fat percentage** | rs12939514 | 17 | 46194264 | T | C | 0.02 | 0.003 | 2.E-17 |
| **Body fat percentage** | rs12945575 | 17 | 40713071 | C | T | -0.01 | 0.002 | 4.E-09 |
| **Body fat percentage** | rs12951079 | 17 | 34933059 | G | A | 0.01 | 0.002 | 1.E-12 |
| **Body fat percentage** | rs1296328 | 4 | 137083193 | A | C | 0.01 | 0.002 | 6.E-15 |
| **Body fat percentage** | rs12997625 | 2 | 202970250 | C | T | -0.01 | 0.001 | 7.E-12 |
| **Body fat percentage** | rs13062093 | 3 | 35667057 | T | G | -0.01 | 0.002 | 1.E-11 |
| **Body fat percentage** | rs13160735 | 5 | 144485978 | G | A | -0.01 | 0.002 | 4.E-10 |
| **Body fat percentage** | rs1322842 | 6 | 20488897 | A | G | 0.01 | 0.002 | 4.E-15 |
| **Body fat percentage** | rs13259730 | 8 | 10578041 | A | G | 0.01 | 0.001 | 5.E-09 |
| **Body fat percentage** | rs1327805 | 9 | 120896164 | A | T | -0.01 | 0.002 | 2.E-08 |
| **Body fat percentage** | rs13329943 | 16 | 24733751 | C | T | -0.01 | 0.002 | 1.E-15 |
| **Body fat percentage** | rs13410783 | 2 | 36789166 | A | G | -0.01 | 0.002 | 8.E-15 |
| **Body fat percentage** | rs13427822 | 2 | 213414265 | A | G | 0.01 | 0.002 | 3.E-08 |
| **Body fat percentage** | rs1348252 | 3 | 42418752 | C | T | 0.01 | 0.002 | 2.E-11 |
| **Body fat percentage** | rs1373349 | 18 | 63282992 | C | T | 0.01 | 0.002 | 3.E-12 |
| **Body fat percentage** | rs1377184 | 1 | 215246432 | A | T | -0.01 | 0.002 | 8.E-13 |
| **Body fat percentage** | rs140183533 | 1 | 106877379 | A | C | 0.01 | 0.001 | 1.E-08 |
| **Body fat percentage** | rs141285286 | 4 | 120134796 | G | GATATAT | -0.01 | 0.002 | 5.E-09 |
| **Body fat percentage** | rs143020014 | 9 | 86463210 | G | T | 0.01 | 0.002 | 2.E-09 |
| **Body fat percentage** | rs143281807 | 4 | 145951966 | T | C | 0.02 | 0.004 | 4.E-08 |
| **Body fat percentage** | rs1436348 | 3 | 104612668 | A | G | -0.01 | 0.002 | 6.E-13 |
| **Body fat percentage** | rs1438945 | 5 | 152510937 | T | A | 0.01 | 0.002 | 1.E-08 |
| **Body fat percentage** | rs1441264 | 13 | 79580919 | G | A | -0.01 | 0.002 | 9.E-14 |
| **Body fat percentage** | rs1446585 | 2 | 136407479 | A | G | 0.01 | 0.002 | 1.E-08 |
| **Body fat percentage** | rs1453055 | 2 | 181329491 | G | A | -0.01 | 0.002 | 4.E-14 |
| **Body fat percentage** | rs145350287 | 12 | 120907309 | T | A | 0.03 | 0.004 | 1.E-16 |
| **Body fat percentage** | rs1454687 | 3 | 94038085 | C | G | 0.01 | 0.001 | 6.E-20 |
| **Body fat percentage** | rs145698793 | 2 | 57351713 | TACACAC | T | -0.01 | 0.002 | 2.E-08 |
| **Body fat percentage** | rs1469760 | 2 | 204125426 | T | C | 0.01 | 0.001 | 5.E-09 |
| **Body fat percentage** | rs1485554 | 4 | 20120863 | A | G | 0.02 | 0.002 | 1.E-13 |
| **Body fat percentage** | rs149053776 | 17 | 43130624 | G | GGTTT | 0.01 | 0.002 | 5.E-13 |
| **Body fat percentage** | rs1491592 | 3 | 70654244 | C | T | -0.01 | 0.001 | 3.E-09 |
| **Body fat percentage** | rs149820955 | 15 | 87659256 | C | T | 0.04 | 0.007 | 4.E-08 |
| **Body fat percentage** | rs1503526 | 5 | 63020706 | T | C | -0.01 | 0.001 | 3.E-13 |
| **Body fat percentage** | rs1526573 | 12 | 18202053 | A | C | 0.01 | 0.002 | 2.E-10 |
| **Body fat percentage** | rs1568488 | 3 | 153657951 | G | C | -0.01 | 0.002 | 2.E-14 |
| **Body fat percentage** | rs159030 | 5 | 94212620 | A | C | -0.01 | 0.002 | 7.E-09 |
| **Body fat percentage** | rs159961 | 1 | 8484228 | C | T | -0.01 | 0.002 | 8.E-11 |
| **Body fat percentage** | rs16846136 | 2 | 212291371 | C | A | -0.01 | 0.002 | 1.E-10 |
| **Body fat percentage** | rs16916303 | 9 | 30823761 | A | G | 0.02 | 0.002 | 1.E-11 |
| **Body fat percentage** | rs16966801 | 17 | 39573713 | A | G | -0.01 | 0.002 | 2.E-08 |
| **Body fat percentage** | rs16975918 | 18 | 39914522 | C | T | -0.01 | 0.002 | 4.E-12 |
| **Body fat percentage** | rs16996657 | 20 | 15816236 | T | C | -0.01 | 0.002 | 1.E-10 |
| **Body fat percentage** | rs17016133 | 3 | 25313167 | T | C | 0.01 | 0.002 | 9.E-09 |
| **Body fat percentage** | rs17172722 | 7 | 46620312 | C | T | 0.01 | 0.002 | 2.E-11 |
| **Body fat percentage** | rs1717776 | 11 | 30415776 | A | G | 0.01 | 0.002 | 2.E-09 |
| **Body fat percentage** | rs17256211 | 14 | 23754580 | A | G | -0.01 | 0.002 | 1.E-08 |
| **Body fat percentage** | rs1731260 | 2 | 26953354 | G | T | -0.01 | 0.001 | 3.E-14 |
| **Body fat percentage** | rs17449259 | 7 | 50733714 | G | T | -0.02 | 0.003 | 2.E-09 |
| **Body fat percentage** | rs17491275 | 1 | 39672545 | T | G | -0.01 | 0.002 | 4.E-08 |
| **Body fat percentage** | rs17704028 | 7 | 95140031 | C | T | 0.01 | 0.002 | 3.E-10 |
| **Body fat percentage** | rs17744603 | 17 | 52926401 | C | G | -0.01 | 0.002 | 1.E-08 |
| **Body fat percentage** | rs17770336 | 9 | 28414625 | C | T | -0.02 | 0.002 | 2.E-21 |
| **Body fat percentage** | rs17820010 | 5 | 50384662 | T | G | -0.01 | 0.002 | 3.E-12 |
| **Body fat percentage** | rs1808629 | 8 | 73435964 | G | A | 0.02 | 0.002 | 2.E-24 |
| **Body fat percentage** | rs1841025 | 12 | 41846272 | T | C | -0.01 | 0.001 | 3.E-13 |
| **Body fat percentage** | rs1854662 | 6 | 120209860 | C | G | -0.01 | 0.002 | 6.E-09 |
| **Body fat percentage** | rs1867458 | 5 | 179597784 | A | G | -0.01 | 0.002 | 4.E-08 |
| **Body fat percentage** | rs187067151 | 20 | 29539588 | G | T | 0.02 | 0.004 | 2.E-08 |
| **Body fat percentage** | rs1881505 | 11 | 1483177 | T | C | 0.02 | 0.003 | 7.E-09 |
| **Body fat percentage** | rs188242066 | 16 | 2803298 | C | T | -0.05 | 0.007 | 4.E-12 |
| **Body fat percentage** | rs188955288 | 19 | 4067314 | C | T | 0.02 | 0.002 | 5.E-15 |
| **Body fat percentage** | rs189194497 | 1 | 174347661 | T | C | 0.06 | 0.008 | 5.E-12 |
| **Body fat percentage** | rs191034207 | 11 | 50706566 | A | G | 0.04 | 0.008 | 2.E-08 |
| **Body fat percentage** | rs1970341 | 6 | 141361290 | G | A | -0.02 | 0.003 | 3.E-09 |
| **Body fat percentage** | rs1988145 | 5 | 92232724 | A | C | 0.01 | 0.002 | 2.E-09 |
| **Body fat percentage** | rs199569565 | 11 | 130749351 | TAG | T | -0.01 | 0.001 | 2.E-15 |
| **Body fat percentage** | rs199764228 | 15 | 48745490 | AC | A | -0.01 | 0.002 | 4.E-09 |
| **Body fat percentage** | rs200119412 | 8 | 77224420 | T | C | -0.02 | 0.002 | 3.E-24 |
| **Body fat percentage** | rs2002023 | 10 | 76848524 | C | T | -0.01 | 0.002 | 4.E-09 |
| **Body fat percentage** | rs2003655 | 1 | 11923338 | G | C | -0.01 | 0.002 | 2.E-10 |
| **Body fat percentage** | rs200442988 | 17 | 44566824 | T | TTTTG | 0.01 | 0.002 | 9.E-14 |
| **Body fat percentage** | rs200800095 | 10 | 77630139 | C | CAT | -0.01 | 0.002 | 8.E-11 |
| **Body fat percentage** | rs200882902 | 2 | 228996202 | C | CT | -0.01 | 0.002 | 1.E-17 |
| **Body fat percentage** | rs200915692 | 3 | 196115344 | G | GT | -0.01 | 0.002 | 1.E-10 |
| **Body fat percentage** | rs2026752 | 13 | 31012459 | G | T | -0.01 | 0.002 | 5.E-12 |
| **Body fat percentage** | rs2028506 | 8 | 122652136 | G | C | 0.01 | 0.001 | 4.E-09 |
| **Body fat percentage** | rs2043016 | 2 | 198146381 | C | T | -0.01 | 0.002 | 2.E-11 |
| **Body fat percentage** | rs2063521 | 20 | 53459627 | A | T | 0.01 | 0.002 | 2.E-09 |
| **Body fat percentage** | rs207301 | 4 | 19095959 | G | A | -0.01 | 0.002 | 1.E-08 |
| **Body fat percentage** | rs2077569 | 1 | 103350876 | G | A | 0.01 | 0.002 | 3.E-10 |
| **Body fat percentage** | rs2108635 | 12 | 2159556 | A | G | -0.01 | 0.002 | 1.E-09 |
| **Body fat percentage** | rs2129475 | 2 | 172916772 | A | G | -0.01 | 0.002 | 1.E-10 |
| **Body fat percentage** | rs2133561 | 5 | 139086651 | A | T | 0.01 | 0.002 | 1.E-10 |
| **Body fat percentage** | rs2146641 | 13 | 53493728 | C | T | 0.01 | 0.002 | 1.E-08 |
| **Body fat percentage** | rs215669 | 7 | 32378979 | G | A | 0.01 | 0.002 | 8.E-15 |
| **Body fat percentage** | rs2172131 | 10 | 133978962 | T | C | 0.01 | 0.002 | 1.E-12 |
| **Body fat percentage** | rs217672 | 14 | 62361021 | A | C | -0.01 | 0.002 | 4.E-15 |
| **Body fat percentage** | rs2181938 | 6 | 23716153 | C | T | 0.01 | 0.002 | 4.E-08 |
| **Body fat percentage** | rs2192527 | 4 | 18329824 | A | G | -0.01 | 0.001 | 4.E-21 |
| **Body fat percentage** | rs2246490 | 14 | 103984294 | G | T | 0.01 | 0.002 | 1.E-09 |
| **Body fat percentage** | rs2281819 | 6 | 33771673 | T | A | 0.01 | 0.002 | 4.E-14 |
| **Body fat percentage** | rs2283093 | 7 | 126721231 | C | T | -0.01 | 0.002 | 3.E-08 |
| **Body fat percentage** | rs228446 | 6 | 134901441 | T | C | -0.01 | 0.002 | 2.E-08 |
| **Body fat percentage** | rs2287214 | 12 | 108090518 | A | G | -0.01 | 0.002 | 2.E-16 |
| **Body fat percentage** | rs2289379 | 7 | 44804225 | C | T | 0.01 | 0.002 | 7.E-10 |
| **Body fat percentage** | rs2292238 | 12 | 56493822 | A | C | 0.01 | 0.002 | 5.E-08 |
| **Body fat percentage** | rs2298777 | 18 | 44343254 | C | T | -0.01 | 0.001 | 3.E-08 |
| **Body fat percentage** | rs2319849 | 16 | 64717191 | C | G | 0.01 | 0.002 | 2.E-08 |
| **Body fat percentage** | rs2370959 | 3 | 27397148 | G | A | -0.01 | 0.002 | 3.E-10 |
| **Body fat percentage** | rs2371767 | 3 | 64718258 | G | C | -0.01 | 0.002 | 2.E-12 |
| **Body fat percentage** | rs238675 | 1 | 42470274 | G | A | 0.01 | 0.002 | 4.E-12 |
| **Body fat percentage** | rs2456523 | 15 | 53082481 | T | G | -0.01 | 0.002 | 1.E-11 |
| **Body fat percentage** | rs2474896 | 6 | 51760527 | C | T | -0.01 | 0.001 | 4.E-16 |
| **Body fat percentage** | rs2481899 | 14 | 56460686 | A | G | -0.01 | 0.002 | 6.E-09 |
| **Body fat percentage** | rs249612 | 5 | 66200783 | C | T | -0.01 | 0.002 | 4.E-11 |
| **Body fat percentage** | rs252749 | 5 | 77389973 | G | A | 0.01 | 0.002 | 4.E-08 |
| **Body fat percentage** | rs254027 | 5 | 103940415 | T | A | -0.01 | 0.001 | 9.E-12 |
| **Body fat percentage** | rs2546109 | 5 | 112162299 | C | A | 0.01 | 0.002 | 1.E-09 |
| **Body fat percentage** | rs256112 | 5 | 52880175 | G | A | 0.01 | 0.002 | 2.E-12 |
| **Body fat percentage** | rs2596121 | 8 | 76660225 | G | A | 0.01 | 0.002 | 4.E-20 |
| **Body fat percentage** | rs2612012 | 3 | 53745625 | A | C | 0.01 | 0.002 | 3.E-10 |
| **Body fat percentage** | rs2620829 | 19 | 5150934 | T | G | 0.01 | 0.002 | 3.E-09 |
| **Body fat percentage** | rs2678204 | 1 | 201800511 | T | G | -0.01 | 0.002 | 3.E-18 |
| **Body fat percentage** | rs2712169 | 2 | 217671349 | G | A | 0.01 | 0.002 | 4.E-10 |
| **Body fat percentage** | rs2717609 | 8 | 143769252 | A | T | 0.01 | 0.002 | 4.E-11 |
| **Body fat percentage** | rs2798297 | 4 | 3064004 | G | A | -0.01 | 0.002 | 3.E-11 |
| **Body fat percentage** | rs2812001 | 1 | 84641304 | A | C | 0.01 | 0.002 | 9.E-09 |
| **Body fat percentage** | rs281827 | 1 | 16810301 | T | C | -0.01 | 0.002 | 5.E-12 |
| **Body fat percentage** | rs2824002 | 21 | 18092738 | C | T | 0.01 | 0.002 | 1.E-10 |
| **Body fat percentage** | rs28433072 | 4 | 56269683 | A | C | -0.01 | 0.001 | 1.E-14 |
| **Body fat percentage** | rs28457808 | 7 | 99023246 | C | G | 0.01 | 0.002 | 1.E-12 |
| **Body fat percentage** | rs28472946 | 8 | 8443173 | T | G | 0.01 | 0.002 | 2.E-08 |
| **Body fat percentage** | rs28482057 | 6 | 70336847 | T | A | 0.01 | 0.002 | 2.E-10 |
| **Body fat percentage** | rs2855818 | 17 | 42290015 | G | A | -0.02 | 0.002 | 1.E-21 |
| **Body fat percentage** | rs28558703 | 4 | 26323082 | G | C | 0.01 | 0.002 | 4.E-09 |
| **Body fat percentage** | rs2957668 | 11 | 10404382 | T | C | 0.01 | 0.001 | 4.E-13 |
| **Body fat percentage** | rs2966859 | 16 | 85324544 | A | G | 0.01 | 0.002 | 4.E-09 |
| **Body fat percentage** | rs2991519 | 14 | 102189932 | A | C | -0.02 | 0.003 | 2.E-10 |
| **Body fat percentage** | rs302189 | 7 | 18301970 | A | C | 0.01 | 0.002 | 2.E-08 |
| **Body fat percentage** | rs3045391 | 22 | 41867105 | T | TTGTC | 0.01 | 0.002 | 3.E-15 |
| **Body fat percentage** | rs3085964 | 2 | 242021008 | A | AGGTCC | -0.01 | 0.002 | 3.E-09 |
| **Body fat percentage** | rs308911 | 2 | 86855977 | A | G | 0.01 | 0.002 | 4.E-09 |
| **Body fat percentage** | rs3113509 | 4 | 52932825 | C | T | 0.01 | 0.002 | 3.E-09 |
| **Body fat percentage** | rs3122160 | 6 | 55084697 | G | T | 0.01 | 0.002 | 1.E-08 |
| **Body fat percentage** | rs314279 | 6 | 105402083 | C | A | 0.02 | 0.002 | 1.E-11 |
| **Body fat percentage** | rs3215750 | 2 | 230633572 | T | TAAATC | -0.01 | 0.002 | 2.E-19 |
| **Body fat percentage** | rs329651 | 11 | 133767622 | G | T | -0.01 | 0.002 | 1.E-08 |
| **Body fat percentage** | rs33807 | 3 | 45334692 | G | A | -0.01 | 0.002 | 2.E-08 |
| **Body fat percentage** | rs33994795 | 8 | 9473429 | C | T | 0.01 | 0.002 | 2.E-10 |
| **Body fat percentage** | rs340025 | 15 | 60908307 | T | C | -0.01 | 0.002 | 5.E-09 |
| **Body fat percentage** | rs34045894 | 10 | 33967489 | G | A | -0.01 | 0.002 | 4.E-10 |
| **Body fat percentage** | rs34172563 | 3 | 30067884 | C | CTT | -0.01 | 0.001 | 2.E-08 |
| **Body fat percentage** | rs34234296 | 2 | 175166636 | G | A | 0.01 | 0.002 | 8.E-15 |
| **Body fat percentage** | rs34292685 | 11 | 64049021 | C | T | 0.02 | 0.002 | 8.E-15 |
| **Body fat percentage** | rs34388845 | 6 | 28578286 | A | G | -0.01 | 0.002 | 3.E-12 |
| **Body fat percentage** | rs34450535 | 1 | 112316030 | C | CT | -0.01 | 0.002 | 3.E-09 |
| **Body fat percentage** | rs34656389 | 4 | 96153214 | A | G | -0.01 | 0.002 | 6.E-12 |
| **Body fat percentage** | rs34688745 | 14 | 47298299 | T | TA | 0.01 | 0.002 | 2.E-10 |
| **Body fat percentage** | rs34696009 | 9 | 96395221 | G | GCTGT | 0.01 | 0.002 | 3.E-08 |
| **Body fat percentage** | rs34847497 | 9 | 28971673 | G | A | 0.01 | 0.002 | 2.E-08 |
| **Body fat percentage** | rs34858588 | 19 | 19457235 | C | G | -0.02 | 0.003 | 1.E-08 |
| **Body fat percentage** | rs34898535 | 16 | 31025641 | C | T | 0.01 | 0.002 | 1.E-21 |
| **Body fat percentage** | rs35099456 | 11 | 66649527 | G | C | 0.02 | 0.003 | 1.E-11 |
| **Body fat percentage** | rs35154152 | 1 | 155172725 | T | C | 0.02 | 0.002 | 4.E-16 |
| **Body fat percentage** | rs35159593 | 12 | 103651399 | G | GA | 0.01 | 0.002 | 5.E-10 |
| **Body fat percentage** | rs35307904 | 9 | 78511889 | G | A | 0.01 | 0.002 | 9.E-09 |
| **Body fat percentage** | rs35343117 | 15 | 86079115 | C | G | 0.01 | 0.002 | 4.E-08 |
| **Body fat percentage** | rs35359254 | 19 | 18901618 | C | G | 0.02 | 0.003 | 3.E-13 |
| **Body fat percentage** | rs35475612 | 10 | 16750948 | C | CTT | -0.01 | 0.002 | 3.E-11 |
| **Body fat percentage** | rs35523808 | 6 | 75834971 | T | A | -0.02 | 0.004 | 7.E-09 |
| **Body fat percentage** | rs35574473 | 1 | 204299016 | C | CA | -0.01 | 0.002 | 5.E-08 |
| **Body fat percentage** | rs35697691 | 15 | 52353498 | C | G | -0.02 | 0.003 | 2.E-15 |
| **Body fat percentage** | rs35939555 | 18 | 13065174 | G | GT | -0.01 | 0.002 | 5.E-11 |
| **Body fat percentage** | rs36045023 | 3 | 81813351 | T | TA | -0.01 | 0.002 | 7.E-12 |
| **Body fat percentage** | rs36090025 | 10 | 114774433 | A | C | 0.01 | 0.002 | 1.E-11 |
| **Body fat percentage** | rs36094062 | 11 | 28260678 | A | T | 0.01 | 0.003 | 6.E-09 |
| **Body fat percentage** | rs369982 | 7 | 94048482 | G | A | -0.01 | 0.001 | 4.E-08 |
| **Body fat percentage** | rs372818134 | 8 | 25711754 | C | CA | 0.01 | 0.002 | 3.E-08 |
| **Body fat percentage** | rs3730071 | 12 | 49168798 | C | A | 0.03 | 0.004 | 1.E-09 |
| **Body fat percentage** | rs3737992 | 1 | 33234128 | G | A | 0.01 | 0.002 | 8.E-09 |
| **Body fat percentage** | rs3743861 | 16 | 89818340 | G | C | 0.01 | 0.002 | 1.E-13 |
| **Body fat percentage** | rs3748126 | 7 | 76632736 | C | G | 0.01 | 0.002 | 5.E-09 |
| **Body fat percentage** | rs374873119 | 10 | 128836112 | CT | C | -0.01 | 0.002 | 1.E-10 |
| **Body fat percentage** | rs3751859 | 16 | 81735012 | G | A | 0.01 | 0.002 | 1.E-09 |
| **Body fat percentage** | rs3754963 | 2 | 166185707 | A | T | 0.01 | 0.002 | 5.E-09 |
| **Body fat percentage** | rs3762444 | 1 | 2427712 | C | T | 0.01 | 0.001 | 6.E-09 |
| **Body fat percentage** | rs376633755 | 8 | 106441929 | C | CAA | -0.01 | 0.002 | 4.E-09 |
| **Body fat percentage** | rs3766823 | 1 | 32197257 | G | A | -0.01 | 0.002 | 4.E-10 |
| **Body fat percentage** | rs3771653 | 2 | 159483811 | C | T | -0.01 | 0.002 | 5.E-09 |
| **Body fat percentage** | rs377404583 | 7 | 11202327 | CT | C | -0.01 | 0.002 | 4.E-08 |
| **Body fat percentage** | rs3803286 | 14 | 103246470 | A | G | 0.01 | 0.002 | 6.E-15 |
| **Body fat percentage** | rs3810291 | 19 | 47569003 | G | A | -0.01 | 0.002 | 1.E-13 |
| **Body fat percentage** | rs3817428 | 15 | 89415247 | C | G | 0.01 | 0.002 | 1.E-18 |
| **Body fat percentage** | rs3840590 | 7 | 77827064 | G | GA | 0.01 | 0.002 | 1.E-13 |
| **Body fat percentage** | rs3856595 | 3 | 66492604 | G | T | 0.01 | 0.001 | 4.E-09 |
| **Body fat percentage** | rs3911063 | 3 | 85906928 | T | C | 0.01 | 0.002 | 2.E-14 |
| **Body fat percentage** | rs3931548 | 9 | 103113652 | C | A | -0.01 | 0.002 | 4.E-12 |
| **Body fat percentage** | rs393308 | 11 | 85135100 | T | C | -0.01 | 0.002 | 2.E-10 |
| **Body fat percentage** | rs394608 | 21 | 46581798 | T | C | -0.01 | 0.002 | 7.E-17 |
| **Body fat percentage** | rs4055791 | 13 | 59266053 | C | T | 0.01 | 0.002 | 2.E-09 |
| **Body fat percentage** | rs410681 | 6 | 42670469 | G | A | -0.01 | 0.002 | 2.E-08 |
| **Body fat percentage** | rs412243 | 16 | 339672 | T | C | 0.01 | 0.002 | 7.E-12 |
| **Body fat percentage** | rs41279738 | 1 | 110082551 | T | G | -0.05 | 0.005 | 4.E-22 |
| **Body fat percentage** | rs41307479 | 9 | 116082647 | C | G | -0.01 | 0.002 | 8.E-12 |
| **Body fat percentage** | rs41310284 | 10 | 102447647 | C | A | 0.02 | 0.002 | 7.E-17 |
| **Body fat percentage** | rs424539 | 9 | 14442595 | C | G | -0.01 | 0.002 | 1.E-11 |
| **Body fat percentage** | rs4253755 | 22 | 46615376 | G | A | -0.02 | 0.002 | 2.E-12 |
| **Body fat percentage** | rs429343 | 2 | 147903382 | A | G | 0.01 | 0.001 | 4.E-13 |
| **Body fat percentage** | rs4307239 | 7 | 24354300 | A | G | -0.01 | 0.001 | 4.E-08 |
| **Body fat percentage** | rs4377779 | 6 | 12117344 | T | C | 0.01 | 0.002 | 1.E-12 |
| **Body fat percentage** | rs4398538 | 4 | 130724740 | T | C | 0.01 | 0.002 | 2.E-09 |
| **Body fat percentage** | rs441792 | 2 | 105404221 | A | G | -0.01 | 0.001 | 1.E-14 |
| **Body fat percentage** | rs4430672 | 14 | 63094407 | T | C | 0.01 | 0.002 | 4.E-09 |
| **Body fat percentage** | rs4477562 | 13 | 54104968 | C | T | -0.02 | 0.002 | 3.E-18 |
| **Body fat percentage** | rs4482463 | 2 | 205375909 | C | A | 0.02 | 0.003 | 4.E-16 |
| **Body fat percentage** | rs4497915 | 2 | 48690596 | T | G | -0.01 | 0.001 | 2.E-08 |
| **Body fat percentage** | rs4500770 | 16 | 74658430 | A | T | 0.01 | 0.002 | 5.E-10 |
| **Body fat percentage** | rs4549685 | 7 | 39326478 | C | T | 0.01 | 0.002 | 3.E-13 |
| **Body fat percentage** | rs4577503 | 3 | 99657922 | G | A | 0.01 | 0.002 | 3.E-09 |
| **Body fat percentage** | rs4580429 | 2 | 46891454 | C | G | 0.01 | 0.002 | 2.E-15 |
| **Body fat percentage** | rs4619804 | 3 | 18674644 | A | C | -0.01 | 0.002 | 2.E-13 |
| **Body fat percentage** | rs4662318 | 2 | 144016529 | C | T | 0.01 | 0.002 | 2.E-09 |
| **Body fat percentage** | rs4668314 | 2 | 171631258 | G | T | 0.01 | 0.002 | 4.E-10 |
| **Body fat percentage** | rs4669869 | 2 | 12898460 | T | C | -0.01 | 0.001 | 3.E-09 |
| **Body fat percentage** | rs4672338 | 2 | 60217457 | C | T | -0.01 | 0.002 | 1.E-08 |
| **Body fat percentage** | rs4677813 | 3 | 194863860 | T | C | 0.01 | 0.002 | 8.E-09 |
| **Body fat percentage** | rs4718964 | 7 | 70038969 | G | T | -0.01 | 0.002 | 2.E-15 |
| **Body fat percentage** | rs4721319 | 7 | 14334844 | A | G | -0.01 | 0.002 | 2.E-08 |
| **Body fat percentage** | rs4722398 | 7 | 3125220 | C | T | -0.01 | 0.002 | 7.E-11 |
| **Body fat percentage** | rs4737188 | 8 | 64756657 | A | T | 0.01 | 0.001 | 3.E-08 |
| **Body fat percentage** | rs4752182 | 10 | 120397131 | A | G | 0.01 | 0.002 | 5.E-08 |
| **Body fat percentage** | rs4780885 | 16 | 20380004 | G | C | 0.01 | 0.001 | 8.E-12 |
| **Body fat percentage** | rs4811602 | 20 | 36849088 | G | A | 0.01 | 0.002 | 6.E-09 |
| **Body fat percentage** | rs4839906 | 6 | 97761611 | C | T | 0.01 | 0.002 | 9.E-12 |
| **Body fat percentage** | rs4894808 | 3 | 171833266 | G | C | 0.01 | 0.002 | 8.E-12 |
| **Body fat percentage** | rs4908676 | 1 | 7737099 | A | G | -0.01 | 0.001 | 2.E-12 |
| **Body fat percentage** | rs4936175 | 11 | 132641959 | T | C | -0.01 | 0.001 | 1.E-08 |
| **Body fat percentage** | rs4946840 | 6 | 107899049 | A | G | -0.01 | 0.002 | 3.E-08 |
| **Body fat percentage** | rs4959613 | 6 | 1835403 | C | A | -0.01 | 0.002 | 9.E-10 |
| **Body fat percentage** | rs505922 | 9 | 136149229 | T | C | -0.01 | 0.002 | 2.E-08 |
| **Body fat percentage** | rs522110 | 10 | 99772885 | A | G | -0.01 | 0.001 | 9.E-19 |
| **Body fat percentage** | rs528296052 | 1 | 213287698 | C | CTGTT | 0.01 | 0.002 | 2.E-10 |
| **Body fat percentage** | rs529200 | 3 | 173114305 | A | G | -0.01 | 0.001 | 6.E-12 |
| **Body fat percentage** | rs530311131 | 3 | 10096319 | C | CA | -0.02 | 0.003 | 1.E-08 |
| **Body fat percentage** | rs534930508 | 14 | 40104783 | A | AGGAGT | -0.01 | 0.002 | 2.E-09 |
| **Body fat percentage** | rs541582524 | 2 | 100827641 | G | GCT | 0.01 | 0.002 | 5.E-19 |
| **Body fat percentage** | rs543933820 | 17 | 79992138 | T | C | -0.01 | 0.003 | 1.E-08 |
| **Body fat percentage** | rs546438579 | 15 | 85266873 | C | CT | -0.01 | 0.002 | 2.E-16 |
| **Body fat percentage** | rs546865674 | 4 | 102197018 | G | GT | -0.02 | 0.002 | 1.E-13 |
| **Body fat percentage** | rs547561306 | 1 | 31570982 | A | AT | -0.02 | 0.003 | 3.E-08 |
| **Body fat percentage** | rs555219016 | 2 | 50215757 | TAAAAA | T | 0.01 | 0.002 | 4.E-11 |
| **Body fat percentage** | rs555996 | 9 | 124396311 | C | G | 0.01 | 0.002 | 3.E-08 |
| **Body fat percentage** | rs55726687 | 12 | 991306 | G | A | -0.01 | 0.002 | 2.E-13 |
| **Body fat percentage** | rs560547656 | 15 | 75443633 | C | CAAAAAA | 0.01 | 0.002 | 3.E-10 |
| **Body fat percentage** | rs56130739 | 10 | 118777907 | G | GA | 0.01 | 0.002 | 2.E-12 |
| **Body fat percentage** | rs56218501 | 20 | 46365636 | C | T | 0.01 | 0.002 | 5.E-15 |
| **Body fat percentage** | rs56351202 | 7 | 5843573 | T | A | 0.02 | 0.003 | 2.E-08 |
| **Body fat percentage** | rs56411094 | 16 | 70444094 | A | AT | -0.01 | 0.001 | 3.E-11 |
| **Body fat percentage** | rs564988630 | 4 | 83192564 | C | CA | 0.01 | 0.001 | 5.E-10 |
| **Body fat percentage** | rs565418819 | 6 | 26890368 | T | C | 0.02 | 0.002 | 5.E-17 |
| **Body fat percentage** | rs566516442 | 5 | 164498450 | G | GT | 0.01 | 0.002 | 2.E-08 |
| **Body fat percentage** | rs56803094 | 15 | 99222509 | A | G | 0.01 | 0.002 | 7.E-12 |
| **Body fat percentage** | rs569541320 | 19 | 30286450 | G | GA | -0.01 | 0.002 | 5.E-17 |
| **Body fat percentage** | rs5742915 | 15 | 74336633 | T | C | 0.01 | 0.001 | 3.E-10 |
| **Body fat percentage** | rs574565290 | 15 | 59129738 | C | CA | -0.01 | 0.002 | 3.E-09 |
| **Body fat percentage** | rs57800857 | 4 | 140863365 | A | C | 0.01 | 0.002 | 9.E-18 |
| **Body fat percentage** | rs57804557 | 7 | 26705632 | A | ACA | -0.02 | 0.003 | 5.E-10 |
| **Body fat percentage** | rs57989773 | 6 | 100629078 | T | C | -0.01 | 0.002 | 5.E-12 |
| **Body fat percentage** | rs58083390 | 4 | 73549559 | C | T | -0.02 | 0.003 | 2.E-14 |
| **Body fat percentage** | rs58243949 | 18 | 52509833 | C | T | -0.01 | 0.002 | 7.E-09 |
| **Body fat percentage** | rs5824977 | 18 | 50441033 | T | TAGA | -0.01 | 0.002 | 3.E-08 |
| **Body fat percentage** | rs58300328 | 2 | 69646357 | G | A | 0.01 | 0.002 | 3.E-18 |
| **Body fat percentage** | rs58312965 | 16 | 6703971 | C | T | -0.01 | 0.002 | 4.E-08 |
| **Body fat percentage** | rs583893 | 11 | 118904233 | G | T | 0.01 | 0.002 | 3.E-11 |
| **Body fat percentage** | rs5849410 | 3 | 61264379 | C | CAT | 0.01 | 0.002 | 1.E-12 |
| **Body fat percentage** | rs5864662 | 4 | 181552417 | A | AT | 0.01 | 0.002 | 2.E-08 |
| **Body fat percentage** | rs59428052 | 2 | 53861389 | A | G | 0.01 | 0.002 | 3.E-08 |
| **Body fat percentage** | rs59499656 | 18 | 40768309 | A | T | 0.01 | 0.002 | 2.E-18 |
| **Body fat percentage** | rs59628956 | 4 | 48807270 | G | A | 0.01 | 0.002 | 4.E-09 |
| **Body fat percentage** | rs59934506 | 20 | 25373782 | C | A | 0.01 | 0.002 | 4.E-16 |
| **Body fat percentage** | rs6031847 | 20 | 43514203 | C | T | -0.01 | 0.002 | 3.E-09 |
| **Body fat percentage** | rs6103254 | 20 | 41990761 | T | C | 0.02 | 0.002 | 6.E-12 |
| **Body fat percentage** | rs61104729 | 6 | 153374165 | C | CT | -0.01 | 0.002 | 5.E-09 |
| **Body fat percentage** | rs61754230 | 12 | 72179446 | C | T | -0.03 | 0.005 | 2.E-09 |
| **Body fat percentage** | rs61782665 | 1 | 47692035 | A | G | 0.01 | 0.002 | 4.E-15 |
| **Body fat percentage** | rs61791109 | 3 | 170734377 | T | C | -0.01 | 0.002 | 1.E-09 |
| **Body fat percentage** | rs61875182 | 10 | 93808142 | T | A | -0.01 | 0.002 | 3.E-08 |
| **Body fat percentage** | rs61903695 | 11 | 89922417 | A | G | -0.01 | 0.002 | 2.E-10 |
| **Body fat percentage** | rs61910767 | 11 | 134515899 | C | T | 0.01 | 0.002 | 1.E-13 |
| **Body fat percentage** | rs61975147 | 14 | 59416558 | T | C | 0.01 | 0.002 | 4.E-12 |
| **Body fat percentage** | rs61979560 | 14 | 30727033 | C | A | -0.01 | 0.002 | 2.E-10 |
| **Body fat percentage** | rs62169721 | 2 | 151471941 | G | T | -0.03 | 0.005 | 5.E-10 |
| **Body fat percentage** | rs62217799 | 20 | 62347191 | G | T | -0.01 | 0.002 | 9.E-16 |
| **Body fat percentage** | rs62244189 | 3 | 44743883 | T | G | -0.01 | 0.002 | 2.E-08 |
| **Body fat percentage** | rs62259475 | 3 | 82710898 | G | A | -0.01 | 0.002 | 7.E-10 |
| **Body fat percentage** | rs62276243 | 3 | 90121534 | C | T | 0.01 | 0.001 | 8.E-17 |
| **Body fat percentage** | rs62413414 | 6 | 80310375 | C | T | -0.01 | 0.002 | 1.E-08 |
| **Body fat percentage** | rs62466588 | 7 | 66458903 | T | C | 0.02 | 0.004 | 3.E-08 |
| **Body fat percentage** | rs62621197 | 19 | 8670147 | C | T | 0.02 | 0.004 | 2.E-09 |
| **Body fat percentage** | rs643428 | 1 | 54728858 | C | T | -0.01 | 0.002 | 9.E-09 |
| **Body fat percentage** | rs6477495 | 9 | 99255608 | G | A | 0.01 | 0.002 | 2.E-08 |
| **Body fat percentage** | rs6545966 | 2 | 62861353 | G | A | -0.01 | 0.001 | 9.E-09 |
| **Body fat percentage** | rs654718 | 11 | 94190115 | T | C | 0.01 | 0.002 | 5.E-08 |
| **Body fat percentage** | rs6561937 | 13 | 58257667 | T | A | 0.01 | 0.002 | 4.E-10 |
| **Body fat percentage** | rs6575340 | 14 | 94023972 | G | A | -0.01 | 0.002 | 9.E-18 |
| **Body fat percentage** | rs6597653 | 9 | 133788465 | G | C | -0.01 | 0.002 | 6.E-09 |
| **Body fat percentage** | rs6688826 | 1 | 80812329 | T | C | -0.01 | 0.002 | 2.E-09 |
| **Body fat percentage** | rs6693294 | 1 | 49879122 | A | G | 0.01 | 0.002 | 3.E-18 |
| **Body fat percentage** | rs6707445 | 2 | 104420858 | G | A | -0.01 | 0.001 | 2.E-10 |
| **Body fat percentage** | rs67518031 | 3 | 88742702 | G | GAA | 0.01 | 0.001 | 1.E-12 |
| **Body fat percentage** | rs6763913 | 3 | 134660284 | T | C | 0.01 | 0.001 | 4.E-08 |
| **Body fat percentage** | rs67802835 | 2 | 113979160 | A | G | -0.01 | 0.002 | 3.E-08 |
| **Body fat percentage** | rs6782581 | 3 | 196979106 | C | G | 0.01 | 0.001 | 9.E-12 |
| **Body fat percentage** | rs67913249 | 5 | 43204126 | C | G | 0.01 | 0.002 | 3.E-11 |
| **Body fat percentage** | rs68177066 | 13 | 81104260 | T | C | -0.01 | 0.002 | 3.E-10 |
| **Body fat percentage** | rs6831020 | 4 | 55500226 | C | A | 0.01 | 0.002 | 8.E-09 |
| **Body fat percentage** | rs6857 | 19 | 45392254 | C | T | 0.02 | 0.002 | 5.E-21 |
| **Body fat percentage** | rs6899218 | 5 | 178988608 | T | A | -0.01 | 0.001 | 1.E-11 |
| **Body fat percentage** | rs6927268 | 6 | 108865663 | T | G | 0.01 | 0.002 | 4.E-14 |
| **Body fat percentage** | rs6938973 | 6 | 98421721 | T | C | -0.02 | 0.002 | 7.E-26 |
| **Body fat percentage** | rs6963767 | 7 | 114402803 | G | A | -0.01 | 0.002 | 3.E-09 |
| **Body fat percentage** | rs6973700 | 7 | 1856669 | A | G | 0.01 | 0.002 | 3.E-12 |
| **Body fat percentage** | rs6986434 | 8 | 61944091 | T | C | 0.01 | 0.002 | 8.E-09 |
| **Body fat percentage** | rs6997359 | 8 | 14220732 | C | T | -0.01 | 0.002 | 2.E-10 |
| **Body fat percentage** | rs699929 | 3 | 157931837 | A | T | 0.01 | 0.002 | 6.E-12 |
| **Body fat percentage** | rs7037043 | 9 | 77160815 | A | G | -0.01 | 0.002 | 4.E-08 |
| **Body fat percentage** | rs7038943 | 9 | 120377178 | T | C | 0.01 | 0.002 | 2.E-09 |
| **Body fat percentage** | rs704061 | 12 | 89771903 | T | C | -0.01 | 0.001 | 8.E-24 |
| **Body fat percentage** | rs7070670 | 10 | 61842645 | C | T | 0.01 | 0.002 | 1.E-08 |
| **Body fat percentage** | rs7081254 | 10 | 132955696 | T | C | 0.01 | 0.002 | 1.E-09 |
| **Body fat percentage** | rs7102705 | 11 | 69143284 | A | G | -0.01 | 0.002 | 5.E-09 |
| **Body fat percentage** | rs71036299 | 2 | 236819547 | A | ATGG | 0.01 | 0.002 | 3.E-09 |
| **Body fat percentage** | rs71474196 | 11 | 46977160 | C | T | 0.02 | 0.002 | 9.E-11 |
| **Body fat percentage** | rs7161194 | 14 | 101529005 | A | G | 0.01 | 0.002 | 2.E-10 |
| **Body fat percentage** | rs7166081 | 15 | 67492301 | G | A | -0.02 | 0.002 | 2.E-20 |
| **Body fat percentage** | rs7191378 | 16 | 58115034 | A | G | -0.01 | 0.002 | 2.E-08 |
| **Body fat percentage** | rs7191938 | 16 | 71407530 | A | G | -0.01 | 0.002 | 2.E-09 |
| **Body fat percentage** | rs7192628 | 16 | 87494858 | C | T | -0.01 | 0.002 | 2.E-08 |
| **Body fat percentage** | rs719802 | 11 | 113234679 | T | C | 0.01 | 0.002 | 6.E-12 |
| **Body fat percentage** | rs7203729 | 16 | 2140010 | A | G | 0.01 | 0.002 | 5.E-12 |
| **Body fat percentage** | rs7206608 | 16 | 82872628 | C | G | -0.01 | 0.002 | 1.E-09 |
| **Body fat percentage** | rs7213608 | 17 | 21279289 | C | T | 0.01 | 0.002 | 4.E-16 |
| **Body fat percentage** | rs7238896 | 18 | 1840658 | A | G | -0.01 | 0.002 | 2.E-10 |
| **Body fat percentage** | rs7239114 | 18 | 45921214 | G | A | -0.01 | 0.002 | 3.E-09 |
| **Body fat percentage** | rs725959 | 9 | 81349608 | G | T | 0.01 | 0.002 | 6.E-10 |
| **Body fat percentage** | rs72634813 | 1 | 1537887 | C | A | 0.01 | 0.002 | 2.E-15 |
| **Body fat percentage** | rs72649373 | 4 | 80609966 | T | C | -0.02 | 0.002 | 6.E-13 |
| **Body fat percentage** | rs72665129 | 13 | 104092350 | T | A | 0.01 | 0.002 | 1.E-08 |
| **Body fat percentage** | rs72681698 | 14 | 51207741 | T | C | 0.05 | 0.007 | 2.E-13 |
| **Body fat percentage** | rs72681869 | 14 | 50655357 | G | C | 0.06 | 0.007 | 1.E-15 |
| **Body fat percentage** | rs72755233 | 15 | 100692953 | G | A | 0.02 | 0.002 | 1.E-10 |
| **Body fat percentage** | rs72767253 | 5 | 51090842 | C | A | 0.03 | 0.004 | 8.E-13 |
| **Body fat percentage** | rs72798148 | 16 | 29926552 | T | C | 0.02 | 0.002 | 7.E-18 |
| **Body fat percentage** | rs72828935 | 10 | 126587488 | G | C | -0.01 | 0.002 | 2.E-12 |
| **Body fat percentage** | rs72851476 | 2 | 142874787 | A | C | 0.01 | 0.002 | 3.E-08 |
| **Body fat percentage** | rs72867447 | 11 | 13301875 | C | G | -0.01 | 0.001 | 5.E-14 |
| **Body fat percentage** | rs730177 | 7 | 67239016 | T | C | -0.01 | 0.002 | 3.E-08 |
| **Body fat percentage** | rs7308788 | 12 | 19240689 | G | A | -0.01 | 0.002 | 3.E-08 |
| **Body fat percentage** | rs73170864 | 7 | 69206288 | C | T | -0.02 | 0.003 | 2.E-09 |
| **Body fat percentage** | rs73196575 | 12 | 113179738 | G | C | -0.01 | 0.002 | 1.E-09 |
| **Body fat percentage** | rs73197346 | 21 | 36770189 | T | C | 0.01 | 0.002 | 6.E-11 |
| **Body fat percentage** | rs73232637 | 7 | 96613246 | A | G | -0.02 | 0.004 | 3.E-09 |
| **Body fat percentage** | rs7327210 | 13 | 98026620 | C | G | 0.01 | 0.002 | 1.E-08 |
| **Body fat percentage** | rs7328213 | 13 | 33377830 | T | C | 0.01 | 0.001 | 6.E-15 |
| **Body fat percentage** | rs73794390 | 5 | 151267073 | A | G | -0.03 | 0.006 | 3.E-08 |
| **Body fat percentage** | rs74548136 | 17 | 79522023 | A | T | -0.01 | 0.002 | 1.E-08 |
| **Body fat percentage** | rs74618095 | 21 | 40683196 | T | C | -0.01 | 0.002 | 3.E-08 |
| **Body fat percentage** | rs746336168 | 1 | 184706212 | GT | G | -0.01 | 0.001 | 5.E-11 |
| **Body fat percentage** | rs746773119 | 1 | 51104117 | CT | C | -0.01 | 0.002 | 5.E-08 |
| **Body fat percentage** | rs747489841 | 16 | 73095430 | AAC | A | 0.01 | 0.002 | 1.E-10 |
| **Body fat percentage** | rs747686078 | 1 | 29453545 | CAAAA | C | -0.02 | 0.002 | 6.E-10 |
| **Body fat percentage** | rs74806710 | 1 | 57833008 | T | A | -0.01 | 0.002 | 2.E-08 |
| **Body fat percentage** | rs7498044 | 15 | 92573639 | G | A | 0.01 | 0.002 | 1.E-09 |
| **Body fat percentage** | rs7499489 | 16 | 9411221 | T | C | -0.01 | 0.002 | 3.E-08 |
| **Body fat percentage** | rs7519259 | 1 | 66434743 | G | A | -0.01 | 0.001 | 4.E-12 |
| **Body fat percentage** | rs75192636 | 10 | 103218567 | A | C | 0.01 | 0.002 | 4.E-08 |
| **Body fat percentage** | rs753461944 | 11 | 57551881 | CA | C | -0.01 | 0.002 | 9.E-09 |
| **Body fat percentage** | rs75412871 | 12 | 121709430 | C | T | 0.02 | 0.003 | 2.E-10 |
| **Body fat percentage** | rs75846784 | 5 | 124536677 | T | G | 0.02 | 0.003 | 6.E-09 |
| **Body fat percentage** | rs7589069 | 2 | 80640417 | C | G | -0.01 | 0.002 | 2.E-08 |
| **Body fat percentage** | rs75983170 | 13 | 40788838 | T | G | -0.01 | 0.002 | 1.E-09 |
| **Body fat percentage** | rs7601895 | 2 | 55281901 | C | G | 0.01 | 0.002 | 4.E-11 |
| **Body fat percentage** | rs76102184 | 1 | 156171486 | C | T | -0.04 | 0.005 | 2.E-13 |
| **Body fat percentage** | rs76115469 | 17 | 429221 | G | A | 0.01 | 0.002 | 7.E-09 |
| **Body fat percentage** | rs76115890 | 13 | 91993627 | T | C | 0.01 | 0.002 | 4.E-09 |
| **Body fat percentage** | rs7630228 | 3 | 71681487 | T | C | 0.01 | 0.002 | 4.E-12 |
| **Body fat percentage** | rs76345589 | 3 | 84185140 | C | G | 0.02 | 0.003 | 1.E-11 |
| **Body fat percentage** | rs7637852 | 3 | 44041777 | A | G | 0.01 | 0.002 | 1.E-13 |
| **Body fat percentage** | rs76638898 | 10 | 21099584 | G | A | 0.03 | 0.005 | 4.E-08 |
| **Body fat percentage** | rs766615746 | 1 | 210336584 | CTAAT | C | -0.01 | 0.002 | 8.E-11 |
| **Body fat percentage** | rs7680610 | 4 | 24118165 | A | G | -0.01 | 0.002 | 2.E-08 |
| **Body fat percentage** | rs7690704 | 4 | 864126 | A | G | 0.01 | 0.002 | 1.E-11 |
| **Body fat percentage** | rs7692075 | 4 | 78808669 | T | G | -0.01 | 0.002 | 1.E-10 |
| **Body fat percentage** | rs7700167 | 4 | 34927607 | T | A | -0.01 | 0.002 | 3.E-12 |
| **Body fat percentage** | rs77043842 | 6 | 111647203 | A | AT | -0.02 | 0.003 | 9.E-11 |
| **Body fat percentage** | rs7707628 | 5 | 153546900 | T | C | 0.01 | 0.002 | 9.E-13 |
| **Body fat percentage** | rs771140684 | 16 | 4925963 | C | CT | -0.01 | 0.002 | 3.E-12 |
| **Body fat percentage** | rs7733087 | 5 | 176164941 | G | A | 0.01 | 0.002 | 4.E-10 |
| **Body fat percentage** | rs77560793 | 1 | 175001179 | G | A | 0.03 | 0.004 | 9.E-12 |
| **Body fat percentage** | rs77737253 | 12 | 3351243 | A | G | 0.02 | 0.003 | 8.E-13 |
| **Body fat percentage** | rs7777351 | 7 | 121962953 | C | A | -0.01 | 0.002 | 3.E-09 |
| **Body fat percentage** | rs779765342 | 1 | 23353906 | CT | C | -0.01 | 0.002 | 3.E-12 |
| **Body fat percentage** | rs7843109 | 8 | 112360702 | C | T | -0.01 | 0.002 | 9.E-09 |
| **Body fat percentage** | rs786420 | 2 | 44719893 | C | T | -0.01 | 0.002 | 7.E-09 |
| **Body fat percentage** | rs7868878 | 9 | 11755491 | G | A | -0.01 | 0.002 | 1.E-10 |
| **Body fat percentage** | rs79100766 | 1 | 151690804 | CA | C | -0.01 | 0.001 | 5.E-10 |
| **Body fat percentage** | rs7925725 | 11 | 131449365 | A | C | -0.01 | 0.002 | 2.E-13 |
| **Body fat percentage** | rs7931311 | 11 | 65286516 | A | C | -0.01 | 0.002 | 4.E-10 |
| **Body fat percentage** | rs7950270 | 11 | 49437043 | C | T | -0.01 | 0.002 | 6.E-10 |
| **Body fat percentage** | rs7962636 | 12 | 116450006 | T | C | 0.01 | 0.002 | 8.E-09 |
| **Body fat percentage** | rs796663884 | 6 | 52426285 | CT | C | 0.01 | 0.002 | 5.E-10 |
| **Body fat percentage** | rs796970069 | 6 | 13177596 | G | GCT | 0.01 | 0.002 | 2.E-10 |
| **Body fat percentage** | rs7973834 | 12 | 118409548 | G | A | 0.01 | 0.002 | 7.E-09 |
| **Body fat percentage** | rs79869125 | 2 | 176422238 | G | T | 0.02 | 0.002 | 9.E-13 |
| **Body fat percentage** | rs8003790 | 14 | 64947181 | C | T | 0.01 | 0.001 | 5.E-09 |
| **Body fat percentage** | rs80082351 | 3 | 114415926 | A | G | 0.02 | 0.003 | 2.E-08 |
| **Body fat percentage** | rs8011566 | 14 | 42939471 | T | A | -0.01 | 0.002 | 4.E-08 |
| **Body fat percentage** | rs801738 | 11 | 65924217 | C | G | 0.01 | 0.002 | 7.E-18 |
| **Body fat percentage** | rs8041325 | 15 | 41732386 | C | T | -0.01 | 0.002 | 4.E-11 |
| **Body fat percentage** | rs8059064 | 16 | 51797169 | G | T | 0.02 | 0.003 | 2.E-10 |
| **Body fat percentage** | rs8071840 | 17 | 70721707 | A | G | -0.01 | 0.001 | 2.E-08 |
| **Body fat percentage** | rs8074454 | 17 | 3981148 | G | C | -0.01 | 0.002 | 3.E-11 |
| **Body fat percentage** | rs8082551 | 17 | 1310661 | C | T | 0.01 | 0.002 | 7.E-11 |
| **Body fat percentage** | rs8103489 | 19 | 3508499 | G | A | -0.01 | 0.002 | 1.E-10 |
| **Body fat percentage** | rs8108198 | 19 | 56146469 | G | A | -0.01 | 0.002 | 7.E-09 |
| **Body fat percentage** | rs811054 | 16 | 72251132 | C | T | -0.01 | 0.001 | 2.E-10 |
| **Body fat percentage** | rs8119351 | 20 | 33754405 | G | A | 0.01 | 0.003 | 3.E-08 |
| **Body fat percentage** | rs812949 | 5 | 170506141 | T | C | -0.01 | 0.002 | 6.E-16 |
| **Body fat percentage** | rs815163 | 1 | 190294726 | T | C | 0.01 | 0.001 | 3.E-09 |
| **Body fat percentage** | rs8176166 | 17 | 41240277 | T | C | 0.01 | 0.002 | 9.E-12 |
| **Body fat percentage** | rs8180470 | 5 | 119388744 | C | T | 0.01 | 0.001 | 1.E-08 |
| **Body fat percentage** | rs825680 | 16 | 73606563 | A | T | 0.01 | 0.002 | 2.E-08 |
| **Body fat percentage** | rs843901 | 20 | 32904636 | T | G | -0.02 | 0.003 | 2.E-09 |
| **Body fat percentage** | rs858672 | 17 | 45269868 | G | A | -0.01 | 0.002 | 6.E-09 |
| **Body fat percentage** | rs879620 | 16 | 4015729 | C | T | -0.01 | 0.002 | 1.E-19 |
| **Body fat percentage** | rs9257761 | 6 | 29319349 | C | A | -0.01 | 0.002 | 6.E-10 |
| **Body fat percentage** | rs9260127 | 6 | 29910478 | G | C | 0.01 | 0.002 | 8.E-12 |
| **Body fat percentage** | rs9289630 | 3 | 141178670 | G | C | -0.01 | 0.002 | 5.E-17 |
| **Body fat percentage** | rs9289970 | 3 | 156301324 | A | C | 0.01 | 0.002 | 2.E-09 |
| **Body fat percentage** | rs9294260 | 6 | 83433228 | G | A | -0.01 | 0.001 | 5.E-09 |
| **Body fat percentage** | rs9319615 | 17 | 79072594 | A | C | 0.01 | 0.001 | 3.E-11 |
| **Body fat percentage** | rs9321191 | 6 | 130165691 | T | C | 0.01 | 0.002 | 4.E-08 |
| **Body fat percentage** | rs9352694 | 6 | 79800818 | A | G | -0.01 | 0.002 | 1.E-08 |
| **Body fat percentage** | rs9371992 | 6 | 156685642 | A | G | -0.01 | 0.002 | 2.E-08 |
| **Body fat percentage** | rs9435341 | 1 | 107616641 | T | C | 0.01 | 0.002 | 2.E-11 |
| **Body fat percentage** | rs9449999 | 6 | 85371011 | A | G | 0.01 | 0.002 | 1.E-08 |
| **Body fat percentage** | rs9471333 | 6 | 40362023 | C | T | 0.01 | 0.001 | 7.E-22 |
| **Body fat percentage** | rs9512696 | 13 | 28012527 | A | G | -0.01 | 0.002 | 1.E-13 |
| **Body fat percentage** | rs9527958 | 13 | 59841918 | A | G | -0.01 | 0.002 | 9.E-09 |
| **Body fat percentage** | rs9673839 | 16 | 76895693 | A | G | -0.01 | 0.001 | 5.E-08 |
| **Body fat percentage** | rs9706403 | 12 | 27271251 | A | G | 0.01 | 0.002 | 1.E-08 |
| **Body fat percentage** | rs9770544 | 7 | 27232126 | C | G | 0.02 | 0.002 | 8.E-15 |
| **Body fat percentage** | rs9788550 | 14 | 29681138 | G | C | 0.02 | 0.002 | 9.E-20 |
| **Body fat percentage** | rs9843653 | 3 | 49920571 | T | C | -0.02 | 0.001 | 4.E-26 |
| **Body fat percentage** | rs9847672 | 3 | 131618541 | C | T | -0.01 | 0.002 | 3.E-18 |
| **Body fat percentage** | rs9859077 | 3 | 101136402 | G | C | 0.01 | 0.002 | 4.E-11 |
| **Body fat percentage** | rs9888067 | 10 | 96648224 | T | G | -0.01 | 0.002 | 6.E-12 |
| **Body fat percentage** | rs9968060 | 3 | 62471282 | C | T | -0.01 | 0.002 | 8.E-13 |
| **Body fat percentage** | rs9975329 | 21 | 45418604 | A | T | 0.01 | 0.002 | 2.E-08 |
| **Body fat percentage - FA** | rs10876529 | 12 | 54421810 | T | C | -0.01 | 0.002 | 5.E-12 |
| **Body fat percentage - FA** | rs11045172 | 12 | 20470221 | A | C | -0.01 | 0.002 | 5.E-09 |
| **Body fat percentage - FA** | rs11135038 | 5 | 157930133 | T | G | -0.01 | 0.002 | 3.E-16 |
| **Body fat percentage - FA** | rs113222038 | 11 | 62380027 | C | T | 0.01 | 0.002 | 3.E-12 |
| **Body fat percentage - FA** | rs11664106 | 18 | 2846812 | A | T | -0.01 | 0.002 | 5.E-09 |
| **Body fat percentage - FA** | rs12130231 | 1 | 219631304 | A | G | 0.02 | 0.002 | 4.E-41 |
| **Body fat percentage - FA** | rs12369179 | 12 | 122963550 | C | T | 0.03 | 0.003 | 2.E-29 |
| **Body fat percentage - FA** | rs12441543 | 15 | 31689543 | G | A | -0.01 | 0.002 | 1.E-12 |
| **Body fat percentage - FA** | rs12681990 | 8 | 36859186 | T | C | 0.01 | 0.002 | 5.E-09 |
| **Body fat percentage - FA** | rs12940684 | 17 | 7453919 | C | T | 0.01 | 0.002 | 1.E-11 |
| **Body fat percentage - FA** | rs13132853 | 4 | 38680015 | A | G | 0.01 | 0.002 | 1.E-08 |
| **Body fat percentage - FA** | rs13389219 | 2 | 165528876 | C | T | -0.02 | 0.002 | 2.E-29 |
| **Body fat percentage - FA** | rs142186653 | 17 | 73879851 | A | C | -0.01 | 0.002 | 1.E-11 |
| **Body fat percentage - FA** | rs2802774 | 1 | 203527812 | C | A | -0.01 | 0.002 | 4.E-13 |
| **Body fat percentage - FA** | rs2943653 | 2 | 227047771 | C | T | 0.02 | 0.002 | 1.E-23 |
| **Body fat percentage - FA** | rs2980888 | 8 | 126507308 | T | C | -0.01 | 0.002 | 4.E-14 |
| **Body fat percentage - FA** | rs30351 | 5 | 55794632 | G | A | 0.01 | 0.002 | 2.E-12 |
| **Body fat percentage - FA** | rs4450871 | 4 | 4990298 | A | G | -0.01 | 0.001 | 1.E-08 |
| **Body fat percentage - FA** | rs4684847 | 3 | 12386337 | C | T | -0.03 | 0.002 | 3.E-37 |
| **Body fat percentage - FA** | rs4821764 | 22 | 38599364 | G | A | 0.02 | 0.002 | 3.E-29 |
| **Body fat percentage - FA** | rs4976033 | 5 | 67714246 | A | G | 0.01 | 0.002 | 5.E-14 |
| **Body fat percentage - FA** | rs555162510 | 19 | 46183031 | A | AT | 0.02 | 0.002 | 5.E-34 |
| **Body fat percentage - FA** | rs573454216 | 6 | 139837429 | G | A | -0.01 | 0.002 | 1.E-08 |
| **Body fat percentage - FA** | rs6029180 | 20 | 39178923 | A | G | -0.01 | 0.002 | 2.E-08 |
| **Body fat percentage - FA** | rs62271373 | 3 | 150066540 | T | A | 0.02 | 0.003 | 2.E-14 |
| **Body fat percentage - FA** | rs6977416 | 7 | 150542711 | G | A | 0.01 | 0.002 | 3.E-14 |
| **Body fat percentage - FA** | rs7133378 | 12 | 124409502 | G | A | -0.02 | 0.002 | 4.E-33 |
| **Body fat percentage - FA** | rs7233512 | 18 | 42595076 | G | A | 0.01 | 0.002 | 8.E-11 |
| **Body fat percentage - FA** | rs7258937 | 19 | 33938800 | C | T | -0.02 | 0.001 | 2.E-26 |
| **Body fat percentage - FA** | rs72697297 | 14 | 93069989 | T | C | 0.02 | 0.002 | 8.E-15 |
| **Body fat percentage - FA** | rs72959041 | 6 | 127454893 | G | A | 0.02 | 0.003 | 2.E-12 |
| **Body fat percentage - FA** | rs972283 | 7 | 130466854 | A | G | 0.01 | 0.001 | 2.E-22 |
| **Body fat percentage - FA** | rs9764678 | 5 | 118726662 | T | C | -0.01 | 0.002 | 3.E-10 |
| **Body fat percentage - FA** | rs9851766 | 3 | 138121509 | A | G | 0.01 | 0.002 | 1.E-11 |
| **Body fat percentage - FA** | rs987469 | 4 | 89706643 | C | G | 0.01 | 0.001 | 6.E-17 |
| **Body fat percentage - FA** | rs998584 | 6 | 43757896 | C | A | 0.01 | 0.001 | 1.E-09 |
| **Body fat percentage - UFA** | 14:79940130_TAGGAGTTTTTCCAGATCATTAGCCACTTATACGGAG_T | 14 | 79940130 | TAGGAGTTTTTCCAGATCATTAGCCACTTATACGGAG | T | -0.02 | 0.002 | 7.E-30 |
| **Body fat percentage - UFA** | 15:73322940_AT_A | 15 | 73322940 | AT | A | -0.01 | 0.002 | 3.E-21 |
| **Body fat percentage - UFA** | 1:113202203_TCTCTC_T | 1 | 113202203 | TCTCTC | T | 0.01 | 0.002 | 3.E-14 |
| **Body fat percentage - UFA** | 1:72767554_CA_C | 1 | 72767554 | CA | C | 0.02 | 0.002 | 2.E-25 |
| **Body fat percentage - UFA** | 5:87969925_CGG_C | 5 | 87969925 | CGG | C | -0.02 | 0.002 | 3.E-30 |
| **Body fat percentage - UFA** | 6:34650934_CGT_C | 6 | 34650934 | CGT | C | -0.03 | 0.002 | 2.E-33 |
| **Body fat percentage - UFA** | rs10623997 | 5 | 107478679 | T | TATAATA | 0.01 | 0.002 | 2.E-16 |
| **Body fat percentage - UFA** | rs10756713 | 9 | 15880555 | A | G | 0.02 | 0.002 | 1.E-37 |
| **Body fat percentage - UFA** | rs10938397 | 4 | 45182527 | A | G | -0.02 | 0.001 | 7.E-41 |
| **Body fat percentage - UFA** | rs11122450 | 1 | 230301811 | T | G | 0.01 | 0.002 | 6.E-11 |
| **Body fat percentage - UFA** | rs11642015 | 16 | 53802494 | C | T | -0.04 | 0.002 | 7.E-165 |
| **Body fat percentage - UFA** | rs11666808 | 19 | 18383506 | T | C | 0.02 | 0.002 | 6.E-27 |
| **Body fat percentage - UFA** | rs13107325 | 4 | 103188709 | C | T | -0.03 | 0.003 | 3.E-28 |
| **Body fat percentage - UFA** | rs143684747 | 2 | 633053 | A | AC | -0.03 | 0.002 | 3.E-49 |
| **Body fat percentage - UFA** | rs1471740 | 3 | 136328270 | T | C | -0.01 | 0.002 | 4.E-09 |
| **Body fat percentage - UFA** | rs17764730 | 5 | 127357526 | C | T | 0.01 | 0.002 | 8.E-12 |
| **Body fat percentage - UFA** | rs2112347 | 5 | 75015242 | T | G | 0.02 | 0.002 | 2.E-31 |
| **Body fat percentage - UFA** | rs2274224 | 10 | 96039597 | G | C | 0.02 | 0.001 | 7.E-29 |
| **Body fat percentage - UFA** | rs236660 | 7 | 75050086 | T | C | -0.01 | 0.002 | 2.E-19 |
| **Body fat percentage - UFA** | rs3764002 | 12 | 108618630 | C | T | 0.02 | 0.002 | 2.E-29 |
| **Body fat percentage - UFA** | rs4755725 | 11 | 43637975 | C | A | 0.02 | 0.002 | 3.E-26 |
| **Body fat percentage - UFA** | rs4776985 | 15 | 68123021 | T | G | 0.02 | 0.002 | 9.E-31 |
| **Body fat percentage - UFA** | rs4790292 | 17 | 1824305 | C | A | 0.02 | 0.002 | 6.E-24 |
| **Body fat percentage - UFA** | rs4876611 | 8 | 116671848 | A | G | -0.02 | 0.002 | 7.E-28 |
| **Body fat percentage - UFA** | rs539515 | 1 | 177889025 | A | C | -0.03 | 0.002 | 9.E-54 |
| **Body fat percentage - UFA** | rs55931203 | 17 | 65854602 | C | T | -0.02 | 0.002 | 6.E-28 |
| **Body fat percentage - UFA** | rs56186137 | 16 | 28825953 | A | G | -0.02 | 0.002 | 3.E-50 |
| **Body fat percentage - UFA** | rs61888762 | 11 | 27709630 | C | G | -0.02 | 0.002 | 3.E-29 |
| **Body fat percentage - UFA** | rs6567160 | 18 | 57829135 | T | C | -0.03 | 0.002 | 5.E-48 |
| **Body fat percentage - UFA** | rs6602997 | 15 | 84521398 | C | T | -0.02 | 0.002 | 6.E-43 |
| **Body fat percentage - UFA** | rs6752378 | 2 | 25150116 | C | A | -0.02 | 0.001 | 7.E-55 |
| **Body fat percentage - UFA** | rs7124681 | 11 | 47529947 | C | A | -0.02 | 0.002 | 7.E-50 |
| **Body fat percentage - UFA** | rs7132908 | 12 | 50263148 | G | A | -0.02 | 0.002 | 1.E-36 |
| **Body fat percentage - UFA** | rs71658797 | 1 | 77967507 | T | A | -0.02 | 0.002 | 6.E-26 |
| **Body fat percentage - UFA** | rs72892910 | 6 | 50816887 | G | T | -0.02 | 0.002 | 2.E-32 |
| **Body fat percentage - UFA** | rs771025058 | 18 | 21122207 | AAG | A | 0.02 | 0.001 | 4.E-30 |
| **Body fat percentage - UFA** | rs8049669 | 16 | 69551467 | A | T | 0.02 | 0.002 | 7.E-26 |
| **Body fat percentage - UFA** | rs9358912 | 6 | 26211146 | G | T | 0.02 | 0.002 | 5.E-39 |

***Supplementary File 1e.*** *The inverse-variance weighted two-sample MR analysis/meta-analysis of 37 identified diseases from published GWAS and/or FinnGen for body mass index (BMI), body fat percentage, “favourable adiposity” (FA) and “unfavourable adiposity” (UFA) clusters. Italicised results are those that were interpreted – including all BMI, body fat percentage if a causal effect of BMI was indicated, and FA/UFA if a causal effect of BMI and body fat percentage was indicated. OR: odds ratio, 95% CI: 95% confidence interval; P: p-value.*

|  | | | **BMI** | | | **Body fat percentage** | | | **FA** | | | **UFA** | | |
| --- | --- | --- | --- | --- | --- | --- | --- | --- | --- | --- | --- | --- | --- | --- |
| **System** | **Disease** | **Study** | **OR** | **95% CI** | **P** | **OR** | **95% CI** | **P** | **OR** | **95% CI** | **P** | **OR** | **95% CI** | **P** |
| **Cardiovascular and metabolic** | **Abdominal aneurysm** | **Meta-analysis** | *1.16* | *(0.83, 1.62)* | *0.394* | 1.16 | (0.91, 1.48) | 0.233 | 0.81 | (0.43, 1.51) | 0.506 | 1.26 | (0.71, 2.26) | 0.430 |
| **Cardiovascular and metabolic** | **Atrial fibrillation** | **Meta-analysis** | *1.65* | *(1.33, 2.05)* | *5.E-06* | *1.53* | *(1.21, 1.92)* | *3.E-04* | *0.79* | *(0.39, 1.61)* | *0.518* | *1.78* | *(1.50, 2.12)* | *6.E-11* |
| **Cardiovascular and metabolic** | **Coronary artery disease** | **Meta-analysis** | *1.41* | *(1.10, 1.81)* | *0.007* | *1.39* | *(1.16, 1.68)* | *5.E-04* | *0.34* | *(0.25, 0.47)* | *2.E-11* | *1.66* | *(1.08, 2.54)* | *0.021* |
| **Cardiovascular and metabolic** | **Deep vein thrombosis** | **FinnGen** | *1.68* | *(1.20, 2.37)* | *0.004* | *1.71* | *(1.40, 2.09)* | *2.E-07* | *3.45* | *(1.81, 6.57)* | *6.E-04* | *2.05* | *(1.15, 3.64)* | *0.020* |
| **Cardiovascular and metabolic** | **Heart failure** | **Meta-analysis** | *1.86* | *(1.60, 2.16)* | *2.E-16* | *1.76* | *(1.65, 1.88)* | *2.E-67* | *0.85* | *(0.65, 1.11)* | *0.238* | *2.29* | *(1.85, 2.83)* | *2.E-14* |
| **Cardiovascular and metabolic** | **Hypertension** | **FinnGen** | *2.18* | *(1.80, 2.64)* | *2.E-11* | *2.01* | *(1.79, 2.26)* | *6.E-29* | *0.34* | *(0.21, 0.55)* | *1.E-04* | *3.03* | *(2.18, 4.22)* | *2.E-07* |
| **Cardiovascular and metabolic** | **Peripheral artery disease** | **FinnGen** | *1.87* | *(1.46, 2.39)* | *4.E-06* | *1.80* | *(1.52, 2.14)* | *4.E-11* | *0.20* | *(0.11, 0.38)* | *2.E-05* | *3.31* | *(2.09, 5.24)* | *1.E-05* |
| **Cardiovascular and metabolic** | **Pulmonary embolism** | **FinnGen** | *1.23* | *(0.85, 1.78)* | *0.269* | 1.26 | (1.02, 1.55) | 0.032 | 2.40 | (0.99, 5.84) | 0.061 | 1.66 | (0.94, 2.94) | 0.090 |
| **Cardiovascular and metabolic** | **Stroke** | **Meta-analysis** | *1.19* | *(1.07, 1.31)* | *1.E-03* | *1.23* | *(1.15, 1.31)* | *7.E-10* | *0.65* | *(0.52, 0.83)* | *4.E-04* | *1.43* | *(1.23, 1.67)* | *3.E-06* |
| **Cardiovascular and metabolic** | **Venous thromboembolism** | **Meta-analysis** | *1.45* | *(1.27, 1.67)* | *8.E-08* | *1.57* | *(1.40, 1.77)* | *4.E-14* | *2.52* | *(1.82, 3.47)* | *2.E-08* | *1.63* | *(1.25, 2.13)* | *3.E-04* |
| **Cardiovascular and metabolic** | **Polycystic ovary syndrome** | **Meta-analysis** | *4.11* | *(2.97, 5.70)* | *2.E-17* | *2.64* | *(2.11, 3.29)* | *1.E-17* | *0.51* | *(0.21, 1.23)* | *0.132* | *7.13* | *(3.66, 13.90)* | *8.E-09* |
| **Cardiovascular and metabolic** | **Type 2 diabetes** | **Meta-analysis** | *2.97* | *(2.60, 3.39)* | *3.E-57* | *2.38* | *(2.12, 2.68)* | *5.E-49* | *0.11* | *(0.08, 0.16)* | *4.E-33* | *5.50* | *(4.29, 7.05)* | *4.E-41* |
| **Cardiovascular and metabolic** | **Chronic kidney disease** | **Meta-analysis** | *1.21* | *(1.08, 1.36)* | *0.002* | *1.25* | *(0.98, 1.59)* | *0.071* | 0.64 | (0.48, 0.84) | 0.002 | 1.19 | (0.97, 1.45) | 0.092 |
| **Musculoskeletal** | **Gout** | **Meta-analysis** | *1.68* | *(1.42, 2.00)* | *3.E-09* | *1.66* | *(1.45, 1.89)* | *3.E-14* | *0.44* | *(0.29, 0.68)* | *2.E-04* | *2.49* | *(1.88, 3.29)* | *2.E-10* |
| **Musculoskeletal** | **Osteoarthritis** | **Meta-analysis** | *1.72* | *(1.37, 2.16)* | *2.E-06* | *1.82* | *(1.73, 1.91)* | *1.E-124* | *1.45* | *(1.19, 1.76)* | *2.E-04* | *2.20* | *(1.64, 2.95)* | *1.E-07* |
| **Musculoskeletal** | **Osteoporosis** | **Meta-analysis** | *1.04* | *(0.77, 1.40)* | *0.808* | 1.13 | (1.03, 1.25) | 0.010 | 1.13 | (0.77, 1.64) | 0.530 | 1.18 | (1.01, 1.37) | 0.034 |
| **Musculoskeletal** | **Rheumatoid arthritis** | **Meta-analysis** | *1.23* | *(1.04, 1.46)* | *0.017* | *1.49* | *(1.30, 1.70)* | *5.E-09* | *1.95* | *(1.24, 3.08)* | *0.004* | *1.61* | *(1.14, 2.27)* | *0.007* |
| **Gastrointestinal** | **Gallstones** | **FinnGen** | *1.87* | *(1.58, 2.23)* | *7.E-10* | *1.99* | *(1.78, 2.22)* | *1.E-30* | *1.37* | *(0.86, 2.19)* | *0.200* | *2.55* | *(1.88, 3.45)* | *9.E-07* |
| **Gastrointestinal** | **Gastro-oesophageal reflux disease** | **Meta-analysis** | *1.15* | *(1.05, 1.25)* | *0.002* | *1.48* | *(1.00, 2.18)* | *0.051* | *1.16* | *(0.96, 1.41)* | *0.119* | *1.26* | *(0.99, 1.61)* | *0.062* |
| **Nervous** | **Alzheimer's disease** | **Meta-analysis** | *0.98* | *(0.85, 1.13)* | *0.791* | 0.99 | (0.96, 1.03) | 0.697 | 1.00 | (0.92, 1.08) | 0.939 | 1.02 | (0.98, 1.07) | 0.335 |
| **Nervous** | **Depression** | **Meta-analysis** | *1.12* | *(0.98, 1.27)* | *0.092* | 1.19 | (1.11, 1.28) | 1.E-06 | 1.20 | (0.98, 1.48) | 0.074 | 1.02 | (0.81, 1.30) | 0.855 |
| **Nervous** | **Multiple sclerosis** | **FinnGen** | *0.81* | *(0.47, 1.40)* | *0.443* | 0.85 | (0.58, 1.25) | 0.407 | 1.53 | (0.38, 6.20) | 0.559 | 0.62 | (0.24, 1.59) | 0.332 |
| **Nervous** | **Parkinson's disease** | **Meta-analysis** | *0.96* | *(0.77, 1.19)* | *0.704* | 0.88 | (0.63, 1.24) | 0.472 | 1.35 | (0.87, 2.08) | 0.176 | 0.83 | (0.44, 1.55) | 0.551 |
| **Integumentary** | **Psoriasis** | **Meta-analysis** | *1.62* | *(1.20, 2.19)* | *0.001* | *1.78* | *(1.54, 2.05)* | *3.E-15* | *1.20* | *(0.70, 2.06)* | *0.514* | *2.11* | *(1.49, 2.99)* | *3.E-05* |
| **Respiratory** | **Adult-onset asthma** | **Meta-analysis** | *1.25* | *(1.03, 1.52)* | *0.022* | *1.43* | *(1.25, 1.63)* | *3.E-07* | *1.14* | *(0.88, 1.49)* | *0.319* | *1.34* | *(0.97, 1.87)* | *0.080* |
| **Cancer** | **Barrett's oesophagus** | **FinnGen** | *1.29* | *(0.55, 3.02)* | *0.558* | 1.42 | (0.79, 2.54) | 0.242 | 3.16 | (0.39, 25.47) | 0.288 | 0.62 | (0.14, 2.65) | 0.522 |
| **Cancer** | **Breast cancer** | **Meta-analysis** | *0.60* | *(0.51, 0.70)* | *4.E-10* | *0.84* | *(0.71, 0.98)* | *0.032* | *1.12* | *(0.64, 1.94)* | *0.689* | *0.52* | *(0.39, 0.68)* | *4.E-06* |
| **Cancer** | **Cancer myeloma** | **FinnGen** | *1.60* | *(0.76, 3.40)* | *0.221* | 1.43 | (0.85, 2.39) | 0.179 | 1.96 | (0.25, 15.59) | 0.529 | 1.33 | (0.30, 5.99) | 0.710 |
| **Cancer** | **Colorectal cancer** | **Meta-analysis** | *1.09* | *(0.89, 1.35)* | *0.408* | 0.96 | (0.77, 1.19) | 0.697 | 0.67 | (0.52, 0.85) | 0.001 | 1.20 | (0.97, 1.50) | 0.094 |
| **Cancer** | **Endometrial cancer** | **Meta-analysis** | *1.82* | *(1.26, 2.63)* | *0.002* | *1.36* | *(0.86, 2.16)* | *0.190* | 0.93 | (0.53, 1.63) | 0.802 | 1.48 | (0.44, 4.98) | 0.527 |
| **Cancer** | **Lung cancer** | **FinnGen** | *1.21* | *(0.75, 1.96)* | *0.444* | 1.33 | (1.00, 1.77) | 0.051 | 0.64 | (0.23, 1.78) | 0.401 | 1.36 | (0.65, 2.84) | 0.426 |
| **Cancer** | **Meningioma** | **FinnGen** | *0.62* | *(0.36, 1.06)* | *0.085* | 1.01 | (0.71, 1.43) | 0.967 | 1.06 | (0.31, 3.61) | 0.928 | 0.36 | (0.15, 0.86) | 0.028 |
| **Cancer** | **Ovarian cancer** | **Meta-analysis** | *1.15* | *(0.72, 1.85)* | *0.562* | 1.16 | (0.69, 1.93) | 0.579 | 0.35 | (0.18, 0.70) | 0.003 | 1.26 | (0.80, 1.99) | 0.315 |
| **Cancer** | **Pancreatic cancer** | **FinnGen** | *1.39* | *(0.67, 2.88)* | *0.376* | 1.25 | (0.79, 1.97) | 0.345 | 3.30 | (0.50, 21.83) | 0.224 | 1.45 | (0.45, 4.61) | 0.536 |
| **Cancer** | **Prostate cancer** | **Meta-analysis** | *0.91* | *(0.81, 1.03)* | *0.135* | 0.90 | (0.83, 0.97) | 0.006 | 0.97 | (0.79, 1.19) | 0.772 | 0.94 | (0.77, 1.15) | 0.565 |
| **Cancer** | **Renal cancer** | **Meta-analysis** | *1.47* | *(1.12, 1.92)* | *0.005* | *1.23* | *(0.94, 1.60)* | *0.126* | 0.94 | (0.33, 2.72) | 0.910 | 1.72 | (1.18, 2.51) | 0.005 |
| **Cancer** | **Thyroid cancer** | **FinnGen** | *0.90* | *(0.52, 1.56)* | *0.719* | 0.73 | (0.51, 1.06) | 0.103 | 0.91 | (0.24, 3.40) | 0.888 | 1.09 | (0.42, 2.86) | 0.859 |

**Supplementary File 1f. Heterogeneity statistics from random-effects meta-analysis of inverse-variance weighted Mendelian randomisation of published GWAS and FinnGen studies. BMI: body mass index; FA: “favourable adiposity”; UFA: “unfavourable adiposity”, Q: Q-statistic; P: p-value; I^2^: I^2^-statistic.**

|  | | **BMI** | | | **Body fat percentage** | | | **FA** | | | **UFA** | | |
| --- | --- | --- | --- | --- | --- | --- | --- | --- | --- | --- | --- | --- | --- |
| **System** | **Disease** | **Q** | **P** | **I^2^** | **Q** | **P** | **I^2^** | **Q** | **P** | **I^2^** | **Q** | **P** | **I^2^** |
| **Cardiovascular**  **and metabolic** | **Abdominal aneurysm** | 2.73 | 0.098 | 63.43 | 3.56 | 0.059 | 71.93 | 1.88 | 0.170 | 46.79 | 2.56 | 0.109 | 60.96 |
| **Cardiovascular**  **and metabolic** | **Atrial fibrillation** | 3.86 | 0.049 | 74.09 | 9.24 | 0.002 | 89.18 | 5.03 | 0.025 | 80.10 | 0.79 | 0.375 | 0 |
| **Cardiovascular**  **and metabolic** | **Coronary artery disease** | 5.10 | 0.024 | 80.38 | 7.16 | 0.007 | 86.03 | 0.00 | 0.982 | 0 | 5.27 | 0.022 | 81.01 |
| **Cardiovascular**  **and metabolic** | **Heart failure** | 1.69 | 0.194 | 40.71 | 0.00 | 0.996 | 0 | 0.42 | 0.518 | 0 | 0.68 | 0.408 | 0 |
| **Cardiovascular**  **and metabolic** | **Ischemic stroke** | 0.00 | 0.956 | 0 | 0.04 | 0.844 | 0 | 0.05 | 0.828 | 0 | 0.26 | 0.610 | 0 |
| **Cardiovascular**  **and metabolic** | **Stroke** | 0.47 | 0.492 | 0 | 0.04 | 0.836 | 0 | 0.80 | 0.372 | 0 | 0.01 | 0.929 | 0 |
| **Cardiovascular**  **and metabolic** | **Venous thromboembolism** | 0.02 | 0.884 | 0 | 1.23 | 0.268 | 18.65 | 0.38 | 0.535 | 0 | 0.05 | 0.827 | 0 |
| **Cardiovascular**  **and metabolic** | **Polycystic ovary syndrome** | 0.14 | 0.705 | 0 | 0.00 | 0.962 | 0 | 0.23 | 0.630 | 0 | 0.44 | 0.507 | 0 |
| **Cardiovascular**  **and metabolic** | **Type 2 diabetes** | 0.09 | 0.759 | 0 | 1.20 | 0.274 | 16.41 | 0.01 | 0.909 | 0 | 0.03 | 0.862 | 0 |
| **Cardiovascular**  **and metabolic** | **Chronic kidney disease** | 0.48 | 0.488 | 0 | 4.34 | 0.037 | 76.95 | 0.83 | 0.362 | 0 | 0.92 | 0.337 | 0 |
| **Musculoskeletal** | **Gout** | 0.00 | 0.978 | 0 | 1.22 | 0.269 | 18.17 | 0.00 | 0.956 | 0 | 0.35 | 0.554 | 0 |
| **Musculoskeletal** | **Osteoarthritis** | 6.14 | 0.013 | 83.72 | 0.03 | 0.853 | 0 | 0.00 | 0.949 | 0 | 2.66 | 0.103 | 62.35 |
| **Musculoskeletal** | **Osteoporosis** | 3.66 | 0.056 | 72.69 | 1.37 | 0.241 | 27.16 | 1.37 | 0.242 | 26.86 | 0.60 | 0.440 | 0 |
| **Musculoskeletal** | **Rheumatoid arthritis** | 0.11 | 0.736 | 0 | 0.64 | 0.423 | 0 | 0.05 | 0.819 | 0 | 0.46 | 0.497 | 0 |
| **Gastrointestinal** | **Gastro-oesophageal reflux disease** | 0.48 | 0.487 | 0 | 33.54 | 7.E-09 | 97.02 | 0.12 | 0.726 | 0 | 1.52 | 0.218 | 34.13 |
| **Nervous** | **Alzheimer's disease** | 0.01 | 0.923 | 0 | 0.02 | 0.884 | 0 | 0.49 | 0.482 | 0 | 0.78 | 0.379 | 0 |
| **Nervous** | **Depression** | 1.77 | 0.183 | 43.65 | 1.21 | 0.271 | 17.43 | 0.00 | 0.985 | 0 | 1.49 | 0.223 | 32.69 |
| **Nervous** | **Parkinson's disease** | 0.11 | 0.743 | 0 | 4.35 | 0.037 | 77.04 | 0.41 | 0.521 | 0 | 2.38 | 0.123 | 57.93 |
| **Integumentary** | **Psoriasis** | 2.33 | 0.127 | 57.16 | 0.26 | 0.610 | 0 | 0.61 | 0.434 | 0 | 0.23 | 0.631 | 0 |
| **Respiratory** | **Adult-onset asthma** | 3.89 | 0.049 | 74.29 | 4.09 | 0.043 | 75.57 | 0.35 | 0.551 | 0 | 3.13 | 0.077 | 68.03 |
| **Respiratory** | **Child-onset asthma** | 3.63 | 0.057 | 72.43 | 31.14 | 2.E-08 | 96.79 | 4.01 | 0.045 | 75.07 | 5.76 | 0.016 | 82.65 |
| **Cancer** | **Breast cancer** | 0.42 | 0.519 | 0 | 2.13 | 0.145 | 52.97 | 1.12 | 0.290 | 10.50 | 0.28 | 0.598 | 0 |
| **Cancer** | **Colorectal cancer** | 0.03 | 0.857 | 0 | 1.70 | 0.192 | 41.31 | 1.66 | 0.436 | 12.58 | 2.77 | 0.251 | 33.47 |
| **Cancer** | **Endometrial cancer** | 1.71 | 0.192 | 41.36 | 5.87 | 0.015 | 82.96 | 0.04 | 0.835 | 0 | 4.38 | 0.036 | 77.14 |
| **Cancer** | **Ovarian cancer** | 1.90 | 0.168 | 47.34 | 4.58 | 0.032 | 78.19 | 0.00 | 0.983 | 0 | 0.04 | 0.832 | 0 |
| **Cancer** | **Prostate cancer** | 0.00 | 0.991 | 0 | 0.00 | 0.973 | 0 | 0.04 | 0.847 | 0 | 0.85 | 0.357 | 0 |
| **Cancer** | **Renal cancer** | 0.02 | 0.879 | 0 | 0.26 | 0.612 | 0 | 2.23 | 0.136 | 55.07 | 0.19 | 0.664 | 0 |

**Supplementary File 1gi. The inverse-variance weighted, weighted median, Egger and penalised weighted median MR analyses for body mass index using FinnGen and published GWAS. OR: odds ratio; LCI: lower 95% confidence interval; UCI: upper 95% confidence interval; P: p-value; Intercept P: intercept p-value; Q: Q-statistic; I^2^ MR-Egger: I^2^-statistic MR-Egger.**

|  | | | | | | | | | | **Heterogeneity** | | |
| --- | --- | --- | --- | --- | --- | --- | --- | --- | --- | --- | --- | --- |
| **System** | **Disease** | **Study** | **Analysis** | **OR** | **LCI** | **UCI** | **P** | **Egger intercept** | **Intercept P** | **Q** | **P** | **I^2^ MR-Egger** |
| **Cardiovascular and metabolic** | **Abdominal aortic aneurysm** | **FinnGen** | **Inverse-variance weighted** | 1.48 | 0.97 | 2.26 | 0.076 |  |  | 97.54 | 0.024 |  |
| **Cardiovascular and metabolic** | **Abdominal aortic aneurysm** | **FinnGen** | **Weighted median** | 1.28 | 0.70 | 2.33 | 0.416 |  |  |  |  |  |
| **Cardiovascular and metabolic** | **Abdominal aortic aneurysm** | **FinnGen** | **MR-Egger** | 0.81 | 0.33 | 1.95 | 0.635 | 0.02 | 0.130 | 94.41 | 0.033 | 0.96 |
| **Cardiovascular and metabolic** | **Abdominal aortic aneurysm** | **FinnGen** | **Penalised weighted median** | 1.26 | 0.70 | 2.29 | 0.440 |  |  |  |  |  |
| **Cardiovascular and metabolic** | **Abdominal aortic aneurysm** | **Published GWAS** | **Inverse-variance weighted** | 1.03 | 0.95 | 1.11 | 0.528 |  |  | 82.51 | 0.186 |  |
| **Cardiovascular and metabolic** | **Abdominal aortic aneurysm** | **Published GWAS** | **Weighted median** | 1.00 | 0.89 | 1.13 | 0.956 |  |  |  |  |  |
| **Cardiovascular and metabolic** | **Abdominal aortic aneurysm** | **Published GWAS** | **MR-Egger** | 1.05 | 0.89 | 1.24 | 0.568 | 0.00 | 0.759 | 82.40 | 0.167 | 0.96 |
| **Cardiovascular and metabolic** | **Abdominal aortic aneurysm** | **Published GWAS** | **Penalised weighted median** | 1.05 | 0.90 | 1.23 | 0.508 |  |  |  |  |  |
| **Cardiovascular and metabolic** | **Atrial fibrillation** | **FinnGen** | **Inverse-variance weighted** | 1.88 | 1.54 | 2.30 | 4.E-08 |  |  | 94.87 | 0.037 |  |
| **Cardiovascular and metabolic** | **Atrial fibrillation** | **FinnGen** | **Weighted median** | 1.99 | 1.48 | 2.68 | 5.E-06 |  |  |  |  |  |
| **Cardiovascular and metabolic** | **Atrial fibrillation** | **FinnGen** | **MR-Egger** | 1.35 | 0.90 | 2.05 | 0.156 | 0.01 | 0.080 | 90.84 | 0.056 | 0.96 |
| **Cardiovascular and metabolic** | **Atrial fibrillation** | **FinnGen** | **Penalised weighted median** | 1.99 | 1.45 | 2.72 | 2.E-05 |  |  |  |  |  |
| **Cardiovascular and metabolic** | **Atrial fibrillation** | **Published GWAS** | **Inverse-variance weighted** | 1.50 | 1.37 | 1.66 | 3.E-12 |  |  | 110.12 | 0.003 |  |
| **Cardiovascular and metabolic** | **Atrial fibrillation** | **Published GWAS** | **Weighted median** | 1.45 | 1.28 | 1.66 | 2.E-08 |  |  |  |  |  |
| **Cardiovascular and metabolic** | **Atrial fibrillation** | **Published GWAS** | **MR-Egger** | 1.29 | 1.06 | 1.58 | 0.014 | 0.00 | 0.095 | 105.85 | 0.005 | 0.96 |
| **Cardiovascular and metabolic** | **Atrial fibrillation** | **Published GWAS** | **Penalised weighted median** | 1.39 | 1.20 | 1.60 | 6.E-06 |  |  |  |  |  |
| **Cardiovascular and metabolic** | **Cardioembolic stroke** | **Published GWAS** | **Inverse-variance weighted** | 1.22 | 0.99 | 1.50 | 0.067 |  |  | 66.55 | 0.659 |  |
| **Cardiovascular and metabolic** | **Cardioembolic stroke** | **Published GWAS** | **Weighted median** | 1.15 | 0.81 | 1.65 | 0.434 |  |  |  |  |  |
| **Cardiovascular and metabolic** | **Cardioembolic stroke** | **Published GWAS** | **MR-Egger** | 1.19 | 0.76 | 1.84 | 0.450 | 0.00 | 0.886 | 66.53 | 0.628 | 0.96 |
| **Cardiovascular and metabolic** | **Cardioembolic stroke** | **Published GWAS** | **Penalised weighted median** | 1.17 | 0.82 | 1.66 | 0.397 |  |  |  |  |  |
| **Cardiovascular and metabolic** | **Cardiovascular disease** | **FinnGen** | **Inverse-variance weighted** | 1.64 | 1.46 | 1.84 | 6.E-12 |  |  | 114.66 | 0.001 |  |
| **Cardiovascular and metabolic** | **Cardiovascular disease** | **FinnGen** | **Weighted median** | 1.72 | 1.48 | 1.98 | 3.E-13 |  |  |  |  |  |
| **Cardiovascular and metabolic** | **Cardiovascular disease** | **FinnGen** | **MR-Egger** | 1.55 | 1.21 | 1.98 | 1.E-03 | 0.00 | 0.597 | 114.21 | 9.E-04 | 0.96 |
| **Cardiovascular and metabolic** | **Cardiovascular disease** | **FinnGen** | **Penalised weighted median** | 1.71 | 1.47 | 1.99 | 6.E-12 |  |  |  |  |  |
| **Cardiovascular and metabolic** | **Coronary artery disease** | **Published GWAS** | **Inverse-variance weighted** | 1.60 | 1.37 | 1.87 | 1.E-07 |  |  | 168.71 | 1.E-09 |  |
| **Cardiovascular and metabolic** | **Coronary artery disease** | **Published GWAS** | **Weighted median** | 1.53 | 1.30 | 1.81 | 6.E-07 |  |  |  |  |  |
| **Cardiovascular and metabolic** | **Coronary artery disease** | **Published GWAS** | **MR-Egger** | 1.65 | 1.19 | 2.30 | 0.004 | 0.00 | 0.825 | 168.60 | 6.E-10 | 0.96 |
| **Cardiovascular and metabolic** | **Coronary artery disease** | **Published GWAS** | **Penalised weighted median** | 1.60 | 1.33 | 1.92 | 8.E-07 |  |  |  |  |  |
| **Cardiovascular and metabolic** | **Deep vein thrombosis** | **FinnGen** | **Inverse-variance weighted** | 1.68 | 1.20 | 2.37 | 0.004 |  |  | 117.12 | 6.E-04 |  |
| **Cardiovascular and metabolic** | **Deep vein thrombosis** | **FinnGen** | **Weighted median** | 2.34 | 1.50 | 3.67 | 2.E-04 |  |  |  |  |  |
| **Cardiovascular and metabolic** | **Deep vein thrombosis** | **FinnGen** | **MR-Egger** | 1.69 | 0.83 | 3.47 | 0.155 | 0.00 | 0.987 | 117.12 | 5.E-04 | 0.96 |
| **Cardiovascular and metabolic** | **Deep vein thrombosis** | **FinnGen** | **Penalised weighted median** | 2.36 | 1.48 | 3.76 | 3.E-04 |  |  |  |  |  |
| **Cardiovascular and metabolic** | **Heart failure** | **FinnGen** | **Inverse-variance weighted** | 2.05 | 1.68 | 2.50 | 7.E-10 |  |  | 86.31 | 0.120 |  |
| **Cardiovascular and metabolic** | **Heart failure** | **FinnGen** | **Weighted median** | 1.66 | 1.24 | 2.23 | 7.E-04 |  |  |  |  |  |
| **Cardiovascular and metabolic** | **Heart failure** | **FinnGen** | **MR-Egger** | 1.10 | 0.75 | 1.61 | 0.623 | 0.02 | 5.E-04 | 72.83 | 0.418 | 0.96 |
| **Cardiovascular and metabolic** | **Heart failure** | **FinnGen** | **Penalised weighted median** | 1.66 | 1.23 | 2.24 | 9.E-04 |  |  |  |  |  |
| **Cardiovascular and metabolic** | **Heart failure** | **Published GWAS** | **Inverse-variance weighted** | 1.75 | 1.54 | 2.00 | 1.E-12 |  |  | 164.10 | 4.E-09 |  |
| **Cardiovascular and metabolic** | **Heart failure** | **Published GWAS** | **Weighted median** | 1.79 | 1.54 | 2.09 | 8.E-14 |  |  |  |  |  |
| **Cardiovascular and metabolic** | **Heart failure** | **Published GWAS** | **MR-Egger** | 1.61 | 1.23 | 2.12 | 1.E-03 | 0.00 | 0.495 | 163.02 | 3.E-09 | 0.96 |
| **Cardiovascular and metabolic** | **Heart failure** | **Published GWAS** | **Penalised weighted median** | 1.73 | 1.47 | 2.04 | 9.E-11 |  |  |  |  |  |
| **Cardiovascular and metabolic** | **Hypertension** | **FinnGen** | **Inverse-variance weighted** | 2.18 | 1.80 | 2.64 | 2.E-11 |  |  | 176.19 | 1.E-10 |  |
| **Cardiovascular and metabolic** | **Hypertension** | **FinnGen** | **Weighted median** | 2.29 | 1.87 | 2.81 | 2.E-15 |  |  |  |  |  |
| **Cardiovascular and metabolic** | **Hypertension** | **FinnGen** | **MR-Egger** | 1.60 | 1.08 | 2.37 | 0.022 | 0.01 | 0.084 | 168.89 | 6.E-10 | 0.96 |
| **Cardiovascular and metabolic** | **Hypertension** | **FinnGen** | **Penalised weighted median** | 1.82 | 1.45 | 2.28 | 2.E-07 |  |  |  |  |  |
| **Cardiovascular and metabolic** | **Ischemic heart disease** | **FinnGen** | **Inverse-variance weighted** | 1.24 | 1.06 | 1.45 | 0.009 |  |  | 104.84 | 0.007 |  |
| **Cardiovascular and metabolic** | **Ischemic heart disease** | **FinnGen** | **Weighted median** | 1.23 | 0.96 | 1.56 | 0.098 |  |  |  |  |  |
| **Cardiovascular and metabolic** | **Ischemic heart disease** | **FinnGen** | **MR-Egger** | 1.10 | 0.79 | 1.53 | 0.568 | 0.00 | 0.429 | 103.92 | 0.007 | 0.96 |
| **Cardiovascular and metabolic** | **Ischemic heart disease** | **FinnGen** | **Penalised weighted median** | 1.24 | 1.01 | 1.52 | 0.043 |  |  |  |  |  |
| **Cardiovascular and metabolic** | **Ischemic stroke** | **FinnGen** | **Inverse-variance weighted** | 1.19 | 0.96 | 1.48 | 0.119 |  |  | 101.91 | 0.012 |  |
| **Cardiovascular and metabolic** | **Ischemic stroke** | **FinnGen** | **Weighted median** | 1.13 | 0.82 | 1.57 | 0.447 |  |  |  |  |  |
| **Cardiovascular and metabolic** | **Ischemic stroke** | **FinnGen** | **MR-Egger** | 0.92 | 0.59 | 1.45 | 0.730 | 0.01 | 0.213 | 99.69 | 0.014 | 0.96 |
| **Cardiovascular and metabolic** | **Ischemic stroke** | **FinnGen** | **Penalised weighted median** | 1.12 | 0.85 | 1.47 | 0.433 |  |  |  |  |  |
| **Cardiovascular and metabolic** | **Ischemic stroke** | **Published GWAS** | **Inverse-variance weighted** | 1.18 | 1.03 | 1.35 | 0.018 |  |  | 110.66 | 0.002 |  |
| **Cardiovascular and metabolic** | **Ischemic stroke** | **Published GWAS** | **Weighted median** | 1.13 | 0.94 | 1.36 | 0.195 |  |  |  |  |  |
| **Cardiovascular and metabolic** | **Ischemic stroke** | **Published GWAS** | **MR-Egger** | 1.13 | 0.85 | 1.51 | 0.409 | 0.00 | 0.729 | 110.47 | 0.002 | 0.96 |
| **Cardiovascular and metabolic** | **Ischemic stroke** | **Published GWAS** | **Penalised weighted median** | 1.10 | 0.93 | 1.30 | 0.266 |  |  |  |  |  |
| **Cardiovascular and metabolic** | **Large artery stroke** | **Published GWAS** | **Inverse-variance weighted** | 1.42 | 1.02 | 1.97 | 0.043 |  |  | 107.12 | 0.005 |  |
| **Cardiovascular and metabolic** | **Large artery stroke** | **Published GWAS** | **Weighted median** | 1.77 | 1.14 | 2.74 | 0.011 |  |  |  |  |  |
| **Cardiovascular and metabolic** | **Large artery stroke** | **Published GWAS** | **MR-Egger** | 1.29 | 0.64 | 2.62 | 0.483 | 0.00 | 0.773 | 107.00 | 0.004 | 0.96 |
| **Cardiovascular and metabolic** | **Large artery stroke** | **Published GWAS** | **Penalised weighted median** | 1.78 | 1.14 | 2.79 | 0.012 |  |  |  |  |  |
| **Cardiovascular and metabolic** | **Peripheral artery disease** | **FinnGen** | **Inverse-variance weighted** | 1.87 | 1.46 | 2.39 | 4.E-06 |  |  | 84.33 | 0.152 |  |
| **Cardiovascular and metabolic** | **Peripheral artery disease** | **FinnGen** | **Weighted median** | 1.44 | 0.99 | 2.11 | 0.058 |  |  |  |  |  |
| **Cardiovascular and metabolic** | **Peripheral artery disease** | **FinnGen** | **MR-Egger** | 1.72 | 1.02 | 2.89 | 0.045 | 0.00 | 0.718 | 84.17 | 0.136 | 0.96 |
| **Cardiovascular and metabolic** | **Peripheral artery disease** | **FinnGen** | **Penalised weighted median** | 1.43 | 0.97 | 2.11 | 0.069 |  |  |  |  |  |
| **Cardiovascular and metabolic** | **Pulmonary embolism** | **FinnGen** | **Inverse-variance weighted** | 1.23 | 0.85 | 1.78 | 0.269 |  |  | 116.96 | 6.E-04 |  |
| **Cardiovascular and metabolic** | **Pulmonary embolism** | **FinnGen** | **Weighted median** | 1.35 | 0.80 | 2.28 | 0.260 |  |  |  |  |  |
| **Cardiovascular and metabolic** | **Pulmonary embolism** | **FinnGen** | **MR-Egger** | 1.72 | 0.80 | 3.73 | 0.172 | -0.01 | 0.337 | 115.44 | 7.E-04 | 0.96 |
| **Cardiovascular and metabolic** | **Pulmonary embolism** | **FinnGen** | **Penalised weighted median** | 1.37 | 0.82 | 2.30 | 0.230 |  |  |  |  |  |
| **Cardiovascular and metabolic** | **Small vessel stroke** | **Published GWAS** | **Inverse-variance weighted** | 1.00 | 0.72 | 1.39 | 0.994 |  |  | 123.45 | 2.E-04 |  |
| **Cardiovascular and metabolic** | **Small vessel stroke** | **Published GWAS** | **Weighted median** | 0.94 | 0.61 | 1.45 | 0.771 |  |  |  |  |  |
| **Cardiovascular and metabolic** | **Small vessel stroke** | **Published GWAS** | **MR-Egger** | 1.05 | 0.52 | 2.10 | 0.900 | 0.00 | 0.889 | 123.41 | 1.E-04 | 0.96 |
| **Cardiovascular and metabolic** | **Small vessel stroke** | **Published GWAS** | **Penalised weighted median** | 0.93 | 0.61 | 1.42 | 0.726 |  |  |  |  |  |
| **Cardiovascular and metabolic** | **Stroke** | **FinnGen** | **Inverse-variance weighted** | 1.24 | 1.05 | 1.47 | 0.013 |  |  | 84.56 | 0.148 |  |
| **Cardiovascular and metabolic** | **Stroke** | **FinnGen** | **Weighted median** | 1.10 | 0.86 | 1.40 | 0.458 |  |  |  |  |  |
| **Cardiovascular and metabolic** | **Stroke** | **FinnGen** | **MR-Egger** | 0.95 | 0.67 | 1.33 | 0.750 | 0.01 | 0.081 | 80.99 | 0.196 | 0.96 |
| **Cardiovascular and metabolic** | **Stroke** | **FinnGen** | **Penalised weighted median** | 1.16 | 0.90 | 1.50 | 0.256 |  |  |  |  |  |
| **Cardiovascular and metabolic** | **Stroke** | **Published GWAS** | **Inverse-variance weighted** | 1.15 | 1.02 | 1.31 | 0.030 |  |  | 117.31 | 6.E-04 |  |
| **Cardiovascular and metabolic** | **Stroke** | **Published GWAS** | **Weighted median** | 1.09 | 0.92 | 1.29 | 0.330 |  |  |  |  |  |
| **Cardiovascular and metabolic** | **Stroke** | **Published GWAS** | **MR-Egger** | 1.06 | 0.81 | 1.39 | 0.658 | 0.00 | 0.497 | 116.54 | 5.E-04 | 0.96 |
| **Cardiovascular and metabolic** | **Stroke** | **Published GWAS** | **Penalised weighted median** | 1.07 | 0.89 | 1.28 | 0.467 |  |  |  |  |  |
| **Cardiovascular and metabolic** | **Stroke (excl. subarachnoid hemorrhage)** | **FinnGen** | **Inverse-variance weighted** | 1.18 | 0.96 | 1.45 | 0.126 |  |  | 103.67 | 0.009 |  |
| **Cardiovascular and metabolic** | **Stroke (excl. subarachnoid hemorrhage)** | **FinnGen** | **Weighted median** | 1.12 | 0.83 | 1.51 | 0.471 |  |  |  |  |  |
| **Cardiovascular and metabolic** | **Stroke (excl. subarachnoid hemorrhage)** | **FinnGen** | **MR-Egger** | 0.89 | 0.58 | 1.38 | 0.612 | 0.01 | 0.158 | 100.78 | 0.012 | 0.96 |
| **Cardiovascular and metabolic** | **Stroke (excl. subarachnoid hemorrhage)** | **FinnGen** | **Penalised weighted median** | 1.12 | 0.82 | 1.53 | 0.483 |  |  |  |  |  |
| **Cardiovascular and metabolic** | **Venous thromboembolism** | **FinnGen** | **Inverse-variance weighted** | 1.43 | 1.09 | 1.87 | 0.011 |  |  | 135.12 | 1.E-05 |  |
| **Cardiovascular and metabolic** | **Venous thromboembolism** | **FinnGen** | **Weighted median** | 1.72 | 1.22 | 2.42 | 0.002 |  |  |  |  |  |
| **Cardiovascular and metabolic** | **Venous thromboembolism** | **FinnGen** | **MR-Egger** | 1.73 | 0.99 | 3.04 | 0.060 | -0.01 | 0.451 | 134.03 | 9.E-06 | 0.96 |
| **Cardiovascular and metabolic** | **Venous thromboembolism** | **FinnGen** | **Penalised weighted median** | 1.51 | 1.12 | 2.05 | 0.007 |  |  |  |  |  |
| **Cardiovascular and metabolic** | **Venous thromboembolism** | **Published GWAS** | **Inverse-variance weighted** | 1.46 | 1.25 | 1.72 | 1.E-05 |  |  | 72.81 | 0.451 |  |
| **Cardiovascular and metabolic** | **Venous thromboembolism** | **Published GWAS** | **Weighted median** | 1.54 | 1.19 | 1.99 | 1.E-03 |  |  |  |  |  |
| **Cardiovascular and metabolic** | **Venous thromboembolism** | **Published GWAS** | **MR-Egger** | 1.56 | 1.11 | 2.18 | 0.012 | 0.00 | 0.679 | 72.63 | 0.424 | 0.96 |
| **Cardiovascular and metabolic** | **Venous thromboembolism** | **Published GWAS** | **Penalised weighted median** | 1.54 | 1.17 | 2.02 | 0.002 |  |  |  |  |  |
| **Cardiovascular and metabolic** | **Polycystic ovary syndrome** | **FinnGen** | **Inverse-variance weighted** | 3.58 | 1.62 | 7.90 | 0.002 |  |  | 86.87 | 0.112 |  |
| **Cardiovascular and metabolic** | **Polycystic ovary syndrome** | **FinnGen** | **Weighted median** | 2.94 | 0.96 | 9.07 | 0.060 |  |  |  |  |  |
| **Cardiovascular and metabolic** | **Polycystic ovary syndrome** | **FinnGen** | **MR-Egger** | 8.81 | 1.69 | 45.96 | 0.012 | -0.02 | 0.228 | 85.10 | 0.121 | 0.96 |
| **Cardiovascular and metabolic** | **Polycystic ovary syndrome** | **FinnGen** | **Penalised weighted median** | 1.98 | 0.58 | 6.76 | 0.276 |  |  |  |  |  |
| **Cardiovascular and metabolic** | **Polycystic ovary syndrome** | **Published GWAS** | **Inverse-variance weighted** | 4.23 | 2.96 | 6.06 | 2.E-11 |  |  | 81.44 | 0.209 |  |
| **Cardiovascular and metabolic** | **Polycystic ovary syndrome** | **Published GWAS** | **Weighted median** | 3.84 | 2.13 | 6.95 | 8.E-06 |  |  |  |  |  |
| **Cardiovascular and metabolic** | **Polycystic ovary syndrome** | **Published GWAS** | **MR-Egger** | 2.79 | 1.31 | 5.92 | 0.010 | 0.01 | 0.221 | 79.73 | 0.224 | 0.96 |
| **Cardiovascular and metabolic** | **Polycystic ovary syndrome** | **Published GWAS** | **Penalised weighted median** | 3.65 | 2.05 | 6.50 | 1.E-05 |  |  |  |  |  |
| **Cardiovascular and metabolic** | **Type 2 diabetes** | **FinnGen** | **Inverse-variance weighted** | 3.04 | 2.47 | 3.74 | 3.E-16 |  |  | 188.97 | 2.E-12 |  |
| **Cardiovascular and metabolic** | **Type 2 diabetes** | **FinnGen** | **Weighted median** | 3.39 | 2.67 | 4.32 | 3.E-23 |  |  |  |  |  |
| **Cardiovascular and metabolic** | **Type 2 diabetes** | **FinnGen** | **MR-Egger** | 3.06 | 1.98 | 4.72 | 3.E-06 | 0.00 | 0.975 | 188.97 | 1.E-12 | 0.96 |
| **Cardiovascular and metabolic** | **Type 2 diabetes** | **FinnGen** | **Penalised weighted median** | 2.02 | 1.53 | 2.67 | 7.E-07 |  |  |  |  |  |
| **Cardiovascular and metabolic** | **Type 2 diabetes** | **Published GWAS** | **Inverse-variance weighted** | 2.91 | 2.45 | 3.47 | 9.E-19 |  |  | 271.64 | 6.E-25 |  |
| **Cardiovascular and metabolic** | **Type 2 diabetes** | **Published GWAS** | **Weighted median** | 2.99 | 2.49 | 3.60 | 7.E-32 |  |  |  |  |  |
| **Cardiovascular and metabolic** | **Type 2 diabetes** | **Published GWAS** | **MR-Egger** | 3.40 | 2.36 | 4.92 | 9.E-09 | 0.00 | 0.349 | 268.28 | 1.E-24 | 0.96 |
| **Cardiovascular and metabolic** | **Type 2 diabetes** | **Published GWAS** | **Penalised weighted median** | 2.11 | 1.66 | 2.69 | 1.E-09 |  |  |  |  |  |
| **Cardiovascular and metabolic** | **Chronic kidney disease** | **FinnGen** | **Inverse-variance weighted** | 1.34 | 0.98 | 1.83 | 0.072 |  |  | 79.38 | 0.258 |  |
| **Cardiovascular and metabolic** | **Chronic kidney disease** | **FinnGen** | **Weighted median** | 1.68 | 1.04 | 2.70 | 0.034 |  |  |  |  |  |
| **Cardiovascular and metabolic** | **Chronic kidney disease** | **FinnGen** | **MR-Egger** | 1.53 | 0.79 | 2.96 | 0.210 | 0.00 | 0.652 | 79.15 | 0.237 | 0.96 |
| **Cardiovascular and metabolic** | **Chronic kidney disease** | **FinnGen** | **Penalised weighted median** | 1.10 | 0.68 | 1.79 | 0.700 |  |  |  |  |  |
| **Cardiovascular and metabolic** | **Chronic kidney disease** | **Published GWAS** | **Inverse-variance weighted** | 1.19 | 1.05 | 1.35 | 0.009 |  |  | 114.22 | 0.001 |  |
| **Cardiovascular and metabolic** | **Chronic kidney disease** | **Published GWAS** | **Weighted median** | 1.23 | 1.03 | 1.47 | 0.022 |  |  |  |  |  |
| **Cardiovascular and metabolic** | **Chronic kidney disease** | **Published GWAS** | **MR-Egger** | 1.01 | 0.78 | 1.31 | 0.943 | 0.00 | 0.171 | 111.22 | 0.002 | 0.96 |
| **Cardiovascular and metabolic** | **Chronic kidney disease** | **Published GWAS** | **Penalised weighted median** | 1.23 | 1.05 | 1.44 | 0.011 |  |  |  |  |  |
| **Musculoskeletal** | **Gout** | **FinnGen** | **Inverse-variance weighted** | 1.69 | 1.16 | 2.46 | 0.008 |  |  | 100.33 | 0.015 |  |
| **Musculoskeletal** | **Gout** | **FinnGen** | **Weighted median** | 1.51 | 0.86 | 2.65 | 0.149 |  |  |  |  |  |
| **Musculoskeletal** | **Gout** | **FinnGen** | **MR-Egger** | 1.12 | 0.51 | 2.46 | 0.775 | 0.01 | 0.247 | 98.44 | 0.017 | 0.96 |
| **Musculoskeletal** | **Gout** | **FinnGen** | **Penalised weighted median** | 1.49 | 0.85 | 2.63 | 0.164 |  |  |  |  |  |
| **Musculoskeletal** | **Gout** | **Published GWAS** | **Inverse-variance weighted** | 1.68 | 1.39 | 2.04 | 1.E-06 |  |  | 136.63 | 7.E-06 |  |
| **Musculoskeletal** | **Gout** | **Published GWAS** | **Weighted median** | 1.70 | 1.32 | 2.18 | 4.E-05 |  |  |  |  |  |
| **Musculoskeletal** | **Gout** | **Published GWAS** | **MR-Egger** | 1.82 | 1.21 | 2.74 | 0.005 | 0.00 | 0.664 | 136.27 | 5.E-06 | 0.96 |
| **Musculoskeletal** | **Gout** | **Published GWAS** | **Penalised weighted median** | 1.69 | 1.25 | 2.28 | 6.E-04 |  |  |  |  |  |
| **Musculoskeletal** | **Osteoarthritis** | **FinnGen** | **Inverse-variance weighted** | 1.95 | 1.66 | 2.29 | 7.E-12 |  |  | 137.18 | 6.E-06 |  |
| **Musculoskeletal** | **Osteoarthritis** | **FinnGen** | **Weighted median** | 2.11 | 1.72 | 2.60 | 2.E-12 |  |  |  |  |  |
| **Musculoskeletal** | **Osteoarthritis** | **FinnGen** | **MR-Egger** | 1.88 | 1.34 | 2.63 | 5.E-04 | 0.00 | 0.802 | 137.06 | 4.E-06 | 0.96 |
| **Musculoskeletal** | **Osteoarthritis** | **FinnGen** | **Penalised weighted median** | 1.56 | 1.23 | 1.98 | 2.E-04 |  |  |  |  |  |
| **Musculoskeletal** | **Osteoarthritis** | **Published GWAS** | **Inverse-variance weighted** | 1.55 | 1.42 | 1.69 | 9.E-15 |  |  | 156.99 | 3.E-08 |  |
| **Musculoskeletal** | **Osteoarthritis** | **Published GWAS** | **Weighted median** | 1.46 | 1.33 | 1.60 | 1.E-14 |  |  |  |  |  |
| **Musculoskeletal** | **Osteoarthritis** | **Published GWAS** | **MR-Egger** | 1.41 | 1.17 | 1.70 | 5.E-04 | 0.00 | 0.275 | 154.36 | 4.E-08 | 0.96 |
| **Musculoskeletal** | **Osteoarthritis** | **Published GWAS** | **Penalised weighted median** | 1.46 | 1.30 | 1.64 | 3.E-10 |  |  |  |  |  |
| **Musculoskeletal** | **Osteoarthritis (hip and/or knee)** | **Published GWAS** | **Inverse-variance weighted** | 1.80 | 1.59 | 2.03 | 4.E-14 |  |  | 159.89 | 1.E-08 |  |
| **Musculoskeletal** | **Osteoarthritis (hip and/or knee)** | **Published GWAS** | **Weighted median** | 1.85 | 1.60 | 2.14 | 7.E-17 |  |  |  |  |  |
| **Musculoskeletal** | **Osteoarthritis (hip and/or knee)** | **Published GWAS** | **MR-Egger** | 1.72 | 1.33 | 2.23 | 1.E-04 | 0.00 | 0.699 | 159.56 | 9.E-09 | 0.96 |
| **Musculoskeletal** | **Osteoarthritis (hip and/or knee)** | **Published GWAS** | **Penalised weighted median** | 1.85 | 1.57 | 2.16 | 4.E-14 |  |  |  |  |  |
| **Musculoskeletal** | **Osteoarthritis (hip)** | **FinnGen** | **Inverse-variance weighted** | 2.05 | 1.64 | 2.57 | 3.E-08 |  |  | 100.05 | 0.016 |  |
| **Musculoskeletal** | **Osteoarthritis (hip)** | **FinnGen** | **Weighted median** | 2.19 | 1.60 | 2.99 | 1.E-06 |  |  |  |  |  |
| **Musculoskeletal** | **Osteoarthritis (hip)** | **FinnGen** | **MR-Egger** | 1.69 | 1.05 | 2.71 | 0.033 | 0.01 | 0.361 | 98.87 | 0.016 | 0.96 |
| **Musculoskeletal** | **Osteoarthritis (hip)** | **FinnGen** | **Penalised weighted median** | 1.96 | 1.42 | 2.69 | 4.E-05 |  |  |  |  |  |
| **Musculoskeletal** | **Osteoarthritis (knee)** | **FinnGen** | **Inverse-variance weighted** | 2.47 | 2.04 | 3.00 | 7.E-14 |  |  | 140.03 | 3.E-06 |  |
| **Musculoskeletal** | **Osteoarthritis (knee)** | **FinnGen** | **Weighted median** | 2.57 | 2.05 | 3.22 | 3.E-16 |  |  |  |  |  |
| **Musculoskeletal** | **Osteoarthritis (knee)** | **FinnGen** | **MR-Egger** | 2.57 | 1.72 | 3.85 | 2.E-05 | 0.00 | 0.831 | 139.94 | 2.E-06 | 0.96 |
| **Musculoskeletal** | **Osteoarthritis (knee)** | **FinnGen** | **Penalised weighted median** | 1.76 | 1.33 | 2.33 | 7.E-05 |  |  |  |  |  |
| **Musculoskeletal** | **Osteoporosis** | **FinnGen** | **Inverse-variance weighted** | 0.85 | 0.62 | 1.17 | 0.324 |  |  | 65.18 | 0.702 |  |
| **Musculoskeletal** | **Osteoporosis** | **FinnGen** | **Weighted median** | 0.74 | 0.43 | 1.27 | 0.275 |  |  |  |  |  |
| **Musculoskeletal** | **Osteoporosis** | **FinnGen** | **MR-Egger** | 1.14 | 0.58 | 2.23 | 0.706 | -0.01 | 0.311 | 64.23 | 0.702 | 0.96 |
| **Musculoskeletal** | **Osteoporosis** | **FinnGen** | **Penalised weighted median** | 0.74 | 0.45 | 1.23 | 0.247 |  |  |  |  |  |
| **Musculoskeletal** | **Osteoporosis** | **Published GWAS** | **Inverse-variance weighted** | 1.17 | 1.10 | 1.24 | 3.E-06 |  |  | 648.83 | 1.E-93 |  |
| **Musculoskeletal** | **Osteoporosis** | **Published GWAS** | **Weighted median** | 1.28 | 1.24 | 1.33 | 3.E-37 |  |  |  |  |  |
| **Musculoskeletal** | **Osteoporosis** | **Published GWAS** | **MR-Egger** | 1.34 | 1.18 | 1.51 | 1.E-05 | 0.00 | 0.016 | 597.66 | 3.E-84 | 0.96 |
| **Musculoskeletal** | **Osteoporosis** | **Published GWAS** | **Penalised weighted median** | 1.20 | 1.12 | 1.28 | 3.E-08 |  |  |  |  |  |
| **Musculoskeletal** | **Rheumatoid arthritis** | **FinnGen** | **Inverse-variance weighted** | 1.27 | 0.99 | 1.63 | 0.062 |  |  | 75.81 | 0.357 |  |
| **Musculoskeletal** | **Rheumatoid arthritis** | **FinnGen** | **Weighted median** | 1.27 | 0.83 | 1.94 | 0.269 |  |  |  |  |  |
| **Musculoskeletal** | **Rheumatoid arthritis** | **FinnGen** | **MR-Egger** | 1.04 | 0.62 | 1.75 | 0.878 | 0.01 | 0.396 | 75.04 | 0.349 | 0.96 |
| **Musculoskeletal** | **Rheumatoid arthritis** | **FinnGen** | **Penalised weighted median** | 1.27 | 0.84 | 1.94 | 0.259 |  |  |  |  |  |
| **Musculoskeletal** | **Rheumatoid arthritis** | **Published GWAS** | **Inverse-variance weighted** | 1.20 | 0.95 | 1.52 | 0.137 |  |  | 105.85 | 0.005 |  |
| **Musculoskeletal** | **Rheumatoid arthritis** | **Published GWAS** | **Weighted median** | 0.96 | 0.70 | 1.32 | 0.793 |  |  |  |  |  |
| **Musculoskeletal** | **Rheumatoid arthritis** | **Published GWAS** | **MR-Egger** | 0.85 | 0.53 | 1.37 | 0.515 | 0.01 | 0.112 | 102.06 | 0.007 | 0.97 |
| **Musculoskeletal** | **Rheumatoid arthritis** | **Published GWAS** | **Penalised weighted median** | 0.95 | 0.68 | 1.33 | 0.748 |  |  |  |  |  |
| **Gastrointestinal** | **Gallstones** | **FinnGen** | **Inverse-variance weighted** | 1.87 | 1.58 | 2.23 | 7.E-10 |  |  | 104.09 | 0.008 |  |
| **Gastrointestinal** | **Gallstones** | **FinnGen** | **Weighted median** | 1.82 | 1.45 | 2.28 | 3.E-07 |  |  |  |  |  |
| **Gastrointestinal** | **Gallstones** | **FinnGen** | **MR-Egger** | 2.06 | 1.43 | 2.97 | 2.E-04 | 0.00 | 0.562 | 103.60 | 0.007 | 0.96 |
| **Gastrointestinal** | **Gallstones** | **FinnGen** | **Penalised weighted median** | 1.50 | 1.16 | 1.94 | 0.002 |  |  |  |  |  |
| **Gastrointestinal** | **Gastro-oesophageal reflux disease** | **Published GWAS** | **Inverse-variance weighted** | 1.17 | 1.06 | 1.29 | 0.003 |  |  | 179.07 | 4.E-11 |  |
| **Gastrointestinal** | **Gastro-oesophageal reflux disease** | **Published GWAS** | **Weighted median** | 1.05 | 0.93 | 1.19 | 0.430 |  |  |  |  |  |
| **Gastrointestinal** | **Gastro-oesophageal reflux disease** | **Published GWAS** | **MR-Egger** | 1.00 | 0.81 | 1.23 | 0.997 | 0.00 | 0.105 | 172.52 | 2.E-10 | 0.96 |
| **Gastrointestinal** | **Gastro-oesophageal reflux disease** | **Published GWAS** | **Penalised weighted median** | 1.08 | 0.95 | 1.21 | 0.229 |  |  |  |  |  |
| **Nervous** | **Alzheimer's disease** | **FinnGen** | **Inverse-variance weighted** | 1.02 | 0.48 | 2.18 | 0.963 |  |  | 442.20 | 1.E-54 |  |
| **Nervous** | **Alzheimer's disease** | **FinnGen** | **Weighted median** | 1.31 | 0.82 | 2.09 | 0.267 |  |  |  |  |  |
| **Nervous** | **Alzheimer's disease** | **FinnGen** | **MR-Egger** | 1.37 | 0.28 | 6.81 | 0.698 | -0.01 | 0.677 | 441.11 | 8.E-55 | 0.96 |
| **Nervous** | **Alzheimer's disease** | **FinnGen** | **Penalised weighted median** | 1.45 | 0.89 | 2.36 | 0.137 |  |  |  |  |  |
| **Nervous** | **Alzheimer's disease** | **Published GWAS (1)** | **Inverse-variance weighted** | 0.98 | 0.85 | 1.13 | 0.782 |  |  | 2848.14 | 0.E+00 |  |
| **Nervous** | **Alzheimer's disease** | **Published GWAS (1)** | **Weighted median** | 1.03 | 0.99 | 1.08 | 0.132 |  |  |  |  |  |
| **Nervous** | **Alzheimer's disease** | **Published GWAS (1)** | **MR-Egger** | 0.97 | 0.72 | 1.32 | 0.863 | 0.00 | 0.961 | 2848.04 | 0.E+00 | 0.97 |
| **Nervous** | **Alzheimer's disease** | **Published GWAS (1)** | **Penalised weighted median** | 1.06 | 1.00 | 1.12 | 0.037 |  |  |  |  |  |
| **Nervous** | **Alzheimer's disease** | **Published GWAS (2)** | **Inverse-variance weighted** | 0.63 | 0.24 | 1.62 | 0.339 |  |  | 2712.26 | 0.E+00 |  |
| **Nervous** | **Alzheimer's disease** | **Published GWAS (2)** | **Weighted median** | 1.00 | 0.78 | 1.29 | 0.993 |  |  |  |  |  |
| **Nervous** | **Alzheimer's disease** | **Published GWAS (2)** | **MR-Egger** | 0.66 | 0.09 | 4.99 | 0.689 | 0.00 | 0.954 | 2712.13 | 0.E+00 | 0.96 |
| **Nervous** | **Alzheimer's disease** | **Published GWAS (2)** | **Penalised weighted median** | 0.85 | 0.65 | 1.12 | 0.256 |  |  |  |  |  |
| **Nervous** | **Depression** | **FinnGen** | **Inverse-variance weighted** | 1.05 | 0.92 | 1.20 | 0.503 |  |  | 68.76 | 0.586 |  |
| **Nervous** | **Depression** | **FinnGen** | **Weighted median** | 1.08 | 0.86 | 1.34 | 0.511 |  |  |  |  |  |
| **Nervous** | **Depression** | **FinnGen** | **MR-Egger** | 0.88 | 0.67 | 1.16 | 0.377 | 0.00 | 0.160 | 66.86 | 0.617 | 0.96 |
| **Nervous** | **Depression** | **FinnGen** | **Penalised weighted median** | 1.08 | 0.88 | 1.31 | 0.474 |  |  |  |  |  |
| **Nervous** | **Depression** | **Published GWAS** | **Inverse-variance weighted** | 1.19 | 1.04 | 1.37 | 0.014 |  |  | 145.94 | 6.E-07 |  |
| **Nervous** | **Depression** | **Published GWAS** | **Weighted median** | 1.22 | 1.02 | 1.46 | 0.030 |  |  |  |  |  |
| **Nervous** | **Depression** | **Published GWAS** | **MR-Egger** | 1.12 | 0.84 | 1.50 | 0.439 | 0.00 | 0.646 | 145.50 | 5.E-07 | 0.96 |
| **Nervous** | **Depression** | **Published GWAS** | **Penalised weighted median** | 1.22 | 1.01 | 1.47 | 0.036 |  |  |  |  |  |
| **Nervous** | **Multiple sclerosis** | **FinnGen** | **Inverse-variance weighted** | 0.81 | 0.47 | 1.40 | 0.443 |  |  | 72.79 | 0.452 |  |
| **Nervous** | **Multiple sclerosis** | **FinnGen** | **Weighted median** | 0.65 | 0.26 | 1.64 | 0.366 |  |  |  |  |  |
| **Nervous** | **Multiple sclerosis** | **FinnGen** | **MR-Egger** | 0.99 | 0.31 | 3.15 | 0.990 | -0.01 | 0.688 | 72.62 | 0.424 | 0.96 |
| **Nervous** | **Multiple sclerosis** | **FinnGen** | **Penalised weighted median** | 0.66 | 0.27 | 1.58 | 0.346 |  |  |  |  |  |
| **Nervous** | **Parkinson's disease** | **FinnGen** | **Inverse-variance weighted** | 0.89 | 0.54 | 1.47 | 0.647 |  |  | 109.01 | 0.003 |  |
| **Nervous** | **Parkinson's disease** | **FinnGen** | **Weighted median** | 1.22 | 0.62 | 2.42 | 0.560 |  |  |  |  |  |
| **Nervous** | **Parkinson's disease** | **FinnGen** | **MR-Egger** | 0.55 | 0.19 | 1.56 | 0.263 | 0.01 | 0.305 | 107.40 | 0.003 | 0.96 |
| **Nervous** | **Parkinson's disease** | **FinnGen** | **Penalised weighted median** | 1.33 | 0.67 | 2.63 | 0.412 |  |  |  |  |  |
| **Nervous** | **Parkinson's disease** | **Published GWAS** | **Inverse-variance weighted** | 0.98 | 0.77 | 1.24 | 0.842 |  |  | 115.96 | 8.E-04 |  |
| **Nervous** | **Parkinson's disease** | **Published GWAS** | **Weighted median** | 0.96 | 0.71 | 1.28 | 0.757 |  |  |  |  |  |
| **Nervous** | **Parkinson's disease** | **Published GWAS** | **MR-Egger** | 0.95 | 0.57 | 1.59 | 0.850 | 0.00 | 0.914 | 115.94 | 6.E-04 | 0.97 |
| **Nervous** | **Parkinson's disease** | **Published GWAS** | **Penalised weighted median** | 1.08 | 0.80 | 1.45 | 0.613 |  |  |  |  |  |
| **Integumentary** | **Psoriasis** | **FinnGen** | **Inverse-variance weighted** | 1.39 | 1.05 | 1.84 | 0.025 |  |  | 74.95 | 0.383 |  |
| **Integumentary** | **Psoriasis** | **FinnGen** | **Weighted median** | 1.21 | 0.76 | 1.91 | 0.427 |  |  |  |  |  |
| **Integumentary** | **Psoriasis** | **FinnGen** | **MR-Egger** | 1.35 | 0.75 | 2.44 | 0.325 | 0.00 | 0.911 | 74.94 | 0.352 | 0.96 |
| **Integumentary** | **Psoriasis** | **FinnGen** | **Penalised weighted median** | 1.22 | 0.77 | 1.93 | 0.403 |  |  |  |  |  |
| **Integumentary** | **Psoriasis** | **Published GWAS** | **Inverse-variance weighted** | 1.89 | 1.44 | 2.47 | 2.E-05 |  |  | 134.25 | 1.E-05 |  |
| **Integumentary** | **Psoriasis** | **Published GWAS** | **Weighted median** | 1.99 | 1.43 | 2.77 | 5.E-05 |  |  |  |  |  |
| **Integumentary** | **Psoriasis** | **Published GWAS** | **MR-Egger** | 1.92 | 1.10 | 3.36 | 0.026 | 0.00 | 0.944 | 134.25 | 9.E-06 | 0.97 |
| **Integumentary** | **Psoriasis** | **Published GWAS** | **Penalised weighted median** | 1.99 | 1.42 | 2.81 | 8.E-05 |  |  |  |  |  |
| **Respiratory** | **Adult-onset asthma** | **Published GWAS** | **Inverse-variance weighted** | 1.14 | 1.01 | 1.29 | 0.040 |  |  | 135.39 | 9.E-06 |  |
| **Respiratory** | **Adult-onset asthma** | **Published GWAS** | **Weighted median** | 1.16 | 0.98 | 1.37 | 0.076 |  |  |  |  |  |
| **Respiratory** | **Adult-onset asthma** | **Published GWAS** | **MR-Egger** | 1.07 | 0.83 | 1.40 | 0.596 | 0.00 | 0.613 | 134.90 | 7.E-06 | 0.96 |
| **Respiratory** | **Adult-onset asthma** | **Published GWAS** | **Penalised weighted median** | 1.16 | 0.97 | 1.38 | 0.096 |  |  |  |  |  |
| **Respiratory** | **Asthma** | **FinnGen** | **Inverse-variance weighted** | 1.39 | 1.19 | 1.62 | 7.E-05 |  |  | 85.79 | 0.128 |  |
| **Respiratory** | **Asthma** | **FinnGen** | **Weighted median** | 1.41 | 1.12 | 1.78 | 0.004 |  |  |  |  |  |
| **Respiratory** | **Asthma** | **FinnGen** | **MR-Egger** | 1.14 | 0.83 | 1.56 | 0.424 | 0.01 | 0.165 | 83.47 | 0.148 | 0.96 |
| **Respiratory** | **Asthma** | **FinnGen** | **Penalised weighted median** | 1.40 | 1.13 | 1.73 | 0.002 |  |  |  |  |  |
| **Respiratory** | **Child-onset asthma** | **Published GWAS** | **Inverse-variance weighted** | 1.12 | 0.96 | 1.31 | 0.149 |  |  | 112.69 | 0.002 |  |
| **Respiratory** | **Child-onset asthma** | **Published GWAS** | **Weighted median** | 1.05 | 0.84 | 1.30 | 0.690 |  |  |  |  |  |
| **Respiratory** | **Child-onset asthma** | **Published GWAS** | **MR-Egger** | 1.02 | 0.73 | 1.42 | 0.910 | 0.00 | 0.518 | 112.02 | 0.001 | 0.96 |
| **Respiratory** | **Child-onset asthma** | **Published GWAS** | **Penalised weighted median** | 1.07 | 0.86 | 1.34 | 0.530 |  |  |  |  |  |
| **Cancer** | **Barrett's oesophagus** | **FinnGen** | **Inverse-variance weighted** | 1.29 | 0.55 | 3.02 | 0.558 |  |  | 58.69 | 0.871 |  |
| **Cancer** | **Barrett's oesophagus** | **FinnGen** | **Weighted median** | 2.09 | 0.56 | 7.80 | 0.275 |  |  |  |  |  |
| **Cancer** | **Barrett's oesophagus** | **FinnGen** | **MR-Egger** | 3.12 | 0.53 | 18.47 | 0.215 | -0.02 | 0.224 | 57.48 | 0.877 | 0.96 |
| **Cancer** | **Barrett's oesophagus** | **FinnGen** | **Penalised weighted median** | 2.10 | 0.54 | 8.15 | 0.284 |  |  |  |  |  |
| **Cancer** | **Breast cancer** | **FinnGen** | **Inverse-variance weighted** | 0.63 | 0.51 | 0.78 | 7.E-05 |  |  | 59.12 | 0.862 |  |
| **Cancer** | **Breast cancer** | **FinnGen** | **Weighted median** | 0.49 | 0.35 | 0.68 | 3.E-05 |  |  |  |  |  |
| **Cancer** | **Breast cancer** | **FinnGen** | **MR-Egger** | 0.43 | 0.27 | 0.67 | 4.E-04 | 0.01 | 0.033 | 55.43 | 0.913 | 0.96 |
| **Cancer** | **Breast cancer** | **FinnGen** | **Penalised weighted median** | 0.82 | 0.58 | 1.16 | 0.259 |  |  |  |  |  |
| **Cancer** | **Breast cancer** | **Published GWAS** | **Inverse-variance weighted** | 0.56 | 0.44 | 0.72 | 1.E-05 |  |  | 112.91 | 0.001 |  |
| **Cancer** | **Breast cancer** | **Published GWAS** | **Weighted median** | 0.57 | 0.41 | 0.78 | 6.E-04 |  |  |  |  |  |
| **Cancer** | **Breast cancer** | **Published GWAS** | **MR-Egger** | 0.48 | 0.29 | 0.80 | 0.006 | 0.00 | 0.485 | 112.13 | 0.001 | 0.96 |
| **Cancer** | **Breast cancer** | **Published GWAS** | **Penalised weighted median** | 0.76 | 0.53 | 1.08 | 0.123 |  |  |  |  |  |
| **Cancer** | **Cancer myeloma** | **FinnGen** | **Inverse-variance weighted** | 1.60 | 0.76 | 3.40 | 0.221 |  |  | 69.59 | 0.558 |  |
| **Cancer** | **Cancer myeloma** | **FinnGen** | **Weighted median** | 1.29 | 0.38 | 4.40 | 0.683 |  |  |  |  |  |
| **Cancer** | **Cancer myeloma** | **FinnGen** | **MR-Egger** | 1.68 | 0.35 | 8.09 | 0.521 | 0.00 | 0.949 | 69.59 | 0.525 | 0.96 |
| **Cancer** | **Cancer myeloma** | **FinnGen** | **Penalised weighted median** | 1.29 | 0.38 | 4.35 | 0.685 |  |  |  |  |  |
| **Cancer** | **Colorectal cancer** | **FinnGen** | **Inverse-variance weighted** | 1.07 | 0.75 | 1.50 | 0.718 |  |  | 84.07 | 0.156 |  |
| **Cancer** | **Colorectal cancer** | **FinnGen** | **Weighted median** | 1.28 | 0.73 | 2.23 | 0.387 |  |  |  |  |  |
| **Cancer** | **Colorectal cancer** | **FinnGen** | **MR-Egger** | 1.50 | 0.73 | 3.08 | 0.277 | -0.01 | 0.298 | 82.79 | 0.160 | 0.96 |
| **Cancer** | **Colorectal cancer** | **FinnGen** | **Penalised weighted median** | 1.30 | 0.72 | 2.34 | 0.391 |  |  |  |  |  |
| **Cancer** | **Colorectal cancer** | **Published GWAS (1)** | **Inverse-variance weighted** | 1.11 | 0.85 | 1.45 | 0.455 |  |  | 41.81 | 4.E-04 |  |
| **Cancer** | **Colorectal cancer** | **Published GWAS (1)** | **Weighted median** | 1.04 | 0.82 | 1.33 | 0.736 |  |  |  |  |  |
| **Cancer** | **Colorectal cancer** | **Published GWAS (1)** | **MR-Egger** | 1.12 | 0.57 | 2.20 | 0.753 | 0.00 | 0.983 | 41.81 | 2.E-04 | 0.98 |
| **Cancer** | **Colorectal cancer** | **Published GWAS (1)** | **Penalised weighted median** | 1.46 | 1.06 | 2.01 | 0.019 |  |  |  |  |  |
| **Cancer** | **Endometrial cancer** | **FinnGen** | **Inverse-variance weighted** | 1.32 | 0.71 | 2.45 | 0.376 |  |  | 97.99 | 0.023 |  |
| **Cancer** | **Endometrial cancer** | **FinnGen** | **Weighted median** | 0.63 | 0.26 | 1.52 | 0.303 |  |  |  |  |  |
| **Cancer** | **Endometrial cancer** | **FinnGen** | **MR-Egger** | 1.21 | 0.33 | 4.44 | 0.771 | 0.00 | 0.882 | 97.96 | 0.019 | 0.96 |
| **Cancer** | **Endometrial cancer** | **FinnGen** | **Penalised weighted median** | 0.63 | 0.26 | 1.50 | 0.298 |  |  |  |  |  |
| **Cancer** | **Endometrial cancer** | **Published GWAS** | **Inverse-variance weighted** | 2.03 | 1.67 | 2.47 | 6.E-10 |  |  | 99.82 | 0.017 |  |
| **Cancer** | **Endometrial cancer** | **Published GWAS** | **Weighted median** | 1.89 | 1.42 | 2.52 | 1.E-05 |  |  |  |  |  |
| **Cancer** | **Endometrial cancer** | **Published GWAS** | **MR-Egger** | 1.64 | 1.09 | 2.47 | 0.020 | 0.01 | 0.245 | 97.92 | 0.019 | 0.96 |
| **Cancer** | **Endometrial cancer** | **Published GWAS** | **Penalised weighted median** | 1.81 | 1.33 | 2.44 | 1.E-04 |  |  |  |  |  |
| **Cancer** | **Lung cancer** | **FinnGen** | **Inverse-variance weighted** | 1.21 | 0.75 | 1.96 | 0.444 |  |  | 94.71 | 0.038 |  |
| **Cancer** | **Lung cancer** | **FinnGen** | **Weighted median** | 0.78 | 0.41 | 1.51 | 0.466 |  |  |  |  |  |
| **Cancer** | **Lung cancer** | **FinnGen** | **MR-Egger** | 0.92 | 0.34 | 2.55 | 0.879 | 0.01 | 0.557 | 94.24 | 0.034 | 0.96 |
| **Cancer** | **Lung cancer** | **FinnGen** | **Penalised weighted median** | 0.93 | 0.49 | 1.77 | 0.818 |  |  |  |  |  |
| **Cancer** | **Meningioma** | **FinnGen** | **Inverse-variance weighted** | 0.62 | 0.36 | 1.06 | 0.085 |  |  | 82.68 | 0.183 |  |
| **Cancer** | **Meningioma** | **FinnGen** | **Weighted median** | 0.65 | 0.25 | 1.69 | 0.379 |  |  |  |  |  |
| **Cancer** | **Meningioma** | **FinnGen** | **MR-Egger** | 0.85 | 0.27 | 2.65 | 0.774 | -0.01 | 0.539 | 82.24 | 0.170 | 0.96 |
| **Cancer** | **Meningioma** | **FinnGen** | **Penalised weighted median** | 0.72 | 0.28 | 1.81 | 0.481 |  |  |  |  |  |
| **Cancer** | **Ovarian cancer** | **FinnGen** | **Inverse-variance weighted** | 0.81 | 0.42 | 1.58 | 0.547 |  |  | 80.20 | 0.238 |  |
| **Cancer** | **Ovarian cancer** | **FinnGen** | **Weighted median** | 0.95 | 0.30 | 2.94 | 0.924 |  |  |  |  |  |
| **Cancer** | **Ovarian cancer** | **FinnGen** | **MR-Egger** | 2.01 | 0.51 | 8.00 | 0.324 | -0.02 | 0.148 | 77.85 | 0.270 | 0.96 |
| **Cancer** | **Ovarian cancer** | **FinnGen** | **Penalised weighted median** | 0.95 | 0.31 | 2.88 | 0.921 |  |  |  |  |  |
| **Cancer** | **Ovarian cancer - clear cell cancer** | **Published GWAS** | **Inverse-variance weighted** | 1.19 | 0.75 | 1.89 | 0.472 |  |  | 82.03 | 0.196 |  |
| **Cancer** | **Ovarian cancer - clear cell cancer** | **Published GWAS** | **Weighted median** | 1.64 | 0.83 | 3.24 | 0.157 |  |  |  |  |  |
| **Cancer** | **Ovarian cancer - clear cell cancer** | **Published GWAS** | **MR-Egger** | 2.57 | 0.98 | 6.74 | 0.059 | -0.02 | 0.079 | 78.51 | 0.253 | 0.96 |
| **Cancer** | **Ovarian cancer - clear cell cancer** | **Published GWAS** | **Penalised weighted median** | 1.63 | 0.84 | 3.19 | 0.151 |  |  |  |  |  |
| **Cancer** | **Ovarian cancer - endometrioid** | **Published GWAS** | **Inverse-variance weighted** | 1.39 | 0.98 | 1.97 | 0.067 |  |  | 90.17 | 0.073 |  |
| **Cancer** | **Ovarian cancer - endometrioid** | **Published GWAS** | **Weighted median** | 1.48 | 0.85 | 2.58 | 0.166 |  |  |  |  |  |
| **Cancer** | **Ovarian cancer - endometrioid** | **Published GWAS** | **MR-Egger** | 1.69 | 0.81 | 3.52 | 0.168 | -0.01 | 0.560 | 89.73 | 0.066 | 0.96 |
| **Cancer** | **Ovarian cancer - endometrioid** | **Published GWAS** | **Penalised weighted median** | 1.52 | 0.87 | 2.66 | 0.138 |  |  |  |  |  |
| **Cancer** | **Ovarian cancer - epithelial** | **Published GWAS** | **Inverse-variance weighted** | 1.36 | 1.00 | 1.85 | 0.052 |  |  | 61.88 | 0.797 |  |
| **Cancer** | **Ovarian cancer - epithelial** | **Published GWAS** | **Weighted median** | 1.46 | 0.90 | 2.36 | 0.122 |  |  |  |  |  |
| **Cancer** | **Ovarian cancer - epithelial** | **Published GWAS** | **MR-Egger** | 1.58 | 0.83 | 3.02 | 0.169 | 0.00 | 0.582 | 61.61 | 0.779 | 0.96 |
| **Cancer** | **Ovarian cancer - epithelial** | **Published GWAS** | **Penalised weighted median** | 1.48 | 0.91 | 2.40 | 0.116 |  |  |  |  |  |
| **Cancer** | **Ovarian cancer - high grade serous** | **Published GWAS** | **Inverse-variance weighted** | 1.26 | 1.01 | 1.58 | 0.040 |  |  | 69.13 | 0.574 |  |
| **Cancer** | **Ovarian cancer - high grade serous** | **Published GWAS** | **Weighted median** | 1.16 | 0.81 | 1.67 | 0.420 |  |  |  |  |  |
| **Cancer** | **Ovarian cancer - high grade serous** | **Published GWAS** | **MR-Egger** | 1.18 | 0.74 | 1.87 | 0.489 | 0.00 | 0.733 | 69.02 | 0.544 | 0.96 |
| **Cancer** | **Ovarian cancer - high grade serous** | **Published GWAS** | **Penalised weighted median** | 1.17 | 0.81 | 1.68 | 0.414 |  |  |  |  |  |
| **Cancer** | **Ovarian cancer - invasive epithelial** | **Published GWAS** | **Inverse-variance weighted** | 1.24 | 1.08 | 1.44 | 0.004 |  |  | 72.70 | 0.455 |  |
| **Cancer** | **Ovarian cancer - invasive epithelial** | **Published GWAS** | **Weighted median** | 1.29 | 1.01 | 1.65 | 0.040 |  |  |  |  |  |
| **Cancer** | **Ovarian cancer - invasive epithelial** | **Published GWAS** | **MR-Egger** | 1.42 | 1.05 | 1.92 | 0.027 | 0.00 | 0.337 | 71.76 | 0.452 | 0.96 |
| **Cancer** | **Ovarian cancer - invasive epithelial** | **Published GWAS** | **Penalised weighted median** | 1.31 | 1.03 | 1.67 | 0.029 |  |  |  |  |  |
| **Cancer** | **Ovarian cancer - low grade** | **Published GWAS** | **Inverse-variance weighted** | 1.05 | 0.59 | 1.86 | 0.874 |  |  | 87.48 | 0.103 |  |
| **Cancer** | **Ovarian cancer - low grade** | **Published GWAS** | **Weighted median** | 1.10 | 0.47 | 2.56 | 0.830 |  |  |  |  |  |
| **Cancer** | **Ovarian cancer - low grade** | **Published GWAS** | **MR-Egger** | 1.22 | 0.36 | 4.10 | 0.755 | 0.00 | 0.787 | 87.39 | 0.091 | 0.96 |
| **Cancer** | **Ovarian cancer - low grade** | **Published GWAS** | **Penalised weighted median** | 1.14 | 0.49 | 2.67 | 0.755 |  |  |  |  |  |
| **Cancer** | **Ovarian cancer - mucinous ovarian carcinoma** | **Published GWAS** | **Inverse-variance weighted** | 1.24 | 0.88 | 1.74 | 0.227 |  |  | 78.65 | 0.276 |  |
| **Cancer** | **Ovarian cancer - mucinous ovarian carcinoma** | **Published GWAS** | **Weighted median** | 1.36 | 0.77 | 2.40 | 0.296 |  |  |  |  |  |
| **Cancer** | **Ovarian cancer - mucinous ovarian carcinoma** | **Published GWAS** | **MR-Egger** | 2.06 | 1.02 | 4.19 | 0.049 | -0.01 | 0.112 | 75.88 | 0.324 | 0.96 |
| **Cancer** | **Ovarian cancer - mucinous ovarian carcinoma** | **Published GWAS** | **Penalised weighted median** | 1.35 | 0.78 | 2.35 | 0.288 |  |  |  |  |  |
| **Cancer** | **Ovarian cancer - serous invasive** | **Published GWAS** | **Inverse-variance weighted** | 1.19 | 1.00 | 1.42 | 0.054 |  |  | 79.63 | 0.251 |  |
| **Cancer** | **Ovarian cancer - serous invasive** | **Published GWAS** | **Weighted median** | 1.16 | 0.87 | 1.54 | 0.322 |  |  |  |  |  |
| **Cancer** | **Ovarian cancer - serous invasive** | **Published GWAS** | **MR-Egger** | 1.18 | 0.82 | 1.71 | 0.381 | 0.00 | 0.964 | 79.63 | 0.226 | 0.96 |
| **Cancer** | **Ovarian cancer - serous invasive** | **Published GWAS** | **Penalised weighted median** | 1.16 | 0.86 | 1.56 | 0.336 |  |  |  |  |  |
| **Cancer** | **Ovarian cancer - serous lowgrade borderline** | **Published GWAS** | **Inverse-variance weighted** | 1.33 | 0.97 | 1.83 | 0.082 |  |  | 75.28 | 0.373 |  |
| **Cancer** | **Ovarian cancer - serous lowgrade borderline** | **Published GWAS** | **Weighted median** | 1.62 | 0.97 | 2.72 | 0.065 |  |  |  |  |  |
| **Cancer** | **Ovarian cancer - serous lowgrade borderline** | **Published GWAS** | **MR-Egger** | 1.56 | 0.80 | 3.07 | 0.198 | 0.00 | 0.598 | 74.99 | 0.350 | 0.96 |
| **Cancer** | **Ovarian cancer - serous lowgrade borderline** | **Published GWAS** | **Penalised weighted median** | 1.63 | 0.97 | 2.76 | 0.067 |  |  |  |  |  |
| **Cancer** | **Pancreatic cancer** | **FinnGen** | **Inverse-variance weighted** | 1.39 | 0.67 | 2.88 | 0.376 |  |  | 83.24 | 0.172 |  |
| **Cancer** | **Pancreatic cancer** | **FinnGen** | **Weighted median** | 1.17 | 0.36 | 3.78 | 0.794 |  |  |  |  |  |
| **Cancer** | **Pancreatic cancer** | **FinnGen** | **MR-Egger** | 2.79 | 0.61 | 12.75 | 0.191 | -0.02 | 0.311 | 82.04 | 0.174 | 0.96 |
| **Cancer** | **Pancreatic cancer** | **FinnGen** | **Penalised weighted median** | 1.18 | 0.38 | 3.66 | 0.780 |  |  |  |  |  |
| **Cancer** | **Prostate cancer** | **FinnGen** | **Inverse-variance weighted** | 0.91 | 0.67 | 1.24 | 0.566 |  |  | 105.87 | 0.006 |  |
| **Cancer** | **Prostate cancer** | **FinnGen** | **Weighted median** | 0.67 | 0.44 | 1.02 | 0.060 |  |  |  |  |  |
| **Cancer** | **Prostate cancer** | **FinnGen** | **MR-Egger** | 0.97 | 0.51 | 1.84 | 0.926 | 0.00 | 0.838 | 105.81 | 0.005 | 0.96 |
| **Cancer** | **Prostate cancer** | **FinnGen** | **Penalised weighted median** | 0.67 | 0.44 | 1.03 | 0.066 |  |  |  |  |  |
| **Cancer** | **Prostate cancer** | **Published GWAS** | **Inverse-variance weighted** | 0.91 | 0.80 | 1.04 | 0.172 |  |  | 158.17 | 2.E-08 |  |
| **Cancer** | **Prostate cancer** | **Published GWAS** | **Weighted median** | 0.81 | 0.69 | 0.95 | 0.010 |  |  |  |  |  |
| **Cancer** | **Prostate cancer** | **Published GWAS** | **MR-Egger** | 0.83 | 0.63 | 1.09 | 0.180 | 0.00 | 0.429 | 156.78 | 2.E-08 | 0.96 |
| **Cancer** | **Prostate cancer** | **Published GWAS** | **Penalised weighted median** | 0.81 | 0.68 | 0.96 | 0.014 |  |  |  |  |  |
| **Cancer** | **Renal cancer** | **FinnGen** | **Inverse-variance weighted** | 1.41 | 0.81 | 2.46 | 0.228 |  |  | 67.58 | 0.626 |  |
| **Cancer** | **Renal cancer** | **FinnGen** | **Weighted median** | 1.98 | 0.80 | 4.92 | 0.141 |  |  |  |  |  |
| **Cancer** | **Renal cancer** | **FinnGen** | **MR-Egger** | 1.74 | 0.54 | 5.59 | 0.353 | -0.01 | 0.680 | 67.42 | 0.599 | 0.96 |
| **Cancer** | **Renal cancer** | **FinnGen** | **Penalised weighted median** | 2.00 | 0.80 | 4.99 | 0.139 |  |  |  |  |  |
| **Cancer** | **Renal cancer** | **Published GWAS** | **Inverse-variance weighted** | 1.48 | 1.09 | 2.02 | 0.024 |  |  | 18.27 | 0.249 |  |
| **Cancer** | **Renal cancer** | **Published GWAS** | **Weighted median** | 1.61 | 1.08 | 2.39 | 0.019 |  |  |  |  |  |
| **Cancer** | **Renal cancer** | **Published GWAS** | **MR-Egger** | 1.34 | 0.61 | 2.97 | 0.480 | 0.00 | 0.791 | 18.17 | 0.199 | 0.98 |
| **Cancer** | **Renal cancer** | **Published GWAS** | **Penalised weighted median** | 1.55 | 0.95 | 2.51 | 0.079 |  |  |  |  |  |
| **Cancer** | **Thyroid cancer** | **FinnGen** | **Inverse-variance weighted** | 0.90 | 0.52 | 1.56 | 0.719 |  |  | 54.33 | 0.940 |  |
| **Cancer** | **Thyroid cancer** | **FinnGen** | **Weighted median** | 0.81 | 0.34 | 1.94 | 0.631 |  |  |  |  |  |
| **Cancer** | **Thyroid cancer** | **FinnGen** | **MR-Egger** | 0.66 | 0.21 | 2.08 | 0.483 | 0.01 | 0.489 | 53.97 | 0.934 | 0.96 |
| **Cancer** | **Thyroid cancer** | **FinnGen** | **Penalised weighted median** | 0.81 | 0.33 | 1.98 | 0.643 |  |  |  |  |  |

**Supplementary File 1gii. The inverse-variance weighted, weighted median, Egger and penalised weighted median MR analyses for body fat percentage using FinnGen and published GWAS. OR: odds ratio; LCI: lower 95% confidence interval; UCI: upper 95% confidence interval; P: p-value; Intercept P: intercept p-value; Q: Q-statistic; I^2^ MR-Egger: I^2^-statistic MR-Egger.**

|  | | | | | | | | | | **Heterogeneity** | | |
| --- | --- | --- | --- | --- | --- | --- | --- | --- | --- | --- | --- | --- |
| **System** | **Disease** | **Study** | **Analysis** | **OR** | **LCI** | **UCI** | **P** | **Egger intercept** | **Intercept P** | **Q** | **P** | **I^2^ MR-Egger** |
| **Cardiovascular and metabolic** | **Abdominal aortic aneurysm** | **FinnGen** | **Inverse-variance weighted** | 1.36 | 1.05 | 1.77 | 0.021 |  |  | 726.75 | 0.022 |  |
| **Cardiovascular and metabolic** | **Abdominal aortic aneurysm** | **FinnGen** | **Weighted median** | 1.45 | 0.97 | 2.14 | 0.067 |  |  |  |  |  |
| **Cardiovascular and metabolic** | **Abdominal aortic aneurysm** | **FinnGen** | **MR-Egger** | 2.38 | 1.03 | 5.50 | 0.043 | -0.01 | 0.170 | 724.65 | 0.023 | 0.95 |
| **Cardiovascular and metabolic** | **Abdominal aortic aneurysm** | **FinnGen** | **Penalised weighted median** | 1.39 | 0.92 | 2.10 | 0.119 |  |  |  |  |  |
| **Cardiovascular and metabolic** | **Abdominal aortic aneurysm** | **Published GWAS** | **Inverse-variance weighted** | 1.05 | 1.00 | 1.11 | 0.048 |  |  | 752.68 | 0.002 |  |
| **Cardiovascular and metabolic** | **Abdominal aortic aneurysm** | **Published GWAS** | **Weighted median** | 1.07 | 0.99 | 1.16 | 0.093 |  |  |  |  |  |
| **Cardiovascular and metabolic** | **Abdominal aortic aneurysm** | **Published GWAS** | **MR-Egger** | 1.10 | 0.96 | 1.26 | 0.181 | 0.00 | 0.520 | 752.20 | 0.002 | 0.80 |
| **Cardiovascular and metabolic** | **Abdominal aortic aneurysm** | **Published GWAS** | **Penalised weighted median** | 1.10 | 1.01 | 1.19 | 0.025 |  |  |  |  |  |
| **Cardiovascular and metabolic** | **Atrial fibrillation** | **FinnGen** | **Inverse-variance weighted** | 1.73 | 1.51 | 1.97 | 4.E-15 |  |  | 822.56 | 6.E-06 |  |
| **Cardiovascular and metabolic** | **Atrial fibrillation** | **FinnGen** | **Weighted median** | 1.78 | 1.48 | 2.15 | 1.E-09 |  |  |  |  |  |
| **Cardiovascular and metabolic** | **Atrial fibrillation** | **FinnGen** | **MR-Egger** | 2.04 | 1.33 | 3.13 | 0.001 | 0.00 | 0.415 | 821.72 | 6.E-06 | 0.96 |
| **Cardiovascular and metabolic** | **Atrial fibrillation** | **FinnGen** | **Penalised weighted median** | 1.72 | 1.43 | 2.08 | 2.E-08 |  |  |  |  |  |
| **Cardiovascular and metabolic** | **Atrial fibrillation** | **Published GWAS** | **Inverse-variance weighted** | 1.37 | 1.27 | 1.47 | 9.E-17 |  |  | 1188.60 | 2.E-34 |  |
| **Cardiovascular and metabolic** | **Atrial fibrillation** | **Published GWAS** | **Weighted median** | 1.43 | 1.32 | 1.56 | 3.E-17 |  |  |  |  |  |
| **Cardiovascular and metabolic** | **Atrial fibrillation** | **Published GWAS** | **MR-Egger** | 1.78 | 1.42 | 2.24 | 1.E-06 | 0.00 | 0.017 | 1178.20 | 2.E-33 | 0.73 |
| **Cardiovascular and metabolic** | **Atrial fibrillation** | **Published GWAS** | **Penalised weighted median** | 1.42 | 1.30 | 1.55 | 3.E-15 |  |  |  |  |  |
| **Cardiovascular and metabolic** | **Cardioembolic stroke** | **Published GWAS** | **Inverse-variance weighted** | 1.23 | 1.04 | 1.44 | 0.014 |  |  | 814.22 | 5.E-06 |  |
| **Cardiovascular and metabolic** | **Cardioembolic stroke** | **Published GWAS** | **Weighted median** | 1.28 | 1.01 | 1.63 | 0.041 |  |  |  |  |  |
| **Cardiovascular and metabolic** | **Cardioembolic stroke** | **Published GWAS** | **MR-Egger** | 1.15 | 0.68 | 1.95 | 0.595 | 0.00 | 0.813 | 814.15 | 4.E-06 | 0.75 |
| **Cardiovascular and metabolic** | **Cardioembolic stroke** | **Published GWAS** | **Penalised weighted median** | 1.29 | 1.02 | 1.63 | 0.035 |  |  |  |  |  |
| **Cardiovascular and metabolic** | **Cardiovascular disease** | **FinnGen** | **Inverse-variance weighted** | 1.55 | 1.43 | 1.68 | 3.E-25 |  |  | 1029.68 | 2.E-19 |  |
| **Cardiovascular and metabolic** | **Cardiovascular disease** | **FinnGen** | **Weighted median** | 1.71 | 1.54 | 1.89 | 1.E-25 |  |  |  |  |  |
| **Cardiovascular and metabolic** | **Cardiovascular disease** | **FinnGen** | **MR-Egger** | 1.65 | 1.28 | 2.12 | 1.E-04 | 0.00 | 0.631 | 1029.32 | 2.E-19 | 0.96 |
| **Cardiovascular and metabolic** | **Cardiovascular disease** | **FinnGen** | **Penalised weighted median** | 1.68 | 1.51 | 1.87 | 3.E-21 |  |  |  |  |  |
| **Cardiovascular and metabolic** | **Coronary artery disease** | **Published GWAS** | **Inverse-variance weighted** | 1.53 | 1.39 | 1.68 | 6.E-18 |  |  | 1151.55 | 1.E-31 |  |
| **Cardiovascular and metabolic** | **Coronary artery disease** | **Published GWAS** | **Weighted median** | 1.65 | 1.47 | 1.86 | 1.E-16 |  |  |  |  |  |
| **Cardiovascular and metabolic** | **Coronary artery disease** | **Published GWAS** | **MR-Egger** | 1.50 | 1.11 | 2.04 | 0.009 | 0.00 | 0.891 | 1151.52 | 1.E-31 | 0.77 |
| **Cardiovascular and metabolic** | **Coronary artery disease** | **Published GWAS** | **Penalised weighted median** | 1.60 | 1.41 | 1.81 | 7.E-14 |  |  |  |  |  |
| **Cardiovascular and metabolic** | **Deep vein thrombosis** | **FinnGen** | **Inverse-variance weighted** | 1.71 | 1.40 | 2.09 | 2.E-07 |  |  | 798.74 | 7.E-05 |  |
| **Cardiovascular and metabolic** | **Deep vein thrombosis** | **FinnGen** | **Weighted median** | 2.05 | 1.52 | 2.75 | 2.E-06 |  |  |  |  |  |
| **Cardiovascular and metabolic** | **Deep vein thrombosis** | **FinnGen** | **MR-Egger** | 2.62 | 1.38 | 4.97 | 0.003 | -0.01 | 0.169 | 796.42 | 8.E-05 | 0.95 |
| **Cardiovascular and metabolic** | **Deep vein thrombosis** | **FinnGen** | **Penalised weighted median** | 2.00 | 1.51 | 2.63 | 1.E-06 |  |  |  |  |  |
| **Cardiovascular and metabolic** | **Heart failure** | **FinnGen** | **Inverse-variance weighted** | 1.76 | 1.55 | 2.00 | 5.E-17 |  |  | 721.99 | 0.029 |  |
| **Cardiovascular and metabolic** | **Heart failure** | **FinnGen** | **Weighted median** | 1.94 | 1.60 | 2.36 | 3.E-11 |  |  |  |  |  |
| **Cardiovascular and metabolic** | **Heart failure** | **FinnGen** | **MR-Egger** | 1.81 | 1.19 | 2.73 | 0.005 | 0.00 | 0.900 | 721.97 | 0.028 | 0.95 |
| **Cardiovascular and metabolic** | **Heart failure** | **FinnGen** | **Penalised weighted median** | 1.87 | 1.54 | 2.29 | 7.E-10 |  |  |  |  |  |
| **Cardiovascular and metabolic** | **Heart failure** | **Published GWAS** | **Inverse-variance weighted** | 1.76 | 1.64 | 1.89 | 4.E-44 |  |  | 1011.28 | 9.E-19 |  |
| **Cardiovascular and metabolic** | **Heart failure** | **Published GWAS** | **Weighted median** | 1.76 | 1.60 | 1.95 | 6.E-29 |  |  |  |  |  |
| **Cardiovascular and metabolic** | **Heart failure** | **Published GWAS** | **MR-Egger** | 1.91 | 1.50 | 2.42 | 1.E-07 | 0.00 | 0.481 | 1010.49 | 8.E-19 | 0.77 |
| **Cardiovascular and metabolic** | **Heart failure** | **Published GWAS** | **Penalised weighted median** | 1.62 | 1.47 | 1.79 | 2.E-22 |  |  |  |  |  |
| **Cardiovascular and metabolic** | **Hypertension** | **FinnGen** | **Inverse-variance weighted** | 2.01 | 1.79 | 2.26 | 6.E-29 |  |  | 1297.56 | 4.E-45 |  |
| **Cardiovascular and metabolic** | **Hypertension** | **FinnGen** | **Weighted median** | 2.28 | 1.99 | 2.62 | 2.E-32 |  |  |  |  |  |
| **Cardiovascular and metabolic** | **Hypertension** | **FinnGen** | **MR-Egger** | 1.98 | 1.36 | 2.88 | 4.E-04 | 0.00 | 0.929 | 1297.54 | 3.E-45 | 0.96 |
| **Cardiovascular and metabolic** | **Hypertension** | **FinnGen** | **Penalised weighted median** | 2.01 | 1.75 | 2.30 | 2.E-23 |  |  |  |  |  |
| **Cardiovascular and metabolic** | **Ischemic heart disease** | **FinnGen** | **Inverse-variance weighted** | 1.27 | 1.14 | 1.40 | 1.E-05 |  |  | 904.57 | 2.E-10 |  |
| **Cardiovascular and metabolic** | **Ischemic heart disease** | **FinnGen** | **Weighted median** | 1.36 | 1.17 | 1.58 | 8.E-05 |  |  |  |  |  |
| **Cardiovascular and metabolic** | **Ischemic heart disease** | **FinnGen** | **MR-Egger** | 1.05 | 0.75 | 1.46 | 0.774 | 0.00 | 0.248 | 902.71 | 2.E-10 | 0.96 |
| **Cardiovascular and metabolic** | **Ischemic heart disease** | **FinnGen** | **Penalised weighted median** | 1.37 | 1.18 | 1.59 | 4.E-05 |  |  |  |  |  |
| **Cardiovascular and metabolic** | **Ischemic stroke** | **FinnGen** | **Inverse-variance weighted** | 1.22 | 1.06 | 1.39 | 0.004 |  |  | 775.98 | 6.E-04 |  |
| **Cardiovascular and metabolic** | **Ischemic stroke** | **FinnGen** | **Weighted median** | 1.29 | 1.05 | 1.57 | 0.013 |  |  |  |  |  |
| **Cardiovascular and metabolic** | **Ischemic stroke** | **FinnGen** | **MR-Egger** | 1.65 | 1.07 | 2.54 | 0.023 | 0.00 | 0.145 | 773.44 | 6.E-04 | 0.96 |
| **Cardiovascular and metabolic** | **Ischemic stroke** | **FinnGen** | **Penalised weighted median** | 1.28 | 1.04 | 1.58 | 0.019 |  |  |  |  |  |
| **Cardiovascular and metabolic** | **Ischemic stroke** | **Published GWAS** | **Inverse-variance weighted** | 1.24 | 1.13 | 1.35 | 2.E-06 |  |  | 886.42 | 5.E-10 |  |
| **Cardiovascular and metabolic** | **Ischemic stroke** | **Published GWAS** | **Weighted median** | 1.22 | 1.07 | 1.39 | 0.004 |  |  |  |  |  |
| **Cardiovascular and metabolic** | **Ischemic stroke** | **Published GWAS** | **MR-Egger** | 1.04 | 0.79 | 1.38 | 0.763 | 0.00 | 0.217 | 884.31 | 6.E-10 | 0.74 |
| **Cardiovascular and metabolic** | **Ischemic stroke** | **Published GWAS** | **Penalised weighted median** | 1.22 | 1.07 | 1.39 | 0.003 |  |  |  |  |  |
| **Cardiovascular and metabolic** | **Large artery stroke** | **Published GWAS** | **Inverse-variance weighted** | 1.37 | 1.11 | 1.69 | 0.004 |  |  | 848.02 | 1.E-07 |  |
| **Cardiovascular and metabolic** | **Large artery stroke** | **Published GWAS** | **Weighted median** | 1.62 | 1.19 | 2.21 | 0.002 |  |  |  |  |  |
| **Cardiovascular and metabolic** | **Large artery stroke** | **Published GWAS** | **MR-Egger** | 1.28 | 0.64 | 2.54 | 0.485 | 0.00 | 0.838 | 847.97 | 9.E-08 | 0.75 |
| **Cardiovascular and metabolic** | **Large artery stroke** | **Published GWAS** | **Penalised weighted median** | 1.66 | 1.23 | 2.26 | 0.001 |  |  |  |  |  |
| **Cardiovascular and metabolic** | **Peripheral artery disease** | **FinnGen** | **Inverse-variance weighted** | 1.80 | 1.52 | 2.14 | 4.E-11 |  |  | 806.01 | 3.E-05 |  |
| **Cardiovascular and metabolic** | **Peripheral artery disease** | **FinnGen** | **Weighted median** | 1.77 | 1.36 | 2.31 | 2.E-05 |  |  |  |  |  |
| **Cardiovascular and metabolic** | **Peripheral artery disease** | **FinnGen** | **MR-Egger** | 2.33 | 1.35 | 4.04 | 0.003 | 0.00 | 0.332 | 804.84 | 3.E-05 | 0.95 |
| **Cardiovascular and metabolic** | **Peripheral artery disease** | **FinnGen** | **Penalised weighted median** | 1.77 | 1.37 | 2.29 | 1.E-05 |  |  |  |  |  |
| **Cardiovascular and metabolic** | **Pulmonary embolism** | **FinnGen** | **Inverse-variance weighted** | 1.26 | 1.02 | 1.55 | 0.032 |  |  | 751.01 | 0.004 |  |
| **Cardiovascular and metabolic** | **Pulmonary embolism** | **FinnGen** | **Weighted median** | 1.32 | 0.95 | 1.85 | 0.100 |  |  |  |  |  |
| **Cardiovascular and metabolic** | **Pulmonary embolism** | **FinnGen** | **MR-Egger** | 1.96 | 1.00 | 3.85 | 0.050 | -0.01 | 0.174 | 748.88 | 0.005 | 0.95 |
| **Cardiovascular and metabolic** | **Pulmonary embolism** | **FinnGen** | **Penalised weighted median** | 1.33 | 0.96 | 1.82 | 0.084 |  |  |  |  |  |
| **Cardiovascular and metabolic** | **Small vessel stroke** | **Published GWAS** | **Inverse-variance weighted** | 1.26 | 1.05 | 1.51 | 0.015 |  |  | 752.76 | 0.002 |  |
| **Cardiovascular and metabolic** | **Small vessel stroke** | **Published GWAS** | **Weighted median** | 1.45 | 1.08 | 1.93 | 0.012 |  |  |  |  |  |
| **Cardiovascular and metabolic** | **Small vessel stroke** | **Published GWAS** | **MR-Egger** | 0.81 | 0.45 | 1.48 | 0.496 | 0.01 | 0.133 | 750.12 | 0.002 | 0.74 |
| **Cardiovascular and metabolic** | **Small vessel stroke** | **Published GWAS** | **Penalised weighted median** | 1.44 | 1.09 | 1.92 | 0.012 |  |  |  |  |  |
| **Cardiovascular and metabolic** | **Stroke** | **FinnGen** | **Inverse-variance weighted** | 1.24 | 1.10 | 1.39 | 3.E-04 |  |  | 790.88 | 1.E-04 |  |
| **Cardiovascular and metabolic** | **Stroke** | **FinnGen** | **Weighted median** | 1.17 | 0.97 | 1.42 | 0.094 |  |  |  |  |  |
| **Cardiovascular and metabolic** | **Stroke** | **FinnGen** | **MR-Egger** | 1.39 | 0.96 | 2.01 | 0.080 | 0.00 | 0.521 | 790.38 | 1.E-04 | 0.96 |
| **Cardiovascular and metabolic** | **Stroke** | **FinnGen** | **Penalised weighted median** | 1.17 | 0.98 | 1.41 | 0.082 |  |  |  |  |  |
| **Cardiovascular and metabolic** | **Stroke** | **Published GWAS** | **Inverse-variance weighted** | 1.22 | 1.13 | 1.32 | 9.E-07 |  |  | 847.87 | 9.E-08 |  |
| **Cardiovascular and metabolic** | **Stroke** | **Published GWAS** | **Weighted median** | 1.19 | 1.05 | 1.34 | 0.006 |  |  |  |  |  |
| **Cardiovascular and metabolic** | **Stroke** | **Published GWAS** | **MR-Egger** | 1.09 | 0.84 | 1.40 | 0.520 | 0.00 | 0.351 | 846.73 | 9.E-08 | 0.74 |
| **Cardiovascular and metabolic** | **Stroke** | **Published GWAS** | **Penalised weighted median** | 1.20 | 1.06 | 1.35 | 0.004 |  |  |  |  |  |
| **Cardiovascular and metabolic** | **Stroke (excl. subarachnoid hemorrhage)** | **FinnGen** | **Inverse-variance weighted** | 1.22 | 1.07 | 1.38 | 0.003 |  |  | 777.02 | 5.E-04 |  |
| **Cardiovascular and metabolic** | **Stroke (excl. subarachnoid hemorrhage)** | **FinnGen** | **Weighted median** | 1.20 | 0.99 | 1.46 | 0.068 |  |  |  |  |  |
| **Cardiovascular and metabolic** | **Stroke (excl. subarachnoid hemorrhage)** | **FinnGen** | **MR-Egger** | 1.80 | 1.19 | 2.71 | 0.005 | -0.01 | 0.051 | 772.50 | 7.E-04 | 0.96 |
| **Cardiovascular and metabolic** | **Stroke (excl. subarachnoid hemorrhage)** | **FinnGen** | **Penalised weighted median** | 1.19 | 0.97 | 1.46 | 0.098 |  |  |  |  |  |
| **Cardiovascular and metabolic** | **Venous thromboembolism** | **FinnGen** | **Inverse-variance weighted** | 1.48 | 1.27 | 1.72 | 6.E-07 |  |  | 854.70 | 1.E-07 |  |
| **Cardiovascular and metabolic** | **Venous thromboembolism** | **FinnGen** | **Weighted median** | 1.77 | 1.43 | 2.20 | 2.E-07 |  |  |  |  |  |
| **Cardiovascular and metabolic** | **Venous thromboembolism** | **FinnGen** | **MR-Egger** | 2.26 | 1.39 | 3.68 | 0.001 | -0.01 | 0.070 | 850.38 | 2.E-07 | 0.95 |
| **Cardiovascular and metabolic** | **Venous thromboembolism** | **FinnGen** | **Penalised weighted median** | 1.77 | 1.44 | 2.19 | 1.E-07 |  |  |  |  |  |
| **Cardiovascular and metabolic** | **Venous thromboembolism** | **Published GWAS** | **Inverse-variance weighted** | 1.66 | 1.44 | 1.93 | 3.E-11 |  |  | 1156.97 | 2.E-30 |  |
| **Cardiovascular and metabolic** | **Venous thromboembolism** | **Published GWAS** | **Weighted median** | 1.61 | 1.34 | 1.93 | 3.E-07 |  |  |  |  |  |
| **Cardiovascular and metabolic** | **Venous thromboembolism** | **Published GWAS** | **MR-Egger** | 1.70 | 1.05 | 2.75 | 0.032 | 0.00 | 0.934 | 1156.96 | 1.E-30 | 0.83 |
| **Cardiovascular and metabolic** | **Venous thromboembolism** | **Published GWAS** | **Penalised weighted median** | 1.60 | 1.34 | 1.91 | 2.E-07 |  |  |  |  |  |
| **Cardiovascular and metabolic** | **Polycystic ovary syndrome** | **FinnGen** | **Inverse-variance weighted** | 2.67 | 1.62 | 4.40 | 1.E-04 |  |  | 685.65 | 0.175 |  |
| **Cardiovascular and metabolic** | **Polycystic ovary syndrome** | **FinnGen** | **Weighted median** | 2.13 | 0.95 | 4.75 | 0.065 |  |  |  |  |  |
| **Cardiovascular and metabolic** | **Polycystic ovary syndrome** | **FinnGen** | **MR-Egger** | 4.90 | 0.98 | 24.44 | 0.053 | -0.01 | 0.434 | 685.01 | 0.172 | 0.96 |
| **Cardiovascular and metabolic** | **Polycystic ovary syndrome** | **FinnGen** | **Penalised weighted median** | 2.10 | 0.93 | 4.76 | 0.075 |  |  |  |  |  |
| **Cardiovascular and metabolic** | **Polycystic ovary syndrome** | **Published GWAS** | **Inverse-variance weighted** | 2.63 | 2.05 | 3.37 | 6.E-14 |  |  | 739.75 | 0.007 |  |
| **Cardiovascular and metabolic** | **Polycystic ovary syndrome** | **Published GWAS** | **Weighted median** | 2.55 | 1.75 | 3.71 | 1.E-06 |  |  |  |  |  |
| **Cardiovascular and metabolic** | **Polycystic ovary syndrome** | **Published GWAS** | **MR-Egger** | 4.93 | 2.21 | 11.03 | 1.E-04 | -0.01 | 0.108 | 736.80 | 0.008 | 0.77 |
| **Cardiovascular and metabolic** | **Polycystic ovary syndrome** | **Published GWAS** | **Penalised weighted median** | 2.25 | 1.54 | 3.27 | 3.E-05 |  |  |  |  |  |
| **Cardiovascular and metabolic** | **Type 2 diabetes** | **FinnGen** | **Inverse-variance weighted** | 2.24 | 1.92 | 2.61 | 7.E-23 |  |  | 2089.76 | 5.E-150 |  |
| **Cardiovascular and metabolic** | **Type 2 diabetes** | **FinnGen** | **Weighted median** | 2.80 | 2.40 | 3.27 | 5.E-39 |  |  |  |  |  |
| **Cardiovascular and metabolic** | **Type 2 diabetes** | **FinnGen** | **MR-Egger** | 2.86 | 1.74 | 4.69 | 4.E-05 | 0.00 | 0.308 | 2086.42 | 9.E-150 | 0.96 |
| **Cardiovascular and metabolic** | **Type 2 diabetes** | **FinnGen** | **Penalised weighted median** | 2.32 | 2.00 | 2.68 | 2.E-29 |  |  |  |  |  |
| **Cardiovascular and metabolic** | **Type 2 diabetes** | **Published GWAS** | **Inverse-variance weighted** | 2.52 | 2.18 | 2.91 | 3.E-32 |  |  | 3647.58 | 0.E+00 |  |
| **Cardiovascular and metabolic** | **Type 2 diabetes** | **Published GWAS** | **Weighted median** | 3.14 | 2.81 | 3.52 | 2.E-89 |  |  |  |  |  |
| **Cardiovascular and metabolic** | **Type 2 diabetes** | **Published GWAS** | **MR-Egger** | 2.44 | 1.54 | 3.87 | 2.E-04 | 0.00 | 0.885 | 3647.46 | 0.E+00 | 0.72 |
| **Cardiovascular and metabolic** | **Type 2 diabetes** | **Published GWAS** | **Penalised weighted median** | 2.11 | 1.88 | 2.38 | 6.E-36 |  |  |  |  |  |
| **Cardiovascular and metabolic** | **Chronic kidney disease** | **FinnGen** | **Inverse-variance weighted** | 1.45 | 1.16 | 1.80 | 0.001 |  |  | 768.85 | 0.001 |  |
| **Cardiovascular and metabolic** | **Chronic kidney disease** | **FinnGen** | **Weighted median** | 1.43 | 1.03 | 1.98 | 0.033 |  |  |  |  |  |
| **Cardiovascular and metabolic** | **Chronic kidney disease** | **FinnGen** | **MR-Egger** | 0.80 | 0.39 | 1.61 | 0.524 | 0.01 | 0.080 | 765.23 | 0.001 | 0.95 |
| **Cardiovascular and metabolic** | **Chronic kidney disease** | **FinnGen** | **Penalised weighted median** | 1.45 | 1.06 | 1.99 | 0.022 |  |  |  |  |  |
| **Cardiovascular and metabolic** | **Chronic kidney disease** | **Published GWAS** | **Inverse-variance weighted** | 1.13 | 1.03 | 1.23 | 0.007 |  |  | 977.27 | 3.E-16 |  |
| **Cardiovascular and metabolic** | **Chronic kidney disease** | **Published GWAS** | **Weighted median** | 1.19 | 1.06 | 1.34 | 0.003 |  |  |  |  |  |
| **Cardiovascular and metabolic** | **Chronic kidney disease** | **Published GWAS** | **MR-Egger** | 0.94 | 0.71 | 1.23 | 0.648 | 0.00 | 0.170 | 974.40 | 4.E-16 | 0.74 |
| **Cardiovascular and metabolic** | **Chronic kidney disease** | **Published GWAS** | **Penalised weighted median** | 1.22 | 1.08 | 1.37 | 9.E-04 |  |  |  |  |  |
| **Musculoskeletal** | **Gout** | **FinnGen** | **Inverse-variance weighted** | 1.49 | 1.18 | 1.88 | 9.E-04 |  |  | 757.77 | 0.003 |  |
| **Musculoskeletal** | **Gout** | **FinnGen** | **Weighted median** | 1.55 | 1.08 | 2.23 | 0.017 |  |  |  |  |  |
| **Musculoskeletal** | **Gout** | **FinnGen** | **MR-Egger** | 1.28 | 0.61 | 2.70 | 0.518 | 0.00 | 0.678 | 757.57 | 0.002 | 0.95 |
| **Musculoskeletal** | **Gout** | **FinnGen** | **Penalised weighted median** | 1.49 | 1.04 | 2.15 | 0.030 |  |  |  |  |  |
| **Musculoskeletal** | **Gout** | **Published GWAS** | **Inverse-variance weighted** | 1.73 | 1.52 | 1.96 | 8.E-17 |  |  | 1071.14 | 4.E-22 |  |
| **Musculoskeletal** | **Gout** | **Published GWAS** | **Weighted median** | 1.89 | 1.60 | 2.24 | 1.E-13 |  |  |  |  |  |
| **Musculoskeletal** | **Gout** | **Published GWAS** | **MR-Egger** | 1.49 | 1.00 | 2.21 | 0.048 | 0.00 | 0.442 | 1070.18 | 3.E-22 | 0.91 |
| **Musculoskeletal** | **Gout** | **Published GWAS** | **Penalised weighted median** | 1.73 | 1.48 | 2.03 | 6.E-12 |  |  |  |  |  |
| **Musculoskeletal** | **Osteoarthritis** | **FinnGen** | **Inverse-variance weighted** | 1.81 | 1.65 | 1.98 | 3.E-32 |  |  | 907.56 | 1.E-10 |  |
| **Musculoskeletal** | **Osteoarthritis** | **FinnGen** | **Weighted median** | 1.78 | 1.56 | 2.02 | 8.E-19 |  |  |  |  |  |
| **Musculoskeletal** | **Osteoarthritis** | **FinnGen** | **MR-Egger** | 2.55 | 1.90 | 3.43 | 1.E-09 | 0.00 | 0.016 | 899.52 | 3.E-10 | 0.96 |
| **Musculoskeletal** | **Osteoarthritis** | **FinnGen** | **Penalised weighted median** | 1.60 | 1.41 | 1.81 | 3.E-13 |  |  |  |  |  |
| **Musculoskeletal** | **Osteoarthritis** | **Published GWAS** | **Inverse-variance weighted** | 1.83 | 1.72 | 1.94 | 1.E-71 |  |  | 1577.67 | 2.E-70 |  |
| **Musculoskeletal** | **Osteoarthritis** | **Published GWAS** | **Weighted median** | 1.88 | 1.75 | 2.02 | 2.E-68 |  |  |  |  |  |
| **Musculoskeletal** | **Osteoarthritis** | **Published GWAS** | **MR-Egger** | 1.93 | 1.60 | 2.33 | 2.E-11 | 0.00 | 0.544 | 1576.83 | 2.E-70 | 0.71 |
| **Musculoskeletal** | **Osteoarthritis** | **Published GWAS** | **Penalised weighted median** | 1.69 | 1.58 | 1.81 | 3.E-52 |  |  |  |  |  |
| **Musculoskeletal** | **Osteoarthritis (hip and/or knee)** | **Published GWAS** | **Inverse-variance weighted** | 2.08 | 1.93 | 2.25 | 5.E-64 |  |  | 1400.76 | 7.E-50 |  |
| **Musculoskeletal** | **Osteoarthritis (hip and/or knee)** | **Published GWAS** | **Weighted median** | 2.14 | 1.96 | 2.34 | 5.E-61 |  |  |  |  |  |
| **Musculoskeletal** | **Osteoarthritis (hip and/or knee)** | **Published GWAS** | **MR-Egger** | 2.12 | 1.66 | 2.72 | 3.E-09 | 0.00 | 0.864 | 1400.71 | 5.E-50 | 0.71 |
| **Musculoskeletal** | **Osteoarthritis (hip and/or knee)** | **Published GWAS** | **Penalised weighted median** | 1.77 | 1.62 | 1.94 | 6.E-34 |  |  |  |  |  |
| **Musculoskeletal** | **Osteoarthritis (hip)** | **FinnGen** | **Inverse-variance weighted** | 1.65 | 1.41 | 1.92 | 3.E-10 |  |  | 917.09 | 3.E-11 |  |
| **Musculoskeletal** | **Osteoarthritis (hip)** | **FinnGen** | **Weighted median** | 1.76 | 1.44 | 2.15 | 4.E-08 |  |  |  |  |  |
| **Musculoskeletal** | **Osteoarthritis (hip)** | **FinnGen** | **MR-Egger** | 2.31 | 1.41 | 3.78 | 9.E-04 | 0.00 | 0.156 | 914.25 | 4.E-11 | 0.96 |
| **Musculoskeletal** | **Osteoarthritis (hip)** | **FinnGen** | **Penalised weighted median** | 1.52 | 1.24 | 1.87 | 6.E-05 |  |  |  |  |  |
| **Musculoskeletal** | **Osteoarthritis (knee)** | **FinnGen** | **Inverse-variance weighted** | 2.17 | 1.94 | 2.42 | 5.E-38 |  |  | 912.15 | 6.E-11 |  |
| **Musculoskeletal** | **Osteoarthritis (knee)** | **FinnGen** | **Weighted median** | 2.12 | 1.83 | 2.45 | 4.E-24 |  |  |  |  |  |
| **Musculoskeletal** | **Osteoarthritis (knee)** | **FinnGen** | **MR-Egger** | 3.72 | 2.62 | 5.29 | 6.E-13 | -0.01 | 0.002 | 898.18 | 4.E-10 | 0.96 |
| **Musculoskeletal** | **Osteoarthritis (knee)** | **FinnGen** | **Penalised weighted median** | 1.88 | 1.62 | 2.18 | 1.E-16 |  |  |  |  |  |
| **Musculoskeletal** | **Osteoporosis** | **FinnGen** | **Inverse-variance weighted** | 1.02 | 0.82 | 1.26 | 0.893 |  |  | 660.63 | 0.399 |  |
| **Musculoskeletal** | **Osteoporosis** | **FinnGen** | **Weighted median** | 0.86 | 0.61 | 1.22 | 0.401 |  |  |  |  |  |
| **Musculoskeletal** | **Osteoporosis** | **FinnGen** | **MR-Egger** | 1.17 | 0.58 | 2.37 | 0.657 | 0.00 | 0.672 | 660.45 | 0.390 | 0.96 |
| **Musculoskeletal** | **Osteoporosis** | **FinnGen** | **Penalised weighted median** | 0.86 | 0.62 | 1.20 | 0.380 |  |  |  |  |  |
| **Musculoskeletal** | **Osteoporosis** | **Published GWAS** | **Inverse-variance weighted** | 1.16 | 1.11 | 1.21 | 5.E-11 |  |  | 6533.58 | 0.E+00 |  |
| **Musculoskeletal** | **Osteoporosis** | **Published GWAS** | **Weighted median** | 1.17 | 1.14 | 1.21 | 9.E-27 |  |  |  |  |  |
| **Musculoskeletal** | **Osteoporosis** | **Published GWAS** | **MR-Egger** | 1.25 | 1.09 | 1.43 | 0.002 | 0.00 | 0.268 | 6520.91 | 0.E+00 | 0.72 |
| **Musculoskeletal** | **Osteoporosis** | **Published GWAS** | **Penalised weighted median** | 1.17 | 1.14 | 1.21 | 1.E-22 |  |  |  |  |  |
| **Musculoskeletal** | **Rheumatoid arthritis** | **FinnGen** | **Inverse-variance weighted** | 1.56 | 1.30 | 1.87 | 1.E-06 |  |  | 790.09 | 2.E-04 |  |
| **Musculoskeletal** | **Rheumatoid arthritis** | **FinnGen** | **Weighted median** | 1.55 | 1.19 | 2.03 | 0.001 |  |  |  |  |  |
| **Musculoskeletal** | **Rheumatoid arthritis** | **FinnGen** | **MR-Egger** | 2.05 | 1.15 | 3.65 | 0.015 | 0.00 | 0.326 | 788.92 | 2.E-04 | 0.96 |
| **Musculoskeletal** | **Rheumatoid arthritis** | **FinnGen** | **Penalised weighted median** | 1.53 | 1.16 | 2.01 | 0.002 |  |  |  |  |  |
| **Musculoskeletal** | **Rheumatoid arthritis** | **Published GWAS** | **Inverse-variance weighted** | 1.40 | 1.15 | 1.71 | 1.E-03 |  |  | 1282.48 | 6.E-48 |  |
| **Musculoskeletal** | **Rheumatoid arthritis** | **Published GWAS** | **Weighted median** | 1.00 | 0.80 | 1.26 | 1.000 |  |  |  |  |  |
| **Musculoskeletal** | **Rheumatoid arthritis** | **Published GWAS** | **MR-Egger** | 1.36 | 0.72 | 2.58 | 0.343 | 0.00 | 0.933 | 1282.46 | 4.E-48 | 0.84 |
| **Musculoskeletal** | **Rheumatoid arthritis** | **Published GWAS** | **Penalised weighted median** | 1.00 | 0.79 | 1.27 | 1.000 |  |  |  |  |  |
| **Gastrointestinal** | **Gallstones** | **FinnGen** | **Inverse-variance weighted** | 1.99 | 1.78 | 2.22 | 1.E-30 |  |  | 844.14 | 5.E-07 |  |
| **Gastrointestinal** | **Gallstones** | **FinnGen** | **Weighted median** | 2.00 | 1.71 | 2.34 | 4.E-18 |  |  |  |  |  |
| **Gastrointestinal** | **Gallstones** | **FinnGen** | **MR-Egger** | 3.09 | 2.17 | 4.41 | 7.E-10 | -0.01 | 0.010 | 835.58 | 1.E-06 | 0.96 |
| **Gastrointestinal** | **Gallstones** | **FinnGen** | **Penalised weighted median** | 1.91 | 1.63 | 2.23 | 4.E-16 |  |  |  |  |  |
| **Gastrointestinal** | **Gastro-oesophageal reflux disease** | **Published GWAS** | **Inverse-variance weighted** | 1.80 | 1.69 | 1.91 | 2.E-63 |  |  | 1522.46 | 3.E-64 |  |
| **Gastrointestinal** | **Gastro-oesophageal reflux disease** | **Published GWAS** | **Weighted median** | 1.70 | 1.58 | 1.83 | 1.E-44 |  |  |  |  |  |
| **Gastrointestinal** | **Gastro-oesophageal reflux disease** | **Published GWAS** | **MR-Egger** | 1.41 | 1.16 | 1.72 | 7.E-04 | 0.00 | 0.012 | 1508.52 | 8.E-63 | 0.71 |
| **Gastrointestinal** | **Gastro-oesophageal reflux disease** | **Published GWAS** | **Penalised weighted median** | 1.47 | 1.37 | 1.58 | 2.E-24 |  |  |  |  |  |
| **Nervous** | **Alzheimer's disease** | **FinnGen** | **Inverse-variance weighted** | 0.97 | 0.74 | 1.28 | 0.843 |  |  | 1143.85 | 2.E-29 |  |
| **Nervous** | **Alzheimer's disease** | **FinnGen** | **Weighted median** | 1.01 | 0.72 | 1.41 | 0.973 |  |  |  |  |  |
| **Nervous** | **Alzheimer's disease** | **FinnGen** | **MR-Egger** | 0.93 | 0.38 | 2.25 | 0.872 | 0.00 | 0.917 | 1143.83 | 1.E-29 | 0.95 |
| **Nervous** | **Alzheimer's disease** | **FinnGen** | **Penalised weighted median** | 1.01 | 0.73 | 1.40 | 0.937 |  |  |  |  |  |
| **Nervous** | **Alzheimer's disease** | **Published GWAS (1)** | **Inverse-variance weighted** | 0.99 | 0.96 | 1.03 | 0.714 |  |  | 3646.97 | 0.E+00 |  |
| **Nervous** | **Alzheimer's disease** | **Published GWAS (1)** | **Weighted median** | 1.03 | 1.00 | 1.06 | 0.026 |  |  |  |  |  |
| **Nervous** | **Alzheimer's disease** | **Published GWAS (1)** | **MR-Egger** | 0.95 | 0.84 | 1.07 | 0.414 | 0.00 | 0.460 | 3643.92 | 0.E+00 | 0.96 |
| **Nervous** | **Alzheimer's disease** | **Published GWAS (1)** | **Penalised weighted median** | 1.06 | 1.03 | 1.09 | 1.E-04 |  |  |  |  |  |
| **Nervous** | **Alzheimer's disease** | **Published GWAS (2)** | **Inverse-variance weighted** | 0.75 | 0.59 | 0.95 | 0.019 |  |  | 3401.61 | 0.E+00 |  |
| **Nervous** | **Alzheimer's disease** | **Published GWAS (2)** | **Weighted median** | 0.84 | 0.71 | 1.01 | 0.058 |  |  |  |  |  |
| **Nervous** | **Alzheimer's disease** | **Published GWAS (2)** | **MR-Egger** | 0.38 | 0.17 | 0.83 | 0.015 | 0.01 | 0.073 | 3384.71 | 0.E+00 | 0.75 |
| **Nervous** | **Alzheimer's disease** | **Published GWAS (2)** | **Penalised weighted median** | 0.92 | 0.77 | 1.10 | 0.359 |  |  |  |  |  |
| **Nervous** | **Depression** | **FinnGen** | **Inverse-variance weighted** | 1.14 | 1.03 | 1.26 | 0.009 |  |  | 814.82 | 1.E-05 |  |
| **Nervous** | **Depression** | **FinnGen** | **Weighted median** | 1.19 | 1.02 | 1.38 | 0.024 |  |  |  |  |  |
| **Nervous** | **Depression** | **FinnGen** | **MR-Egger** | 0.91 | 0.66 | 1.25 | 0.551 | 0.00 | 0.138 | 812.06 | 2.E-05 | 0.96 |
| **Nervous** | **Depression** | **FinnGen** | **Penalised weighted median** | 1.19 | 1.02 | 1.39 | 0.031 |  |  |  |  |  |
| **Nervous** | **Depression** | **Published GWAS** | **Inverse-variance weighted** | 1.23 | 1.13 | 1.34 | 1.E-06 |  |  | 1011.76 | 2.E-18 |  |
| **Nervous** | **Depression** | **Published GWAS** | **Weighted median** | 1.33 | 1.18 | 1.49 | 2.E-06 |  |  |  |  |  |
| **Nervous** | **Depression** | **Published GWAS** | **MR-Egger** | 1.22 | 0.93 | 1.58 | 0.147 | 0.00 | 0.927 | 1011.74 | 1.E-18 | 0.83 |
| **Nervous** | **Depression** | **Published GWAS** | **Penalised weighted median** | 1.35 | 1.21 | 1.52 | 2.E-07 |  |  |  |  |  |
| **Nervous** | **Multiple sclerosis** | **FinnGen** | **Inverse-variance weighted** | 0.85 | 0.58 | 1.25 | 0.407 |  |  | 695.88 | 0.114 |  |
| **Nervous** | **Multiple sclerosis** | **FinnGen** | **Weighted median** | 0.56 | 0.31 | 1.02 | 0.059 |  |  |  |  |  |
| **Nervous** | **Multiple sclerosis** | **FinnGen** | **MR-Egger** | 0.44 | 0.13 | 1.49 | 0.186 | 0.01 | 0.262 | 694.54 | 0.115 | 0.96 |
| **Nervous** | **Multiple sclerosis** | **FinnGen** | **Penalised weighted median** | 0.60 | 0.33 | 1.08 | 0.087 |  |  |  |  |  |
| **Nervous** | **Parkinson's disease** | **FinnGen** | **Inverse-variance weighted** | 1.07 | 0.81 | 1.42 | 0.630 |  |  | 672.41 | 0.282 |  |
| **Nervous** | **Parkinson's disease** | **FinnGen** | **Weighted median** | 1.29 | 0.84 | 1.97 | 0.246 |  |  |  |  |  |
| **Nervous** | **Parkinson's disease** | **FinnGen** | **MR-Egger** | 0.84 | 0.35 | 2.06 | 0.710 | 0.00 | 0.582 | 672.10 | 0.275 | 0.96 |
| **Nervous** | **Parkinson's disease** | **FinnGen** | **Penalised weighted median** | 1.32 | 0.83 | 2.08 | 0.240 |  |  |  |  |  |
| **Nervous** | **Parkinson's disease** | **Published GWAS** | **Inverse-variance weighted** | 0.76 | 0.65 | 0.89 | 9.E-04 |  |  | 854.49 | 8.E-08 |  |
| **Nervous** | **Parkinson's disease** | **Published GWAS** | **Weighted median** | 0.84 | 0.65 | 1.08 | 0.176 |  |  |  |  |  |
| **Nervous** | **Parkinson's disease** | **Published GWAS** | **MR-Egger** | 0.71 | 0.44 | 1.17 | 0.180 | 0.00 | 0.789 | 854.39 | 7.E-08 | 0.81 |
| **Nervous** | **Parkinson's disease** | **Published GWAS** | **Penalised weighted median** | 0.88 | 0.70 | 1.12 | 0.296 |  |  |  |  |  |
| **Integumentary** | **Psoriasis** | **FinnGen** | **Inverse-variance weighted** | 1.72 | 1.41 | 2.09 | 8.E-08 |  |  | 716.06 | 0.041 |  |
| **Integumentary** | **Psoriasis** | **FinnGen** | **Weighted median** | 1.86 | 1.38 | 2.52 | 6.E-05 |  |  |  |  |  |
| **Integumentary** | **Psoriasis** | **FinnGen** | **MR-Egger** | 1.63 | 0.87 | 3.05 | 0.126 | 0.00 | 0.864 | 716.03 | 0.039 | 0.96 |
| **Integumentary** | **Psoriasis** | **FinnGen** | **Penalised weighted median** | 1.86 | 1.38 | 2.51 | 5.E-05 |  |  |  |  |  |
| **Integumentary** | **Psoriasis** | **Published GWAS** | **Inverse-variance weighted** | 1.85 | 1.50 | 2.29 | 2.E-08 |  |  | 1025.76 | 4.E-32 |  |
| **Integumentary** | **Psoriasis** | **Published GWAS** | **Weighted median** | 1.76 | 1.35 | 2.29 | 2.E-05 |  |  |  |  |  |
| **Integumentary** | **Psoriasis** | **Published GWAS** | **MR-Egger** | 2.26 | 1.18 | 4.35 | 0.015 | 0.00 | 0.527 | 1025.00 | 3.E-32 | 0.84 |
| **Integumentary** | **Psoriasis** | **Published GWAS** | **Penalised weighted median** | 1.50 | 1.16 | 1.94 | 0.002 |  |  |  |  |  |
| **Respiratory** | **Adult-onset asthma** | **Published GWAS** | **Inverse-variance weighted** | 1.34 | 1.23 | 1.45 | 3.E-12 |  |  | 1212.22 | 3.E-34 |  |
| **Respiratory** | **Adult-onset asthma** | **Published GWAS** | **Weighted median** | 1.30 | 1.17 | 1.45 | 1.E-06 |  |  |  |  |  |
| **Respiratory** | **Adult-onset asthma** | **Published GWAS** | **MR-Egger** | 1.13 | 0.88 | 1.47 | 0.341 | 0.00 | 0.185 | 1209.02 | 4.E-34 | 0.71 |
| **Respiratory** | **Adult-onset asthma** | **Published GWAS** | **Penalised weighted median** | 1.30 | 1.16 | 1.44 | 2.E-06 |  |  |  |  |  |
| **Respiratory** | **Asthma** | **FinnGen** | **Inverse-variance weighted** | 1.54 | 1.38 | 1.71 | 2.E-14 |  |  | 843.92 | 5.E-07 |  |
| **Respiratory** | **Asthma** | **FinnGen** | **Weighted median** | 1.55 | 1.33 | 1.81 | 1.E-08 |  |  |  |  |  |
| **Respiratory** | **Asthma** | **FinnGen** | **MR-Egger** | 1.48 | 1.05 | 2.09 | 0.025 | 0.00 | 0.832 | 843.86 | 5.E-07 | 0.96 |
| **Respiratory** | **Asthma** | **FinnGen** | **Penalised weighted median** | 1.50 | 1.30 | 1.75 | 9.E-08 |  |  |  |  |  |
| **Respiratory** | **Child-onset asthma** | **Published GWAS** | **Inverse-variance weighted** | 0.97 | 0.86 | 1.09 | 0.603 |  |  | 1437.46 | 1.E-58 |  |
| **Respiratory** | **Child-onset asthma** | **Published GWAS** | **Weighted median** | 0.96 | 0.83 | 1.12 | 0.620 |  |  |  |  |  |
| **Respiratory** | **Child-onset asthma** | **Published GWAS** | **MR-Egger** | 1.08 | 0.73 | 1.60 | 0.695 | 0.00 | 0.560 | 1436.73 | 9.E-59 | 0.71 |
| **Respiratory** | **Child-onset asthma** | **Published GWAS** | **Penalised weighted median** | 1.02 | 0.88 | 1.19 | 0.780 |  |  |  |  |  |
| **Cancer** | **Barrett's oesophagus** | **FinnGen** | **Inverse-variance weighted** | 1.42 | 0.79 | 2.54 | 0.242 |  |  | 666.89 | 0.334 |  |
| **Cancer** | **Barrett's oesophagus** | **FinnGen** | **Weighted median** | 1.77 | 0.72 | 4.34 | 0.216 |  |  |  |  |  |
| **Cancer** | **Barrett's oesophagus** | **FinnGen** | **MR-Egger** | 2.99 | 0.46 | 19.29 | 0.251 | -0.01 | 0.410 | 666.20 | 0.331 | 0.96 |
| **Cancer** | **Barrett's oesophagus** | **FinnGen** | **Penalised weighted median** | 1.82 | 0.72 | 4.63 | 0.207 |  |  |  |  |  |
| **Cancer** | **Breast cancer** | **FinnGen** | **Inverse-variance weighted** | 0.77 | 0.65 | 0.90 | 0.001 |  |  | 794.57 | 1.E-04 |  |
| **Cancer** | **Breast cancer** | **FinnGen** | **Weighted median** | 0.75 | 0.59 | 0.93 | 0.010 |  |  |  |  |  |
| **Cancer** | **Breast cancer** | **FinnGen** | **MR-Egger** | 0.55 | 0.33 | 0.92 | 0.023 | 0.00 | 0.178 | 792.35 | 1.E-04 | 0.96 |
| **Cancer** | **Breast cancer** | **FinnGen** | **Penalised weighted median** | 0.78 | 0.62 | 0.98 | 0.032 |  |  |  |  |  |
| **Cancer** | **Breast cancer** | **Published GWAS** | **Inverse-variance weighted** | 0.91 | 0.78 | 1.06 | 0.217 |  |  | 1015.51 | 3.E-15 |  |
| **Cancer** | **Breast cancer** | **Published GWAS** | **Weighted median** | 0.87 | 0.71 | 1.07 | 0.180 |  |  |  |  |  |
| **Cancer** | **Breast cancer** | **Published GWAS** | **MR-Egger** | 0.52 | 0.31 | 0.87 | 0.012 | 0.01 | 0.025 | 1008.07 | 9.E-15 | 0.76 |
| **Cancer** | **Breast cancer** | **Published GWAS** | **Penalised weighted median** | 1.05 | 0.85 | 1.28 | 0.662 |  |  |  |  |  |
| **Cancer** | **Cancer myeloma** | **FinnGen** | **Inverse-variance weighted** | 1.43 | 0.85 | 2.39 | 0.179 |  |  | 675.50 | 0.254 |  |
| **Cancer** | **Cancer myeloma** | **FinnGen** | **Weighted median** | 1.70 | 0.70 | 4.10 | 0.239 |  |  |  |  |  |
| **Cancer** | **Cancer myeloma** | **FinnGen** | **MR-Egger** | 2.67 | 0.51 | 14.03 | 0.247 | -0.01 | 0.437 | 674.87 | 0.251 | 0.96 |
| **Cancer** | **Cancer myeloma** | **FinnGen** | **Penalised weighted median** | 1.70 | 0.74 | 3.89 | 0.211 |  |  |  |  |  |
| **Cancer** | **Colorectal cancer** | **FinnGen** | **Inverse-variance weighted** | 1.07 | 0.85 | 1.35 | 0.573 |  |  | 767.38 | 0.001 |  |
| **Cancer** | **Colorectal cancer** | **FinnGen** | **Weighted median** | 1.00 | 0.70 | 1.44 | 0.998 |  |  |  |  |  |
| **Cancer** | **Colorectal cancer** | **FinnGen** | **MR-Egger** | 1.14 | 0.54 | 2.42 | 0.734 | 0.00 | 0.863 | 767.34 | 0.001 | 0.96 |
| **Cancer** | **Colorectal cancer** | **FinnGen** | **Penalised weighted median** | 1.00 | 0.70 | 1.43 | 0.995 |  |  |  |  |  |
| **Cancer** | **Colorectal cancer** | **Published GWAS (1)** | **Inverse-variance weighted** | 0.86 | 0.68 | 1.08 | 0.204 |  |  | 149.54 | 3.E-07 |  |
| **Cancer** | **Colorectal cancer** | **Published GWAS (1)** | **Weighted median** | 0.90 | 0.68 | 1.20 | 0.466 |  |  |  |  |  |
| **Cancer** | **Colorectal cancer** | **Published GWAS (1)** | **MR-Egger** | 1.33 | 0.69 | 2.57 | 0.403 | -0.01 | 0.171 | 145.67 | 6.E-07 | 0.93 |
| **Cancer** | **Colorectal cancer** | **Published GWAS (1)** | **Penalised weighted median** | 0.93 | 0.69 | 1.25 | 0.623 |  |  |  |  |  |
| **Cancer** | **Endometrial cancer** | **FinnGen** | **Inverse-variance weighted** | 1.04 | 0.72 | 1.49 | 0.829 |  |  | 668.75 | 0.316 |  |
| **Cancer** | **Endometrial cancer** | **FinnGen** | **Weighted median** | 0.79 | 0.45 | 1.40 | 0.425 |  |  |  |  |  |
| **Cancer** | **Endometrial cancer** | **FinnGen** | **MR-Egger** | 1.77 | 0.56 | 5.65 | 0.334 | -0.01 | 0.344 | 667.83 | 0.315 | 0.96 |
| **Cancer** | **Endometrial cancer** | **FinnGen** | **Penalised weighted median** | 0.81 | 0.44 | 1.48 | 0.490 |  |  |  |  |  |
| **Cancer** | **Endometrial cancer** | **Published GWAS** | **Inverse-variance weighted** | 1.68 | 1.47 | 1.91 | 6.E-14 |  |  | 969.90 | 9.E-13 |  |
| **Cancer** | **Endometrial cancer** | **Published GWAS** | **Weighted median** | 1.66 | 1.39 | 1.99 | 4.E-08 |  |  |  |  |  |
| **Cancer** | **Endometrial cancer** | **Published GWAS** | **MR-Egger** | 1.75 | 1.14 | 2.70 | 0.011 | 0.00 | 0.828 | 969.83 | 7.E-13 | 0.73 |
| **Cancer** | **Endometrial cancer** | **Published GWAS** | **Penalised weighted median** | 1.61 | 1.35 | 1.92 | 1.E-07 |  |  |  |  |  |
| **Cancer** | **Lung cancer** | **FinnGen** | **Inverse-variance weighted** | 1.33 | 1.00 | 1.77 | 0.051 |  |  | 654.14 | 0.469 |  |
| **Cancer** | **Lung cancer** | **FinnGen** | **Weighted median** | 1.24 | 0.79 | 1.94 | 0.354 |  |  |  |  |  |
| **Cancer** | **Lung cancer** | **FinnGen** | **MR-Egger** | 1.54 | 0.62 | 3.85 | 0.353 | 0.00 | 0.737 | 654.03 | 0.459 | 0.96 |
| **Cancer** | **Lung cancer** | **FinnGen** | **Penalised weighted median** | 1.16 | 0.74 | 1.80 | 0.518 |  |  |  |  |  |
| **Cancer** | **Meningioma** | **FinnGen** | **Inverse-variance weighted** | 1.01 | 0.71 | 1.43 | 0.967 |  |  | 678.44 | 0.229 |  |
| **Cancer** | **Meningioma** | **FinnGen** | **Weighted median** | 0.58 | 0.33 | 1.01 | 0.052 |  |  |  |  |  |
| **Cancer** | **Meningioma** | **FinnGen** | **MR-Egger** | 0.39 | 0.13 | 1.21 | 0.105 | 0.01 | 0.085 | 675.36 | 0.247 | 0.96 |
| **Cancer** | **Meningioma** | **FinnGen** | **Penalised weighted median** | 0.62 | 0.35 | 1.07 | 0.088 |  |  |  |  |  |
| **Cancer** | **Ovarian cancer** | **FinnGen** | **Inverse-variance weighted** | 0.86 | 0.55 | 1.33 | 0.485 |  |  | 689.51 | 0.150 |  |
| **Cancer** | **Ovarian cancer** | **FinnGen** | **Weighted median** | 0.91 | 0.44 | 1.85 | 0.789 |  |  |  |  |  |
| **Cancer** | **Ovarian cancer** | **FinnGen** | **MR-Egger** | 0.73 | 0.18 | 2.99 | 0.664 | 0.00 | 0.819 | 689.45 | 0.144 | 0.96 |
| **Cancer** | **Ovarian cancer** | **FinnGen** | **Penalised weighted median** | 0.97 | 0.48 | 1.96 | 0.939 |  |  |  |  |  |
| **Cancer** | **Ovarian cancer - clear cell cancer** | **Published GWAS** | **Inverse-variance weighted** | 0.95 | 0.71 | 1.26 | 0.708 |  |  | 673.06 | 0.651 |  |
| **Cancer** | **Ovarian cancer - clear cell cancer** | **Published GWAS** | **Weighted median** | 0.95 | 0.62 | 1.46 | 0.810 |  |  |  |  |  |
| **Cancer** | **Ovarian cancer - clear cell cancer** | **Published GWAS** | **MR-Egger** | 1.83 | 0.72 | 4.63 | 0.204 | -0.01 | 0.141 | 670.93 | 0.662 | 0.79 |
| **Cancer** | **Ovarian cancer - clear cell cancer** | **Published GWAS** | **Penalised weighted median** | 0.94 | 0.62 | 1.44 | 0.781 |  |  |  |  |  |
| **Cancer** | **Ovarian cancer - endometrioid** | **Published GWAS** | **Inverse-variance weighted** | 1.21 | 0.97 | 1.51 | 0.087 |  |  | 792.07 | 0.004 |  |
| **Cancer** | **Ovarian cancer - endometrioid** | **Published GWAS** | **Weighted median** | 1.29 | 0.91 | 1.83 | 0.154 |  |  |  |  |  |
| **Cancer** | **Ovarian cancer - endometrioid** | **Published GWAS** | **MR-Egger** | 1.60 | 0.79 | 3.27 | 0.195 | 0.00 | 0.419 | 791.32 | 0.003 | 0.78 |
| **Cancer** | **Ovarian cancer - endometrioid** | **Published GWAS** | **Penalised weighted median** | 1.32 | 0.93 | 1.87 | 0.125 |  |  |  |  |  |
| **Cancer** | **Ovarian cancer - epithelial** | **Published GWAS** | **Inverse-variance weighted** | 1.45 | 1.18 | 1.79 | 4.E-04 |  |  | 727.75 | 0.142 |  |
| **Cancer** | **Ovarian cancer - epithelial** | **Published GWAS** | **Weighted median** | 1.41 | 1.02 | 1.93 | 0.036 |  |  |  |  |  |
| **Cancer** | **Ovarian cancer - epithelial** | **Published GWAS** | **MR-Egger** | 1.26 | 0.64 | 2.47 | 0.506 | 0.00 | 0.659 | 727.54 | 0.138 | 0.95 |
| **Cancer** | **Ovarian cancer - epithelial** | **Published GWAS** | **Penalised weighted median** | 1.34 | 0.96 | 1.87 | 0.082 |  |  |  |  |  |
| **Cancer** | **Ovarian cancer - high grade serous** | **Published GWAS** | **Inverse-variance weighted** | 1.13 | 0.98 | 1.32 | 0.100 |  |  | 736.54 | 0.097 |  |
| **Cancer** | **Ovarian cancer - high grade serous** | **Published GWAS** | **Weighted median** | 1.23 | 0.97 | 1.57 | 0.082 |  |  |  |  |  |
| **Cancer** | **Ovarian cancer - high grade serous** | **Published GWAS** | **MR-Egger** | 0.97 | 0.60 | 1.58 | 0.905 | 0.00 | 0.509 | 736.07 | 0.095 | 0.81 |
| **Cancer** | **Ovarian cancer - high grade serous** | **Published GWAS** | **Penalised weighted median** | 1.24 | 0.97 | 1.57 | 0.082 |  |  |  |  |  |
| **Cancer** | **Ovarian cancer - invasive epithelial** | **Published GWAS** | **Inverse-variance weighted** | 1.20 | 1.07 | 1.33 | 0.001 |  |  | 900.65 | 8.E-08 |  |
| **Cancer** | **Ovarian cancer - invasive epithelial** | **Published GWAS** | **Weighted median** | 1.24 | 1.07 | 1.44 | 0.005 |  |  |  |  |  |
| **Cancer** | **Ovarian cancer - invasive epithelial** | **Published GWAS** | **MR-Egger** | 1.44 | 1.02 | 2.05 | 0.041 | 0.00 | 0.271 | 899.06 | 8.E-08 | 0.79 |
| **Cancer** | **Ovarian cancer - invasive epithelial** | **Published GWAS** | **Penalised weighted median** | 1.23 | 1.06 | 1.44 | 0.007 |  |  |  |  |  |
| **Cancer** | **Ovarian cancer - low grade** | **Published GWAS** | **Inverse-variance weighted** | 1.10 | 0.77 | 1.57 | 0.593 |  |  | 743.90 | 0.069 |  |
| **Cancer** | **Ovarian cancer - low grade** | **Published GWAS** | **Weighted median** | 0.98 | 0.57 | 1.68 | 0.928 |  |  |  |  |  |
| **Cancer** | **Ovarian cancer - low grade** | **Published GWAS** | **MR-Egger** | 1.12 | 0.35 | 3.58 | 0.844 | 0.00 | 0.972 | 743.90 | 0.065 | 0.90 |
| **Cancer** | **Ovarian cancer - low grade** | **Published GWAS** | **Penalised weighted median** | 0.97 | 0.56 | 1.69 | 0.927 |  |  |  |  |  |
| **Cancer** | **Ovarian cancer - mucinous ovarian carcinoma** | **Published GWAS** | **Inverse-variance weighted** | 1.50 | 1.21 | 1.87 | 2.E-04 |  |  | 708.77 | 0.284 |  |
| **Cancer** | **Ovarian cancer - mucinous ovarian carcinoma** | **Published GWAS** | **Weighted median** | 1.54 | 1.09 | 2.17 | 0.013 |  |  |  |  |  |
| **Cancer** | **Ovarian cancer - mucinous ovarian carcinoma** | **Published GWAS** | **MR-Egger** | 2.09 | 1.03 | 4.25 | 0.041 | 0.00 | 0.335 | 707.81 | 0.283 | 0.81 |
| **Cancer** | **Ovarian cancer - mucinous ovarian carcinoma** | **Published GWAS** | **Penalised weighted median** | 1.53 | 1.08 | 2.17 | 0.017 |  |  |  |  |  |
| **Cancer** | **Ovarian cancer - serous invasive** | **Published GWAS** | **Inverse-variance weighted** | 1.18 | 1.04 | 1.34 | 0.009 |  |  | 887.36 | 4.E-07 |  |
| **Cancer** | **Ovarian cancer - serous invasive** | **Published GWAS** | **Weighted median** | 1.14 | 0.94 | 1.38 | 0.191 |  |  |  |  |  |
| **Cancer** | **Ovarian cancer - serous invasive** | **Published GWAS** | **MR-Egger** | 1.20 | 0.80 | 1.79 | 0.379 | 0.00 | 0.942 | 887.35 | 3.E-07 | 0.79 |
| **Cancer** | **Ovarian cancer - serous invasive** | **Published GWAS** | **Penalised weighted median** | 1.14 | 0.95 | 1.37 | 0.166 |  |  |  |  |  |
| **Cancer** | **Ovarian cancer - serous lowgrade borderline** | **Published GWAS** | **Inverse-variance weighted** | 1.28 | 1.03 | 1.60 | 0.028 |  |  | 804.71 | 0.001 |  |
| **Cancer** | **Ovarian cancer - serous lowgrade borderline** | **Published GWAS** | **Weighted median** | 1.45 | 1.05 | 1.99 | 0.022 |  |  |  |  |  |
| **Cancer** | **Ovarian cancer - serous lowgrade borderline** | **Published GWAS** | **MR-Egger** | 1.41 | 0.68 | 2.89 | 0.355 | 0.00 | 0.794 | 804.63 | 0.001 | 0.93 |
| **Cancer** | **Ovarian cancer - serous lowgrade borderline** | **Published GWAS** | **Penalised weighted median** | 1.48 | 1.05 | 2.08 | 0.025 |  |  |  |  |  |
| **Cancer** | **Pancreatic cancer** | **FinnGen** | **Inverse-variance weighted** | 1.25 | 0.79 | 1.97 | 0.345 |  |  | 637.92 | 0.646 |  |
| **Cancer** | **Pancreatic cancer** | **FinnGen** | **Weighted median** | 1.25 | 0.59 | 2.66 | 0.556 |  |  |  |  |  |
| **Cancer** | **Pancreatic cancer** | **FinnGen** | **MR-Egger** | 6.70 | 1.55 | 29.06 | 0.011 | -0.02 | 0.017 | 632.32 | 0.693 | 0.95 |
| **Cancer** | **Pancreatic cancer** | **FinnGen** | **Penalised weighted median** | 1.22 | 0.58 | 2.54 | 0.603 |  |  |  |  |  |
| **Cancer** | **Prostate cancer** | **FinnGen** | **Inverse-variance weighted** | 0.89 | 0.75 | 1.07 | 0.217 |  |  | 698.87 | 0.099 |  |
| **Cancer** | **Prostate cancer** | **FinnGen** | **Weighted median** | 0.86 | 0.66 | 1.12 | 0.260 |  |  |  |  |  |
| **Cancer** | **Prostate cancer** | **FinnGen** | **MR-Egger** | 0.91 | 0.52 | 1.60 | 0.742 | 0.00 | 0.953 | 698.87 | 0.094 | 0.95 |
| **Cancer** | **Prostate cancer** | **FinnGen** | **Penalised weighted median** | 0.90 | 0.69 | 1.16 | 0.413 |  |  |  |  |  |
| **Cancer** | **Prostate cancer** | **Published GWAS** | **Inverse-variance weighted** | 0.90 | 0.82 | 0.98 | 0.013 |  |  | 1497.67 | 3.E-62 |  |
| **Cancer** | **Prostate cancer** | **Published GWAS** | **Weighted median** | 0.88 | 0.80 | 0.96 | 0.006 |  |  |  |  |  |
| **Cancer** | **Prostate cancer** | **Published GWAS** | **MR-Egger** | 0.87 | 0.66 | 1.15 | 0.334 | 0.00 | 0.828 | 1497.56 | 2.E-62 | 0.81 |
| **Cancer** | **Prostate cancer** | **Published GWAS** | **Penalised weighted median** | 1.02 | 0.93 | 1.12 | 0.704 |  |  |  |  |  |
| **Cancer** | **Renal cancer** | **FinnGen** | **Inverse-variance weighted** | 1.14 | 0.78 | 1.67 | 0.485 |  |  | 645.14 | 0.568 |  |
| **Cancer** | **Renal cancer** | **FinnGen** | **Weighted median** | 0.98 | 0.53 | 1.80 | 0.936 |  |  |  |  |  |
| **Cancer** | **Renal cancer** | **FinnGen** | **MR-Egger** | 0.92 | 0.27 | 3.08 | 0.892 | 0.00 | 0.709 | 645.00 | 0.559 | 0.95 |
| **Cancer** | **Renal cancer** | **FinnGen** | **Penalised weighted median** | 0.98 | 0.53 | 1.79 | 0.938 |  |  |  |  |  |
| **Cancer** | **Renal cancer** | **Published GWAS** | **Inverse-variance weighted** | 1.31 | 0.91 | 1.89 | 0.151 |  |  | 117.65 | 2.E-04 |  |
| **Cancer** | **Renal cancer** | **Published GWAS** | **Weighted median** | 1.35 | 0.88 | 2.07 | 0.171 |  |  |  |  |  |
| **Cancer** | **Renal cancer** | **Published GWAS** | **MR-Egger** | 2.87 | 1.02 | 8.02 | 0.049 | -0.01 | 0.116 | 113.42 | 5.E-04 | 0.93 |
| **Cancer** | **Renal cancer** | **Published GWAS** | **Penalised weighted median** | 1.35 | 0.85 | 2.15 | 0.207 |  |  |  |  |  |
| **Cancer** | **Thyroid cancer** | **FinnGen** | **Inverse-variance weighted** | 0.73 | 0.51 | 1.06 | 0.103 |  |  | 655.75 | 0.451 |  |
| **Cancer** | **Thyroid cancer** | **FinnGen** | **Weighted median** | 0.67 | 0.36 | 1.24 | 0.200 |  |  |  |  |  |
| **Cancer** | **Thyroid cancer** | **FinnGen** | **MR-Egger** | 1.47 | 0.45 | 4.82 | 0.528 | -0.01 | 0.230 | 654.30 | 0.456 | 0.96 |
| **Cancer** | **Thyroid cancer** | **FinnGen** | **Penalised weighted median** | 0.70 | 0.39 | 1.29 | 0.255 |  |  |  |  |  |

**Supplementary File 1giii. The inverse-variance weighted, weighted median, Egger and penalised weighted median MR analyses for "favourable adiposity" using FinnGen and published GWAS. OR: odds ratio; LCI: lower 95% confidence interval; UCI: upper 95% confidence interval; P: p-value; Intercept P: intercept p-value; Q: Q-statistic; I^2^ MR-Egger: I^2^-statistic MR-Egger.**

|  | | | | | | | | | | **Heterogeneity** | | |
| --- | --- | --- | --- | --- | --- | --- | --- | --- | --- | --- | --- | --- |
| **System** | **Disease** | **Study** | **Analysis** | **OR** | **LCI** | **UCI** | **P** | **Egger intercept** | **Intercept P** | **Q** | **P** | **I^2^ MR-Egger** |
| **Cardiovascular and metabolic** | **Abdominal aortic aneurysm** | **FinnGen** | **Inverse-variance weighted** | 0.47 | 0.17 | 1.30 | 0.156 |  |  | 45.56 | 0.109 |  |
| **Cardiovascular and metabolic** | **Abdominal aortic aneurysm** | **FinnGen** | **Weighted median** | 0.27 | 0.08 | 0.96 | 0.043 |  |  |  |  |  |
| **Cardiovascular and metabolic** | **Abdominal aortic aneurysm** | **FinnGen** | **MR-Egger** | 2.49 | 0.12 | 53.57 | 0.565 | -0.02 | 0.271 | 43.94 | 0.118 | 0.84 |
| **Cardiovascular and metabolic** | **Abdominal aortic aneurysm** | **FinnGen** | **Penalised weighted median** | 0.24 | 0.06 | 0.89 | 0.034 |  |  |  |  |  |
| **Cardiovascular and metabolic** | **Abdominal aortic aneurysm** | **Published GWAS** | **Inverse-variance weighted** | 0.98 | 0.78 | 1.23 | 0.845 |  |  | 55.17 | 0.016 |  |
| **Cardiovascular and metabolic** | **Abdominal aortic aneurysm** | **Published GWAS** | **Weighted median** | 1.13 | 0.84 | 1.51 | 0.426 |  |  |  |  |  |
| **Cardiovascular and metabolic** | **Abdominal aortic aneurysm** | **Published GWAS** | **MR-Egger** | 1.76 | 0.94 | 3.27 | 0.084 | -0.01 | 0.056 | 49.45 | 0.042 | 0.86 |
| **Cardiovascular and metabolic** | **Abdominal aortic aneurysm** | **Published GWAS** | **Penalised weighted median** | 1.06 | 0.76 | 1.49 | 0.721 |  |  |  |  |  |
| **Cardiovascular and metabolic** | **Atrial fibrillation** | **FinnGen** | **Inverse-variance weighted** | 0.53 | 0.31 | 0.91 | 0.027 |  |  | 56.41 | 0.012 |  |
| **Cardiovascular and metabolic** | **Atrial fibrillation** | **FinnGen** | **Weighted median** | 0.58 | 0.30 | 1.09 | 0.092 |  |  |  |  |  |
| **Cardiovascular and metabolic** | **Atrial fibrillation** | **FinnGen** | **MR-Egger** | 0.82 | 0.16 | 4.27 | 0.813 | -0.01 | 0.594 | 55.93 | 0.010 | 0.84 |
| **Cardiovascular and metabolic** | **Atrial fibrillation** | **FinnGen** | **Penalised weighted median** | 0.62 | 0.33 | 1.18 | 0.143 |  |  |  |  |  |
| **Cardiovascular and metabolic** | **Atrial fibrillation** | **Published GWAS** | **Inverse-variance weighted** | 1.10 | 0.78 | 1.56 | 0.576 |  |  | 114.27 | 2.E-10 |  |
| **Cardiovascular and metabolic** | **Atrial fibrillation** | **Published GWAS** | **Weighted median** | 1.13 | 0.83 | 1.54 | 0.434 |  |  |  |  |  |
| **Cardiovascular and metabolic** | **Atrial fibrillation** | **Published GWAS** | **MR-Egger** | 0.83 | 0.27 | 2.55 | 0.750 | 0.00 | 0.605 | 113.37 | 2.E-10 | 0.82 |
| **Cardiovascular and metabolic** | **Atrial fibrillation** | **Published GWAS** | **Penalised weighted median** | 1.21 | 0.87 | 1.67 | 0.264 |  |  |  |  |  |
| **Cardiovascular and metabolic** | **Cardioembolic stroke** | **Published GWAS** | **Inverse-variance weighted** | 0.73 | 0.43 | 1.24 | 0.249 |  |  | 35.42 | 0.449 |  |
| **Cardiovascular and metabolic** | **Cardioembolic stroke** | **Published GWAS** | **Weighted median** | 0.98 | 0.46 | 2.11 | 0.966 |  |  |  |  |  |
| **Cardiovascular and metabolic** | **Cardioembolic stroke** | **Published GWAS** | **MR-Egger** | 0.54 | 0.09 | 3.07 | 0.489 | 0.00 | 0.723 | 35.28 | 0.407 | 0.82 |
| **Cardiovascular and metabolic** | **Cardioembolic stroke** | **Published GWAS** | **Penalised weighted median** | 1.07 | 0.50 | 2.32 | 0.859 |  |  |  |  |  |
| **Cardiovascular and metabolic** | **Cardiovascular disease** | **FinnGen** | **Inverse-variance weighted** | 0.58 | 0.44 | 0.77 | 6.E-04 |  |  | 53.98 | 0.021 |  |
| **Cardiovascular and metabolic** | **Cardiovascular disease** | **FinnGen** | **Weighted median** | 0.59 | 0.42 | 0.83 | 0.002 |  |  |  |  |  |
| **Cardiovascular and metabolic** | **Cardiovascular disease** | **FinnGen** | **MR-Egger** | 0.93 | 0.39 | 2.17 | 0.859 | -0.01 | 0.267 | 52.03 | 0.025 | 0.83 |
| **Cardiovascular and metabolic** | **Cardiovascular disease** | **FinnGen** | **Penalised weighted median** | 0.66 | 0.46 | 0.96 | 0.029 |  |  |  |  |  |
| **Cardiovascular and metabolic** | **Coronary artery disease** | **Published GWAS** | **Inverse-variance weighted** | 0.34 | 0.22 | 0.54 | 4.E-05 |  |  | 100.84 | 3.E-08 |  |
| **Cardiovascular and metabolic** | **Coronary artery disease** | **Published GWAS** | **Weighted median** | 0.27 | 0.17 | 0.43 | 3.E-08 |  |  |  |  |  |
| **Cardiovascular and metabolic** | **Coronary artery disease** | **Published GWAS** | **MR-Egger** | 1.95 | 0.50 | 7.53 | 0.341 | -0.03 | 0.012 | 83.58 | 5.E-06 | 0.86 |
| **Cardiovascular and metabolic** | **Coronary artery disease** | **Published GWAS** | **Penalised weighted median** | 0.59 | 0.35 | 0.98 | 0.040 |  |  |  |  |  |
| **Cardiovascular and metabolic** | **Deep vein thrombosis** | **FinnGen** | **Inverse-variance weighted** | 3.45 | 1.81 | 6.57 | 6.E-04 |  |  | 34.15 | 0.509 |  |
| **Cardiovascular and metabolic** | **Deep vein thrombosis** | **FinnGen** | **Weighted median** | 3.80 | 1.48 | 9.76 | 0.005 |  |  |  |  |  |
| **Cardiovascular and metabolic** | **Deep vein thrombosis** | **FinnGen** | **MR-Egger** | 0.84 | 0.12 | 6.01 | 0.864 | 0.02 | 0.134 | 31.93 | 0.569 | 0.84 |
| **Cardiovascular and metabolic** | **Deep vein thrombosis** | **FinnGen** | **Penalised weighted median** | 3.23 | 1.29 | 8.11 | 0.012 |  |  |  |  |  |
| **Cardiovascular and metabolic** | **Heart failure** | **FinnGen** | **Inverse-variance weighted** | 0.76 | 0.49 | 1.17 | 0.224 |  |  | 34.00 | 0.516 |  |
| **Cardiovascular and metabolic** | **Heart failure** | **FinnGen** | **Weighted median** | 0.70 | 0.36 | 1.36 | 0.288 |  |  |  |  |  |
| **Cardiovascular and metabolic** | **Heart failure** | **FinnGen** | **MR-Egger** | 0.79 | 0.21 | 2.98 | 0.726 | 0.00 | 0.956 | 33.99 | 0.468 | 0.84 |
| **Cardiovascular and metabolic** | **Heart failure** | **FinnGen** | **Penalised weighted median** | 0.70 | 0.37 | 1.33 | 0.279 |  |  |  |  |  |
| **Cardiovascular and metabolic** | **Heart failure** | **Published GWAS** | **Inverse-variance weighted** | 0.91 | 0.65 | 1.28 | 0.601 |  |  | 91.97 | 5.E-07 |  |
| **Cardiovascular and metabolic** | **Heart failure** | **Published GWAS** | **Weighted median** | 0.87 | 0.61 | 1.23 | 0.425 |  |  |  |  |  |
| **Cardiovascular and metabolic** | **Heart failure** | **Published GWAS** | **MR-Egger** | 1.56 | 0.53 | 4.58 | 0.424 | -0.01 | 0.310 | 89.18 | 8.E-07 | 0.85 |
| **Cardiovascular and metabolic** | **Heart failure** | **Published GWAS** | **Penalised weighted median** | 0.85 | 0.59 | 1.24 | 0.408 |  |  |  |  |  |
| **Cardiovascular and metabolic** | **Hypertension** | **FinnGen** | **Inverse-variance weighted** | 0.34 | 0.21 | 0.55 | 1.E-04 |  |  | 92.39 | 5.E-07 |  |
| **Cardiovascular and metabolic** | **Hypertension** | **FinnGen** | **Weighted median** | 0.33 | 0.21 | 0.53 | 3.E-06 |  |  |  |  |  |
| **Cardiovascular and metabolic** | **Hypertension** | **FinnGen** | **MR-Egger** | 0.75 | 0.17 | 3.25 | 0.706 | -0.01 | 0.269 | 89.09 | 8.E-07 | 0.83 |
| **Cardiovascular and metabolic** | **Hypertension** | **FinnGen** | **Penalised weighted median** | 0.33 | 0.20 | 0.56 | 3.E-05 |  |  |  |  |  |
| **Cardiovascular and metabolic** | **Ischemic heart disease** | **FinnGen** | **Inverse-variance weighted** | 0.34 | 0.22 | 0.53 | 2.E-05 |  |  | 65.35 | 0.001 |  |
| **Cardiovascular and metabolic** | **Ischemic heart disease** | **FinnGen** | **Weighted median** | 0.28 | 0.17 | 0.46 | 8.E-07 |  |  |  |  |  |
| **Cardiovascular and metabolic** | **Ischemic heart disease** | **FinnGen** | **MR-Egger** | 0.88 | 0.24 | 3.19 | 0.843 | -0.01 | 0.142 | 61.27 | 0.003 | 0.84 |
| **Cardiovascular and metabolic** | **Ischemic heart disease** | **FinnGen** | **Penalised weighted median** | 0.53 | 0.31 | 0.90 | 0.020 |  |  |  |  |  |
| **Cardiovascular and metabolic** | **Ischemic stroke** | **FinnGen** | **Inverse-variance weighted** | 0.65 | 0.38 | 1.10 | 0.121 |  |  | 50.37 | 0.045 |  |
| **Cardiovascular and metabolic** | **Ischemic stroke** | **FinnGen** | **Weighted median** | 0.50 | 0.26 | 0.95 | 0.035 |  |  |  |  |  |
| **Cardiovascular and metabolic** | **Ischemic stroke** | **FinnGen** | **MR-Egger** | 1.05 | 0.21 | 5.37 | 0.949 | -0.01 | 0.543 | 49.81 | 0.039 | 0.84 |
| **Cardiovascular and metabolic** | **Ischemic stroke** | **FinnGen** | **Penalised weighted median** | 0.49 | 0.25 | 0.97 | 0.040 |  |  |  |  |  |
| **Cardiovascular and metabolic** | **Ischemic stroke** | **Published GWAS** | **Inverse-variance weighted** | 0.70 | 0.50 | 0.97 | 0.042 |  |  | 51.54 | 0.035 |  |
| **Cardiovascular and metabolic** | **Ischemic stroke** | **Published GWAS** | **Weighted median** | 0.63 | 0.42 | 0.94 | 0.024 |  |  |  |  |  |
| **Cardiovascular and metabolic** | **Ischemic stroke** | **Published GWAS** | **MR-Egger** | 0.52 | 0.18 | 1.52 | 0.241 | 0.00 | 0.573 | 51.06 | 0.030 | 0.82 |
| **Cardiovascular and metabolic** | **Ischemic stroke** | **Published GWAS** | **Penalised weighted median** | 0.63 | 0.42 | 0.94 | 0.024 |  |  |  |  |  |
| **Cardiovascular and metabolic** | **Large artery stroke** | **Published GWAS** | **Inverse-variance weighted** | 0.57 | 0.25 | 1.27 | 0.175 |  |  | 48.70 | 0.062 |  |
| **Cardiovascular and metabolic** | **Large artery stroke** | **Published GWAS** | **Weighted median** | 0.74 | 0.26 | 2.10 | 0.570 |  |  |  |  |  |
| **Cardiovascular and metabolic** | **Large artery stroke** | **Published GWAS** | **MR-Egger** | 0.99 | 0.07 | 13.28 | 0.995 | -0.01 | 0.660 | 48.42 | 0.052 | 0.83 |
| **Cardiovascular and metabolic** | **Large artery stroke** | **Published GWAS** | **Penalised weighted median** | 0.78 | 0.29 | 2.10 | 0.622 |  |  |  |  |  |
| **Cardiovascular and metabolic** | **Peripheral artery disease** | **FinnGen** | **Inverse-variance weighted** | 0.20 | 0.11 | 0.38 | 2.E-05 |  |  | 48.01 | 0.070 |  |
| **Cardiovascular and metabolic** | **Peripheral artery disease** | **FinnGen** | **Weighted median** | 0.20 | 0.09 | 0.44 | 7.E-05 |  |  |  |  |  |
| **Cardiovascular and metabolic** | **Peripheral artery disease** | **FinnGen** | **MR-Egger** | 0.54 | 0.07 | 3.83 | 0.538 | -0.01 | 0.310 | 46.56 | 0.074 | 0.83 |
| **Cardiovascular and metabolic** | **Peripheral artery disease** | **FinnGen** | **Penalised weighted median** | 0.25 | 0.11 | 0.60 | 0.002 |  |  |  |  |  |
| **Cardiovascular and metabolic** | **Pulmonary embolism** | **FinnGen** | **Inverse-variance weighted** | 2.40 | 0.99 | 5.84 | 0.061 |  |  | 56.33 | 0.013 |  |
| **Cardiovascular and metabolic** | **Pulmonary embolism** | **FinnGen** | **Weighted median** | 1.72 | 0.56 | 5.31 | 0.344 |  |  |  |  |  |
| **Cardiovascular and metabolic** | **Pulmonary embolism** | **FinnGen** | **MR-Egger** | 3.63 | 0.23 | 56.60 | 0.364 | -0.01 | 0.757 | 56.17 | 0.010 | 0.84 |
| **Cardiovascular and metabolic** | **Pulmonary embolism** | **FinnGen** | **Penalised weighted median** | 0.92 | 0.31 | 2.69 | 0.873 |  |  |  |  |  |
| **Cardiovascular and metabolic** | **Small vessel stroke** | **Published GWAS** | **Inverse-variance weighted** | 0.32 | 0.14 | 0.75 | 0.012 |  |  | 61.07 | 0.004 |  |
| **Cardiovascular and metabolic** | **Small vessel stroke** | **Published GWAS** | **Weighted median** | 0.18 | 0.07 | 0.47 | 4.E-04 |  |  |  |  |  |
| **Cardiovascular and metabolic** | **Small vessel stroke** | **Published GWAS** | **MR-Egger** | 0.03 | 0.00 | 0.38 | 0.011 | 0.04 | 0.060 | 54.96 | 0.013 | 0.83 |
| **Cardiovascular and metabolic** | **Small vessel stroke** | **Published GWAS** | **Penalised weighted median** | 0.21 | 0.07 | 0.57 | 0.003 |  |  |  |  |  |
| **Cardiovascular and metabolic** | **Stroke** | **FinnGen** | **Inverse-variance weighted** | 0.56 | 0.37 | 0.85 | 0.010 |  |  | 43.96 | 0.142 |  |
| **Cardiovascular and metabolic** | **Stroke** | **FinnGen** | **Weighted median** | 0.62 | 0.36 | 1.06 | 0.081 |  |  |  |  |  |
| **Cardiovascular and metabolic** | **Stroke** | **FinnGen** | **MR-Egger** | 1.36 | 0.39 | 4.74 | 0.636 | -0.01 | 0.150 | 41.33 | 0.181 | 0.84 |
| **Cardiovascular and metabolic** | **Stroke** | **FinnGen** | **Penalised weighted median** | 0.63 | 0.36 | 1.09 | 0.098 |  |  |  |  |  |
| **Cardiovascular and metabolic** | **Stroke** | **Published GWAS** | **Inverse-variance weighted** | 0.70 | 0.53 | 0.93 | 0.019 |  |  | 43.07 | 0.164 |  |
| **Cardiovascular and metabolic** | **Stroke** | **Published GWAS** | **Weighted median** | 0.67 | 0.47 | 0.95 | 0.026 |  |  |  |  |  |
| **Cardiovascular and metabolic** | **Stroke** | **Published GWAS** | **MR-Egger** | 0.46 | 0.19 | 1.12 | 0.097 | 0.01 | 0.331 | 41.87 | 0.166 | 0.82 |
| **Cardiovascular and metabolic** | **Stroke** | **Published GWAS** | **Penalised weighted median** | 0.66 | 0.46 | 0.95 | 0.024 |  |  |  |  |  |
| **Cardiovascular and metabolic** | **Stroke (excl. subarachnoid hemorrhage)** | **FinnGen** | **Inverse-variance weighted** | 0.70 | 0.43 | 1.15 | 0.168 |  |  | 47.53 | 0.077 |  |
| **Cardiovascular and metabolic** | **Stroke (excl. subarachnoid hemorrhage)** | **FinnGen** | **Weighted median** | 0.77 | 0.43 | 1.40 | 0.399 |  |  |  |  |  |
| **Cardiovascular and metabolic** | **Stroke (excl. subarachnoid hemorrhage)** | **FinnGen** | **MR-Egger** | 1.24 | 0.27 | 5.58 | 0.784 | -0.01 | 0.442 | 46.70 | 0.072 | 0.84 |
| **Cardiovascular and metabolic** | **Stroke (excl. subarachnoid hemorrhage)** | **FinnGen** | **Penalised weighted median** | 0.79 | 0.41 | 1.55 | 0.499 |  |  |  |  |  |
| **Cardiovascular and metabolic** | **Venous thromboembolism** | **FinnGen** | **Inverse-variance weighted** | 2.86 | 1.70 | 4.82 | 3.E-04 |  |  | 42.39 | 0.182 |  |
| **Cardiovascular and metabolic** | **Venous thromboembolism** | **FinnGen** | **Weighted median** | 4.17 | 2.04 | 8.53 | 9.E-05 |  |  |  |  |  |
| **Cardiovascular and metabolic** | **Venous thromboembolism** | **FinnGen** | **MR-Egger** | 2.62 | 0.52 | 13.16 | 0.250 | 0.00 | 0.910 | 42.38 | 0.153 | 0.84 |
| **Cardiovascular and metabolic** | **Venous thromboembolism** | **FinnGen** | **Penalised weighted median** | 2.28 | 1.09 | 4.79 | 0.029 |  |  |  |  |  |
| **Cardiovascular and metabolic** | **Venous thromboembolism** | **Published GWAS** | **Inverse-variance weighted** | 2.32 | 1.54 | 3.50 | 3.E-04 |  |  | 34.75 | 0.480 |  |
| **Cardiovascular and metabolic** | **Venous thromboembolism** | **Published GWAS** | **Weighted median** | 1.82 | 0.97 | 3.38 | 0.060 |  |  |  |  |  |
| **Cardiovascular and metabolic** | **Venous thromboembolism** | **Published GWAS** | **MR-Egger** | 2.30 | 0.60 | 8.87 | 0.234 | 0.00 | 0.989 | 34.75 | 0.432 | 0.94 |
| **Cardiovascular and metabolic** | **Venous thromboembolism** | **Published GWAS** | **Penalised weighted median** | 1.75 | 0.90 | 3.40 | 0.097 |  |  |  |  |  |
| **Cardiovascular and metabolic** | **Polycystic ovary syndrome** | **FinnGen** | **Inverse-variance weighted** | 0.35 | 0.06 | 2.02 | 0.249 |  |  | 24.09 | 0.918 |  |
| **Cardiovascular and metabolic** | **Polycystic ovary syndrome** | **FinnGen** | **Weighted median** | 0.37 | 0.03 | 4.34 | 0.428 |  |  |  |  |  |
| **Cardiovascular and metabolic** | **Polycystic ovary syndrome** | **FinnGen** | **MR-Egger** | 0.65 | 0.00 | 134.33 | 0.876 | -0.01 | 0.778 | 24.03 | 0.898 | 0.84 |
| **Cardiovascular and metabolic** | **Polycystic ovary syndrome** | **FinnGen** | **Penalised weighted median** | 0.83 | 0.08 | 8.87 | 0.878 |  |  |  |  |  |
| **Cardiovascular and metabolic** | **Polycystic ovary syndrome** | **Published GWAS** | **Inverse-variance weighted** | 0.58 | 0.21 | 1.59 | 0.296 |  |  | 48.08 | 0.069 |  |
| **Cardiovascular and metabolic** | **Polycystic ovary syndrome** | **Published GWAS** | **Weighted median** | 0.72 | 0.20 | 2.58 | 0.610 |  |  |  |  |  |
| **Cardiovascular and metabolic** | **Polycystic ovary syndrome** | **Published GWAS** | **MR-Egger** | 2.57 | 0.10 | 68.82 | 0.578 | -0.02 | 0.357 | 46.88 | 0.070 | 0.85 |
| **Cardiovascular and metabolic** | **Polycystic ovary syndrome** | **Published GWAS** | **Penalised weighted median** | 0.72 | 0.19 | 2.68 | 0.623 |  |  |  |  |  |
| **Cardiovascular and metabolic** | **Type 2 diabetes** | **FinnGen** | **Inverse-variance weighted** | 0.12 | 0.07 | 0.20 | 1.E-08 |  |  | 119.67 | 3.E-11 |  |
| **Cardiovascular and metabolic** | **Type 2 diabetes** | **FinnGen** | **Weighted median** | 0.15 | 0.08 | 0.27 | 5.E-10 |  |  |  |  |  |
| **Cardiovascular and metabolic** | **Type 2 diabetes** | **FinnGen** | **MR-Egger** | 0.14 | 0.02 | 0.82 | 0.037 | 0.00 | 0.812 | 119.47 | 2.E-11 | 0.84 |
| **Cardiovascular and metabolic** | **Type 2 diabetes** | **FinnGen** | **Penalised weighted median** | 0.33 | 0.17 | 0.64 | 9.E-04 |  |  |  |  |  |
| **Cardiovascular and metabolic** | **Type 2 diabetes** | **Published GWAS** | **Inverse-variance weighted** | 0.11 | 0.07 | 0.18 | 4.E-11 |  |  | 150.24 | 3.E-16 |  |
| **Cardiovascular and metabolic** | **Type 2 diabetes** | **Published GWAS** | **Weighted median** | 0.12 | 0.08 | 0.18 | 7.E-23 |  |  |  |  |  |
| **Cardiovascular and metabolic** | **Type 2 diabetes** | **Published GWAS** | **MR-Egger** | 0.11 | 0.03 | 0.49 | 0.007 | 0.00 | 0.982 | 150.23 | 1.E-16 | 0.81 |
| **Cardiovascular and metabolic** | **Type 2 diabetes** | **Published GWAS** | **Penalised weighted median** | 0.36 | 0.23 | 0.57 | 2.E-05 |  |  |  |  |  |
| **Cardiovascular and metabolic** | **Chronic kidney disease** | **FinnGen** | **Inverse-variance weighted** | 0.47 | 0.23 | 0.96 | 0.046 |  |  | 29.66 | 0.723 |  |
| **Cardiovascular and metabolic** | **Chronic kidney disease** | **FinnGen** | **Weighted median** | 0.45 | 0.16 | 1.29 | 0.137 |  |  |  |  |  |
| **Cardiovascular and metabolic** | **Chronic kidney disease** | **FinnGen** | **MR-Egger** | 0.36 | 0.04 | 3.23 | 0.365 | 0.00 | 0.787 | 29.60 | 0.683 | 0.84 |
| **Cardiovascular and metabolic** | **Chronic kidney disease** | **FinnGen** | **Penalised weighted median** | 0.45 | 0.15 | 1.32 | 0.148 |  |  |  |  |  |
| **Cardiovascular and metabolic** | **Chronic kidney disease** | **Published GWAS** | **Inverse-variance weighted** | 0.67 | 0.49 | 0.92 | 0.016 |  |  | 54.55 | 0.019 |  |
| **Cardiovascular and metabolic** | **Chronic kidney disease** | **Published GWAS** | **Weighted median** | 0.71 | 0.48 | 1.05 | 0.085 |  |  |  |  |  |
| **Cardiovascular and metabolic** | **Chronic kidney disease** | **Published GWAS** | **MR-Egger** | 0.60 | 0.22 | 1.62 | 0.317 | 0.00 | 0.808 | 54.46 | 0.014 | 0.84 |
| **Cardiovascular and metabolic** | **Chronic kidney disease** | **Published GWAS** | **Penalised weighted median** | 0.89 | 0.57 | 1.38 | 0.593 |  |  |  |  |  |
| **Musculoskeletal** | **Gout** | **FinnGen** | **Inverse-variance weighted** | 0.43 | 0.16 | 1.13 | 0.096 |  |  | 55.10 | 0.017 |  |
| **Musculoskeletal** | **Gout** | **FinnGen** | **Weighted median** | 0.32 | 0.10 | 1.04 | 0.058 |  |  |  |  |  |
| **Musculoskeletal** | **Gout** | **FinnGen** | **MR-Egger** | 0.23 | 0.01 | 4.53 | 0.340 | 0.01 | 0.663 | 54.79 | 0.013 | 0.84 |
| **Musculoskeletal** | **Gout** | **FinnGen** | **Penalised weighted median** | 0.37 | 0.11 | 1.24 | 0.109 |  |  |  |  |  |
| **Musculoskeletal** | **Gout** | **Published GWAS** | **Inverse-variance weighted** | 0.44 | 0.28 | 0.71 | 0.002 |  |  | 61.77 | 0.003 |  |
| **Musculoskeletal** | **Gout** | **Published GWAS** | **Weighted median** | 0.53 | 0.31 | 0.89 | 0.017 |  |  |  |  |  |
| **Musculoskeletal** | **Gout** | **Published GWAS** | **MR-Egger** | 0.40 | 0.09 | 1.85 | 0.250 | 0.00 | 0.889 | 61.74 | 0.003 | 0.92 |
| **Musculoskeletal** | **Gout** | **Published GWAS** | **Penalised weighted median** | 0.55 | 0.31 | 0.97 | 0.039 |  |  |  |  |  |
| **Musculoskeletal** | **Osteoarthritis** | **FinnGen** | **Inverse-variance weighted** | 1.46 | 1.03 | 2.07 | 0.039 |  |  | 53.59 | 0.023 |  |
| **Musculoskeletal** | **Osteoarthritis** | **FinnGen** | **Weighted median** | 1.47 | 0.95 | 2.28 | 0.086 |  |  |  |  |  |
| **Musculoskeletal** | **Osteoarthritis** | **FinnGen** | **MR-Egger** | 1.51 | 0.52 | 4.44 | 0.456 | 0.00 | 0.947 | 53.58 | 0.018 | 0.83 |
| **Musculoskeletal** | **Osteoarthritis** | **FinnGen** | **Penalised weighted median** | 1.47 | 0.89 | 2.43 | 0.131 |  |  |  |  |  |
| **Musculoskeletal** | **Osteoarthritis** | **Published GWAS** | **Inverse-variance weighted** | 1.44 | 1.14 | 1.82 | 0.004 |  |  | 89.86 | 1.E-06 |  |
| **Musculoskeletal** | **Osteoarthritis** | **Published GWAS** | **Weighted median** | 1.30 | 1.03 | 1.65 | 0.026 |  |  |  |  |  |
| **Musculoskeletal** | **Osteoarthritis** | **Published GWAS** | **MR-Egger** | 0.91 | 0.44 | 1.87 | 0.797 | 0.01 | 0.195 | 85.48 | 3.E-06 | 0.81 |
| **Musculoskeletal** | **Osteoarthritis** | **Published GWAS** | **Penalised weighted median** | 1.02 | 0.79 | 1.30 | 0.899 |  |  |  |  |  |
| **Musculoskeletal** | **Osteoarthritis (hip and/or knee)** | **Published GWAS** | **Inverse-variance weighted** | 1.78 | 1.31 | 2.40 | 7.E-04 |  |  | 78.44 | 4.E-05 |  |
| **Musculoskeletal** | **Osteoarthritis (hip and/or knee)** | **Published GWAS** | **Weighted median** | 1.59 | 1.14 | 2.22 | 0.006 |  |  |  |  |  |
| **Musculoskeletal** | **Osteoarthritis (hip and/or knee)** | **Published GWAS** | **MR-Egger** | 1.12 | 0.43 | 2.89 | 0.819 | 0.01 | 0.319 | 76.15 | 5.E-05 | 0.81 |
| **Musculoskeletal** | **Osteoarthritis (hip and/or knee)** | **Published GWAS** | **Penalised weighted median** | 1.08 | 0.76 | 1.53 | 0.653 |  |  |  |  |  |
| **Musculoskeletal** | **Osteoarthritis (hip)** | **FinnGen** | **Inverse-variance weighted** | 1.06 | 0.59 | 1.90 | 0.846 |  |  | 56.24 | 0.013 |  |
| **Musculoskeletal** | **Osteoarthritis (hip)** | **FinnGen** | **Weighted median** | 1.06 | 0.51 | 2.20 | 0.870 |  |  |  |  |  |
| **Musculoskeletal** | **Osteoarthritis (hip)** | **FinnGen** | **MR-Egger** | 1.37 | 0.22 | 8.35 | 0.738 | 0.00 | 0.774 | 56.10 | 0.010 | 0.84 |
| **Musculoskeletal** | **Osteoarthritis (hip)** | **FinnGen** | **Penalised weighted median** | 0.60 | 0.27 | 1.32 | 0.205 |  |  |  |  |  |
| **Musculoskeletal** | **Osteoarthritis (knee)** | **FinnGen** | **Inverse-variance weighted** | 2.06 | 1.43 | 2.96 | 5.E-04 |  |  | 42.40 | 0.182 |  |
| **Musculoskeletal** | **Osteoarthritis (knee)** | **FinnGen** | **Weighted median** | 2.06 | 1.25 | 3.38 | 0.004 |  |  |  |  |  |
| **Musculoskeletal** | **Osteoarthritis (knee)** | **FinnGen** | **MR-Egger** | 2.69 | 0.87 | 8.32 | 0.096 | 0.00 | 0.627 | 42.11 | 0.160 | 0.84 |
| **Musculoskeletal** | **Osteoarthritis (knee)** | **FinnGen** | **Penalised weighted median** | 1.91 | 1.15 | 3.20 | 0.013 |  |  |  |  |  |
| **Musculoskeletal** | **Osteoporosis** | **FinnGen** | **Inverse-variance weighted** | 1.71 | 0.75 | 3.87 | 0.209 |  |  | 38.91 | 0.298 |  |
| **Musculoskeletal** | **Osteoporosis** | **FinnGen** | **Weighted median** | 1.03 | 0.36 | 2.99 | 0.955 |  |  |  |  |  |
| **Musculoskeletal** | **Osteoporosis** | **FinnGen** | **MR-Egger** | 1.27 | 0.10 | 15.99 | 0.856 | 0.00 | 0.809 | 38.84 | 0.261 | 0.83 |
| **Musculoskeletal** | **Osteoporosis** | **FinnGen** | **Penalised weighted median** | 0.99 | 0.32 | 3.02 | 0.979 |  |  |  |  |  |
| **Musculoskeletal** | **Osteoporosis** | **Published GWAS** | **Inverse-variance weighted** | 1.03 | 0.84 | 1.27 | 0.764 |  |  | 585.90 | 9.E-102 |  |
| **Musculoskeletal** | **Osteoporosis** | **Published GWAS** | **Weighted median** | 1.13 | 1.02 | 1.25 | 0.015 |  |  |  |  |  |
| **Musculoskeletal** | **Osteoporosis** | **Published GWAS** | **MR-Egger** | 0.64 | 0.34 | 1.20 | 0.175 | 0.01 | 0.127 | 545.33 | 4.E-94 | 0.81 |
| **Musculoskeletal** | **Osteoporosis** | **Published GWAS** | **Penalised weighted median** | 1.17 | 1.00 | 1.37 | 0.049 |  |  |  |  |  |
| **Musculoskeletal** | **Rheumatoid arthritis** | **FinnGen** | **Inverse-variance weighted** | 1.85 | 0.97 | 3.53 | 0.069 |  |  | 42.92 | 0.168 |  |
| **Musculoskeletal** | **Rheumatoid arthritis** | **FinnGen** | **Weighted median** | 2.31 | 0.97 | 5.50 | 0.059 |  |  |  |  |  |
| **Musculoskeletal** | **Rheumatoid arthritis** | **FinnGen** | **MR-Egger** | 4.24 | 0.59 | 30.42 | 0.160 | -0.01 | 0.389 | 41.98 | 0.163 | 0.84 |
| **Musculoskeletal** | **Rheumatoid arthritis** | **FinnGen** | **Penalised weighted median** | 2.20 | 0.89 | 5.44 | 0.090 |  |  |  |  |  |
| **Musculoskeletal** | **Rheumatoid arthritis** | **Published GWAS** | **Inverse-variance weighted** | 2.06 | 1.08 | 3.92 | 0.034 |  |  | 52.50 | 0.029 |  |
| **Musculoskeletal** | **Rheumatoid arthritis** | **Published GWAS** | **Weighted median** | 1.63 | 0.72 | 3.69 | 0.238 |  |  |  |  |  |
| **Musculoskeletal** | **Rheumatoid arthritis** | **Published GWAS** | **MR-Egger** | 0.18 | 0.03 | 1.18 | 0.083 | 0.04 | 0.011 | 43.33 | 0.131 | 0.93 |
| **Musculoskeletal** | **Rheumatoid arthritis** | **Published GWAS** | **Penalised weighted median** | 1.58 | 0.71 | 3.52 | 0.258 |  |  |  |  |  |
| **Gastrointestinal** | **Gallstones** | **FinnGen** | **Inverse-variance weighted** | 1.37 | 0.86 | 2.19 | 0.200 |  |  | 63.67 | 0.002 |  |
| **Gastrointestinal** | **Gallstones** | **FinnGen** | **Weighted median** | 1.19 | 0.71 | 2.02 | 0.507 |  |  |  |  |  |
| **Gastrointestinal** | **Gallstones** | **FinnGen** | **MR-Egger** | 1.72 | 0.40 | 7.38 | 0.467 | 0.00 | 0.743 | 63.47 | 0.002 | 0.84 |
| **Gastrointestinal** | **Gallstones** | **FinnGen** | **Penalised weighted median** | 0.93 | 0.54 | 1.58 | 0.777 |  |  |  |  |  |
| **Gastrointestinal** | **Gastro-oesophageal reflux disease** | **Published GWAS** | **Inverse-variance weighted** | 1.19 | 0.95 | 1.47 | 0.133 |  |  | 68.21 | 7.E-04 |  |
| **Gastrointestinal** | **Gastro-oesophageal reflux disease** | **Published GWAS** | **Weighted median** | 1.28 | 0.99 | 1.65 | 0.064 |  |  |  |  |  |
| **Gastrointestinal** | **Gastro-oesophageal reflux disease** | **Published GWAS** | **MR-Egger** | 1.29 | 0.65 | 2.57 | 0.470 | 0.00 | 0.797 | 68.07 | 5.E-04 | 0.81 |
| **Gastrointestinal** | **Gastro-oesophageal reflux disease** | **Published GWAS** | **Penalised weighted median** | 1.29 | 0.97 | 1.73 | 0.081 |  |  |  |  |  |
| **Nervous** | **Alzheimer's disease** | **FinnGen** | **Inverse-variance weighted** | 0.77 | 0.36 | 1.61 | 0.484 |  |  | 34.27 | 0.503 |  |
| **Nervous** | **Alzheimer's disease** | **FinnGen** | **Weighted median** | 0.93 | 0.31 | 2.84 | 0.905 |  |  |  |  |  |
| **Nervous** | **Alzheimer's disease** | **FinnGen** | **MR-Egger** | 0.54 | 0.06 | 5.21 | 0.596 | 0.01 | 0.749 | 34.17 | 0.460 | 0.83 |
| **Nervous** | **Alzheimer's disease** | **FinnGen** | **Penalised weighted median** | 1.29 | 0.43 | 3.84 | 0.650 |  |  |  |  |  |
| **Nervous** | **Alzheimer's disease** | **Published GWAS (1)** | **Inverse-variance weighted** | 1.00 | 0.93 | 1.08 | 0.997 |  |  | 55.84 | 0.014 |  |
| **Nervous** | **Alzheimer's disease** | **Published GWAS (1)** | **Weighted median** | 0.99 | 0.90 | 1.09 | 0.813 |  |  |  |  |  |
| **Nervous** | **Alzheimer's disease** | **Published GWAS (1)** | **MR-Egger** | 0.91 | 0.71 | 1.16 | 0.453 | 0.00 | 0.431 | 54.82 | 0.013 | 0.97 |
| **Nervous** | **Alzheimer's disease** | **Published GWAS (1)** | **Penalised weighted median** | 0.95 | 0.83 | 1.10 | 0.510 |  |  |  |  |  |
| **Nervous** | **Alzheimer's disease** | **Published GWAS (2)** | **Inverse-variance weighted** | 0.94 | 0.63 | 1.39 | 0.751 |  |  | 30.36 | 0.692 |  |
| **Nervous** | **Alzheimer's disease** | **Published GWAS (2)** | **Weighted median** | 0.76 | 0.44 | 1.33 | 0.339 |  |  |  |  |  |
| **Nervous** | **Alzheimer's disease** | **Published GWAS (2)** | **MR-Egger** | 0.59 | 0.16 | 2.11 | 0.420 | 0.01 | 0.426 | 29.79 | 0.674 | 0.86 |
| **Nervous** | **Alzheimer's disease** | **Published GWAS (2)** | **Penalised weighted median** | 0.71 | 0.40 | 1.28 | 0.254 |  |  |  |  |  |
| **Nervous** | **Depression** | **FinnGen** | **Inverse-variance weighted** | 1.21 | 0.87 | 1.66 | 0.260 |  |  | 34.86 | 0.475 |  |
| **Nervous** | **Depression** | **FinnGen** | **Weighted median** | 1.44 | 0.91 | 2.27 | 0.116 |  |  |  |  |  |
| **Nervous** | **Depression** | **FinnGen** | **MR-Egger** | 1.54 | 0.57 | 4.14 | 0.400 | 0.00 | 0.614 | 34.60 | 0.439 | 0.84 |
| **Nervous** | **Depression** | **FinnGen** | **Penalised weighted median** | 1.45 | 0.87 | 2.40 | 0.155 |  |  |  |  |  |
| **Nervous** | **Depression** | **Published GWAS** | **Inverse-variance weighted** | 1.20 | 0.92 | 1.56 | 0.180 |  |  | 40.76 | 0.232 |  |
| **Nervous** | **Depression** | **Published GWAS** | **Weighted median** | 1.41 | 0.99 | 2.01 | 0.054 |  |  |  |  |  |
| **Nervous** | **Depression** | **Published GWAS** | **MR-Egger** | 0.77 | 0.33 | 1.79 | 0.552 | 0.01 | 0.286 | 39.40 | 0.241 | 0.82 |
| **Nervous** | **Depression** | **Published GWAS** | **Penalised weighted median** | 1.47 | 0.99 | 2.18 | 0.055 |  |  |  |  |  |
| **Nervous** | **Multiple sclerosis** | **FinnGen** | **Inverse-variance weighted** | 1.53 | 0.38 | 6.20 | 0.559 |  |  | 39.44 | 0.278 |  |
| **Nervous** | **Multiple sclerosis** | **FinnGen** | **Weighted median** | 0.82 | 0.12 | 5.68 | 0.837 |  |  |  |  |  |
| **Nervous** | **Multiple sclerosis** | **FinnGen** | **MR-Egger** | 5.99 | 0.08 | 450.09 | 0.422 | -0.02 | 0.516 | 38.94 | 0.257 | 0.84 |
| **Nervous** | **Multiple sclerosis** | **FinnGen** | **Penalised weighted median** | 0.82 | 0.12 | 5.71 | 0.838 |  |  |  |  |  |
| **Nervous** | **Parkinson's disease** | **FinnGen** | **Inverse-variance weighted** | 1.01 | 0.38 | 2.69 | 0.984 |  |  | 33.75 | 0.528 |  |
| **Nervous** | **Parkinson's disease** | **FinnGen** | **Weighted median** | 0.83 | 0.19 | 3.68 | 0.809 |  |  |  |  |  |
| **Nervous** | **Parkinson's disease** | **FinnGen** | **MR-Egger** | 1.18 | 0.06 | 23.68 | 0.913 | 0.00 | 0.913 | 33.74 | 0.480 | 0.83 |
| **Nervous** | **Parkinson's disease** | **FinnGen** | **Penalised weighted median** | 1.14 | 0.27 | 4.83 | 0.858 |  |  |  |  |  |
| **Nervous** | **Parkinson's disease** | **Published GWAS** | **Inverse-variance weighted** | 1.45 | 0.89 | 2.34 | 0.143 |  |  | 33.71 | 0.530 |  |
| **Nervous** | **Parkinson's disease** | **Published GWAS** | **Weighted median** | 0.94 | 0.47 | 1.88 | 0.856 |  |  |  |  |  |
| **Nervous** | **Parkinson's disease** | **Published GWAS** | **MR-Egger** | 1.12 | 0.24 | 5.15 | 0.883 | 0.00 | 0.732 | 33.59 | 0.487 | 0.89 |
| **Nervous** | **Parkinson's disease** | **Published GWAS** | **Penalised weighted median** | 0.90 | 0.45 | 1.81 | 0.765 |  |  |  |  |  |
| **Integumentary** | **Psoriasis** | **FinnGen** | **Inverse-variance weighted** | 0.92 | 0.39 | 2.16 | 0.854 |  |  | 56.97 | 0.011 |  |
| **Integumentary** | **Psoriasis** | **FinnGen** | **Weighted median** | 0.77 | 0.27 | 2.24 | 0.634 |  |  |  |  |  |
| **Integumentary** | **Psoriasis** | **FinnGen** | **MR-Egger** | 0.10 | 0.01 | 1.17 | 0.075 | 0.03 | 0.069 | 51.61 | 0.027 | 0.84 |
| **Integumentary** | **Psoriasis** | **FinnGen** | **Penalised weighted median** | 0.77 | 0.28 | 2.14 | 0.611 |  |  |  |  |  |
| **Integumentary** | **Psoriasis** | **Published GWAS** | **Inverse-variance weighted** | 1.43 | 0.71 | 2.90 | 0.323 |  |  | 63.63 | 0.002 |  |
| **Integumentary** | **Psoriasis** | **Published GWAS** | **Weighted median** | 1.06 | 0.49 | 2.29 | 0.887 |  |  |  |  |  |
| **Integumentary** | **Psoriasis** | **Published GWAS** | **MR-Egger** | 0.81 | 0.08 | 7.94 | 0.857 | 0.01 | 0.608 | 63.13 | 0.002 | 0.92 |
| **Integumentary** | **Psoriasis** | **Published GWAS** | **Penalised weighted median** | 0.84 | 0.37 | 1.90 | 0.680 |  |  |  |  |  |
| **Respiratory** | **Adult-onset asthma** | **Published GWAS** | **Inverse-variance weighted** | 1.07 | 0.75 | 1.51 | 0.712 |  |  | 88.24 | 2.E-06 |  |
| **Respiratory** | **Adult-onset asthma** | **Published GWAS** | **Weighted median** | 1.14 | 0.78 | 1.67 | 0.495 |  |  |  |  |  |
| **Respiratory** | **Adult-onset asthma** | **Published GWAS** | **MR-Egger** | 1.63 | 0.55 | 4.86 | 0.388 | -0.01 | 0.430 | 86.62 | 2.E-06 | 0.81 |
| **Respiratory** | **Adult-onset asthma** | **Published GWAS** | **Penalised weighted median** | 0.98 | 0.66 | 1.44 | 0.909 |  |  |  |  |  |
| **Respiratory** | **Asthma** | **FinnGen** | **Inverse-variance weighted** | 1.26 | 0.84 | 1.89 | 0.279 |  |  | 51.50 | 0.036 |  |
| **Respiratory** | **Asthma** | **FinnGen** | **Weighted median** | 1.13 | 0.68 | 1.89 | 0.633 |  |  |  |  |  |
| **Respiratory** | **Asthma** | **FinnGen** | **MR-Egger** | 0.84 | 0.24 | 2.93 | 0.782 | 0.01 | 0.505 | 50.82 | 0.032 | 0.84 |
| **Respiratory** | **Asthma** | **FinnGen** | **Penalised weighted median** | 1.11 | 0.66 | 1.88 | 0.696 |  |  |  |  |  |
| **Respiratory** | **Child-onset asthma** | **Published GWAS** | **Inverse-variance weighted** | 0.66 | 0.40 | 1.07 | 0.099 |  |  | 89.42 | 1.E-06 |  |
| **Respiratory** | **Child-onset asthma** | **Published GWAS** | **Weighted median** | 0.63 | 0.39 | 1.03 | 0.066 |  |  |  |  |  |
| **Respiratory** | **Child-onset asthma** | **Published GWAS** | **MR-Egger** | 1.09 | 0.23 | 5.04 | 0.915 | -0.01 | 0.502 | 88.22 | 1.E-06 | 0.81 |
| **Respiratory** | **Child-onset asthma** | **Published GWAS** | **Penalised weighted median** | 0.77 | 0.46 | 1.29 | 0.319 |  |  |  |  |  |
| **Cancer** | **Barrett's oesophagus** | **FinnGen** | **Inverse-variance weighted** | 3.16 | 0.39 | 25.47 | 0.288 |  |  | 36.10 | 0.417 |  |
| **Cancer** | **Barrett's oesophagus** | **FinnGen** | **Weighted median** | 2.66 | 0.14 | 51.84 | 0.518 |  |  |  |  |  |
| **Cancer** | **Barrett's oesophagus** | **FinnGen** | **MR-Egger** | 262.73 | 0.49 | 140007.26 | 0.091 | -0.07 | 0.153 | 33.97 | 0.469 | 0.84 |
| **Cancer** | **Barrett's oesophagus** | **FinnGen** | **Penalised weighted median** | 2.12 | 0.12 | 38.74 | 0.613 |  |  |  |  |  |
| **Cancer** | **Breast cancer** | **FinnGen** | **Inverse-variance weighted** | 1.56 | 0.69 | 3.54 | 0.291 |  |  | 85.68 | 4.E-06 |  |
| **Cancer** | **Breast cancer** | **FinnGen** | **Weighted median** | 1.53 | 0.71 | 3.27 | 0.277 |  |  |  |  |  |
| **Cancer** | **Breast cancer** | **FinnGen** | **MR-Egger** | 0.49 | 0.04 | 5.90 | 0.576 | 0.02 | 0.339 | 83.38 | 5.E-06 | 0.84 |
| **Cancer** | **Breast cancer** | **FinnGen** | **Penalised weighted median** | 1.36 | 0.62 | 2.99 | 0.445 |  |  |  |  |  |
| **Cancer** | **Breast cancer** | **Published GWAS** | **Inverse-variance weighted** | 0.88 | 0.45 | 1.73 | 0.719 |  |  | 72.31 | 2.E-04 |  |
| **Cancer** | **Breast cancer** | **Published GWAS** | **Weighted median** | 0.47 | 0.22 | 1.00 | 0.050 |  |  |  |  |  |
| **Cancer** | **Breast cancer** | **Published GWAS** | **MR-Egger** | 0.30 | 0.04 | 2.51 | 0.274 | 0.02 | 0.301 | 70.04 | 3.E-04 | 0.86 |
| **Cancer** | **Breast cancer** | **Published GWAS** | **Penalised weighted median** | 0.48 | 0.23 | 0.98 | 0.044 |  |  |  |  |  |
| **Cancer** | **Cancer myeloma** | **FinnGen** | **Inverse-variance weighted** | 1.96 | 0.25 | 15.59 | 0.529 |  |  | 45.67 | 0.107 |  |
| **Cancer** | **Cancer myeloma** | **FinnGen** | **Weighted median** | 2.30 | 0.14 | 38.75 | 0.564 |  |  |  |  |  |
| **Cancer** | **Cancer myeloma** | **FinnGen** | **MR-Egger** | 2.07 | 0.00 | 1276.90 | 0.825 | 0.00 | 0.986 | 45.67 | 0.087 | 0.84 |
| **Cancer** | **Cancer myeloma** | **FinnGen** | **Penalised weighted median** | 2.97 | 0.20 | 44.00 | 0.429 |  |  |  |  |  |
| **Cancer** | **Colon cancer** | **Published GWAS** | **Inverse-variance weighted** | 0.79 | 0.57 | 1.11 | 0.187 |  |  | 41.56 | 0.206 |  |
| **Cancer** | **Colon cancer** | **Published GWAS** | **Weighted median** | 0.91 | 0.58 | 1.42 | 0.673 |  |  |  |  |  |
| **Cancer** | **Colon cancer** | **Published GWAS** | **MR-Egger** | 0.94 | 0.32 | 2.78 | 0.911 | 0.00 | 0.751 | 41.44 | 0.178 | 0.82 |
| **Cancer** | **Colon cancer** | **Published GWAS** | **Penalised weighted median** | 0.84 | 0.52 | 1.37 | 0.496 |  |  |  |  |  |
| **Cancer** | **Colorectal cancer** | **FinnGen** | **Inverse-variance weighted** | 0.56 | 0.25 | 1.25 | 0.167 |  |  | 37.76 | 0.344 |  |
| **Cancer** | **Colorectal cancer** | **FinnGen** | **Weighted median** | 0.43 | 0.14 | 1.27 | 0.126 |  |  |  |  |  |
| **Cancer** | **Colorectal cancer** | **FinnGen** | **MR-Egger** | 0.33 | 0.03 | 3.94 | 0.388 | 0.01 | 0.662 | 37.55 | 0.310 | 0.84 |
| **Cancer** | **Colorectal cancer** | **FinnGen** | **Penalised weighted median** | 0.43 | 0.14 | 1.29 | 0.131 |  |  |  |  |  |
| **Cancer** | **Colorectal cancer** | **Published GWAS (1)** | **Inverse-variance weighted** | 0.57 | 0.39 | 0.83 | 0.005 |  |  | 56.34 | 0.013 |  |
| **Cancer** | **Colorectal cancer** | **Published GWAS (1)** | **Weighted median** | 0.49 | 0.32 | 0.77 | 0.002 |  |  |  |  |  |
| **Cancer** | **Colorectal cancer** | **Published GWAS (1)** | **MR-Egger** | 0.46 | 0.14 | 1.51 | 0.208 | 0.00 | 0.709 | 56.11 | 0.010 | 0.84 |
| **Cancer** | **Colorectal cancer** | **Published GWAS (1)** | **Penalised weighted median** | 0.50 | 0.32 | 0.78 | 0.002 |  |  |  |  |  |
| **Cancer** | **Colorectal cancer** | **Published GWAS (2)** | **Inverse-variance weighted** | 0.76 | 0.57 | 1.02 | 0.080 |  |  | 46.24 | 0.097 |  |
| **Cancer** | **Colorectal cancer** | **Published GWAS (2)** | **Weighted median** | 0.78 | 0.54 | 1.14 | 0.202 |  |  |  |  |  |
| **Cancer** | **Colorectal cancer** | **Published GWAS (2)** | **MR-Egger** | 0.67 | 0.26 | 1.73 | 0.408 | 0.00 | 0.769 | 46.12 | 0.080 | 0.83 |
| **Cancer** | **Colorectal cancer** | **Published GWAS (2)** | **Penalised weighted median** | 0.79 | 0.54 | 1.15 | 0.216 |  |  |  |  |  |
| **Cancer** | **Distal colon cancer** | **Published GWAS** | **Inverse-variance weighted** | 0.84 | 0.55 | 1.28 | 0.428 |  |  | 39.10 | 0.291 |  |
| **Cancer** | **Distal colon cancer** | **Published GWAS** | **Weighted median** | 0.86 | 0.48 | 1.55 | 0.625 |  |  |  |  |  |
| **Cancer** | **Distal colon cancer** | **Published GWAS** | **MR-Egger** | 0.59 | 0.15 | 2.31 | 0.456 | 0.01 | 0.598 | 38.77 | 0.263 | 0.83 |
| **Cancer** | **Distal colon cancer** | **Published GWAS** | **Penalised weighted median** | 0.86 | 0.48 | 1.54 | 0.616 |  |  |  |  |  |
| **Cancer** | **Endometrial cancer** | **FinnGen** | **Inverse-variance weighted** | 1.06 | 0.27 | 4.11 | 0.931 |  |  | 39.45 | 0.278 |  |
| **Cancer** | **Endometrial cancer** | **FinnGen** | **Weighted median** | 1.10 | 0.17 | 7.01 | 0.919 |  |  |  |  |  |
| **Cancer** | **Endometrial cancer** | **FinnGen** | **MR-Egger** | 6.72 | 0.11 | 420.85 | 0.373 | -0.03 | 0.362 | 38.48 | 0.274 | 0.84 |
| **Cancer** | **Endometrial cancer** | **FinnGen** | **Penalised weighted median** | 2.76 | 0.44 | 17.31 | 0.280 |  |  |  |  |  |
| **Cancer** | **Endometrial cancer** | **Published GWAS** | **Inverse-variance weighted** | 0.91 | 0.49 | 1.67 | 0.755 |  |  | 79.07 | 3.E-05 |  |
| **Cancer** | **Endometrial cancer** | **Published GWAS** | **Weighted median** | 0.90 | 0.45 | 1.79 | 0.767 |  |  |  |  |  |
| **Cancer** | **Endometrial cancer** | **Published GWAS** | **MR-Egger** | 0.34 | 0.05 | 2.30 | 0.274 | 0.01 | 0.293 | 76.50 | 4.E-05 | 0.83 |
| **Cancer** | **Endometrial cancer** | **Published GWAS** | **Penalised weighted median** | 0.91 | 0.46 | 1.82 | 0.801 |  |  |  |  |  |
| **Cancer** | **Lung cancer** | **FinnGen** | **Inverse-variance weighted** | 0.64 | 0.23 | 1.78 | 0.401 |  |  | 27.00 | 0.831 |  |
| **Cancer** | **Lung cancer** | **FinnGen** | **Weighted median** | 0.48 | 0.12 | 2.00 | 0.316 |  |  |  |  |  |
| **Cancer** | **Lung cancer** | **FinnGen** | **MR-Egger** | 0.36 | 0.02 | 8.06 | 0.525 | 0.01 | 0.668 | 26.85 | 0.803 | 0.84 |
| **Cancer** | **Lung cancer** | **FinnGen** | **Penalised weighted median** | 0.46 | 0.10 | 2.17 | 0.329 |  |  |  |  |  |
| **Cancer** | **Meningioma** | **FinnGen** | **Inverse-variance weighted** | 1.06 | 0.31 | 3.61 | 0.928 |  |  | 26.53 | 0.848 |  |
| **Cancer** | **Meningioma** | **FinnGen** | **Weighted median** | 0.67 | 0.11 | 4.05 | 0.661 |  |  |  |  |  |
| **Cancer** | **Meningioma** | **FinnGen** | **MR-Egger** | 6.23 | 0.15 | 263.36 | 0.345 | -0.03 | 0.266 | 25.56 | 0.851 | 0.84 |
| **Cancer** | **Meningioma** | **FinnGen** | **Penalised weighted median** | 0.53 | 0.09 | 3.12 | 0.479 |  |  |  |  |  |
| **Cancer** | **Ovarian cancer** | **FinnGen** | **Inverse-variance weighted** | 0.36 | 0.07 | 1.96 | 0.244 |  |  | 43.75 | 0.147 |  |
| **Cancer** | **Ovarian cancer** | **FinnGen** | **Weighted median** | 0.27 | 0.03 | 2.59 | 0.258 |  |  |  |  |  |
| **Cancer** | **Ovarian cancer** | **FinnGen** | **MR-Egger** | 0.10 | 0.00 | 18.40 | 0.390 | 0.02 | 0.609 | 43.41 | 0.129 | 0.84 |
| **Cancer** | **Ovarian cancer** | **FinnGen** | **Penalised weighted median** | 0.40 | 0.04 | 3.91 | 0.430 |  |  |  |  |  |
| **Cancer** | **Ovarian cancer - clear cell cancer** | **Published GWAS** | **Inverse-variance weighted** | 1.25 | 0.43 | 3.62 | 0.684 |  |  | 34.18 | 0.507 |  |
| **Cancer** | **Ovarian cancer - clear cell cancer** | **Published GWAS** | **Weighted median** | 1.59 | 0.35 | 7.19 | 0.550 |  |  |  |  |  |
| **Cancer** | **Ovarian cancer - clear cell cancer** | **Published GWAS** | **MR-Egger** | 0.59 | 0.02 | 16.94 | 0.757 | 0.01 | 0.644 | 33.97 | 0.469 | 0.83 |
| **Cancer** | **Ovarian cancer - clear cell cancer** | **Published GWAS** | **Penalised weighted median** | 1.30 | 0.29 | 5.78 | 0.730 |  |  |  |  |  |
| **Cancer** | **Ovarian cancer - endometrioid** | **Published GWAS** | **Inverse-variance weighted** | 0.42 | 0.17 | 1.06 | 0.074 |  |  | 50.98 | 0.040 |  |
| **Cancer** | **Ovarian cancer - endometrioid** | **Published GWAS** | **Weighted median** | 0.37 | 0.12 | 1.16 | 0.088 |  |  |  |  |  |
| **Cancer** | **Ovarian cancer - endometrioid** | **Published GWAS** | **MR-Egger** | 0.02 | 0.00 | 0.24 | 0.005 | 0.05 | 0.018 | 43.15 | 0.135 | 0.83 |
| **Cancer** | **Ovarian cancer - endometrioid** | **Published GWAS** | **Penalised weighted median** | 0.38 | 0.11 | 1.29 | 0.120 |  |  |  |  |  |
| **Cancer** | **Ovarian cancer - epithelial** | **Published GWAS** | **Inverse-variance weighted** | 0.35 | 0.17 | 0.74 | 0.010 |  |  | 29.32 | 0.738 |  |
| **Cancer** | **Ovarian cancer - epithelial** | **Published GWAS** | **Weighted median** | 0.29 | 0.10 | 0.80 | 0.017 |  |  |  |  |  |
| **Cancer** | **Ovarian cancer - epithelial** | **Published GWAS** | **MR-Egger** | 0.05 | 0.00 | 0.58 | 0.022 | 0.03 | 0.076 | 26.68 | 0.810 | 0.83 |
| **Cancer** | **Ovarian cancer - epithelial** | **Published GWAS** | **Penalised weighted median** | 0.30 | 0.09 | 0.95 | 0.040 |  |  |  |  |  |
| **Cancer** | **Ovarian cancer - high grade serous** | **Published GWAS** | **Inverse-variance weighted** | 1.04 | 0.51 | 2.10 | 0.924 |  |  | 59.68 | 0.006 |  |
| **Cancer** | **Ovarian cancer - high grade serous** | **Published GWAS** | **Weighted median** | 1.03 | 0.46 | 2.32 | 0.950 |  |  |  |  |  |
| **Cancer** | **Ovarian cancer - high grade serous** | **Published GWAS** | **MR-Egger** | 6.43 | 0.74 | 56.01 | 0.101 | -0.03 | 0.090 | 54.78 | 0.013 | 0.83 |
| **Cancer** | **Ovarian cancer - high grade serous** | **Published GWAS** | **Penalised weighted median** | 0.84 | 0.37 | 1.88 | 0.668 |  |  |  |  |  |
| **Cancer** | **Ovarian cancer - invasive epithelial** | **Published GWAS** | **Inverse-variance weighted** | 1.03 | 0.71 | 1.51 | 0.868 |  |  | 40.51 | 0.240 |  |
| **Cancer** | **Ovarian cancer - invasive epithelial** | **Published GWAS** | **Weighted median** | 0.80 | 0.48 | 1.34 | 0.398 |  |  |  |  |  |
| **Cancer** | **Ovarian cancer - invasive epithelial** | **Published GWAS** | **MR-Egger** | 0.54 | 0.16 | 1.77 | 0.317 | 0.01 | 0.267 | 39.05 | 0.253 | 0.83 |
| **Cancer** | **Ovarian cancer - invasive epithelial** | **Published GWAS** | **Penalised weighted median** | 0.83 | 0.48 | 1.42 | 0.498 |  |  |  |  |  |
| **Cancer** | **Ovarian cancer - low grade** | **Published GWAS** | **Inverse-variance weighted** | 1.31 | 0.36 | 4.81 | 0.686 |  |  | 36.60 | 0.394 |  |
| **Cancer** | **Ovarian cancer - low grade** | **Published GWAS** | **Weighted median** | 0.83 | 0.12 | 5.75 | 0.848 |  |  |  |  |  |
| **Cancer** | **Ovarian cancer - low grade** | **Published GWAS** | **MR-Egger** | 0.02 | 0.00 | 1.35 | 0.079 | 0.06 | 0.043 | 32.38 | 0.547 | 0.83 |
| **Cancer** | **Ovarian cancer - low grade** | **Published GWAS** | **Penalised weighted median** | 0.68 | 0.10 | 4.63 | 0.691 |  |  |  |  |  |
| **Cancer** | **Ovarian cancer - mucinous ovarian carcinoma** | **Published GWAS** | **Inverse-variance weighted** | 1.00 | 0.44 | 2.24 | 0.995 |  |  | 35.84 | 0.429 |  |
| **Cancer** | **Ovarian cancer - mucinous ovarian carcinoma** | **Published GWAS** | **Weighted median** | 1.26 | 0.42 | 3.71 | 0.681 |  |  |  |  |  |
| **Cancer** | **Ovarian cancer - mucinous ovarian carcinoma** | **Published GWAS** | **MR-Egger** | 3.19 | 0.25 | 41.32 | 0.380 | -0.02 | 0.354 | 34.93 | 0.424 | 0.83 |
| **Cancer** | **Ovarian cancer - mucinous ovarian carcinoma** | **Published GWAS** | **Penalised weighted median** | 1.28 | 0.39 | 4.15 | 0.686 |  |  |  |  |  |
| **Cancer** | **Ovarian cancer - serous invasive** | **Published GWAS** | **Inverse-variance weighted** | 1.06 | 0.71 | 1.60 | 0.772 |  |  | 35.43 | 0.448 |  |
| **Cancer** | **Ovarian cancer - serous invasive** | **Published GWAS** | **Weighted median** | 1.05 | 0.57 | 1.96 | 0.871 |  |  |  |  |  |
| **Cancer** | **Ovarian cancer - serous invasive** | **Published GWAS** | **MR-Egger** | 0.61 | 0.17 | 2.24 | 0.466 | 0.01 | 0.388 | 34.65 | 0.437 | 0.83 |
| **Cancer** | **Ovarian cancer - serous invasive** | **Published GWAS** | **Penalised weighted median** | 1.24 | 0.69 | 2.22 | 0.476 |  |  |  |  |  |
| **Cancer** | **Ovarian cancer - serous lowgrade borderline** | **Published GWAS** | **Inverse-variance weighted** | 0.48 | 0.22 | 1.07 | 0.081 |  |  | 37.75 | 0.345 |  |
| **Cancer** | **Ovarian cancer - serous lowgrade borderline** | **Published GWAS** | **Weighted median** | 0.62 | 0.21 | 1.82 | 0.383 |  |  |  |  |  |
| **Cancer** | **Ovarian cancer - serous lowgrade borderline** | **Published GWAS** | **MR-Egger** | 0.03 | 0.00 | 0.29 | 0.006 | 0.04 | 0.014 | 31.48 | 0.592 | 0.83 |
| **Cancer** | **Ovarian cancer - serous lowgrade borderline** | **Published GWAS** | **Penalised weighted median** | 0.72 | 0.24 | 2.16 | 0.553 |  |  |  |  |  |
| **Cancer** | **Pancreatic cancer** | **FinnGen** | **Inverse-variance weighted** | 3.30 | 0.50 | 21.83 | 0.224 |  |  | 46.81 | 0.088 |  |
| **Cancer** | **Pancreatic cancer** | **FinnGen** | **Weighted median** | 1.58 | 0.14 | 18.21 | 0.716 |  |  |  |  |  |
| **Cancer** | **Pancreatic cancer** | **FinnGen** | **MR-Egger** | 26.75 | 0.08 | 8973.70 | 0.276 | -0.03 | 0.460 | 46.05 | 0.081 | 0.83 |
| **Cancer** | **Pancreatic cancer** | **FinnGen** | **Penalised weighted median** | 1.28 | 0.11 | 15.27 | 0.847 |  |  |  |  |  |
| **Cancer** | **Prostate cancer** | **FinnGen** | **Inverse-variance weighted** | 0.91 | 0.47 | 1.76 | 0.785 |  |  | 40.79 | 0.231 |  |
| **Cancer** | **Prostate cancer** | **FinnGen** | **Weighted median** | 0.84 | 0.34 | 2.11 | 0.713 |  |  |  |  |  |
| **Cancer** | **Prostate cancer** | **FinnGen** | **MR-Egger** | 0.58 | 0.08 | 4.40 | 0.603 | 0.01 | 0.647 | 40.54 | 0.204 | 0.84 |
| **Cancer** | **Prostate cancer** | **FinnGen** | **Penalised weighted median** | 0.85 | 0.35 | 2.05 | 0.710 |  |  |  |  |  |
| **Cancer** | **Prostate cancer** | **Published GWAS** | **Inverse-variance weighted** | 0.98 | 0.79 | 1.21 | 0.832 |  |  | 35.44 | 0.447 |  |
| **Cancer** | **Prostate cancer** | **Published GWAS** | **Weighted median** | 0.91 | 0.66 | 1.24 | 0.544 |  |  |  |  |  |
| **Cancer** | **Prostate cancer** | **Published GWAS** | **MR-Egger** | 0.72 | 0.36 | 1.41 | 0.342 | 0.00 | 0.351 | 34.53 | 0.442 | 0.83 |
| **Cancer** | **Prostate cancer** | **Published GWAS** | **Penalised weighted median** | 1.03 | 0.72 | 1.46 | 0.873 |  |  |  |  |  |
| **Cancer** | **Proximal colon cancer** | **Published GWAS** | **Inverse-variance weighted** | 0.79 | 0.51 | 1.22 | 0.289 |  |  | 43.85 | 0.145 |  |
| **Cancer** | **Proximal colon cancer** | **Published GWAS** | **Weighted median** | 0.66 | 0.37 | 1.18 | 0.163 |  |  |  |  |  |
| **Cancer** | **Proximal colon cancer** | **Published GWAS** | **MR-Egger** | 1.60 | 0.40 | 6.41 | 0.508 | -0.01 | 0.297 | 42.44 | 0.152 | 0.82 |
| **Cancer** | **Proximal colon cancer** | **Published GWAS** | **Penalised weighted median** | 0.60 | 0.33 | 1.11 | 0.106 |  |  |  |  |  |
| **Cancer** | **Rectal cancer** | **Published GWAS** | **Inverse-variance weighted** | 1.01 | 0.63 | 1.63 | 0.953 |  |  | 48.80 | 0.061 |  |
| **Cancer** | **Rectal cancer** | **Published GWAS** | **Weighted median** | 0.72 | 0.39 | 1.31 | 0.276 |  |  |  |  |  |
| **Cancer** | **Rectal cancer** | **Published GWAS** | **MR-Egger** | 0.40 | 0.09 | 1.79 | 0.238 | 0.01 | 0.208 | 46.55 | 0.074 | 0.83 |
| **Cancer** | **Rectal cancer** | **Published GWAS** | **Penalised weighted median** | 0.72 | 0.39 | 1.34 | 0.298 |  |  |  |  |  |
| **Cancer** | **Renal cancer** | **FinnGen** | **Inverse-variance weighted** | 1.95 | 0.51 | 7.50 | 0.338 |  |  | 25.34 | 0.885 |  |
| **Cancer** | **Renal cancer** | **FinnGen** | **Weighted median** | 2.02 | 0.28 | 14.31 | 0.482 |  |  |  |  |  |
| **Cancer** | **Renal cancer** | **FinnGen** | **MR-Egger** | 18.64 | 0.31 | 1137.46 | 0.172 | -0.03 | 0.184 | 24.04 | 0.898 | 0.84 |
| **Cancer** | **Renal cancer** | **FinnGen** | **Penalised weighted median** | 1.95 | 0.28 | 13.80 | 0.502 |  |  |  |  |  |
| **Cancer** | **Renal cancer** | **Published GWAS** | **Inverse-variance weighted** | 0.63 | 0.33 | 1.18 | 0.158 |  |  | 54.99 | 0.013 |  |
| **Cancer** | **Renal cancer** | **Published GWAS** | **Weighted median** | 0.48 | 0.22 | 1.05 | 0.066 |  |  |  |  |  |
| **Cancer** | **Renal cancer** | **Published GWAS** | **MR-Egger** | 0.57 | 0.08 | 4.26 | 0.589 | 0.00 | 0.923 | 54.98 | 0.010 | 0.83 |
| **Cancer** | **Renal cancer** | **Published GWAS** | **Penalised weighted median** | 0.48 | 0.22 | 1.07 | 0.072 |  |  |  |  |  |
| **Cancer** | **Thyroid cancer** | **FinnGen** | **Inverse-variance weighted** | 0.91 | 0.24 | 3.40 | 0.888 |  |  | 24.84 | 0.899 |  |
| **Cancer** | **Thyroid cancer** | **FinnGen** | **Weighted median** | 1.45 | 0.23 | 8.98 | 0.689 |  |  |  |  |  |
| **Cancer** | **Thyroid cancer** | **FinnGen** | **MR-Egger** | 1.88 | 0.03 | 105.43 | 0.762 | -0.01 | 0.665 | 24.70 | 0.879 | 0.84 |
| **Cancer** | **Thyroid cancer** | **FinnGen** | **Penalised weighted median** | 1.47 | 0.23 | 9.48 | 0.683 |  |  |  |  |  |

**Supplementary File 1giv. The inverse-variance weighted, weighted median, Egger and penalised weighted median MR analyses for "unfavourable adiposity" using FinnGen and published GWAS. OR: odds ratio; LCI: lower 95% confidence interval; UCI: upper 95% confidence interval; P: p-value; Intercept P: intercept p-value; Q: Q-statistic; I^2^ MR-Egger: I^2^-statistic MR-Egger.**

|  | | | | | | | | | | **Heterogeneity** | | |
| --- | --- | --- | --- | --- | --- | --- | --- | --- | --- | --- | --- | --- |
| **System** | **Disease** | **Study** | **Analysis** | **OR** | **LCI** | **UCI** | **P** | **Egger intercept** | **Intercept P** | **Q** | **P** | **I^2^ MR-Egger** |
| **Cardiovascular and metabolic** | **Abdominal aortic aneurysm** | **FinnGen** | **Inverse-variance weighted** | 1.94 | 0.91 | 4.16 | 0.096 |  |  | 48.58 | 0.039 |  |
| **Cardiovascular and metabolic** | **Abdominal aortic aneurysm** | **FinnGen** | **Weighted median** | 3.42 | 1.33 | 8.79 | 0.011 |  |  |  |  |  |
| **Cardiovascular and metabolic** | **Abdominal aortic aneurysm** | **FinnGen** | **MR-Egger** | 1.13 | 0.08 | 16.19 | 0.928 | 0.01 | 0.680 | 48.32 | 0.032 | 0.95 |
| **Cardiovascular and metabolic** | **Abdominal aortic aneurysm** | **FinnGen** | **Penalised weighted median** | 3.42 | 1.33 | 8.82 | 0.011 |  |  |  |  |  |
| **Cardiovascular and metabolic** | **Abdominal aortic aneurysm** | **Published GWAS** | **Inverse-variance weighted** | 1.03 | 0.87 | 1.22 | 0.732 |  |  | 51.03 | 0.023 |  |
| **Cardiovascular and metabolic** | **Abdominal aortic aneurysm** | **Published GWAS** | **Weighted median** | 0.96 | 0.78 | 1.17 | 0.676 |  |  |  |  |  |
| **Cardiovascular and metabolic** | **Abdominal aortic aneurysm** | **Published GWAS** | **MR-Egger** | 0.78 | 0.43 | 1.40 | 0.415 | 0.01 | 0.343 | 49.59 | 0.024 | 0.93 |
| **Cardiovascular and metabolic** | **Abdominal aortic aneurysm** | **Published GWAS** | **Penalised weighted median** | 0.98 | 0.79 | 1.21 | 0.821 |  |  |  |  |  |
| **Cardiovascular and metabolic** | **Atrial fibrillation** | **FinnGen** | **Inverse-variance weighted** | 2.07 | 1.43 | 2.99 | 5.E-04 |  |  | 50.30 | 0.027 |  |
| **Cardiovascular and metabolic** | **Atrial fibrillation** | **FinnGen** | **Weighted median** | 2.18 | 1.41 | 3.39 | 5.E-04 |  |  |  |  |  |
| **Cardiovascular and metabolic** | **Atrial fibrillation** | **FinnGen** | **MR-Egger** | 2.55 | 0.70 | 9.30 | 0.166 | 0.00 | 0.741 | 50.13 | 0.022 | 0.96 |
| **Cardiovascular and metabolic** | **Atrial fibrillation** | **FinnGen** | **Penalised weighted median** | 1.57 | 0.97 | 2.53 | 0.065 |  |  |  |  |  |
| **Cardiovascular and metabolic** | **Atrial fibrillation** | **Published GWAS** | **Inverse-variance weighted** | 1.71 | 1.41 | 2.08 | 6.E-06 |  |  | 72.35 | 9.E-05 |  |
| **Cardiovascular and metabolic** | **Atrial fibrillation** | **Published GWAS** | **Weighted median** | 1.77 | 1.44 | 2.19 | 1.E-07 |  |  |  |  |  |
| **Cardiovascular and metabolic** | **Atrial fibrillation** | **Published GWAS** | **MR-Egger** | 1.45 | 0.75 | 2.78 | 0.279 | 0.00 | 0.601 | 71.73 | 7.E-05 | 0.93 |
| **Cardiovascular and metabolic** | **Atrial fibrillation** | **Published GWAS** | **Penalised weighted median** | 1.48 | 1.18 | 1.85 | 6.E-04 |  |  |  |  |  |
| **Cardiovascular and metabolic** | **Cardioembolic stroke** | **Published GWAS** | **Inverse-variance weighted** | 1.29 | 0.84 | 1.97 | 0.250 |  |  | 47.03 | 0.054 |  |
| **Cardiovascular and metabolic** | **Cardioembolic stroke** | **Published GWAS** | **Weighted median** | 1.23 | 0.69 | 2.17 | 0.481 |  |  |  |  |  |
| **Cardiovascular and metabolic** | **Cardioembolic stroke** | **Published GWAS** | **MR-Egger** | 0.91 | 0.21 | 3.84 | 0.896 | 0.01 | 0.621 | 46.67 | 0.045 | 0.93 |
| **Cardiovascular and metabolic** | **Cardioembolic stroke** | **Published GWAS** | **Penalised weighted median** | 1.22 | 0.68 | 2.22 | 0.504 |  |  |  |  |  |
| **Cardiovascular and metabolic** | **Cardiovascular disease** | **FinnGen** | **Inverse-variance weighted** | 1.98 | 1.55 | 2.53 | 5.E-06 |  |  | 77.28 | 2.E-05 |  |
| **Cardiovascular and metabolic** | **Cardiovascular disease** | **FinnGen** | **Weighted median** | 2.06 | 1.59 | 2.66 | 4.E-08 |  |  |  |  |  |
| **Cardiovascular and metabolic** | **Cardiovascular disease** | **FinnGen** | **MR-Egger** | 3.00 | 1.29 | 6.98 | 0.016 | -0.01 | 0.323 | 74.92 | 3.E-05 | 0.95 |
| **Cardiovascular and metabolic** | **Cardiovascular disease** | **FinnGen** | **Penalised weighted median** | 1.72 | 1.32 | 2.24 | 7.E-05 |  |  |  |  |  |
| **Cardiovascular and metabolic** | **Coronary artery disease** | **Published GWAS** | **Inverse-variance weighted** | 2.06 | 1.58 | 2.68 | 6.E-06 |  |  | 74.39 | 5.E-05 |  |
| **Cardiovascular and metabolic** | **Coronary artery disease** | **Published GWAS** | **Weighted median** | 2.04 | 1.54 | 2.72 | 9.E-07 |  |  |  |  |  |
| **Cardiovascular and metabolic** | **Coronary artery disease** | **Published GWAS** | **MR-Egger** | 2.00 | 0.81 | 4.95 | 0.143 | 0.00 | 0.949 | 74.38 | 3.E-05 | 0.94 |
| **Cardiovascular and metabolic** | **Coronary artery disease** | **Published GWAS** | **Penalised weighted median** | 1.67 | 1.24 | 2.24 | 6.E-04 |  |  |  |  |  |
| **Cardiovascular and metabolic** | **Deep vein thrombosis** | **FinnGen** | **Inverse-variance weighted** | 2.05 | 1.15 | 3.64 | 0.020 |  |  | 52.11 | 0.018 |  |
| **Cardiovascular and metabolic** | **Deep vein thrombosis** | **FinnGen** | **Weighted median** | 2.23 | 1.11 | 4.48 | 0.024 |  |  |  |  |  |
| **Cardiovascular and metabolic** | **Deep vein thrombosis** | **FinnGen** | **MR-Egger** | 3.54 | 0.47 | 26.38 | 0.227 | -0.01 | 0.581 | 51.61 | 0.015 | 0.95 |
| **Cardiovascular and metabolic** | **Deep vein thrombosis** | **FinnGen** | **Penalised weighted median** | 1.63 | 0.80 | 3.32 | 0.176 |  |  |  |  |  |
| **Cardiovascular and metabolic** | **Heart failure** | **FinnGen** | **Inverse-variance weighted** | 2.57 | 1.82 | 3.64 | 7.E-06 |  |  | 41.44 | 0.149 |  |
| **Cardiovascular and metabolic** | **Heart failure** | **FinnGen** | **Weighted median** | 2.65 | 1.64 | 4.31 | 8.E-05 |  |  |  |  |  |
| **Cardiovascular and metabolic** | **Heart failure** | **FinnGen** | **MR-Egger** | 1.77 | 0.53 | 5.92 | 0.364 | 0.01 | 0.529 | 40.92 | 0.134 | 0.95 |
| **Cardiovascular and metabolic** | **Heart failure** | **FinnGen** | **Penalised weighted median** | 2.65 | 1.61 | 4.34 | 1.E-04 |  |  |  |  |  |
| **Cardiovascular and metabolic** | **Heart failure** | **Published GWAS** | **Inverse-variance weighted** | 2.14 | 1.63 | 2.80 | 4.E-06 |  |  | 108.12 | 6.E-10 |  |
| **Cardiovascular and metabolic** | **Heart failure** | **Published GWAS** | **Weighted median** | 2.35 | 1.82 | 3.03 | 4.E-11 |  |  |  |  |  |
| **Cardiovascular and metabolic** | **Heart failure** | **Published GWAS** | **MR-Egger** | 2.17 | 0.87 | 5.40 | 0.106 | 0.00 | 0.974 | 108.11 | 3.E-10 | 0.95 |
| **Cardiovascular and metabolic** | **Heart failure** | **Published GWAS** | **Penalised weighted median** | 1.67 | 1.24 | 2.24 | 7.E-04 |  |  |  |  |  |
| **Cardiovascular and metabolic** | **Hypertension** | **FinnGen** | **Inverse-variance weighted** | 3.03 | 2.18 | 4.22 | 2.E-07 |  |  | 82.53 | 4.E-06 |  |
| **Cardiovascular and metabolic** | **Hypertension** | **FinnGen** | **Weighted median** | 3.12 | 2.22 | 4.39 | 5.E-11 |  |  |  |  |  |
| **Cardiovascular and metabolic** | **Hypertension** | **FinnGen** | **MR-Egger** | 3.19 | 1.00 | 10.19 | 0.059 | 0.00 | 0.930 | 82.51 | 2.E-06 | 0.95 |
| **Cardiovascular and metabolic** | **Hypertension** | **FinnGen** | **Penalised weighted median** | 2.04 | 1.40 | 2.99 | 2.E-04 |  |  |  |  |  |
| **Cardiovascular and metabolic** | **Ischemic heart disease** | **FinnGen** | **Inverse-variance weighted** | 1.33 | 1.02 | 1.73 | 0.041 |  |  | 46.23 | 0.063 |  |
| **Cardiovascular and metabolic** | **Ischemic heart disease** | **FinnGen** | **Weighted median** | 1.56 | 1.11 | 2.21 | 0.011 |  |  |  |  |  |
| **Cardiovascular and metabolic** | **Ischemic heart disease** | **FinnGen** | **MR-Egger** | 1.40 | 0.55 | 3.55 | 0.481 | 0.00 | 0.910 | 46.21 | 0.050 | 0.95 |
| **Cardiovascular and metabolic** | **Ischemic heart disease** | **FinnGen** | **Penalised weighted median** | 1.56 | 1.10 | 2.21 | 0.013 |  |  |  |  |  |
| **Cardiovascular and metabolic** | **Ischemic stroke** | **FinnGen** | **Inverse-variance weighted** | 1.64 | 1.18 | 2.29 | 0.006 |  |  | 37.64 | 0.265 |  |
| **Cardiovascular and metabolic** | **Ischemic stroke** | **FinnGen** | **Weighted median** | 1.51 | 0.92 | 2.47 | 0.102 |  |  |  |  |  |
| **Cardiovascular and metabolic** | **Ischemic stroke** | **FinnGen** | **MR-Egger** | 0.73 | 0.24 | 2.26 | 0.590 | 0.02 | 0.153 | 35.28 | 0.316 | 0.95 |
| **Cardiovascular and metabolic** | **Ischemic stroke** | **FinnGen** | **Penalised weighted median** | 1.54 | 0.93 | 2.55 | 0.096 |  |  |  |  |  |
| **Cardiovascular and metabolic** | **Ischemic stroke** | **Published GWAS** | **Inverse-variance weighted** | 1.48 | 1.21 | 1.81 | 5.E-04 |  |  | 38.53 | 0.233 |  |
| **Cardiovascular and metabolic** | **Ischemic stroke** | **Published GWAS** | **Weighted median** | 1.25 | 0.96 | 1.64 | 0.102 |  |  |  |  |  |
| **Cardiovascular and metabolic** | **Ischemic stroke** | **Published GWAS** | **MR-Egger** | 0.77 | 0.41 | 1.45 | 0.422 | 0.01 | 0.041 | 33.73 | 0.384 | 0.93 |
| **Cardiovascular and metabolic** | **Ischemic stroke** | **Published GWAS** | **Penalised weighted median** | 1.26 | 0.96 | 1.65 | 0.099 |  |  |  |  |  |
| **Cardiovascular and metabolic** | **Large artery stroke** | **Published GWAS** | **Inverse-variance weighted** | 1.98 | 1.13 | 3.47 | 0.023 |  |  | 48.96 | 0.036 |  |
| **Cardiovascular and metabolic** | **Large artery stroke** | **Published GWAS** | **Weighted median** | 2.28 | 1.11 | 4.69 | 0.024 |  |  |  |  |  |
| **Cardiovascular and metabolic** | **Large artery stroke** | **Published GWAS** | **MR-Egger** | 1.14 | 0.17 | 7.77 | 0.892 | 0.01 | 0.561 | 48.44 | 0.031 | 0.93 |
| **Cardiovascular and metabolic** | **Large artery stroke** | **Published GWAS** | **Penalised weighted median** | 2.18 | 1.04 | 4.58 | 0.039 |  |  |  |  |  |
| **Cardiovascular and metabolic** | **Peripheral artery disease** | **FinnGen** | **Inverse-variance weighted** | 3.31 | 2.09 | 5.24 | 1.E-05 |  |  | 45.60 | 0.071 |  |
| **Cardiovascular and metabolic** | **Peripheral artery disease** | **FinnGen** | **Weighted median** | 2.60 | 1.42 | 4.73 | 0.002 |  |  |  |  |  |
| **Cardiovascular and metabolic** | **Peripheral artery disease** | **FinnGen** | **MR-Egger** | 2.40 | 0.48 | 11.94 | 0.295 | 0.01 | 0.683 | 45.36 | 0.059 | 0.95 |
| **Cardiovascular and metabolic** | **Peripheral artery disease** | **FinnGen** | **Penalised weighted median** | 2.24 | 1.17 | 4.30 | 0.015 |  |  |  |  |  |
| **Cardiovascular and metabolic** | **Pulmonary embolism** | **FinnGen** | **Inverse-variance weighted** | 1.66 | 0.94 | 2.94 | 0.090 |  |  | 43.72 | 0.100 |  |
| **Cardiovascular and metabolic** | **Pulmonary embolism** | **FinnGen** | **Weighted median** | 1.86 | 0.85 | 4.09 | 0.121 |  |  |  |  |  |
| **Cardiovascular and metabolic** | **Pulmonary embolism** | **FinnGen** | **MR-Egger** | 1.89 | 0.25 | 13.99 | 0.539 | 0.00 | 0.898 | 43.70 | 0.081 | 0.95 |
| **Cardiovascular and metabolic** | **Pulmonary embolism** | **FinnGen** | **Penalised weighted median** | 1.88 | 0.84 | 4.20 | 0.126 |  |  |  |  |  |
| **Cardiovascular and metabolic** | **Small vessel stroke** | **Published GWAS** | **Inverse-variance weighted** | 1.64 | 0.92 | 2.92 | 0.104 |  |  | 61.01 | 0.002 |  |
| **Cardiovascular and metabolic** | **Small vessel stroke** | **Published GWAS** | **Weighted median** | 1.46 | 0.72 | 2.95 | 0.296 |  |  |  |  |  |
| **Cardiovascular and metabolic** | **Small vessel stroke** | **Published GWAS** | **MR-Egger** | 0.85 | 0.12 | 6.11 | 0.869 | 0.01 | 0.498 | 60.13 | 0.002 | 0.93 |
| **Cardiovascular and metabolic** | **Small vessel stroke** | **Published GWAS** | **Penalised weighted median** | 1.46 | 0.72 | 2.96 | 0.299 |  |  |  |  |  |
| **Cardiovascular and metabolic** | **Stroke** | **FinnGen** | **Inverse-variance weighted** | 1.42 | 1.09 | 1.85 | 0.014 |  |  | 30.57 | 0.589 |  |
| **Cardiovascular and metabolic** | **Stroke** | **FinnGen** | **Weighted median** | 1.23 | 0.81 | 1.86 | 0.324 |  |  |  |  |  |
| **Cardiovascular and metabolic** | **Stroke** | **FinnGen** | **MR-Egger** | 0.79 | 0.32 | 1.98 | 0.625 | 0.01 | 0.180 | 28.88 | 0.625 | 0.95 |
| **Cardiovascular and metabolic** | **Stroke** | **FinnGen** | **Penalised weighted median** | 1.25 | 0.83 | 1.90 | 0.290 |  |  |  |  |  |
| **Cardiovascular and metabolic** | **Stroke** | **Published GWAS** | **Inverse-variance weighted** | 1.44 | 1.20 | 1.73 | 5.E-04 |  |  | 37.96 | 0.254 |  |
| **Cardiovascular and metabolic** | **Stroke** | **Published GWAS** | **Weighted median** | 1.22 | 0.94 | 1.58 | 0.142 |  |  |  |  |  |
| **Cardiovascular and metabolic** | **Stroke** | **Published GWAS** | **MR-Egger** | 0.70 | 0.39 | 1.25 | 0.237 | 0.02 | 0.015 | 31.44 | 0.495 | 0.93 |
| **Cardiovascular and metabolic** | **Stroke** | **Published GWAS** | **Penalised weighted median** | 1.25 | 0.96 | 1.62 | 0.094 |  |  |  |  |  |
| **Cardiovascular and metabolic** | **Stroke (excl. subarachnoid hemorrhage)** | **FinnGen** | **Inverse-variance weighted** | 1.56 | 1.14 | 2.13 | 0.008 |  |  | 35.61 | 0.346 |  |
| **Cardiovascular and metabolic** | **Stroke (excl. subarachnoid hemorrhage)** | **FinnGen** | **Weighted median** | 1.52 | 0.93 | 2.48 | 0.095 |  |  |  |  |  |
| **Cardiovascular and metabolic** | **Stroke (excl. subarachnoid hemorrhage)** | **FinnGen** | **MR-Egger** | 0.71 | 0.25 | 2.05 | 0.536 | 0.02 | 0.139 | 33.22 | 0.408 | 0.95 |
| **Cardiovascular and metabolic** | **Stroke (excl. subarachnoid hemorrhage)** | **FinnGen** | **Penalised weighted median** | 1.52 | 0.92 | 2.51 | 0.101 |  |  |  |  |  |
| **Cardiovascular and metabolic** | **Venous thromboembolism** | **FinnGen** | **Inverse-variance weighted** | 1.71 | 1.03 | 2.84 | 0.046 |  |  | 75.34 | 4.E-05 |  |
| **Cardiovascular and metabolic** | **Venous thromboembolism** | **FinnGen** | **Weighted median** | 2.08 | 1.23 | 3.52 | 0.006 |  |  |  |  |  |
| **Cardiovascular and metabolic** | **Venous thromboembolism** | **FinnGen** | **MR-Egger** | 3.00 | 0.51 | 17.57 | 0.231 | -0.01 | 0.518 | 74.35 | 3.E-05 | 0.95 |
| **Cardiovascular and metabolic** | **Venous thromboembolism** | **FinnGen** | **Penalised weighted median** | 1.85 | 1.05 | 3.24 | 0.033 |  |  |  |  |  |
| **Cardiovascular and metabolic** | **Venous thromboembolism** | **Published GWAS** | **Inverse-variance weighted** | 1.60 | 1.17 | 2.19 | 0.006 |  |  | 43.78 | 0.099 |  |
| **Cardiovascular and metabolic** | **Venous thromboembolism** | **Published GWAS** | **Weighted median** | 1.92 | 1.27 | 2.90 | 0.002 |  |  |  |  |  |
| **Cardiovascular and metabolic** | **Venous thromboembolism** | **Published GWAS** | **MR-Egger** | 3.10 | 1.05 | 9.10 | 0.048 | -0.01 | 0.219 | 41.73 | 0.117 | 0.95 |
| **Cardiovascular and metabolic** | **Venous thromboembolism** | **Published GWAS** | **Penalised weighted median** | 1.81 | 1.21 | 2.70 | 0.004 |  |  |  |  |  |
| **Cardiovascular and metabolic** | **Polycystic ovary syndrome** | **FinnGen** | **Inverse-variance weighted** | 10.15 | 2.95 | 34.93 | 8.E-04 |  |  | 24.35 | 0.862 |  |
| **Cardiovascular and metabolic** | **Polycystic ovary syndrome** | **FinnGen** | **Weighted median** | 5.22 | 0.82 | 33.02 | 0.079 |  |  |  |  |  |
| **Cardiovascular and metabolic** | **Polycystic ovary syndrome** | **FinnGen** | **MR-Egger** | 14.05 | 0.20 | 1005.02 | 0.234 | -0.01 | 0.859 | 24.33 | 0.832 | 0.95 |
| **Cardiovascular and metabolic** | **Polycystic ovary syndrome** | **FinnGen** | **Penalised weighted median** | 2.88 | 0.45 | 18.45 | 0.263 |  |  |  |  |  |
| **Cardiovascular and metabolic** | **Polycystic ovary syndrome** | **Published GWAS** | **Inverse-variance weighted** | 6.17 | 2.80 | 13.63 | 8.E-05 |  |  | 62.13 | 0.002 |  |
| **Cardiovascular and metabolic** | **Polycystic ovary syndrome** | **Published GWAS** | **Weighted median** | 5.69 | 2.26 | 14.32 | 2.E-04 |  |  |  |  |  |
| **Cardiovascular and metabolic** | **Polycystic ovary syndrome** | **Published GWAS** | **MR-Egger** | 17.79 | 1.18 | 268.70 | 0.046 | -0.02 | 0.430 | 60.91 | 0.002 | 0.93 |
| **Cardiovascular and metabolic** | **Polycystic ovary syndrome** | **Published GWAS** | **Penalised weighted median** | 3.59 | 1.39 | 9.26 | 0.008 |  |  |  |  |  |
| **Cardiovascular and metabolic** | **Type 2 diabetes** | **FinnGen** | **Inverse-variance weighted** | 5.65 | 3.81 | 8.38 | 6.E-10 |  |  | 107.82 | 7.E-10 |  |
| **Cardiovascular and metabolic** | **Type 2 diabetes** | **FinnGen** | **Weighted median** | 4.93 | 3.34 | 7.25 | 7.E-16 |  |  |  |  |  |
| **Cardiovascular and metabolic** | **Type 2 diabetes** | **FinnGen** | **MR-Egger** | 13.26 | 3.44 | 51.16 | 7.E-04 | -0.02 | 0.205 | 102.45 | 3.E-09 | 0.95 |
| **Cardiovascular and metabolic** | **Type 2 diabetes** | **FinnGen** | **Penalised weighted median** | 3.15 | 2.12 | 4.69 | 2.E-08 |  |  |  |  |  |
| **Cardiovascular and metabolic** | **Type 2 diabetes** | **Published GWAS** | **Inverse-variance weighted** | 5.40 | 3.92 | 7.43 | 7.E-12 |  |  | 142.25 | 2.E-15 |  |
| **Cardiovascular and metabolic** | **Type 2 diabetes** | **Published GWAS** | **Weighted median** | 5.19 | 3.91 | 6.90 | 6.E-30 |  |  |  |  |  |
| **Cardiovascular and metabolic** | **Type 2 diabetes** | **Published GWAS** | **MR-Egger** | 11.69 | 4.15 | 32.92 | 5.E-05 | -0.02 | 0.135 | 132.49 | 3.E-14 | 0.93 |
| **Cardiovascular and metabolic** | **Type 2 diabetes** | **Published GWAS** | **Penalised weighted median** | 2.25 | 1.51 | 3.35 | 6.E-05 |  |  |  |  |  |
| **Cardiovascular and metabolic** | **Chronic kidney disease** | **FinnGen** | **Inverse-variance weighted** | 1.53 | 0.88 | 2.69 | 0.144 |  |  | 39.62 | 0.199 |  |
| **Cardiovascular and metabolic** | **Chronic kidney disease** | **FinnGen** | **Weighted median** | 1.43 | 0.66 | 3.09 | 0.362 |  |  |  |  |  |
| **Cardiovascular and metabolic** | **Chronic kidney disease** | **FinnGen** | **MR-Egger** | 6.31 | 0.95 | 42.09 | 0.066 | -0.03 | 0.137 | 36.94 | 0.251 | 0.95 |
| **Cardiovascular and metabolic** | **Chronic kidney disease** | **FinnGen** | **Penalised weighted median** | 0.96 | 0.43 | 2.14 | 0.915 |  |  |  |  |  |
| **Cardiovascular and metabolic** | **Chronic kidney disease** | **Published GWAS** | **Inverse-variance weighted** | 1.14 | 0.92 | 1.42 | 0.227 |  |  | 49.96 | 0.029 |  |
| **Cardiovascular and metabolic** | **Chronic kidney disease** | **Published GWAS** | **Weighted median** | 1.34 | 1.03 | 1.73 | 0.030 |  |  |  |  |  |
| **Cardiovascular and metabolic** | **Chronic kidney disease** | **Published GWAS** | **MR-Egger** | 1.59 | 0.79 | 3.21 | 0.204 | -0.01 | 0.339 | 48.54 | 0.031 | 0.94 |
| **Cardiovascular and metabolic** | **Chronic kidney disease** | **Published GWAS** | **Penalised weighted median** | 1.41 | 1.05 | 1.87 | 0.020 |  |  |  |  |  |
| **Musculoskeletal** | **Gout** | **FinnGen** | **Inverse-variance weighted** | 2.13 | 1.20 | 3.81 | 0.015 |  |  | 37.10 | 0.286 |  |
| **Musculoskeletal** | **Gout** | **FinnGen** | **Weighted median** | 2.27 | 0.96 | 5.39 | 0.062 |  |  |  |  |  |
| **Musculoskeletal** | **Gout** | **FinnGen** | **MR-Egger** | 1.99 | 0.26 | 15.16 | 0.513 | 0.00 | 0.943 | 37.09 | 0.246 | 0.95 |
| **Musculoskeletal** | **Gout** | **FinnGen** | **Penalised weighted median** | 2.27 | 0.98 | 5.24 | 0.055 |  |  |  |  |  |
| **Musculoskeletal** | **Gout** | **Published GWAS** | **Inverse-variance weighted** | 2.61 | 1.89 | 3.58 | 1.E-06 |  |  | 54.36 | 0.011 |  |
| **Musculoskeletal** | **Gout** | **Published GWAS** | **Weighted median** | 2.61 | 1.74 | 3.94 | 4.E-06 |  |  |  |  |  |
| **Musculoskeletal** | **Gout** | **Published GWAS** | **MR-Egger** | 2.88 | 0.97 | 8.55 | 0.066 | 0.00 | 0.852 | 54.30 | 0.008 | 0.97 |
| **Musculoskeletal** | **Gout** | **Published GWAS** | **Penalised weighted median** | 2.53 | 1.66 | 3.84 | 1.E-05 |  |  |  |  |  |
| **Musculoskeletal** | **Osteoarthritis** | **FinnGen** | **Inverse-variance weighted** | 2.62 | 1.93 | 3.55 | 5.E-07 |  |  | 77.25 | 2.E-05 |  |
| **Musculoskeletal** | **Osteoarthritis** | **FinnGen** | **Weighted median** | 2.75 | 1.95 | 3.86 | 6.E-09 |  |  |  |  |  |
| **Musculoskeletal** | **Osteoarthritis** | **FinnGen** | **MR-Egger** | 8.80 | 3.33 | 23.28 | 1.E-04 | -0.03 | 0.016 | 64.18 | 6.E-04 | 0.95 |
| **Musculoskeletal** | **Osteoarthritis** | **FinnGen** | **Penalised weighted median** | 1.62 | 1.14 | 2.31 | 0.008 |  |  |  |  |  |
| **Musculoskeletal** | **Osteoarthritis** | **Published GWAS** | **Inverse-variance weighted** | 1.94 | 1.59 | 2.36 | 1.E-07 |  |  | 155.03 | 2.E-16 |  |
| **Musculoskeletal** | **Osteoarthritis** | **Published GWAS** | **Weighted median** | 1.96 | 1.67 | 2.30 | 1.E-16 |  |  |  |  |  |
| **Musculoskeletal** | **Osteoarthritis** | **Published GWAS** | **MR-Egger** | 2.06 | 1.03 | 4.12 | 0.049 | 0.00 | 0.856 | 154.88 | 1.E-16 | 0.92 |
| **Musculoskeletal** | **Osteoarthritis** | **Published GWAS** | **Penalised weighted median** | 1.91 | 1.57 | 2.33 | 1.E-10 |  |  |  |  |  |
| **Musculoskeletal** | **Osteoarthritis (hip and/or knee)** | **Published GWAS** | **Inverse-variance weighted** | 2.15 | 1.67 | 2.76 | 6.E-07 |  |  | 125.62 | 1.E-11 |  |
| **Musculoskeletal** | **Osteoarthritis (hip and/or knee)** | **Published GWAS** | **Weighted median** | 2.68 | 2.16 | 3.34 | 8.E-19 |  |  |  |  |  |
| **Musculoskeletal** | **Osteoarthritis (hip and/or knee)** | **Published GWAS** | **MR-Egger** | 2.96 | 1.26 | 6.97 | 0.018 | -0.01 | 0.448 | 123.60 | 2.E-11 | 0.92 |
| **Musculoskeletal** | **Osteoarthritis (hip and/or knee)** | **Published GWAS** | **Penalised weighted median** | 1.80 | 1.36 | 2.38 | 4.E-05 |  |  |  |  |  |
| **Musculoskeletal** | **Osteoarthritis (hip)** | **FinnGen** | **Inverse-variance weighted** | 2.36 | 1.55 | 3.61 | 4.E-04 |  |  | 55.40 | 0.009 |  |
| **Musculoskeletal** | **Osteoarthritis (hip)** | **FinnGen** | **Weighted median** | 2.83 | 1.70 | 4.72 | 7.E-05 |  |  |  |  |  |
| **Musculoskeletal** | **Osteoarthritis (hip)** | **FinnGen** | **MR-Egger** | 8.64 | 2.10 | 35.56 | 0.005 | -0.03 | 0.070 | 49.92 | 0.023 | 0.95 |
| **Musculoskeletal** | **Osteoarthritis (hip)** | **FinnGen** | **Penalised weighted median** | 2.23 | 1.31 | 3.81 | 0.003 |  |  |  |  |  |
| **Musculoskeletal** | **Osteoarthritis (knee)** | **FinnGen** | **Inverse-variance weighted** | 3.26 | 2.27 | 4.68 | 3.E-07 |  |  | 77.58 | 2.E-05 |  |
| **Musculoskeletal** | **Osteoarthritis (knee)** | **FinnGen** | **Weighted median** | 3.34 | 2.26 | 4.94 | 1.E-09 |  |  |  |  |  |
| **Musculoskeletal** | **Osteoarthritis (knee)** | **FinnGen** | **MR-Egger** | 11.21 | 3.41 | 36.85 | 4.E-04 | -0.03 | 0.041 | 67.98 | 2.E-04 | 0.95 |
| **Musculoskeletal** | **Osteoarthritis (knee)** | **FinnGen** | **Penalised weighted median** | 2.11 | 1.36 | 3.26 | 8.E-04 |  |  |  |  |  |
| **Musculoskeletal** | **Osteoporosis** | **FinnGen** | **Inverse-variance weighted** | 0.95 | 0.55 | 1.65 | 0.868 |  |  | 21.34 | 0.941 |  |
| **Musculoskeletal** | **Osteoporosis** | **FinnGen** | **Weighted median** | 0.81 | 0.37 | 1.79 | 0.604 |  |  |  |  |  |
| **Musculoskeletal** | **Osteoporosis** | **FinnGen** | **MR-Egger** | 0.40 | 0.06 | 2.70 | 0.356 | 0.02 | 0.255 | 20.48 | 0.942 | 0.95 |
| **Musculoskeletal** | **Osteoporosis** | **FinnGen** | **Penalised weighted median** | 0.81 | 0.36 | 1.84 | 0.615 |  |  |  |  |  |
| **Musculoskeletal** | **Osteoporosis** | **Published GWAS** | **Inverse-variance weighted** | 1.20 | 1.02 | 1.40 | 0.031 |  |  | 691.84 | 3.E-124 |  |
| **Musculoskeletal** | **Osteoporosis** | **Published GWAS** | **Weighted median** | 1.35 | 1.24 | 1.46 | 4.E-13 |  |  |  |  |  |
| **Musculoskeletal** | **Osteoporosis** | **Published GWAS** | **MR-Egger** | 1.70 | 1.03 | 2.82 | 0.047 | -0.01 | 0.159 | 649.61 | 3.E-116 | 0.93 |
| **Musculoskeletal** | **Osteoporosis** | **Published GWAS** | **Penalised weighted median** | 1.34 | 1.19 | 1.52 | 3.E-06 |  |  |  |  |  |
| **Musculoskeletal** | **Rheumatoid arthritis** | **FinnGen** | **Inverse-variance weighted** | 1.82 | 1.11 | 2.96 | 0.023 |  |  | 46.35 | 0.062 |  |
| **Musculoskeletal** | **Rheumatoid arthritis** | **FinnGen** | **Weighted median** | 1.60 | 0.85 | 3.02 | 0.145 |  |  |  |  |  |
| **Musculoskeletal** | **Rheumatoid arthritis** | **FinnGen** | **MR-Egger** | 2.64 | 0.48 | 14.59 | 0.275 | -0.01 | 0.658 | 46.06 | 0.051 | 0.96 |
| **Musculoskeletal** | **Rheumatoid arthritis** | **FinnGen** | **Penalised weighted median** | 1.57 | 0.81 | 3.07 | 0.185 |  |  |  |  |  |
| **Musculoskeletal** | **Rheumatoid arthritis** | **Published GWAS** | **Inverse-variance weighted** | 1.43 | 0.89 | 2.32 | 0.152 |  |  | 71.76 | 1.E-04 |  |
| **Musculoskeletal** | **Rheumatoid arthritis** | **Published GWAS** | **Weighted median** | 1.00 | 0.57 | 1.74 | 1.000 |  |  |  |  |  |
| **Musculoskeletal** | **Rheumatoid arthritis** | **Published GWAS** | **MR-Egger** | 1.78 | 0.39 | 8.05 | 0.460 | 0.00 | 0.767 | 71.56 | 7.E-05 | 0.95 |
| **Musculoskeletal** | **Rheumatoid arthritis** | **Published GWAS** | **Penalised weighted median** | 1.83 | 1.01 | 3.32 | 0.045 |  |  |  |  |  |
| **Gastrointestinal** | **Gallstones** | **FinnGen** | **Inverse-variance weighted** | 2.55 | 1.88 | 3.45 | 9.E-07 |  |  | 50.02 | 0.029 |  |
| **Gastrointestinal** | **Gallstones** | **FinnGen** | **Weighted median** | 2.75 | 1.86 | 4.05 | 3.E-07 |  |  |  |  |  |
| **Gastrointestinal** | **Gallstones** | **FinnGen** | **MR-Egger** | 6.05 | 2.18 | 16.79 | 0.002 | -0.02 | 0.093 | 45.72 | 0.055 | 0.95 |
| **Gastrointestinal** | **Gallstones** | **FinnGen** | **Penalised weighted median** | 2.18 | 1.47 | 3.23 | 1.E-04 |  |  |  |  |  |
| **Gastrointestinal** | **Gastro-oesophageal reflux disease** | **Published GWAS** | **Inverse-variance weighted** | 1.37 | 1.12 | 1.68 | 0.004 |  |  | 140.92 | 5.E-14 |  |
| **Gastrointestinal** | **Gastro-oesophageal reflux disease** | **Published GWAS** | **Weighted median** | 1.23 | 1.03 | 1.47 | 0.020 |  |  |  |  |  |
| **Gastrointestinal** | **Gastro-oesophageal reflux disease** | **Published GWAS** | **MR-Egger** | 0.91 | 0.46 | 1.82 | 0.790 | 0.01 | 0.230 | 135.32 | 2.E-13 | 0.92 |
| **Gastrointestinal** | **Gastro-oesophageal reflux disease** | **Published GWAS** | **Penalised weighted median** | 1.22 | 1.00 | 1.49 | 0.055 |  |  |  |  |  |
| **Nervous** | **Alzheimer's disease** | **FinnGen** | **Inverse-variance weighted** | 1.29 | 0.76 | 2.18 | 0.346 |  |  | 27.81 | 0.723 |  |
| **Nervous** | **Alzheimer's disease** | **FinnGen** | **Weighted median** | 1.03 | 0.48 | 2.18 | 0.949 |  |  |  |  |  |
| **Nervous** | **Alzheimer's disease** | **FinnGen** | **MR-Egger** | 0.67 | 0.11 | 4.13 | 0.670 | 0.01 | 0.430 | 27.26 | 0.705 | 0.96 |
| **Nervous** | **Alzheimer's disease** | **FinnGen** | **Penalised weighted median** | 1.02 | 0.49 | 2.12 | 0.964 |  |  |  |  |  |
| **Nervous** | **Alzheimer's disease** | **Published GWAS (1)** | **Inverse-variance weighted** | 1.02 | 0.98 | 1.06 | 0.380 |  |  | 36.35 | 0.315 |  |
| **Nervous** | **Alzheimer's disease** | **Published GWAS (1)** | **Weighted median** | 1.04 | 0.98 | 1.11 | 0.168 |  |  |  |  |  |
| **Nervous** | **Alzheimer's disease** | **Published GWAS (1)** | **MR-Egger** | 1.09 | 0.94 | 1.26 | 0.248 | 0.00 | 0.347 | 35.34 | 0.313 | 0.99 |
| **Nervous** | **Alzheimer's disease** | **Published GWAS (1)** | **Penalised weighted median** | 1.09 | 0.99 | 1.20 | 0.085 |  |  |  |  |  |
| **Nervous** | **Alzheimer's disease** | **Published GWAS (2)** | **Inverse-variance weighted** | 0.70 | 0.52 | 0.95 | 0.030 |  |  | 43.10 | 0.112 |  |
| **Nervous** | **Alzheimer's disease** | **Published GWAS (2)** | **Weighted median** | 0.76 | 0.52 | 1.11 | 0.155 |  |  |  |  |  |
| **Nervous** | **Alzheimer's disease** | **Published GWAS (2)** | **MR-Egger** | 0.93 | 0.33 | 2.61 | 0.888 | -0.01 | 0.589 | 42.70 | 0.098 | 0.93 |
| **Nervous** | **Alzheimer's disease** | **Published GWAS (2)** | **Penalised weighted median** | 0.89 | 0.59 | 1.34 | 0.587 |  |  |  |  |  |
| **Nervous** | **Depression** | **FinnGen** | **Inverse-variance weighted** | 0.90 | 0.68 | 1.20 | 0.479 |  |  | 50.32 | 0.027 |  |
| **Nervous** | **Depression** | **FinnGen** | **Weighted median** | 1.05 | 0.74 | 1.50 | 0.769 |  |  |  |  |  |
| **Nervous** | **Depression** | **FinnGen** | **MR-Egger** | 0.92 | 0.34 | 2.46 | 0.862 | 0.00 | 0.976 | 50.32 | 0.021 | 0.95 |
| **Nervous** | **Depression** | **FinnGen** | **Penalised weighted median** | 1.17 | 0.81 | 1.70 | 0.396 |  |  |  |  |  |
| **Nervous** | **Depression** | **Published GWAS** | **Inverse-variance weighted** | 1.15 | 0.88 | 1.50 | 0.317 |  |  | 88.17 | 6.E-07 |  |
| **Nervous** | **Depression** | **Published GWAS** | **Weighted median** | 1.37 | 1.06 | 1.79 | 0.018 |  |  |  |  |  |
| **Nervous** | **Depression** | **Published GWAS** | **MR-Egger** | 1.62 | 0.65 | 4.05 | 0.307 | -0.01 | 0.444 | 86.55 | 6.E-07 | 0.93 |
| **Nervous** | **Depression** | **Published GWAS** | **Penalised weighted median** | 1.41 | 1.05 | 1.89 | 0.020 |  |  |  |  |  |
| **Nervous** | **Multiple sclerosis** | **FinnGen** | **Inverse-variance weighted** | 0.62 | 0.24 | 1.59 | 0.332 |  |  | 29.14 | 0.660 |  |
| **Nervous** | **Multiple sclerosis** | **FinnGen** | **Weighted median** | 0.50 | 0.13 | 2.00 | 0.326 |  |  |  |  |  |
| **Nervous** | **Multiple sclerosis** | **FinnGen** | **MR-Egger** | 0.70 | 0.03 | 17.66 | 0.828 | 0.00 | 0.942 | 29.13 | 0.613 | 0.95 |
| **Nervous** | **Multiple sclerosis** | **FinnGen** | **Penalised weighted median** | 0.50 | 0.12 | 2.06 | 0.336 |  |  |  |  |  |
| **Nervous** | **Parkinson's disease** | **FinnGen** | **Inverse-variance weighted** | 1.27 | 0.58 | 2.78 | 0.551 |  |  | 41.78 | 0.140 |  |
| **Nervous** | **Parkinson's disease** | **FinnGen** | **Weighted median** | 1.52 | 0.53 | 4.35 | 0.439 |  |  |  |  |  |
| **Nervous** | **Parkinson's disease** | **FinnGen** | **MR-Egger** | 1.41 | 0.09 | 21.96 | 0.809 | 0.00 | 0.940 | 41.78 | 0.116 | 0.95 |
| **Nervous** | **Parkinson's disease** | **FinnGen** | **Penalised weighted median** | 1.53 | 0.52 | 4.51 | 0.438 |  |  |  |  |  |
| **Nervous** | **Parkinson's disease** | **Published GWAS** | **Inverse-variance weighted** | 0.65 | 0.46 | 0.91 | 0.018 |  |  | 36.13 | 0.325 |  |
| **Nervous** | **Parkinson's disease** | **Published GWAS** | **Weighted median** | 0.86 | 0.52 | 1.44 | 0.572 |  |  |  |  |  |
| **Nervous** | **Parkinson's disease** | **Published GWAS** | **MR-Egger** | 0.93 | 0.31 | 2.83 | 0.905 | -0.01 | 0.508 | 35.63 | 0.302 | 0.94 |
| **Nervous** | **Parkinson's disease** | **Published GWAS** | **Penalised weighted median** | 0.88 | 0.52 | 1.48 | 0.632 |  |  |  |  |  |
| **Integumentary** | **Psoriasis** | **FinnGen** | **Inverse-variance weighted** | 1.95 | 1.21 | 3.12 | 0.009 |  |  | 26.73 | 0.771 |  |
| **Integumentary** | **Psoriasis** | **FinnGen** | **Weighted median** | 1.45 | 0.73 | 2.91 | 0.291 |  |  |  |  |  |
| **Integumentary** | **Psoriasis** | **FinnGen** | **MR-Egger** | 2.00 | 0.39 | 10.22 | 0.412 | 0.00 | 0.973 | 26.73 | 0.730 | 0.95 |
| **Integumentary** | **Psoriasis** | **FinnGen** | **Penalised weighted median** | 1.44 | 0.73 | 2.87 | 0.294 |  |  |  |  |  |
| **Integumentary** | **Psoriasis** | **Published GWAS** | **Inverse-variance weighted** | 2.31 | 1.38 | 3.88 | 0.003 |  |  | 76.07 | 3.E-05 |  |
| **Integumentary** | **Psoriasis** | **Published GWAS** | **Weighted median** | 2.69 | 1.55 | 4.65 | 4.E-04 |  |  |  |  |  |
| **Integumentary** | **Psoriasis** | **Published GWAS** | **MR-Egger** | 6.78 | 1.25 | 36.67 | 0.033 | -0.02 | 0.200 | 72.20 | 6.E-05 | 0.96 |
| **Integumentary** | **Psoriasis** | **Published GWAS** | **Penalised weighted median** | 2.03 | 1.11 | 3.73 | 0.022 |  |  |  |  |  |
| **Respiratory** | **Adult-onset asthma** | **Published GWAS** | **Inverse-variance weighted** | 1.14 | 0.89 | 1.46 | 0.291 |  |  | 94.43 | 2.E-07 |  |
| **Respiratory** | **Adult-onset asthma** | **Published GWAS** | **Weighted median** | 1.29 | 1.00 | 1.68 | 0.050 |  |  |  |  |  |
| **Respiratory** | **Adult-onset asthma** | **Published GWAS** | **MR-Egger** | 1.22 | 0.52 | 2.86 | 0.653 | 0.00 | 0.881 | 94.36 | 1.E-07 | 0.92 |
| **Respiratory** | **Adult-onset asthma** | **Published GWAS** | **Penalised weighted median** | 1.32 | 1.01 | 1.73 | 0.044 |  |  |  |  |  |
| **Respiratory** | **Asthma** | **FinnGen** | **Inverse-variance weighted** | 1.60 | 1.21 | 2.13 | 0.002 |  |  | 46.26 | 0.063 |  |
| **Respiratory** | **Asthma** | **FinnGen** | **Weighted median** | 1.64 | 1.12 | 2.38 | 0.010 |  |  |  |  |  |
| **Respiratory** | **Asthma** | **FinnGen** | **MR-Egger** | 2.28 | 0.85 | 6.10 | 0.111 | -0.01 | 0.471 | 45.50 | 0.057 | 0.95 |
| **Respiratory** | **Asthma** | **FinnGen** | **Penalised weighted median** | 1.54 | 1.03 | 2.32 | 0.038 |  |  |  |  |  |
| **Respiratory** | **Child-onset asthma** | **Published GWAS** | **Inverse-variance weighted** | 0.96 | 0.70 | 1.31 | 0.788 |  |  | 78.76 | 3.E-05 |  |
| **Respiratory** | **Child-onset asthma** | **Published GWAS** | **Weighted median** | 1.03 | 0.72 | 1.47 | 0.886 |  |  |  |  |  |
| **Respiratory** | **Child-onset asthma** | **Published GWAS** | **MR-Egger** | 1.15 | 0.39 | 3.38 | 0.804 | 0.00 | 0.733 | 78.49 | 2.E-05 | 0.92 |
| **Respiratory** | **Child-onset asthma** | **Published GWAS** | **Penalised weighted median** | 1.07 | 0.73 | 1.57 | 0.725 |  |  |  |  |  |
| **Cancer** | **Barrett's oesophagus** | **FinnGen** | **Inverse-variance weighted** | 0.62 | 0.14 | 2.65 | 0.522 |  |  | 26.69 | 0.773 |  |
| **Cancer** | **Barrett's oesophagus** | **FinnGen** | **Weighted median** | 1.37 | 0.17 | 11.08 | 0.768 |  |  |  |  |  |
| **Cancer** | **Barrett's oesophagus** | **FinnGen** | **MR-Egger** | 10.00 | 0.07 | 1534.24 | 0.377 | -0.06 | 0.213 | 25.40 | 0.789 | 0.95 |
| **Cancer** | **Barrett's oesophagus** | **FinnGen** | **Penalised weighted median** | 1.37 | 0.16 | 11.47 | 0.772 |  |  |  |  |  |
| **Cancer** | **Breast cancer** | **FinnGen** | **Inverse-variance weighted** | 0.48 | 0.33 | 0.70 | 5.E-04 |  |  | 31.65 | 0.534 |  |
| **Cancer** | **Breast cancer** | **FinnGen** | **Weighted median** | 0.50 | 0.29 | 0.86 | 0.012 |  |  |  |  |  |
| **Cancer** | **Breast cancer** | **FinnGen** | **MR-Egger** | 0.07 | 0.02 | 0.25 | 3.E-04 | 0.04 | 8.E-04 | 22.18 | 0.902 | 0.95 |
| **Cancer** | **Breast cancer** | **FinnGen** | **Penalised weighted median** | 0.77 | 0.45 | 1.33 | 0.352 |  |  |  |  |  |
| **Cancer** | **Breast cancer** | **Published GWAS** | **Inverse-variance weighted** | 0.56 | 0.37 | 0.87 | 0.013 |  |  | 69.27 | 0.001 |  |
| **Cancer** | **Breast cancer** | **Published GWAS** | **Weighted median** | 0.39 | 0.23 | 0.65 | 3.E-04 |  |  |  |  |  |
| **Cancer** | **Breast cancer** | **Published GWAS** | **MR-Egger** | 0.29 | 0.07 | 1.30 | 0.115 | 0.01 | 0.374 | 67.75 | 0.001 | 0.93 |
| **Cancer** | **Breast cancer** | **Published GWAS** | **Penalised weighted median** | 0.94 | 0.53 | 1.66 | 0.833 |  |  |  |  |  |
| **Cancer** | **Cancer myeloma** | **FinnGen** | **Inverse-variance weighted** | 1.33 | 0.30 | 5.99 | 0.710 |  |  | 44.95 | 0.080 |  |
| **Cancer** | **Cancer myeloma** | **FinnGen** | **Weighted median** | 1.55 | 0.19 | 12.50 | 0.682 |  |  |  |  |  |
| **Cancer** | **Cancer myeloma** | **FinnGen** | **MR-Egger** | 13.19 | 0.07 | 2410.59 | 0.339 | -0.05 | 0.374 | 43.84 | 0.079 | 0.95 |
| **Cancer** | **Cancer myeloma** | **FinnGen** | **Penalised weighted median** | 1.48 | 0.21 | 10.51 | 0.693 |  |  |  |  |  |
| **Cancer** | **Colon cancer** | **Published GWAS** | **Inverse-variance weighted** | 1.55 | 1.23 | 1.95 | 8.E-04 |  |  | 39.67 | 0.197 |  |
| **Cancer** | **Colon cancer** | **Published GWAS** | **Weighted median** | 1.49 | 1.12 | 2.00 | 0.007 |  |  |  |  |  |
| **Cancer** | **Colon cancer** | **Published GWAS** | **MR-Egger** | 1.37 | 0.62 | 3.01 | 0.446 | 0.00 | 0.748 | 39.54 | 0.169 | 0.93 |
| **Cancer** | **Colon cancer** | **Published GWAS** | **Penalised weighted median** | 1.46 | 1.07 | 2.00 | 0.018 |  |  |  |  |  |
| **Cancer** | **Colorectal cancer** | **FinnGen** | **Inverse-variance weighted** | 0.96 | 0.56 | 1.66 | 0.891 |  |  | 32.68 | 0.483 |  |
| **Cancer** | **Colorectal cancer** | **FinnGen** | **Weighted median** | 1.08 | 0.47 | 2.49 | 0.858 |  |  |  |  |  |
| **Cancer** | **Colorectal cancer** | **FinnGen** | **MR-Egger** | 4.06 | 0.61 | 26.90 | 0.156 | -0.03 | 0.118 | 30.24 | 0.556 | 0.95 |
| **Cancer** | **Colorectal cancer** | **FinnGen** | **Penalised weighted median** | 1.10 | 0.47 | 2.59 | 0.826 |  |  |  |  |  |
| **Cancer** | **Colorectal cancer** | **Published GWAS (1)** | **Inverse-variance weighted** | 1.08 | 0.82 | 1.43 | 0.576 |  |  | 69.95 | 6.E-04 |  |
| **Cancer** | **Colorectal cancer** | **Published GWAS (1)** | **Weighted median** | 0.97 | 0.70 | 1.36 | 0.881 |  |  |  |  |  |
| **Cancer** | **Colorectal cancer** | **Published GWAS (1)** | **MR-Egger** | 1.40 | 0.54 | 3.63 | 0.491 | -0.01 | 0.582 | 69.33 | 5.E-04 | 0.94 |
| **Cancer** | **Colorectal cancer** | **Published GWAS (1)** | **Penalised weighted median** | 1.06 | 0.76 | 1.50 | 0.719 |  |  |  |  |  |
| **Cancer** | **Colorectal cancer** | **Published GWAS (2)** | **Inverse-variance weighted** | 1.39 | 1.12 | 1.73 | 0.006 |  |  | 50.26 | 0.028 |  |
| **Cancer** | **Colorectal cancer** | **Published GWAS (2)** | **Weighted median** | 1.32 | 1.02 | 1.72 | 0.036 |  |  |  |  |  |
| **Cancer** | **Colorectal cancer** | **Published GWAS (2)** | **MR-Egger** | 1.10 | 0.52 | 2.30 | 0.805 | 0.01 | 0.520 | 49.60 | 0.024 | 0.93 |
| **Cancer** | **Colorectal cancer** | **Published GWAS (2)** | **Penalised weighted median** | 1.25 | 0.91 | 1.71 | 0.170 |  |  |  |  |  |
| **Cancer** | **Distal colon cancer** | **Published GWAS** | **Inverse-variance weighted** | 1.42 | 1.08 | 1.87 | 0.018 |  |  | 32.04 | 0.515 |  |
| **Cancer** | **Distal colon cancer** | **Published GWAS** | **Weighted median** | 1.31 | 0.88 | 1.95 | 0.187 |  |  |  |  |  |
| **Cancer** | **Distal colon cancer** | **Published GWAS** | **MR-Egger** | 1.85 | 0.73 | 4.66 | 0.201 | -0.01 | 0.558 | 31.69 | 0.482 | 0.93 |
| **Cancer** | **Distal colon cancer** | **Published GWAS** | **Penalised weighted median** | 1.28 | 0.84 | 1.94 | 0.245 |  |  |  |  |  |
| **Cancer** | **Endometrial cancer** | **FinnGen** | **Inverse-variance weighted** | 0.70 | 0.23 | 2.14 | 0.542 |  |  | 49.84 | 0.030 |  |
| **Cancer** | **Endometrial cancer** | **FinnGen** | **Weighted median** | 0.41 | 0.10 | 1.60 | 0.196 |  |  |  |  |  |
| **Cancer** | **Endometrial cancer** | **FinnGen** | **MR-Egger** | 24.20 | 0.60 | 969.66 | 0.100 | -0.08 | 0.059 | 44.49 | 0.070 | 0.95 |
| **Cancer** | **Endometrial cancer** | **FinnGen** | **Penalised weighted median** | 0.40 | 0.10 | 1.65 | 0.206 |  |  |  |  |  |
| **Cancer** | **Endometrial cancer** | **Published GWAS** | **Inverse-variance weighted** | 2.48 | 1.67 | 3.69 | 7.E-05 |  |  | 72.08 | 2.E-04 |  |
| **Cancer** | **Endometrial cancer** | **Published GWAS** | **Weighted median** | 3.06 | 1.95 | 4.80 | 1.E-06 |  |  |  |  |  |
| **Cancer** | **Endometrial cancer** | **Published GWAS** | **MR-Egger** | 2.92 | 0.74 | 11.50 | 0.134 | 0.00 | 0.809 | 71.95 | 2.E-04 | 0.93 |
| **Cancer** | **Endometrial cancer** | **Published GWAS** | **Penalised weighted median** | 1.88 | 1.21 | 2.93 | 0.005 |  |  |  |  |  |
| **Cancer** | **Lung cancer** | **FinnGen** | **Inverse-variance weighted** | 1.36 | 0.65 | 2.84 | 0.426 |  |  | 34.91 | 0.378 |  |
| **Cancer** | **Lung cancer** | **FinnGen** | **Weighted median** | 0.67 | 0.23 | 1.92 | 0.457 |  |  |  |  |  |
| **Cancer** | **Lung cancer** | **FinnGen** | **MR-Egger** | 0.18 | 0.01 | 2.15 | 0.184 | 0.04 | 0.105 | 32.11 | 0.461 | 0.95 |
| **Cancer** | **Lung cancer** | **FinnGen** | **Penalised weighted median** | 0.67 | 0.23 | 1.99 | 0.472 |  |  |  |  |  |
| **Cancer** | **Meningioma** | **FinnGen** | **Inverse-variance weighted** | 0.36 | 0.15 | 0.86 | 0.028 |  |  | 31.24 | 0.555 |  |
| **Cancer** | **Meningioma** | **FinnGen** | **Weighted median** | 0.36 | 0.09 | 1.35 | 0.128 |  |  |  |  |  |
| **Cancer** | **Meningioma** | **FinnGen** | **MR-Egger** | 0.30 | 0.01 | 6.00 | 0.433 | 0.00 | 0.893 | 31.23 | 0.506 | 0.95 |
| **Cancer** | **Meningioma** | **FinnGen** | **Penalised weighted median** | 0.39 | 0.10 | 1.55 | 0.180 |  |  |  |  |  |
| **Cancer** | **Ovarian cancer** | **FinnGen** | **Inverse-variance weighted** | 1.14 | 0.39 | 3.34 | 0.819 |  |  | 29.32 | 0.651 |  |
| **Cancer** | **Ovarian cancer** | **FinnGen** | **Weighted median** | 0.92 | 0.18 | 4.62 | 0.915 |  |  |  |  |  |
| **Cancer** | **Ovarian cancer** | **FinnGen** | **MR-Egger** | 7.73 | 0.19 | 321.65 | 0.291 | -0.04 | 0.270 | 28.21 | 0.659 | 0.95 |
| **Cancer** | **Ovarian cancer** | **FinnGen** | **Penalised weighted median** | 0.94 | 0.19 | 4.63 | 0.936 |  |  |  |  |  |
| **Cancer** | **Ovarian cancer - clear cell cancer** | **Published GWAS** | **Inverse-variance weighted** | 1.21 | 0.59 | 2.48 | 0.605 |  |  | 38.08 | 0.420 |  |
| **Cancer** | **Ovarian cancer - clear cell cancer** | **Published GWAS** | **Weighted median** | 1.92 | 0.70 | 5.28 | 0.204 |  |  |  |  |  |
| **Cancer** | **Ovarian cancer - clear cell cancer** | **Published GWAS** | **MR-Egger** | 14.71 | 1.30 | 166.03 | 0.036 | -0.05 | 0.035 | 33.62 | 0.582 | 0.92 |
| **Cancer** | **Ovarian cancer - clear cell cancer** | **Published GWAS** | **Penalised weighted median** | 1.86 | 0.61 | 5.60 | 0.273 |  |  |  |  |  |
| **Cancer** | **Ovarian cancer - endometrioid** | **Published GWAS** | **Inverse-variance weighted** | 1.85 | 0.95 | 3.58 | 0.077 |  |  | 63.30 | 0.005 |  |
| **Cancer** | **Ovarian cancer - endometrioid** | **Published GWAS** | **Weighted median** | 1.75 | 0.76 | 4.03 | 0.193 |  |  |  |  |  |
| **Cancer** | **Ovarian cancer - endometrioid** | **Published GWAS** | **MR-Egger** | 5.66 | 0.58 | 54.83 | 0.144 | -0.02 | 0.320 | 61.56 | 0.005 | 0.92 |
| **Cancer** | **Ovarian cancer - endometrioid** | **Published GWAS** | **Penalised weighted median** | 1.72 | 0.73 | 4.05 | 0.214 |  |  |  |  |  |
| **Cancer** | **Ovarian cancer - epithelial** | **Published GWAS** | **Inverse-variance weighted** | 1.29 | 0.78 | 2.13 | 0.323 |  |  | 37.19 | 0.460 |  |
| **Cancer** | **Ovarian cancer - epithelial** | **Published GWAS** | **Weighted median** | 1.34 | 0.64 | 2.79 | 0.435 |  |  |  |  |  |
| **Cancer** | **Ovarian cancer - epithelial** | **Published GWAS** | **MR-Egger** | 3.08 | 0.56 | 17.10 | 0.206 | -0.02 | 0.305 | 36.10 | 0.464 | 0.92 |
| **Cancer** | **Ovarian cancer - epithelial** | **Published GWAS** | **Penalised weighted median** | 1.35 | 0.65 | 2.78 | 0.419 |  |  |  |  |  |
| **Cancer** | **Ovarian cancer - high grade serous** | **Published GWAS** | **Inverse-variance weighted** | 0.97 | 0.68 | 1.39 | 0.870 |  |  | 24.57 | 0.942 |  |
| **Cancer** | **Ovarian cancer - high grade serous** | **Published GWAS** | **Weighted median** | 1.30 | 0.76 | 2.21 | 0.342 |  |  |  |  |  |
| **Cancer** | **Ovarian cancer - high grade serous** | **Published GWAS** | **MR-Egger** | 2.34 | 0.69 | 7.97 | 0.183 | -0.02 | 0.071 | 22.41 | 0.963 | 0.92 |
| **Cancer** | **Ovarian cancer - high grade serous** | **Published GWAS** | **Penalised weighted median** | 1.30 | 0.76 | 2.23 | 0.334 |  |  |  |  |  |
| **Cancer** | **Ovarian cancer - invasive epithelial** | **Published GWAS** | **Inverse-variance weighted** | 1.27 | 0.96 | 1.68 | 0.097 |  |  | 52.26 | 0.049 |  |
| **Cancer** | **Ovarian cancer - invasive epithelial** | **Published GWAS** | **Weighted median** | 1.36 | 0.95 | 1.94 | 0.095 |  |  |  |  |  |
| **Cancer** | **Ovarian cancer - invasive epithelial** | **Published GWAS** | **MR-Egger** | 1.97 | 0.76 | 5.10 | 0.173 | -0.01 | 0.357 | 51.02 | 0.050 | 0.92 |
| **Cancer** | **Ovarian cancer - invasive epithelial** | **Published GWAS** | **Penalised weighted median** | 1.37 | 0.95 | 1.98 | 0.091 |  |  |  |  |  |
| **Cancer** | **Ovarian cancer - low grade** | **Published GWAS** | **Inverse-variance weighted** | 1.31 | 0.55 | 3.14 | 0.547 |  |  | 39.61 | 0.354 |  |
| **Cancer** | **Ovarian cancer - low grade** | **Published GWAS** | **Weighted median** | 1.27 | 0.34 | 4.68 | 0.720 |  |  |  |  |  |
| **Cancer** | **Ovarian cancer - low grade** | **Published GWAS** | **MR-Egger** | 2.40 | 0.12 | 49.68 | 0.575 | -0.01 | 0.685 | 39.43 | 0.319 | 0.92 |
| **Cancer** | **Ovarian cancer - low grade** | **Published GWAS** | **Penalised weighted median** | 1.29 | 0.36 | 4.66 | 0.693 |  |  |  |  |  |
| **Cancer** | **Ovarian cancer - mucinous ovarian carcinoma** | **Published GWAS** | **Inverse-variance weighted** | 1.69 | 0.99 | 2.86 | 0.061 |  |  | 30.44 | 0.768 |  |
| **Cancer** | **Ovarian cancer - mucinous ovarian carcinoma** | **Published GWAS** | **Weighted median** | 1.74 | 0.79 | 3.82 | 0.166 |  |  |  |  |  |
| **Cancer** | **Ovarian cancer - mucinous ovarian carcinoma** | **Published GWAS** | **MR-Egger** | 1.92 | 0.31 | 11.83 | 0.486 | 0.00 | 0.873 | 30.42 | 0.731 | 0.92 |
| **Cancer** | **Ovarian cancer - mucinous ovarian carcinoma** | **Published GWAS** | **Penalised weighted median** | 1.74 | 0.78 | 3.87 | 0.177 |  |  |  |  |  |
| **Cancer** | **Ovarian cancer - serous invasive** | **Published GWAS** | **Inverse-variance weighted** | 1.08 | 0.81 | 1.43 | 0.614 |  |  | 40.91 | 0.303 |  |
| **Cancer** | **Ovarian cancer - serous invasive** | **Published GWAS** | **Weighted median** | 1.18 | 0.78 | 1.79 | 0.431 |  |  |  |  |  |
| **Cancer** | **Ovarian cancer - serous invasive** | **Published GWAS** | **MR-Egger** | 1.17 | 0.44 | 3.14 | 0.753 | 0.00 | 0.859 | 40.87 | 0.265 | 0.92 |
| **Cancer** | **Ovarian cancer - serous invasive** | **Published GWAS** | **Penalised weighted median** | 1.21 | 0.79 | 1.86 | 0.385 |  |  |  |  |  |
| **Cancer** | **Ovarian cancer - serous lowgrade borderline** | **Published GWAS** | **Inverse-variance weighted** | 1.39 | 0.75 | 2.58 | 0.305 |  |  | 55.22 | 0.027 |  |
| **Cancer** | **Ovarian cancer - serous lowgrade borderline** | **Published GWAS** | **Weighted median** | 2.37 | 1.10 | 5.11 | 0.028 |  |  |  |  |  |
| **Cancer** | **Ovarian cancer - serous lowgrade borderline** | **Published GWAS** | **MR-Egger** | 5.62 | 0.69 | 45.72 | 0.115 | -0.03 | 0.180 | 52.50 | 0.037 | 0.92 |
| **Cancer** | **Ovarian cancer - serous lowgrade borderline** | **Published GWAS** | **Penalised weighted median** | 1.89 | 0.87 | 4.12 | 0.107 |  |  |  |  |  |
| **Cancer** | **Pancreatic cancer** | **FinnGen** | **Inverse-variance weighted** | 1.45 | 0.45 | 4.61 | 0.536 |  |  | 28.33 | 0.699 |  |
| **Cancer** | **Pancreatic cancer** | **FinnGen** | **Weighted median** | 1.60 | 0.27 | 9.48 | 0.605 |  |  |  |  |  |
| **Cancer** | **Pancreatic cancer** | **FinnGen** | **MR-Egger** | 2.37 | 0.04 | 129.97 | 0.675 | -0.01 | 0.790 | 28.27 | 0.656 | 0.95 |
| **Cancer** | **Pancreatic cancer** | **FinnGen** | **Penalised weighted median** | 1.64 | 0.27 | 10.03 | 0.593 |  |  |  |  |  |
| **Cancer** | **Prostate cancer** | **FinnGen** | **Inverse-variance weighted** | 1.19 | 0.70 | 2.03 | 0.531 |  |  | 51.03 | 0.023 |  |
| **Cancer** | **Prostate cancer** | **FinnGen** | **Weighted median** | 1.02 | 0.53 | 1.98 | 0.952 |  |  |  |  |  |
| **Cancer** | **Prostate cancer** | **FinnGen** | **MR-Egger** | 0.35 | 0.06 | 2.19 | 0.272 | 0.03 | 0.183 | 48.24 | 0.033 | 0.95 |
| **Cancer** | **Prostate cancer** | **FinnGen** | **Penalised weighted median** | 1.06 | 0.54 | 2.08 | 0.867 |  |  |  |  |  |
| **Cancer** | **Prostate cancer** | **Published GWAS** | **Inverse-variance weighted** | 0.91 | 0.73 | 1.13 | 0.383 |  |  | 88.77 | 4.E-06 |  |
| **Cancer** | **Prostate cancer** | **Published GWAS** | **Weighted median** | 0.74 | 0.59 | 0.94 | 0.014 |  |  |  |  |  |
| **Cancer** | **Prostate cancer** | **Published GWAS** | **MR-Egger** | 0.49 | 0.23 | 1.04 | 0.070 | 0.01 | 0.103 | 82.36 | 2.E-05 | 0.92 |
| **Cancer** | **Prostate cancer** | **Published GWAS** | **Penalised weighted median** | 1.06 | 0.82 | 1.37 | 0.653 |  |  |  |  |  |
| **Cancer** | **Proximal colon cancer** | **Published GWAS** | **Inverse-variance weighted** | 1.64 | 1.25 | 2.16 | 0.001 |  |  | 34.92 | 0.377 |  |
| **Cancer** | **Proximal colon cancer** | **Published GWAS** | **Weighted median** | 1.44 | 0.99 | 2.09 | 0.058 |  |  |  |  |  |
| **Cancer** | **Proximal colon cancer** | **Published GWAS** | **MR-Egger** | 1.35 | 0.53 | 3.43 | 0.536 | 0.00 | 0.669 | 34.72 | 0.340 | 0.93 |
| **Cancer** | **Proximal colon cancer** | **Published GWAS** | **Penalised weighted median** | 1.39 | 0.92 | 2.11 | 0.119 |  |  |  |  |  |
| **Cancer** | **Rectal cancer** | **Published GWAS** | **Inverse-variance weighted** | 1.25 | 0.95 | 1.65 | 0.121 |  |  | 28.49 | 0.691 |  |
| **Cancer** | **Rectal cancer** | **Published GWAS** | **Weighted median** | 1.21 | 0.81 | 1.80 | 0.348 |  |  |  |  |  |
| **Cancer** | **Rectal cancer** | **Published GWAS** | **MR-Egger** | 1.23 | 0.49 | 3.11 | 0.662 | 0.00 | 0.970 | 28.49 | 0.645 | 0.93 |
| **Cancer** | **Rectal cancer** | **Published GWAS** | **Penalised weighted median** | 1.19 | 0.80 | 1.77 | 0.399 |  |  |  |  |  |
| **Cancer** | **Renal cancer** | **FinnGen** | **Inverse-variance weighted** | 1.40 | 0.51 | 3.81 | 0.516 |  |  | 36.39 | 0.314 |  |
| **Cancer** | **Renal cancer** | **FinnGen** | **Weighted median** | 2.15 | 0.50 | 9.27 | 0.306 |  |  |  |  |  |
| **Cancer** | **Renal cancer** | **FinnGen** | **MR-Egger** | 0.92 | 0.03 | 30.76 | 0.962 | 0.01 | 0.808 | 36.32 | 0.274 | 0.95 |
| **Cancer** | **Renal cancer** | **FinnGen** | **Penalised weighted median** | 2.25 | 0.57 | 8.80 | 0.246 |  |  |  |  |  |
| **Cancer** | **Renal cancer** | **Published GWAS** | **Inverse-variance weighted** | 1.78 | 1.18 | 2.68 | 0.010 |  |  | 47.48 | 0.049 |  |
| **Cancer** | **Renal cancer** | **Published GWAS** | **Weighted median** | 1.88 | 1.10 | 3.20 | 0.020 |  |  |  |  |  |
| **Cancer** | **Renal cancer** | **Published GWAS** | **MR-Egger** | 2.84 | 0.71 | 11.39 | 0.151 | -0.01 | 0.494 | 46.78 | 0.044 | 0.93 |
| **Cancer** | **Renal cancer** | **Published GWAS** | **Penalised weighted median** | 1.36 | 0.78 | 2.38 | 0.277 |  |  |  |  |  |
| **Cancer** | **Thyroid cancer** | **FinnGen** | **Inverse-variance weighted** | 1.09 | 0.42 | 2.86 | 0.859 |  |  | 34.77 | 0.384 |  |
| **Cancer** | **Thyroid cancer** | **FinnGen** | **Weighted median** | 0.60 | 0.15 | 2.44 | 0.478 |  |  |  |  |  |
| **Cancer** | **Thyroid cancer** | **FinnGen** | **MR-Egger** | 0.54 | 0.02 | 15.70 | 0.723 | 0.02 | 0.672 | 34.57 | 0.346 | 0.95 |
| **Cancer** | **Thyroid cancer** | **FinnGen** | **Penalised weighted median** | 0.60 | 0.15 | 2.36 | 0.466 |  |  |  |  |  |

**Supplementary File 1h. The inverse-variance weighted MR analysis of identified diseases from UK Biobank for body mass index (BMI), body fat percentage, "favourable adiposity" (FA) and "unfavourable adiposity" (UFA) clusters. OR: Odds ratio; LCI: lower 95% confidence interval; UCI: upper 95% confidence interval; P: p-value.**

|  | | **BMI** | | | | **Body fat percentage** | | | | **FA** | | | | **UFA** | | | |
| --- | --- | --- | --- | --- | --- | --- | --- | --- | --- | --- | --- | --- | --- | --- | --- | --- | --- |
| **System** | **Disease** | **OR** | **LCI** | **UCI** | **P** | **OR** | **LCI** | **UCI** | **P** | **OR** | **LCI** | **UCI** | **P** | **OR** | **LCI** | **UCI** | **P** |
| **Cardiovascular and metabolic** | **Atrial fibrillation** | 1.20 | 0.93 | 1.54 | 0.159 | 1.24 | 1.03 | 1.48 | 0.020 | 1.15 | 0.49 | 2.69 | 0.753 | 1.37 | 0.92 | 2.06 | 0.134 |
| **Cardiovascular and metabolic** | **Coronary artery disease** | 1.30 | 1.13 | 1.50 | 4.E-04 | 1.72 | 1.60 | 1.85 | 2.E-42 | 0.53 | 0.39 | 0.72 | 3.E-04 | 1.61 | 1.31 | 1.98 | 6.E-05 |
| **Cardiovascular and metabolic** | **Deep vein thrombosis** | 1.66 | 1.41 | 1.94 | 3.E-08 | 1.75 | 1.52 | 2.01 | 3.E-14 | 1.84 | 1.16 | 2.92 | 0.014 | 1.82 | 1.26 | 2.63 | 0.003 |
| **Cardiovascular and metabolic** | **Hypertension** | 1.67 | 1.46 | 1.90 | 8.E-11 | 1.93 | 1.81 | 2.06 | 4.E-68 | 0.57 | 0.42 | 0.77 | 8.E-04 | 1.99 | 1.60 | 2.49 | 5.E-07 |
| **Cardiovascular and metabolic** | **Peripheral vascular disease** | 1.26 | 1.03 | 1.55 | 0.029 | 1.67 | 1.45 | 1.91 | 1.E-12 | 0.67 | 0.39 | 1.13 | 0.140 | 2.08 | 1.38 | 3.13 | 0.001 |
| **Cardiovascular and metabolic** | **Pulmonary embolism** | 1.70 | 1.39 | 2.07 | 2.E-06 | 1.82 | 1.57 | 2.12 | 3.E-14 | 2.00 | 1.28 | 3.11 | 0.004 | 2.00 | 1.29 | 3.12 | 0.004 |
| **Cardiovascular and metabolic** | **Stroke** | 1.06 | 0.92 | 1.23 | 0.410 | 1.54 | 1.40 | 1.70 | 3.E-17 | 0.75 | 0.51 | 1.11 | 0.162 | 1.59 | 1.18 | 2.15 | 0.004 |
| **Cardiovascular and metabolic** | **Polycystic ovary syndrome** | 3.27 | 1.77 | 6.03 | 3.E-04 | 2.58 | 1.77 | 3.76 | 1.E-06 | 0.18 | 0.05 | 0.72 | 0.021 | 7.08 | 2.72 | 18.40 | 3.E-04 |
| **Cardiovascular and metabolic** | **Type 2 diabetes** | 3.90 | 3.24 | 4.70 | 6.E-23 | 3.66 | 3.11 | 4.31 | 2.E-47 | 0.10 | 0.06 | 0.20 | 3.E-08 | 6.98 | 4.96 | 9.83 | 2.E-13 |
| **Cardiovascular and metabolic** | **Chronic kidney disease** | 1.52 | 1.27 | 1.82 | 2.E-05 | 1.82 | 1.61 | 2.06 | 7.E-20 | 0.71 | 0.47 | 1.07 | 0.112 | 1.91 | 1.43 | 2.55 | 8.E-05 |
| **Musculoskeletal** | **Gout** | 1.57 | 1.28 | 1.93 | 6.E-05 | 1.84 | 1.60 | 2.11 | 2.E-17 | 0.29 | 0.18 | 0.47 | 2.E-05 | 2.64 | 1.83 | 3.82 | 8.E-06 |
| **Musculoskeletal** | **Osteoarthritis** | 1.52 | 1.40 | 1.64 | 1.E-15 | 1.72 | 1.63 | 1.82 | 2.E-66 | 1.38 | 1.12 | 1.69 | 0.005 | 1.85 | 1.55 | 2.22 | 6.E-08 |
| **Musculoskeletal** | **Osteoporosis** | 0.73 | 0.63 | 0.84 | 6.E-05 | 0.80 | 0.72 | 0.89 | 3.E-05 | 1.55 | 0.92 | 2.61 | 0.105 | 0.70 | 0.52 | 0.95 | 0.027 |
| **Musculoskeletal** | **Rheumatoid arthritis** | 1.40 | 1.15 | 1.70 | 0.001 | 1.77 | 1.56 | 2.00 | 4.E-18 | 1.61 | 1.01 | 2.56 | 0.055 | 1.66 | 1.19 | 2.31 | 0.005 |
| **Gastrointestinal** | **Gallstones** | 1.78 | 1.58 | 2.02 | 7.E-14 | 2.30 | 2.11 | 2.50 | 2.E-66 | 1.37 | 0.95 | 1.99 | 0.103 | 2.35 | 1.89 | 2.93 | 4.E-09 |
| **Gastrointestinal** | **Gastro-oesophageal reflux disease** | 1.05 | 0.95 | 1.17 | 0.325 | 1.46 | 1.38 | 1.56 | 2.E-31 | 1.15 | 0.93 | 1.43 | 0.195 | 1.19 | 0.99 | 1.42 | 0.068 |
| **Nervous** | **Depression** | 1.12 | 0.99 | 1.26 | 0.070 | 1.27 | 1.18 | 1.36 | 4.E-11 | 1.15 | 0.86 | 1.53 | 0.352 | 1.34 | 1.12 | 1.60 | 0.003 |
| **Nervous** | **Multiple sclerosis** | 0.87 | 0.59 | 1.30 | 0.501 | 1.05 | 0.81 | 1.38 | 0.703 | 1.60 | 0.58 | 4.43 | 0.375 | 0.75 | 0.39 | 1.47 | 0.413 |
| **Nervous** | **Parkinson's disease** | 0.94 | 0.65 | 1.36 | 0.745 | 0.94 | 0.74 | 1.20 | 0.615 | 0.89 | 0.38 | 2.07 | 0.780 | 0.78 | 0.44 | 1.37 | 0.385 |
| **Integumentary** | **Psoriasis** | 1.47 | 1.22 | 1.78 | 2.E-04 | 1.58 | 1.36 | 1.82 | 2.E-09 | 1.09 | 0.64 | 1.87 | 0.745 | 1.62 | 1.08 | 2.43 | 0.024 |
| **Respiratory** | **Asthma** | 1.23 | 1.12 | 1.35 | 5.E-05 | 1.41 | 1.31 | 1.51 | 7.E-21 | 0.93 | 0.69 | 1.26 | 0.664 | 1.28 | 1.04 | 1.58 | 0.025 |
| **Cancer** | **Barrett's oesophagus** | 1.31 | 1.04 | 1.65 | 0.026 | 1.64 | 1.41 | 1.91 | 4.E-10 | 1.33 | 0.78 | 2.27 | 0.299 | 1.49 | 1.02 | 2.19 | 0.047 |
| **Cancer** | **Breast cancer** | 0.74 | 0.61 | 0.89 | 0.002 | 1.02 | 0.91 | 1.13 | 0.769 | 0.74 | 0.42 | 1.33 | 0.324 | 0.77 | 0.56 | 1.05 | 0.105 |
| **Cancer** | **Colorectal cancer** | 1.02 | 0.81 | 1.28 | 0.884 | 0.95 | 0.82 | 1.09 | 0.473 | 0.66 | 0.39 | 1.11 | 0.123 | 0.88 | 0.63 | 1.22 | 0.439 |
| **Cancer** | **Endometrial cancer** | 1.43 | 0.99 | 2.07 | 0.063 | 1.42 | 1.12 | 1.80 | 0.004 | 1.39 | 0.57 | 3.37 | 0.474 | 1.32 | 0.74 | 2.36 | 0.353 |
| **Cancer** | **Lung cancer** | 0.96 | 0.71 | 1.30 | 0.803 | 1.35 | 1.10 | 1.66 | 0.005 | 0.51 | 0.24 | 1.06 | 0.080 | 1.23 | 0.74 | 2.05 | 0.436 |
| **Cancer** | **Ovarian cancer** | 1.15 | 0.76 | 1.74 | 0.506 | 1.06 | 0.80 | 1.41 | 0.687 | 0.34 | 0.12 | 0.92 | 0.041 | 1.09 | 0.51 | 2.34 | 0.827 |
| **Cancer** | **Pancreatic cancer** | 1.88 | 1.12 | 3.15 | 0.019 | 1.37 | 0.97 | 1.94 | 0.071 | 0.68 | 0.15 | 2.98 | 0.610 | 1.51 | 0.65 | 3.50 | 0.339 |
| **Cancer** | **Prostate cancer** | 0.81 | 0.67 | 0.97 | 0.025 | 0.90 | 0.79 | 1.02 | 0.096 | 0.75 | 0.46 | 1.24 | 0.273 | 0.91 | 0.63 | 1.31 | 0.612 |
| **Cancer** | **Renal cancer** | 0.92 | 0.58 | 1.43 | 0.700 | 1.19 | 0.91 | 1.56 | 0.196 | 0.99 | 0.36 | 2.71 | 0.979 | 1.03 | 0.52 | 2.07 | 0.925 |
| **Cancer** | **Thyroid cancer** | 0.93 | 0.50 | 1.74 | 0.830 | 1.46 | 0.97 | 2.19 | 0.068 | 1.62 | 0.25 | 10.46 | 0.613 | 1.00 | 0.32 | 3.10 | 0.994 |
