# Supplementary material for: Disease consequences of higher adiposity uncoupled from its adverse metabolic effects using Mendelian randomisation
Source: eLife. 2022 Jan 25;11:e72452. doi: 10.7554/eLife.72452 (PMC8789289; doi:10.7554/eLife.72452)
Supplement: Transparent reporting form [file elife-72452-transrepform1.docx]

***eLife’s* transparent reporting form**

We encourage authors to provide detailed information *within their submission* to facilitate the interpretation and replication of experiments. Authors can upload supporting documentation to indicate the use of appropriate reporting guidelines for health-related research (see [EQUATOR Network](http://www.equator-network.org/%20)), life science research (see the [BioSharing Information Resource](https://biosharing.org/)), or the [ARRIVE guidelines](http://www.plosbiology.org/article/info:doi/10.1371/journal.pbio.1000412) for reporting work involving animal research. Where applicable, authors should refer to any relevant reporting standards documents in this form.

If you have any questions, please consult our Journal Policies and/or contact us: [editorial@elifesciences.org](mailto:editorial@elifesciences.org).

**Sample-size estimation**

- You should state whether an appropriate sample size was computed when the study was being designed
- You should state the statistical method of sample size computation and any required assumptions
- If no explicit power analysis was used, you should describe how you decided what sample (replicate) size (number) to use

Please outline where this information can be found within the submission (e.g., sections or figure legends), or explain why this information doesn’t apply to your submission:

We did not perform sample size calculations because we used published GWAS in this study (published GWAS, FinnGen and UK Biobank) and had fixed sample sizes but the studies we used have the largest sample size for each disease outcome.

**Replicates**

- You should report how often each experiment was performed
- You should include a definition of biological versus technical replication
- The data obtained should be provided and sufficient information should be provided to indicate the number of independent biological and/or technical replicates
- If you encountered any outliers, you should describe how these were handled
- Criteria for exclusion/inclusion of data should be clearly stated
- High-throughput sequence data should be uploaded before submission, with a private link for reviewers provided (these are available from both GEO and ArrayExpress)

Please outline where this information can be found within the submission (e.g., sections or figure legends), or explain why this information doesn’t apply to your submission:

A flowchart of the study design is provided in Figure 1.

Inclusion/exclusion criteria for the GWAS of UK Biobank participants is given in the Methods section on page 9.

Details of how disease outcomes were identified are given in the Methods section on page 8, and details of the selection of genetic variants used as proxies for the four exposures are given in the Methods section on pages 9-10.

Definitions of disease outcomes used in the MR analyses are given in Supplementary File 1ci-iii.

**Statistical reporting**

- Statistical analysis methods should be described and justified
- Raw data should be presented in figures whenever informative to do so (typically when N per group is less than 10)
- For each experiment, you should identify the statistical tests used, exact values of N, definitions of center, methods of multiple test correction, and dispersion and precision measures (e.g., mean, median, SD, SEM, confidence intervals; and, for the major substantive results, a measure of effect size (e.g., Pearson's r, Cohen's d)
- Report exact p-values wherever possible alongside the summary statistics and 95% confidence intervals. These should be reported for all key questions and not only when the p-value is less than 0.05.

Please outline where this information can be found within the submission (e.g., sections or figure legends), or explain why this information doesn’t apply to your submission:

Details of the statistical analyses are given for the GWAS of UK Biobank participants in the Methods section on page 9, and for the MR analyses in the Methods section on pages 10-11.

Raw data detailing sample sizes and summary statistics of GWAS used are given in Supplementary File 1ci-iii.

Exact p-values, summary statistics and confidence intervals are reported for all MR analyses in Figure 2a-k, Supplementary File 1e-h and Figure 2 – figure supplement 1a-f.

(For large datasets, or papers with a very large number of statistical tests, you may upload a single table file with tests, Ns, etc., with reference to sections in the manuscript.)

**Group allocation**

- Indicate how samples were allocated into experimental groups (in the case of clinical studies, please specify allocation to treatment method); if randomization was used, please also state if restricted randomization was applied
- Indicate if masking was used during group allocation, data collection and/or data analysis

Please outline where this information can be found within the submission (e.g., sections or figure legends), or explain why this information doesn’t apply to your submission:

Not applicable – in this study design we did not allocate samples into groups.

**Additional data files (“source data”)**

- We encourage you to upload relevant additional data files, such as numerical data that are represented as a graph in a figure, or as a summary table
- Where provided, these should be in the most useful format, and they can be uploaded as “Source data” files linked to a main figure or table
- Include model definition files including the full list of parameters used
- Include code used for data analysis (e.g., R, MatLab)
- Avoid stating that data files are “available upon request”

Please indicate the figures or tables for which source data files have been provided:

All MR results and genetic variants used as proxies for the four exposures are given in Supplementary File 1d-h.

GWAS data from the outcome diseases studied is available from links published in the original studies (Supplementary File 1ci). FinnGen data is available at: <https://finngen.gitbook.io/documentation/>. UK Biobank data is available by application to the UK Biobank: <https://www.ukbiobank.ac.uk>.

Code used to conduct this analysis will be made available on GitHub after removing any sensitive information (https://github.com/susiemartin).
